# Quantifying and Improving Stereo Camera Calibration Robustness: An Outlier-Aware Algorithm for Digital Twin Data Acquisition

**DOI:** 10.3390/jimaging12070280

**Published:** 2026-06-25

**Authors:** Madalina Carbureanu, Florin-Stefan Zamfir

**Affiliations:** Department of Automatic Control, Computers, and Electronics, Faculty of Mechanical and Electrical Engineering, Petroleum-Gas University of Ploiesti, 100680 Ploiesti, Romania; mcarbureanu@upg-ploiesti.ro

**Keywords:** geometric consistency, reprojection error, stereo rectification, outlier rejection, robust subset selection, epipolar error

## Abstract

As calibration errors have a direct impact on epipolar consistency, rectification accuracy, and metric 3D reconstruction performance, stereo camera calibration is a fundamental requirement for high-accuracy 3D modeling and reliable digital twin data acquisition. Because current calibration workflows (based on pairwise calibration methods) lack systematic data-quality checks mechanisms, there is a clear need for more robust data selection strategies. The novelty of the approach consists in the development of a new outlier-aware stereo calibration algorithm (OutAw) that introduces a unified multi-stage approach that integrates hard geometric selection, candidate subset generation, multi-criterion ranking, bootstrap stability analysis, and triangulation assessment into a comprehensive and systematic calibration framework. Unlike conventional approaches, OutAw (through its mechanism of detecting and rejecting inconsistent pairs) redefines the calibration strategy from arbitrary to criterion-based data selection. Also, the proposed algorithm is compared with BSC (a baseline OpenCV all-pairs calibration algorithm) and InterFil (an intermediate filtered variant) using 49 stereo pairs (at 1280 × 720 resolution) captured using a planar checkerboard. OutAw algorithm achieved (using only nine image pairs) superior results (epipolar error 0.5119 px, stereo RMS 0.7666 px) to the BSC ones (epipolar error 1.3687 px, stereo RMS 1.9385 px), representing statistically significant improvements (60.5%, respectively 62.3%). OutAw geometric consistency was validated by triangulation-based metrics (square-length standard deviation 0.1140 mm and square absolute error 0.1097 mm). Contamination analysis revealed that as the outlier rate increases, the calibration process degrades progressively. Also, the results obtained highlight that geometric quality-driven image selection is critical for achieving a reliable stereo calibration for DT applications.

## 1. Introduction

Widely used in digital twin (DT) applications, robotics, and metrology, and not only, stereo camera calibration enables accurate 3D reconstruction from pairs of images, a reconstruction that depends on the camera calibration (intrinsic parameters), the stereo pair geometric relationship, and the lens distortion. Usually in DT systems, the calibration errors are inserted into the model, leading to incorrect spatial alignment, biased depth, and incorrect spatial reconstruction.

The conventional stereo calibration methods use planar checkerboard targets and standard optimization algorithms in order to estimate camera parameters from multiple image acquisitions. These approaches are robust when sharp and spatially varied calibration images are used, but are ineffective when the used dataset contains images with motion blur or defocus, weak checkerboard detection, or images with insufficient pose variation [1,2,3,4,5].

In the present paper, the calibration performance (specific features) problem was analyzed starting from a standard OpenCV-based stereo calibration (BSC) algorithm without any outlier-aware filtering, ranking, or refinement, applied to a checkerboard pattern with 25 mm squares captured at 1280 × 720 resolution. Starting from the BSC algorithm, an intermediate filtered method (implemented at the InterFil calibration algorithm level) was introduced as a first-stage improved calibration approach that introduces a quality-based ranking stage in order to remove weak or less informative calibration pairs before recalibration.

While extending the InterFil calibration algorithm, the proposed Outlier-Aware (OutAw) algorithm uses coverage-optimized subset selection, multi-metric subset evaluation, strict geometric rejection, and a final refinement stage. OutAw algorithm, although it maintains the traditional checkerboard-based calibration model, proves that using additional calibration criteria (epipolar error, hard filtering based on sharpness, projection, coverage, triangulation, square variance, stability, diversity bonus) and introducing data-quality control stages based on geometric coverage, LR consistency, reprojection behavior, and multi-metric subset ranking can be obtained a useful tool for evaluating and optimizing stereo calibration accuracy [6,7].

While OutAw does not address all the identified gaps, it solves some of them, such as the data-quality control gap from conventional calibration methods, by integrating dedicated outlier handling and quality-based subset filtering within the Zhang pipeline [5]. Unlike target-free and ML-based calibration methods, OutAw delivers improved transparency, reliability, and 3D consistency assessment through the usage of epipolar error and triangulation angle evaluation [4]. Because it works as a batch calibration sequence, OutAw enhances and does not replace online recalibration techniques [2,3,8].

An important advantage of the OutAw algorithm is that it integrates geometry scoring and image quality assessment within a unified ranking pipeline. To select the most credible calibration subset, OutAw evaluates left–right (LR) span consistency, checkerboard coverage, sharpness, epipolar consistency, reprojection behavior, triangulated square-size error, square-length variance, and bootstrap stability. The main OutAw novelty is in the way that it evaluates data quality both after and before calibration, applying multi-criteria image validation metrics to iteratively reject unreliable stereo pairs before final parameter estimation [7,9,10,11].

OutAw novelty (approach novelty) consists in its systematic assessment of calibration data quality before and after parameter estimation within a unified stereo calibration pipeline (a multi-criterion ranking and validation framework) that integrates sharpness, epipolar consistency, checkerboard coverage, LR consistency, reprojection behavior, square-length variance, triangulated square-size error, and bootstrap stability. This way, OutAw adds a quality-control layer to the traditional checkerboard-based calibration model, allowing the systematic rejection of low-inconsistent stereo pairs prior to final parameter estimation.

The proposed approach is developed to solve (by incorporating LR consistency, geometric coverage, and multi-criteria subset ranking) the specific limitation of conventional stereo calibration models, the lack of explicit outlier handling and data-quality control in the presence of low-quality calibration images. Because it operates as a batch calibration sequence, it complements rather than replaces online recalibration techniques, improves the reliability of calibration under non-ideal acquisition conditions and preserves interpretability (unlike target-free or ML-based calibration methods).

OutAw reduces the impact of low-quality calibration pairs and supplies more stable geometric and triangulation-based quality evaluation across the analyzed datasets. Summarizing the achieved results, the findings show that the proposed filtering and validation approach improves stereo calibration robustness, compared with the baseline and intermediate approaches. Also, the OutAw quality-aware subset selection component can significantly improve the calibration robustness (especially when the input data are suboptimal and heterogeneous).

These results are very important for DT systems (results significance), because stereo calibration errors directly affect spatial alignment, three-dimensional reconstruction accuracy, and digital representation consistency. Reliable acquisition of geometric data and a stable stereo-based DT estimation are ensured by a more robust calibration pipeline. In this sense, OutAw improves not only calibration robustness but also the geometric fidelity and operational value of DT-based measurement pipelines.

The following research questions are addressed in the present paper:

- RQ1: Is the OpenCV algorithm vulnerable (and to what extent) to imprecise calibration from degraded image pairs?
- RQ2: Without changing the standard stereo calibration method, can a robust filtering and subset ranking strategy improve stereo calibration robustness?
- RQ3: Which geometric and triangulation-based metrics best characterize stereo calibration quality for reliable digital twin data acquisition?
- RQ4: Which algorithm (OpenCV, InterFil, or OutAw) better quantifies and improves stereo camera calibration robustness?

The author’s main contributions are:

- The evaluation of stereo calibration with explicit quality metrics for DT acquisition.
- The development of an integrated framework consisting of geometric validation, pair screening, and subset optimization.
- The proposal of an outlier-aware stereo calibration algorithm, OutAw (that integrates hard geometric selection, candidate subset generation, multi-criterion ranking, bootstrap stability analysis, and triangulation assessment) as an extension of the traditional OpenCV method.
- The proposal of an enhanced evaluation protocol for stereo calibration robustness (epipolar, triangulation, stability, and contamination analyses).

## 2. Related Work

The camera calibration methods (including preprocessing techniques such as subframe synchronization and ROCHADE checkerboard detection) are diverse, starting from the traditional pattern-based methods (Zhang’s method) and scene-based methods (vanishing-point calibration) to modern (camera-LiDAR calibration, online stereo recalibration) and advanced ones (machine-learning (ML)-based stereo calibration and calibration supervision via StOCaMo). Current research focuses on improving the mentioned methods’ efficiency, accuracy, and robustness for different types of cameras and applications [12,13,14,15,16,17].

The basic approach is that of Zhang’s method, which uses checkerboard patterns acquired from various orientations, being the foundation for modern calibration (implemented in modern libraries such as OpenCV) [9,18,19,20,21].

According to [21] under extreme orientations, poor resolution, and lens distortion conditions, ROCHADE identifies up to 80% more checkerboards than standard OpenCV detection.

In [9], the limitations of the classical camera calibration models are presented, highlighting the necessity of advanced models for cameras with a field of view that surpasses 100°. The vanishing-point method offers a pattern-free solution for those scenarios where specialized calibration patterns are not available, by estimating the camera intrinsic from natural geometric features such as parallel lines [18].

Several calibration methods deal with more complex operational and multi-sensor scenarios, beyond single-camera calibration. Such a method is camera-LiDAR that handles inter-sensor extrinsic calibration for autonomous vehicles and robotics through hierarchical optimization, SVD-based calibration, and quality assessment metrics [22,23,24]. The online recalibration methods, especially for stereo systems, restore in real-time the performance deteriorated by mechanical disturbances, stabilizing after an average of seven iterations [25], while ML-based approaches correct pitch angle errors using regression models trained on 300,000 simulated data points [26]. Some calibration methods, such as the improved StOCaMo approach, identify small de-calibration occurrences and correlate calibration deterioration with induced SLAM error [8].

The camera calibration methods are different from the accuracy, approach, and applicability point of view. Table 1 presents a short description of the main calibration methods and techniques, respectively, their associated techniques, performance (accuracy) metrics, applicability domain, and associated citations.

Existing literature has revealed some camera calibration process challenges, such as degraded imaging conditions (low-quality images and strong lens distortion [21]), calibration manipulation problems, the calibration accuracy maintenance over long time periods in time-varying environments [9], and adaptation to varied camera types, such as short focal length and ultra-wide angle lenses [9]. Studies in robust calibration [8,9,21,22,23,24] have shown that low-quality images, erroneous feature detection, and limited pose variation can reduce estimation reliability and 3D reconstruction precision. Current approaches include robust loss functions, subset optimization, view rejection, and learning-based calibration quality assessment.

While considerable improvements have been recorded, the literature still presents some unresolved gaps in stereo camera calibration and also in the DT data capture process, such as the usage of inconsistent quality standards, the insufficient validation of temporal synchronization procedures, difficulties in handling heterogeneous data inputs, and the inadequate lifecycle maintenance, all of which highlight the fact that standardized assessment frameworks evolve more slowly than the practical implementations [28,30].

Another identified problem is that at the calibration level, traditional checkerboard calibration does not incorporate input data validation and outlier rejection, while feature validation procedures and enhanced detection techniques function independently, rather than being combined within a single processing pipeline. Also, although the self-supervised calibration methods (the target-free ones) are practical in real-world conditions, they lack epipolar geometric consistency testing. While adaptive, ML-driven calibration methods leave the entire calibration life-cycle unaddressed in stereo systems, because of limited interpretability and reproducibility, and due to the fact that the entire calibration-generation and calibration-health monitoring processes are treated separately [25,31].

Although the methods described above constitute notable advances in camera calibration, a critical analysis identifies important limitations that OutAw directly targets. The checkerboard detection in demanding imaging conditions is improved using ROCHADE [10], but it does not solve the subsequent input pair selection or outlier rejection challenge. OutAw comes to complement ROCHADE by improving calibration robustness after detection has occurred through subset ranking, pair rejection, and refinement. Although notably adaptive, ML-based stereo calibration methods [26] depend on trained (using large data sets) correction models, limiting their interpretability and reproducibility. On the other hand, OutAw uses deterministic geometric and statistical criteria, making its decisions entirely independent and transparent of training data. Calibration monitoring approaches such as StOCaMo [8] are developed in order to detect miscalibration, after it has occurred during operation, while OutAw works as an offline calibration computation methodology focused on supplying a robust calibration (before deployment), through criteria-based data selection. While OutAw ensures the quality of the initial calibration by rigorously selecting the input dataset, on-the-fly stereo recalibration methods [25] recover deteriorated calibration in real-time (during operation). Overall, these differences establish OutAw not as a replacement for existing methods, but as an outlier-aware complement of traditional Zhang checkerboard-based stereo calibration workflows, filling an important gap in the existing approaches.

Furthermore, a detailed systematic comparison between OutAw and the existing calibration techniques (including ROCHADE, ML-based stereo calibration, StOCaMo, etc.) is presented in Section 5 (Discussion), which compares OutAw with the existing calibration methods across multiple dimensions (relation to OutAw, methodological differences, and performance context).

## 3. Materials and Methods

### 3.1. Camera-Based Data Acquisition for DTs

The base for any DT system is the data acquisition phase, where data collection, integration, synchronization, and final validation take place. The virtual model accuracy depends on the collected data quality (consistency, precision), at this stage the errors being inserted into the model decisions [9,15,22].

The acquisition problem is inseparable from the integration problem as a consequence of the fact that the DT data sources are various (sensors, IoT equipment, cameras, control systems, simulation models, etc.), a fact that ensures significant descriptive richness for virtual representation, but comes with problems regarding formats, sampling rates, synchronized systems and communication protocol integration [9,22].

The robustness of a system ranges beyond data quality, also including different error and adjustment mechanisms, consistent data flows, and robust architectures. In this sense, studies pinpoint diverse weaknesses (architectural fragmentation, incorporation failures, inconsistent updates, etc.) that affect robustness, a solution being the usage of constant validation, inconsistency detection, and standardized data mapping. Also, it is mentioned that the acquisition process must be considered and treated as a controlled and validated process, not just like a passive act of measurement [9,14].

In vision-driven DT workflows, cameras are a handy and key source, supplying minimal cost solid details, surface textures and 3D relations captures, without any need of expensive and dedicated hardware (LiDAR scanners, CAD models).

According to [26], image acquisition can be supplied by various types of cameras (360-degree, standard RGB, stereo, thermal, depth) or from drone-attached imaging systems, each one delivering different descriptive information (different forms of semantic or geometric information) for the DT.

Stereo vision (from the camera-based methods) is significant for geometrical DTs, because it allows depth estimation from image correspondences using intrinsic and extrinsic parameters of the camera pair. The practicality of such data depends mainly on the acquisition quality (viewpoint variety, image sharpness, LR stability, visual feature matches), with direct consequences on the 3D reconstruction accuracy and quality.

Therefore, stereo calibration must be seen as an essential acquisition-quality problem that controls the entire data transformation process (the image data mapping into meaningful 3D representations for DT), not only as a preprocessing step [22,26,28].

So, this interpretation lines up with the present paper approach, in which for assessing the stereo image sets pair-level features, corner detection quality, coverage, sharpness, and calibration performance to increase the vision-based DTs robustness are used.

Because stereo DTs are relying on the paired camera observations’ geometric quality, in the next section, are presented the calibration methods used to convert raw stereo images into validated 3D measurement representations.

### 3.2. Methodology

#### 3.2.1. Feature Extraction and Quality Metrics

A set of features is extracted from each image pair (in order to ensure input data quality for calibration) and used to compute quality metrics, including checkerboard coverage, image sharpness, footprint asymmetry, and pair-level feature extraction (each one being detailed in the following subsections).

##### Image Sharpness Estimation Using the Variance of the Laplacian

For image sharpness estimation (for distinguishing between sharp and blurred images), the Laplacian operator response variance, a standard focus measure extensively employed in image focus assessment that highlights edges and subtle details by measuring second-order derivative changes was used (lower Laplacian variance values are obtained for blurred images, while higher values are obtained by well-focused ones) [32].

This operator was implemented using function cv2.Laplacian (image—grayscale image, depth—output image depth used to store the Laplacian response) from Python 3.8 OpenCV library, after which variance computation over the filter response was achieved. The method was interpreted as follows: sharp and well-focused image for values above 50, acceptable focus for values between 25 and 50, blur (defocus or motion blur) for values below 25. In the proposed OutAw algorithm, min_sharpness (accepted sharpness threshold) was set to 40 to exclude blurry calibration images before subset generation and ranking.

The Laplacian operator is given by Equation (1) [32]:

(1) $$\nabla^{2}I=\frac{\partial^{2}I}{\partial x^{2}}+\frac{\partial^{2}I}{\partial y^{2}}$$

where $\nabla^{2}$ represents the Laplace operator, I is the image intensity function (the pixel intensity at point (*x*, *y*)), *x* and *y* are the spatial coordinates in the image plane, $\frac{\partial^{2}I}{\partial x^{2}}$ is the second-order partial derivative of *I* with respect to *x* (measures the change in intensity curvature along the *x*-direction), while $\frac{\partial^{2}I}{\partial y^{2}}$ denotes the second-order partial derivative of *I* with respect to *y* (measures the change in intensity curvature along the y-direction).

The discrete 3 × 3 Laplacian kernel (*L*) used to approximate the continuous Laplace operator is expressed as [17,32]:

(2) $$L=\left[\begin{array}{ccc} 0 & 1 & 0\\ 1 & -4 & 1\\ 0 & 1 & 0 \end{array}\right]$$

Sharpness metric is defined as follows [33]

(3) $$\mathrm{Sharpness}=\mathrm{Var}(\nabla^{2}I)$$

where $\mathrm{Var}(\nabla^{2}I)$ represents the variance of the Laplacian.

##### Checkerboard Coverage

Checkerboard coverage is quantified by the amount of the image taken up by the detected calibration target (computed as a checkerboard coverage score, which is the ratio of the checkerboard bounding box area to the whole image area). Let Abox be the area of the bounding box surrounding the detected checkerboard corners, and let Aimage = W × H be the area of the entire image; then the computed checkerboard coverage is Abox/Aimage. Larger and more evenly spread checkerboard projections correspond to more reliable observations when calibrating, while small or faraway checkerboards degrade calibration stability in most cases [21,34,35].

To filter out images lacking checkerboard support prior to subset ranking and refinement, OutAw sets a hard threshold of minimum coverage of 0.04, selected as a baseline quality-control criterion. Stereo pairs are rejected when the detected checkerboard covers less than 4% of the image, because these images would only provide weak calibration constraints, but still permit enough remaining eligible pairs for later ranking and refinement.

##### Footprint Asymmetry Measurement

Left–right geometric consistency was evaluated by measuring footprint asymmetry, comparing how large the detected checkerboard appears in left and right stereo images. The understanding being that valid calibration pairs will have similar support for the checkerboard in both views. Large differences in perceived width and height can indicate partial visibility, occlusion, failed detection, or stereo mismatch.

Small values of this metric signify that the checkerboard occupies the same geometric extent in both images and thus indicate consistency of stereo observation. Large values signify asymmetric left–right support for the candidate pair, which may indicate it is invalid or unusable for stereo calibration [9].

Within OutAw, this metric was utilized both as a stereo-consistency descriptor in pair-level quality analysis, as well as a hard-filtering criterion before candidate subset generation.

##### Pair-Level Feature Extraction

In order to assess each stereo pair, 19 quantitative descriptors were used, grouped into image quality, geometric distribution, epipolar behavior, estimated depth, and image center distribution metrics, at the pair-level feature computation level. Explicitly, the feature set contains descriptors that are capturing stereo alignment, visual quality, and pose variation, such as: checkerboard coverage normalized by image area for left, right, and mean (coverage), normalized difference between left and right checkerboard dimensions (LR span consistency), left, right, and mean Laplacian variance (sharpness), median, 95th percentile, maximum error (reprojection statistics), normalized *x* and *y* coordinates for LR checkerboard centers (image-center positions), mean, 95th percentile, maximum vertical alignment error (epipolar statistics) and, estimated board *z*-distances for LR cameras (depth features) [19,25,32].

The stereo calibration process can be negatively affected by a pair with high epipolar error, low coverage, or large LR span discrepancy, more than a pair with uniform geometry and small reprojection statistics.

#### 3.2.2. Calibration Methods and Design

Within this section are described the calibration methods designed and evaluated in the present paper. Are presented three approaches, respectively a baseline stereo calibration algorithm (BSC), an intermediate filtered algorithm incorporating data selection (InterFil), and the proposed outlier-aware algorithm (OutAw), each one being described in the following subsections.

##### Baseline Stereo Calibration (BSC)

The calibration problem can be formulated as minimizing the sum of squared reprojection errors [17,36]:

(4) $$\underset{\Theta }{min}\;\sum_{j=1}^{M}\;\sum_{i=1}^{N_{j}}\;\|x_{ij}-{\hat{x}}_{ij}(\Theta ){\|}^{2}$$

where Θ represents the stereo calibration parameters (distortion coefficients D1, D2, intrinsic matrices K1, K2, translation T and rotation R), M is the number of images, N_j_ is the number of corners in image j, $x_{ij}$ is an observed checkerboard corner in image j, and ${\hat{x}}_{ij}(\Theta )$ is the projected image point as predicted by the camera model.

The BSC method (with the associated pseudocode presented in Algorithm 1) is used as the all-pairs baseline by performing classical stereo calibration on all valid image pairs with no outlier ranking, filtering, or refinement. It is based on the classical OpenCV pipeline, using cv2.stereoCalibrate() function (for stereo calibration), cv2.calibrateCamera() function (for single-camera calibration), Levenberg–Marquardt optimization, and a five-parameter radial-tangential camera distortion model [9].

The obtained parameters are used as baseline evaluation and stereo rectification using epipolar error and reprojection error metrics.
**Algorithm 1** Baseline Stereo Calibration (BSC) high-level PseudocodeInput:  Stereo image pairsOutput:  Baseline calibration parameters and metrics1: Detect each stereo pair checkerboard corner2: The selection of those stereo pairs with pattern found in both images3: The refining (to subpixel accuracy) of checkerboard corners4: The construction of 3D checkerboard object points5: The calibration of each camera (LR)6: Run stereo calibration and image rectification7: The evaluation of calibration reprojection and epipolar error8: Output baseline calibration parameters and evaluation metrics

**Figure 1:**
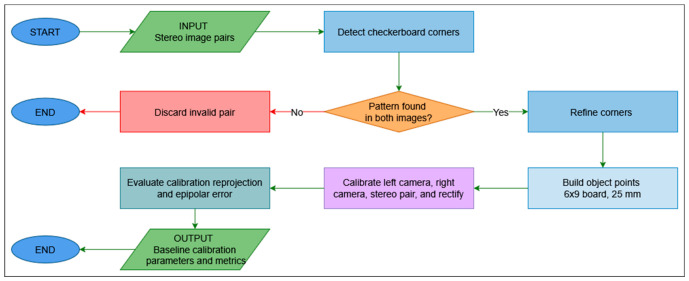
Algorithm 1 (BSC) diagram.

In Figure 1 the BSC algorithm flow diagram is presented based on the pseudocode from Algorithm 1.

##### Intermediate Filtered Algorithm (InterFil)

The InterFil algorithm (with the associated pseudocode presented in Algorithm 2) is an improved version of BSC that uses a preliminary quality-based screening stage, previous to stereo recalibration. Starting from the 49 valid stereo pairs used by the baseline configuration, InterFil computes a set of pair-level indicators (LR span consistency, checkerboard coverage, and reprojection statistics), combining them into a scalar quality score for each pair. First, the entire set of pairs is ranked according to this score, after which the calibration process is repeated on the remaining 42 pairs (the lowest ranked 15% are excluded). Thereby, InterFil is an intermediate method between the standard OpenCV-based BSC method and the full OutAw one, enabling the evaluation of the simple pair selection effect (independent of the more advanced subset-ranking and refinement stages).
**Algorithm 2** InterFil high-level PseudocodeInput:   Valid stereo image pairsOutput:   Filtered calibration parameters and metrics1. Pair-level features (LR span consistency, reprojection error, and checkerboard coverage) extraction for each stereo image pair2. The normalization of the computed features (*Q_k_*-combined quality score computation for each pair)3. All pairs ranking using *Q_k_* and the removal of the lowest 15% from the ranked pairs)4. Calibrate intrinsics on all pairs5. Stereo calibration on filtered pairs6. Compute rectification matrices7. Epipolar error and reprojection metrics evaluation for filtered pairs8. InterFil and BSC results comparison9. Output the final calibration parameters and metrics

**Figure 2:**
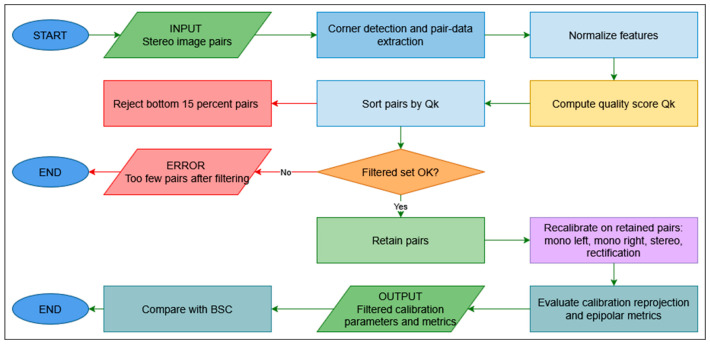
Algorithm 2 (InterFil) diagram.

In Figure 2 the InterFil algorithm flow diagram based on the pseudocode from Algorithm 2 is presented.

##### Proposed Outlier-Aware Algorithm (OutAw)

OutAw was designed as an offline batch pipeline to improve calibration robustness instead of focusing on execution speed. It uses multi-dimensional pose binning along with variability aware subset generation, runs multiple calibrations across different subsets, and integrates bootstrap-based stability analysis. The main computational cost comes from the repeated calibration steps over subsets and bootstrap resamples. So, runtime scales approximately with the total number of evaluations rather than a single calibration pass. This means that the computational cost increases with the number of image pairs and also with the number of calibration iterations.

The proposed OutAw (with the associated pseudocode presented in Algorithm 3) algorithm (extension of the InterFil method) uses multi-criteria ranking, hard geometric filtering, diverse candidate set selection, and refinement (as a final stage). While OutAw (with the assigned pseudocode from Algorithm 3) selects a robust subset setup followed by the refinement of the obtained solution using an additional outlier-rejection step, BSC and InterFil calibrate using a preprocessed set of all candidate pairs.
**Algorithm 3** OutAw high-level PseudocodeInput:  Stereo image pairsOutput:  Final calibration parameters and refined pair set1. The extraction of pair features for each image pair2. Hard geometric filtering (if there are image pairs that fail the hard filtering criteria, then reject and add them to the rejected set)3. The generation of candidate subset by variability-aware sampling4. Multi-subset calibration and ranking (the subset ranking using J-weighted score)5. The selection of the best subset6. Iterative outlier rejection and final refinement7. Supply the final calibration parameters and refined pair set

**Figure 3:**
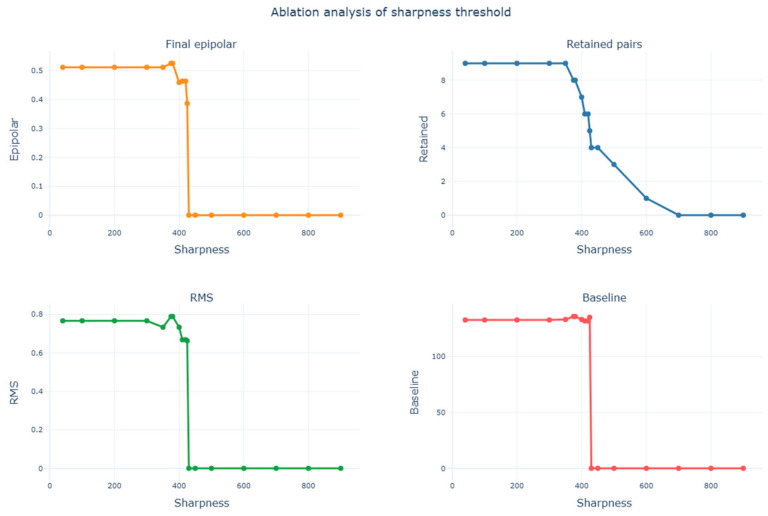
Ablation analysis of the sharpness threshold showing the sharpness effects on final epipolar error, retained pairs, RMS, and baseline performance.

The OutAw workflow (Figure 3) has six stages, which are presented next, respectively: pair feature extraction, hard geometric filtering, diversity-aware subset generation, subset calibration, multi-criteria subset ranking, iterative outlier rejection and final recalibration.

In the first stage (pair feature extraction) of the OutAw method, pair-level descriptors are extracted for each stereo pair. These descriptors (Table 2) characterize image quality, checkerboard coverage, stereo consistency, reprojection and epipolar behavior, and pose-related diversity information used for hard filtering, candidate subset generation, and subset evaluation. The extracted features include sharpness, checkerboard coverage, LR span consistency, reprojection statistics, epipolar statistics, and pose descriptors related to checkerboard location and depth.

In the second stage (hard geometric filtering), for stereo pairs filtering, the OutAw algorithm uses a rule-based threshold criterion (Table 3) instead of a single aggregated scalar score, rejecting pairs below quality thresholds for image sharpness, epipolar consistency, checkerboard coverage, reprojection behavior, and LR span consistency.

While a pair is retained only if it meets all threshold conditions, pairs that fail one or more tests are rejected and tagged according to the failed test (low sharpness, low coverage, high reprojection error, high epipolar error, or large LR span difference).

The sharpness and coverage thresholds were tested for 10 possible values (to evaluate sensitivity), as can be observed in Table 4 and Table 5. The established value of 40 for sharpness threshold functions as a permissive quality selector rather than a restrictive filter (because for this value, all 49 valid pairs were retained, taking into account only the sharpness threshold). As the sharpness threshold increases beyond 400 values, the number of retained pairs decreases (showing that the sharpness metric becomes effective at significantly higher values). So, the choice of 40 value for sharpness threshold must be viewed as a conservative threshold that avoids dataset over-filtering. The minimum sharpness threshold of 40 and the minimum coverage threshold of 0.04 were chosen deliberately in order to avoid being restrictive (they were designed only as lower bounds in order to exclude clearly poor detections), not stringent filters that drive the dataset selection.

Sharpness threshold is dataset specific (their numerical scale varies with image lighting, resolution, content, acquisition conditions), so the suitable value must be established empirically for each setup. It must be mentioned that the sharpness threshold values close to zero (under the established threshold of 40) can be generated by heavily blurred or poorly illuminated images. Therefore, this lower limit is unlikely to over-filter images even in those datasets obtained under considerably various lighting or acquisition conditions. Furthermore, because this was intended to work as a minimal lower limit, a threshold as low as 40 is by design more likely to under-filter than over-filter.

Because in the dataset the observed checkerboard coverage values ranges from 0.08 to 0.22, and, due to the fact that the checkerboard coverage value (0.04) is conservative (it retains all viable calibration pairs that are satisfying the dataset’s empirical coverage distribution), the established threshold operates as a measure against very low-coverage detections. So, the coverage threshold of 0.04 was intentionally set low, in order to avoid biasing pair selection exclusively towards the best positioned checkerboards.

As can be observed in Figure 3, a small ablation style sensitivity analysis chart was performed by varying the sharpness threshold over a limited range. To evaluate whether the selected sharpness threshold is stable or sensitive to changes, and observing its effect on the final epipolar error, stereo RMS, baseline and number of retained pairs.

All four charts highlight a range from 300 to 400 sharpness where all the criteria are optimal. But to not over-fit the OutAw pipeline, a low value that retains good performance was chosen for the sharpness threshold, rather than a fragile optimum that only works at one exact value.

Similarly, a second analysis was made by varying coverage threshold and keeping other ones fixed, as can be observed in Table 5.

Because they capture geometric consistency between the two views (fundamental for reliable rectification and stereo calibration) and because the pairs that fail these criteria are likely to introduce calibration bias (than the ones with reduced sharpness or coverage variations), the reprojection 95th percentile, the epipolar error, and the LR span difference metrics are more decisive in the hard-filtering step. For different datasets and acquisition conditions, the same sensitivity protocol can be straightforwardly applied in order to establish the suitable threshold values.

**Figure 4:**
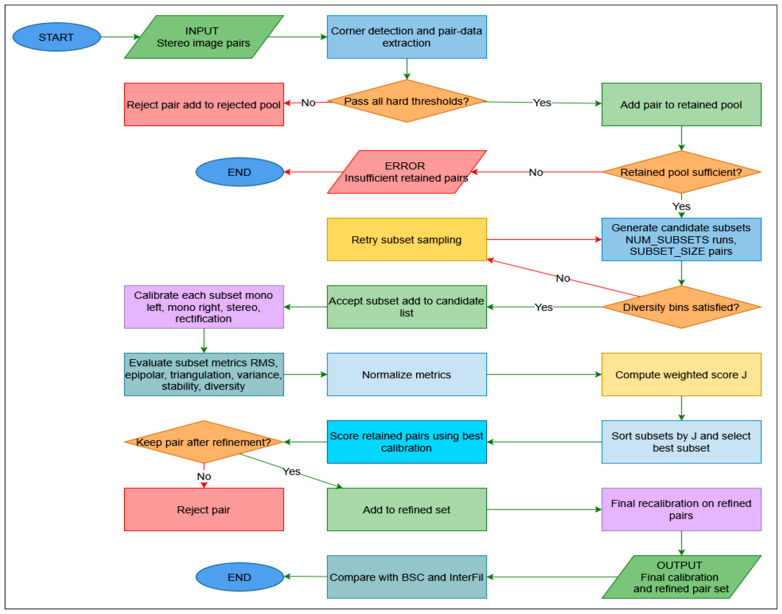
Algorithm 3 (OutAw) diagram.

As can be observed in Figure 4, in the OutAw algorithm’s third stage (diversity-aware subset generation), using a variability-aware sampling strategy, candidate calibration subsets are generated from the retained stereo pairs. In order to improve geometric observability during calibration (rather than selecting only the highest-quality pairs), this stage ensures diversity in image-plane position, checkerboard depth, and orientation.

In order to generate a candidate subset three descriptors were used for each kept stereo pair: one image-position bin chosen from a 3 × 3 grid mapped to the normalized checkerboard center coordinate in the left image, one angle bin based on quantizing the magnitude of the checkerboard pitch to three levels, one depth bin based on discretizing pose_left_z to three levels. Next, in order to ensure representation across the image-position, angle bins, and depth, the candidate subset was generated through a greedy pairs selection. The remaining entries in each subset were filled through score-aware sampling, driven by epipolar error, reprojection error, coverage, and LR span difference.

Because the subset-generation mechanism requires a minimum candidate set (before it can generate geometrically diverse bins in depth, image position, and orientation) the variability-aware sampling stage requires at least five retained stereo pairs to run. The OutAw pipeline associated script terminates with RuntimeError (“Not enough kept pairs for subset generation.”) if less than 5 pairs surpass hard filtering, rather than to continue with an inadequate dataset. This behavior is intentional and it prevents the calibration pipeline from proceeding when the retained set is too narrow to supply reliable variability-aware sampling.

In OutAw fourth stage (subset calibration), each candidate subset is calibrated independently using the standard OpenCV stereo calibration pipeline. In the verified OutAw configuration, this stage is applied to 19 candidate subsets (each subset containing 6 stereo pairs). The checkerboard object points (for each subset) and the corresponding LR image points were collected from the selected image pairs. First, a single-camera calibration for LR cameras was performed using cv2.calibrateCamera(…), while stereo calibration was performed using cv2.stereoCalibrate(…) in order to estimate the relative transformation between the two cameras, together with the epipolar geometry parameters. The resulting calibration parameters were iteratively used for stereo rectification and for later subset evaluation, in the implemented OutAw setup, this process being repeated individually for all 19 candidate subsets.

We combined several complementary quality indicators (presented in Table 6) so that subset selection is not determined by reprojection error alone, all calibrated candidate subsets were ranked using a weighted multi-metric score. So, in order to quantify the subset-level robustness a weighted multi-metric score was used, given by Formula (5):

(5) $$J\left(P\right)=w_{r}\tilde{RMS}\left(P\right)+w_{e}{\tilde{e}}_{epi}\left(P\right)+w_{a}{\tilde{e}}_{tri}\left(P\right)+w_{l}{\tilde{\sigma }}_{sq}\left(P\right)+w_{b}{\tilde{E}}_{stab}\left(P\right)-w_{d}{\tilde{D}}_{b}(P)$$

where P is the candidate calibration subset being evaluated, J(P) is the overall weighted robustness score of subset P, $\tilde{RMS}(P)$ is the normalized RMS reprojection error, ${\tilde{e}}_{epi}(P)$ is the normalized epipolar error, ${\tilde{e}}_{tri}(P)$ is the normalized triangulation error, ${\tilde{\sigma }}_{sq}(P)$ is the normalized square variance, ${\tilde{E}}_{stab}(P)$ is the normalized stability metric, ${\tilde{D}}_{b}(P)$ is the normalized diversity bonus; the sum of all weights ($w_{r},w_{e},w_{a},w_{l},w_{b},w_{d}$) is 1, and lower values for J(P) correspond to more robust subsets.

In order to harmonize image-plane reprojection, stereo consistency, 3D geometric accuracy, intrinsic reconstruction consistency, and calibration stability, while allocating higher importance to epipolar and triangulation performance (because these metrics directly highlight the stereo geometric consistency), the ranking weights were selected empirically.

The selection of the optimal subset that minimizes the weighted multi-metric score $J\left(P\right)$ is given by Formula (6):

(6) $$i^{*}=arg\;\underset{i}{min}\;S({J(P}_{i}))$$

where $i^{*}$ is the index of the selected optimal subset, arg min returns the value of *i* for which $S({J(P}_{i}))$ is minimal, *i* is the current candidate subset index, S() is the scoring function applied to J, and ${J(P}_{i})$ is the weighted multi-metric score of candidate subset P_i_ (evaluated using Formula (6)).

Unlike the baseline approach (BSC), which mainly relies on reprojection performance, in the OutAw fifth stage (multi-criteria subset ranking), each calibrated candidate subset was evaluated using five corresponding metrics, including: RMS reprojection error (obtained directly from the stereo calibration process and used as an image-plane fit measure), epipolar error (computed after stereo rectification as the vertical distance between corresponding checkerboard points in the LR images), triangulated square-size error (measures the deviation between reconstructed checkerboard edge lengths and the known physical square size of 25 mm), square-length variance (defined as the standard deviation of reconstructed checkerboard edge lengths and used to assess internal geometric consistency) and, bootstrap stability (which estimates the calibration robustness by repeated recalibration on bootstrap samples and measures the variability of the recovered stereo baseline and focal lengths) computed with 4 runs and a subset ratio of 0.80.

The OutAw method iterative refinement (stage 6—iterative outlier rejection and final recalibration) uses the best candidate calibration to re-score all retained stereo pairs using epipolar error, then the worst-performing 10–20% are removed as final outliers. Finally, a stereo calibration is performed on the refined set in order to obtain the ultimate robust calibration result.

#### 3.2.3. Post-Calibration Quality Validation

After calibration, four validation procedures (geometric consistency, stereo geometry, bootstrap stability, robustness under contamination) are used to evaluate the quality and robustness of the results, each one being presented in the next subsections.

##### Stereo Geometry Validation

After stereo calibration, the stereo image pairs were geometrically aligned using stereoRectify function (from OpenCV’s calib3d library) with the resulting transformations applied (before evaluation) through the rectification workflow. Stereo rectification determines transformations that are rectifying the two image planes such that corresponding epipolar lines become horizontal, minimizing vertical misalignments.

Stereo geometry was assessed by measuring the vertical epipolar error between rectified LR images corresponding to checkerboard corners.

Epipolar error quantifies stereo geometry by measuring vertical disparity between homologous checkerboard corners in rectified LR images.

The rectified epipolar error, for a corresponding corner pair, is defined as [37,38]:

(7) $$e_{epi}^{\left(j\right)}=\frac{1}{N_{j}}\sum_{i=1}^{N_{j}}\;|v_{i,L}^{\left(j,rect\right)}-v_{i,R}^{\left(j,rect\right)}|$$

where $e_{epi}^{\left(j\right)}$ represents the mean epipolar error for stereo pair j, j is the index of the stereo image pair, $N_{j}$ is the number of detected corners in image pair j, i is the index of the current corner, $v_{i,L}^{\left(j,rect\right)}$ is the vertical coordinate of corner i in the rectified left image and, $v_{i,R}^{\left(j,rect\right)}$ is the vertical coordinate of corner i in the rectified right image.

The mean epipolar error (lower values supplied after rectification, indicating better geometric consistency) was obtained by averaging the errors over all identified image corners and evaluated stereo pairs.

##### Geometric Consistency Validation

Unlike BSC and InterFil workflows, OutAw has explicit 3D geometric validations after stereo calibration and rectification. The first used metric (defined by Formula (8)) is the square absolute error (the absolute difference between the adjacent checkerboard corners reconstructed 3D distance and the known physical square size of 25 mm) [21,39].

(8) $$e_{s}^{\left(j\right)}=\frac{1}{N_{j}}\sum_{k=1}^{N_{j}}\;|{\hat{s}}_{j,k}-s_{0}|$$

where $e_{s}^{\left(j\right)}$ is the mean square absolute error for image pair *j*, *j* is the index of the stereo image pair, $N_{j}$ is the number of detected corners in image pair *j*, *k* is the index of the current adjacent corner pair, ${\hat{s}}_{j,k}$ represents the reconstructed 3D distance between adjacent corners k in pair *j* and, $s_{0}$ is the known physical square size.

The second metric (square-length standard deviation given by Formula (9)), measures the variability of reconstructed edge distances within a checkerboard detection [21,39]:

(9) $$\sigma_{s}^{\left(j\right)}=\sqrt{\frac{1}{N_{j}-1}\sum_{k=1}^{N_{j}}\;\;({\hat{s}}_{j,k}-\bar{s_{j}}{)}^{2}}$$

where $\sigma_{s}^{\left(j\right)}$ is the square-length standard deviation for image pair j, j is the index of the stereo image pair, $N_{j}$ is the number of detected corners in image pair j, k is the index of the current adjacent corner pair, ${\hat{s}}_{j,k}$ is the reconstructed 3D distance between adjacent corners k in pair j, while $\bar{s_{j}}$ represents the mean reconstructed edge distance for image pair j.

While the overall metric is obtained by averaging this standard deviation over all evaluated stereo pairs, for one checkerboard detection, it is computed over all evaluated adjacent segment distances.

While low square-length variance indicates high intrinsic geometric consistency of the estimated checkerboard geometry, low square-size error indicates accurate scale recovery.

##### Bootstrap Stability Analysis

To evaluate the stereo calibration model robustness by iteratively recalibrating the stereo camera system on randomly resampled subsets of image pairs (measuring how much the estimated parameters vary across runs), the bootstrap stability analysis is a very useful resampling-based method. This method involves the selection of a sampling ratio in order to supply various bootstrap samples by resampling with replacement, the stereo camera system calibration on each sample, and the main parameters (both cameras’ focal lengths, stereo baseline, and stereo RMS reprojection error) variability monitoring. In the case of a robust calibration, under repeated resampling, only small parameter variations must be obtained, while high variations indicate that the calibration is sensitive and unstable to the particular image pairs used [40].

The bootstrap stability analysis (from a methodological point of view) is essential because it supplies additional information relative to standard error measures (such as reprojection error), and due to the fact that the calibration process may supply a low reprojection error on a given dataset, while remaining unreliable (its parameters vary significantly under resampling). So, bootstrap stability analysis is very useful in robust stereo calibration workflows, especially in cases where the goal is not only to fit the observed image points accurately, but also to guarantee that the recovered camera model remains consistent under data variation [25].

##### Robustness Under Contamination

The ability of a calibration method to generate robust parameter values when the input dataset contains low-quality observations (including outliers), is what defines robustness under contamination.

In stereo calibration, the sources of contamination are multiple, including poor checkerboard detections, motion-induced blur, poor corner localization, and image pairs that do not adequately represent the stereo geometry. Through contamination analysis, it is evaluated how sensitive a calibration method is to all these contaminated inputs by testing how the estimated stereo model changes as the contamination level increases, using the synthetic outlier injection methodology established in robust statistics literature [41,42]. A formal framework for quantifying an estimator breakdown point (defined as the maximum fraction of contaminated observations it can tolerate before supplying unreliable estimates [41]) was adopted, in order to evaluate whether the calibration pipeline, under increasing contamination, breaks down progressively [8,26,41,42].

**Figure 5:**
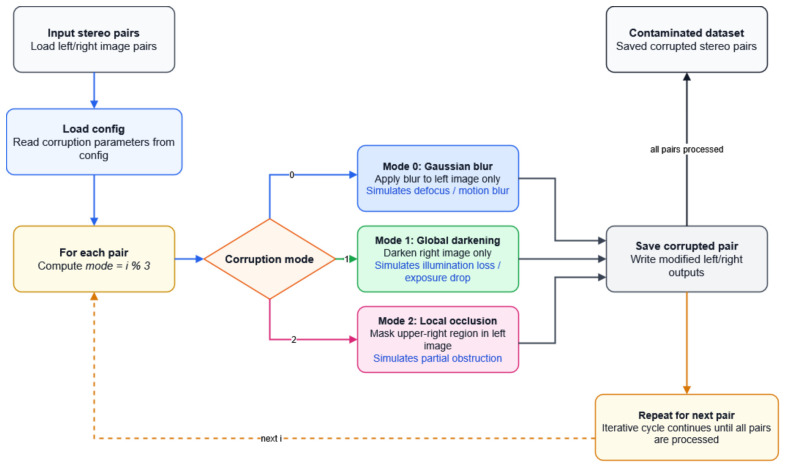
Corruption injection pipeline for synthetic contamination study.

Synthetic contamination (Figure 5) was introduced into the captured stereo pairs using three repeatable corruption modes to assess robustness under contamination. In a cyclic manner, each pair was modified by one of the following methods: Gaussian blurring the left image, darkening the right image through intensity scaling, or overlaying a black rectangular occlusion on the left image. The corresponding parameters, such as blur kernel size and darkening coefficient, were read from the configuration file, ensuring that the contamination process is fully reproducible. This setup provides a controlled test of the method’s sensitivity to blur, illumination degradation, and partial occlusion.

The OutAw workflow was developed especially to minimize the influence of contaminated or geometrically unreliable pairs through a combination of strict threshold constraints, such as reprojection, epipolar error, LR geometric consistency checks, and final refinement subset ranking. Grounded in the synthetic outlier injection methodology of robust statistics [3,4,43], in order to evaluate estimator breakdown behavior, synthetic outliers were injected at controlled contamination levels.

What makes OutAw fundamentally more robust to dataset contamination than baseline all-pairs calibration approaches are the fact that all these integrated mechanisms ensure that outlier or poor-quality observations are identified and rejected before they can compromise the entire calibration process.

### 3.3. Implementation Details

This section presents the implementation aspects of the proposed methodology (experimental setup and stages, checkerboard corner detection, data acquisition and configuration), each one presented in the next subsections.

#### 3.3.1. Experimental Setup and Stages

**Figure 6:**
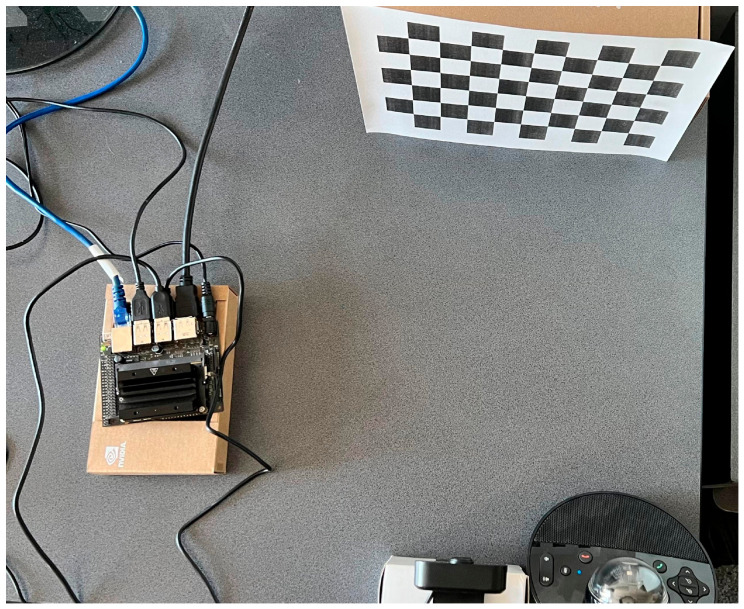
The experimental stereo calibration setup with checkerboard target and dual-camera acquisition platform.

**Figure 7:**
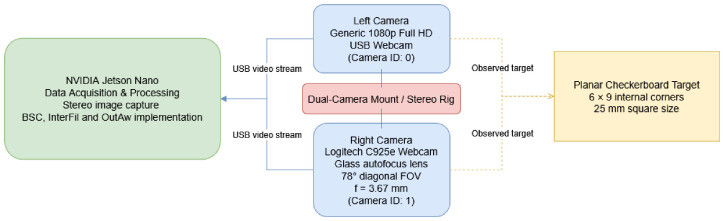
Components diagram for the experimental stereo calibration setup with checkerboard target and dual-camera acquisition platform.

The experimental setup (Figure 6 and Figure 7) contains two USB cameras used to acquire sets of synchronized images with a 1280 × 720-pixel resolution. For the stereo camera system, the first camera is a Logitech C925e webcam, glass lens, autofocus lens with a 78° diagonal field of view and 3.67 mm focal length. The second camera is a generic 1080 p Full HD USB webcam.

Image acquisition and all the necessary stages (calibration, rectification, and assessment) were implemented in Python 3.8 using OpenCV library (version 4.8.0). As can be observed in Figure 5, as calibration target was used a planar 6 rows by 9 columns checkerboard of internal corners with a physical square size of 25 mm.

For the experimental assessment, one stereo dataset that contains 49 image pairs was used. The calibration algorithms that are assessed are: a baseline OpenCV all-pairs calibration (BSC) algorithm that uses all 49 valid image pairs, an intermediate filtered (InterFil) algorithm that uses 42 pairs (after scoring all 49 pairs, it removes the lowest 15%, 7 weak pairs being rejected), and a refined outlier-aware (OutAw) algorithm that examines each pair more carefully using specific descriptors (like those presented in Table 2) that uses only 9 pairs.

**Figure 8:**
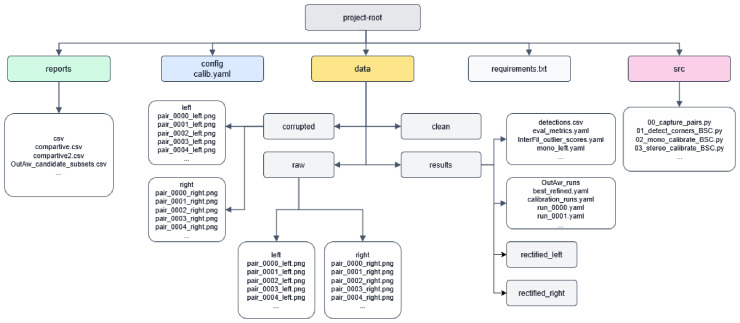
Compact tree diagram for the Python project structure.

For reproducibility purposes, Figure 8 presents the Python project structure, showing the configuration file, input data folders, generated results, report outputs, and source scripts used for BSC, InterFil, and OutAw.

#### 3.3.2. Data Acquisition and Configuration

**Figure 9:**
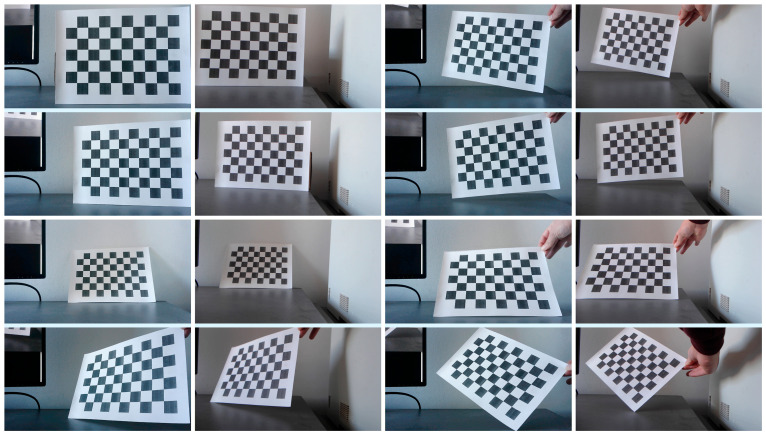
Captured pairs of samples (selection).

As has been mentioned, the stereo dataset contains 49 image pairs (Figure 9) obtained with two different USB cameras detecting the checkerboard configuration. For checkerboard corner detection, OpenCV’s find-Chessboard-Corners function was used, and for their refinement (subpixel precision) cornerSubPix function from the same library was used.

The calibration configuration key parameters were set to the these values: 1280 × 720 pixels for image resolution, 6 rows × 9 columns for checkerboard layout, 25.0 mm for square size, left = 0 and right = 1 for camera IDs, target pair number was set to minimum 30, maximum 100 iteration as stopping criteria, epsilon was set to 0.0001, fixed intrinsics during stereo camera calibration, rejection ratio 0.15 for InterFil filtering, 50 subset calibrations, 70% subset sampling rate, and seed 42 as OutAw parameters.

The thresholds for OutAw hard filtering were set to the next values: 1.0 pixels for epipolar error maximal value, 40.0 for minimal sharpness, 0.04 for minimal coverage, 0.08 for maximum normalized LR span difference, and 1.5 pixels for maximum reprojection in the 95th percentile.

In Table 7 a short description of the assessed camera calibration algorithms (baseline OpenCV all-pairs calibration -BSC, an intermediate filtered variant –InterFil, and a proposed outlier-aware stereo calibration algorithm—OutAw) is presented, while in Table 8 the specific evaluation metrics used for stereo calibration quality evaluation are presented.

The experimental workflow procedure includes the following steps: acquiring stereo image pairs (49 pairs, 1280 × 720 resolution, 6 × 9 checkerboard), checkerboard corner detection and refinement for all image pairs; BSC execution using the entire set of pairs; extraction of 19 pair-level quality metrics; filtering (exclude the lowest 15%, rank the pairs, recalibrate with 42 pairs) using InterFil; hard filtering (exclude pairs that do not meet geometric thresholds) using OutAw; generation of 19 candidate six-pair subsets from OutAw filtered set; calibration and evaluation of each subset using the first five metrics from Table 3; subset ordering by weighted overall score and the selection of the best ones (the 15th run); and final solution refining from the best-ranked subset.

Finally, the BSC, InterFil, and OutAw algorithms are compared using the metrics presented in Table 3, the Bootstrap stability analysis is performed on the baseline (20 runs), and also a contamination study (0%, 10%, 20%, 30%) on 34-pair subsets is achieved.

#### 3.3.3. Checkerboard Corner Detection

For the automatic detection of the checkerboard inner corners in grayscale images, was used cv2.findChessboardCorners (image—grayscale input, patternSize—number of inner corners, flags—other optimal detection parameters) function from Python 3.8 OpenCV library, through the cv2 module. In the current workflow, to obtain the image points needed for stereo camera calibration (the inner corners’ complete array of the specified checkerboard layout size), the mentioned function during the initial feature-extraction step, the function that returns a detection flag with the equivalent pixel coordinates, was used. In order to extract 6 × 9 checkerboard corners from all image stereo pairs, the mentioned detection function was applied uniformly in the BSC, InterFil, and also in the OutAw algorithm workflow.

For improving the localization of formerly detected checkerboard corners (for subpixel corner refinement) cv2.cornerSubPix (image, corners, winSize, zero-Zone, criteria) function, also from the Python 3.8 OpenCV library, was used. Using the mentioned function, the corner positions are refined (at subpixel accuracy level) by improving the corners’ location within a local region through an iterative gradient-based process. Respectively, it searches in a specified window around each corner and finishes when the convergence threshold is encountered or when the maximum iteration count is reached.

The main function parameters are: image (grayscale input image), corners (initial corner coordinates), winSize (half window size identification), and zeroZone (the central exclusion region) and criteria (stopping conditions).

For the current implementation, in order to ensure the subpixel iterative refinement process stable convergence, the stopping criteria were set to max_iter = 100 and epsilon to 0.0001, achieving a balance between OpenCV calibration workflow computational cost and localization precision.

Using the standard planar calibration design described in the literature [9,12,15], especially in Zhang’s calibration method and in the OpenCV camera calibration framework [12,21,44], the 3D checkerboard grid reconstruction technique was used in order to develop the checkerboard configuration 3D coordinate system by associating each inner corner to its physical coordinates. The checkerboard system coordinates are Z = 0 (for reference plane), X and Y represent the distance between neighboring corners, while the inner corners and 3D reference points are defined according to relation (1) [8,41]:

(10) $$O_{ij}=\left(i\cdot d,j\cdot d,0\right),i=0,\ldots ,\mathrm{cols}-1,j=0,\ldots ,\mathrm{rows}-1$$

where $O_{ij}$ represents the 3D coordinates of the inner corner at position (*i*, *j*) in the checkerboard reference coordinate system, and *d* is the size of one checkerboard square, respectively, the equation represents the distance between two adjacent inner corners.

## 4. Results

Experimental results of the three stereo calibration methods tested in this work are described in this chapter. The results corresponding to baseline configuration (BSC), the intermediate filtered algorithm (InterFil) and the proposed outlier-aware algorithm (OutAw) are provided.

### 4.1. Baseline Stereo Calibration (BSC) Results

Baseline stereo calibration (BSC) is considered the standard variant; it was run on all 49 valid stereo pairs, without any further filtering, selection or other improvement of input data. In this variant the algorithm executes the basic OpenCV procedure: corner finding of chessboard in left and right image, followed by subpixel refinement of corner positions. Individual calibration of two cameras and finally stereo calibration to obtain the geometric relationship between the two cameras are performed. BSC resulted in stereo RMS of 1.9385 px and estimated stereo baseline of 134.9002 mm. After rectification, evaluated on 48 image pairs, the mean vertical epipolar error was 1.3687 px, with the median error being 0.8483 px. As can be seen, this basic method provides usable calibration, however with significant deviation between image pairs (Table 9).

The estimated intrinsic matrix $K_{1}$ for the left camera is:

(11) $$K_{1}=\left[\begin{array}{ccc} 1262.2224 & 0 & 582.3646\\ 0 & 1255.4895 & 363.5142\\ 0 & 0 & 1 \end{array}\right]$$

and the distortion coefficient D_1_ is:

(12) $$D_{1}=\left[\begin{array}{ccc} 0.4065 & -2.1033 & 0.0024 \end{array}\;\begin{array}{cc} -0.0163 & 4.2139 \end{array}\right].$$

For the right camera the intrinsic matrix $K_{2}$ is:

(13) $$K_{2}=\left[\begin{array}{ccc} 1085.3653 & 0 & 560.0632\\ 0 & 1078.9389 & 343.5532\\ 0 & 0 & 1 \end{array}\right]$$

and the distortion coefficient D_2_ is:

(14) $$D_{2}=\left[\begin{array}{ccc} 0.4056 & -2.4769 & 0.0059 \end{array}\begin{array}{cc} -0.0233 & 5.8827 \end{array}\right].$$

In the extrinsic case, the estimated translation vector T was:

(15) $$T={\left[\begin{array}{ccc} -133.2537 & -10.9959 & 17.9054 \end{array}\right]}^{T}$$

which translates to about 134.9 mm of physical separation between the cameras.

The focal lengths are different for the two cameras, meaning the two views do not behave symmetrically in terms of pixel geometry, and the same intrinsics should not be assumed for both. Examining the results for each rectified pair, there is a large spread of vertical epipolar error. The lowest average errors are seen for pairs pair_0048 (0.3108 px), pair_0050 (0.3846 px), pair_0038 (0.3862 px), and pair_0033 (0.3936 px), demonstrating very good stereo alignment for those frames.

Conversely, pairs pair_0023 (5.3560 px), pair_0020 (4.9750 px), pair_0022 (4.0306 px) and pair_0042 (3.9434 px) had the highest average errors which suggests they are bad for geometric consistency of the calibration obtained.

The baseline resulted in a calibration that was usable; however, there were several image pairs that had a relatively high epipolar error.

**Figure 10:**
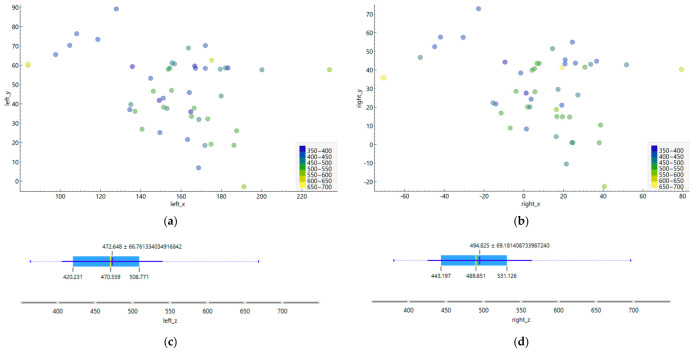
Distribution of calibration board poses for the 49 stereo image pairs. (**a**,**b**) show the board center positions in the left and right camera coordinate frames, color encoding: depth z; (**c**,**d**) show the corresponding box plots of z for the two cameras.

Figure 10 illustrates the spatial coverage of the calibration board poses used for stereo calibration. Figure 10a,b describe the estimated board center translations in the left and right camera coordinate frames, with color encoding the board depth z. Figure 10c,d summarize the corresponding z coordinate distributions using box plots, showing the median, interquartile range and full spread of the board distance observed by each camera. The combined distribution indicates that the calibration set covers a broad range of viewing distances and lateral offsets, which is important for stable estimation of the intrinsic and extrinsic stereo parameters.

### 4.2. Intermediate Filtered Algorithm (InterFil) Results

The proposed InterFil filtering approach: An intermediate filtering algorithm which improved upon the all-pairs baseline calibration by attempting to expand upon it further. It does so by finding the weakest stereo pairs and explicitly eliminating them. It simply inserts a rudimentary ranking stage based on pair-level calibration heuristics which ranked stereo calibration pairs, then recalibrated the stereo system using only the remaining candidates.

Table 10 shows the InterFil results in terms of retained/rejected pairs, ranking features, and achieved calibration accuracy. Also listed are best and worst ranked pairs for qualitative understanding of how filtering distinguished better calibration pairs from weak ones.

InterFil determined three screening features of each of 49 stereo pairs, checkerboard coverage, reprojection behavior and left–right span consistency, which were reported as cov_mea, reproj_mean and lr_span_diff_norm. Representative high-ranked examples included pair_0010, with cov_mean: 0.1106, reproj_mean: 0.3790, and lr_span_diff_norm: 0.0381, and pair_0005, with cov_mean: 0.1744, reproj_mean: 0.4534, and lr_span_diff_norm: 0.0892. These descriptors were normalized and added together into a combined score Q_k_ such that smaller scores identified pairs that were more geometrically reliable and useful in calibration.

The ranking stage placed pair_0010 first with a score of 0.1788, followed by pair_0005 at 0.2186 and pair_0003 at 0.2341, while pair_0014 was the weakest case with a score of 1.9260. Then InterFil discarded the bottom 15% of the ranked data which, in effect, implied discarding 7 of the 49 pairs: pair_0014, pair_0031, pair_0032, pair_0033, pair_0034, pair_0036, and pair_0037. This resulted in 42 pairs left, a subset of which was clearly stated to be adequate to continue with the subsequent calibration steps.

Monocular calibration on the filtered subset gave a left-camera RMS of 1.0089 and a right-camera RMS of 0.9833. The corresponding intrinsic parameters were fx1 = 1262.2224, fy1 = 1255.4895, cx1 = 582.3646, cy1 = 363.5142 for the left camera, and fx2 = 1085.3653, fy2 = 1078.9389, cx2 = 560.0632, cy2 = 343.5532 for the right camera. Stereo calibration of the 42-pair subset followed by rectification resulted in an estimated baseline of 132.9996 mm and an estimated stereo RMS of 1.5540 px.

The InterFil solution had a mean vertical epipolar error of 1.3354 px, a left mean reprojection error of 0.7995 px and a right mean reprojection error of 0.7704 px in the final evaluation. InterFil achieved a stereo RMS reduction of 0.3846 px and an epipolar mean reduction of 0.0227 px compared to the baseline BSC setting, where all 49 pairs were used to achieve a stereo RMS of 1.9385 px, an epipolar mean of 1.3582 px, and a baseline of 134.9002 These findings demonstrate that even a basic score-based pruning strategy increased calibration robustness compared to the all-pairs baseline, but the improvement in epipolar accuracy was small compared to the more significant improvement in stereo RMS.

The final InterFil output was therefore a filtered stereo calibration based on 42 retained pairs.

### 4.3. Proposed Outlier-Aware Method (OutAw) Results

OutAw was assessed as a multi-stage algorithm, consisting of pair quality scoring, filtering, subset generation, ranking and refinement. This process of successive approximation was used to narrow down the calibration set while preserving geometric fidelity. Intermediate outputs allow the understanding of how the final output was arrived at, including how each choice was made based on quantitative reprojection, epipolar, and triangulation-based metrics. Table 11 shows top OutAw results consisting of candidate subset, subset stability stats and final calibration performance.

First stage extracts quality and geometry features from each stereo pair, such as: sharpness, checkerboard coverage, left–right span difference, reprojection statistics, epipolar statistics, normalized image position, and estimated depth.

The representative retained pairs already had good feature values, such as pair_0004’s coverage mean of 0.1684, reprojection p95 of 1.0942, epipolar mean of 0.6051, and left–right span difference of 0.0538, as seen in Table 12. Another example is pair_0007 which had a coverage mean of 0.1654, a reprojection p95 of 1.0618, an epipolar mean of 0.5519, and a left–right span difference of 0.0541.

**Figure 11:**
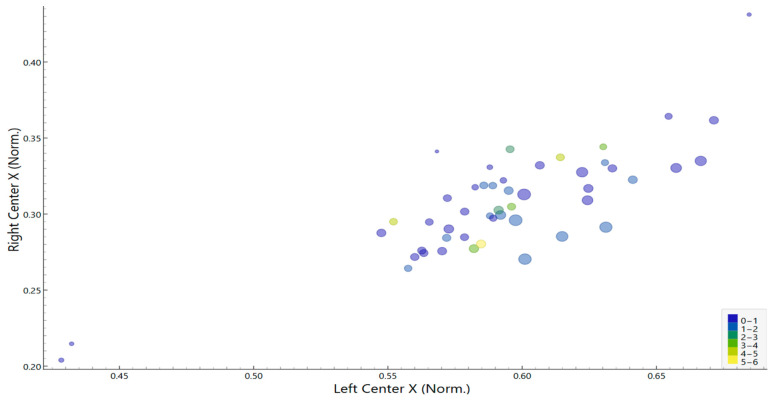
Horizontal consistency of checkerboard center position between left and right stereo images. Marker color: mean epipolar error, and marker size: checkerboard mean coverage.

Figure 11 plots the normalized checkerboard center x-coordinate value from the left image against that of the right image. The approximately 0.823 correlation strongly suggests that when the board is more towards the right of the left image, it also tends to be more towards the right of the right image. The concentration of points into one rather tight cluster suggest most pairs have roughly similar stereo viewing geometry, with only a few board placements far towards one side of the image plane (low-x values near (0.43, 0.20) (0.43, 0.20)) or far towards both sides of the image plane (large xx value near (0.68, 0.43) (0.68, 0.43)).

Marker size indicates that checkerboard coverage is largest among this central cluster of points. From the spread of colors, high epipolar error does not appear to be associated with any horizontal position. Image-plane location thus contributes to the diversity of calibration data without itself explaining calibration quality.

**Figure 12:**
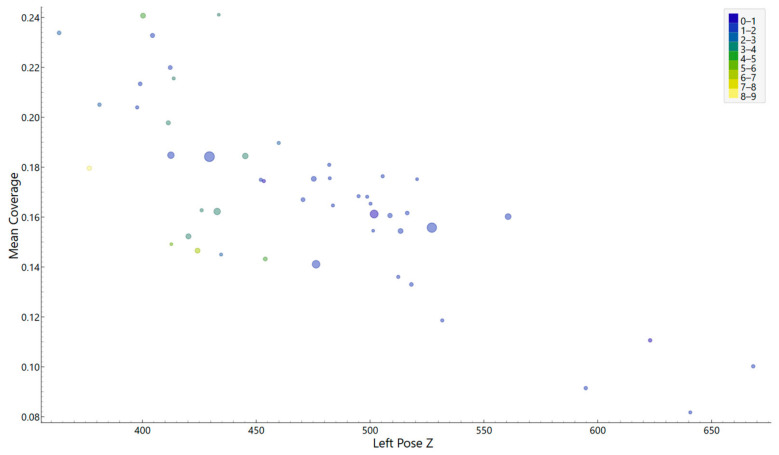
Relationship between checkerboard depth and image coverage in the left camera. Marker color: reprojection error, and marker size: mean epipolar error.

The plot from Figure 12 shows strong negative correlation (−0.777) between pose_left_z and coverage_mean because as the checkerboard gets farther from the left camera it takes up less of the image. This trend makes intuitive sense, as coverage is a proxy for the size of the checkerboard projected in the image, and the low coverage value is documented to be symptomatic of a small or distant checkerboard (0.04 is used as the minimum passing threshold).

Mid-range depths can still suffer from high epipolar error as shown by larger markers, and colors indicate that both moderate and large depths can achieve low reprojection error. Depth strongly influences visibility but is not the sole factor of quality.

**Figure 13:**
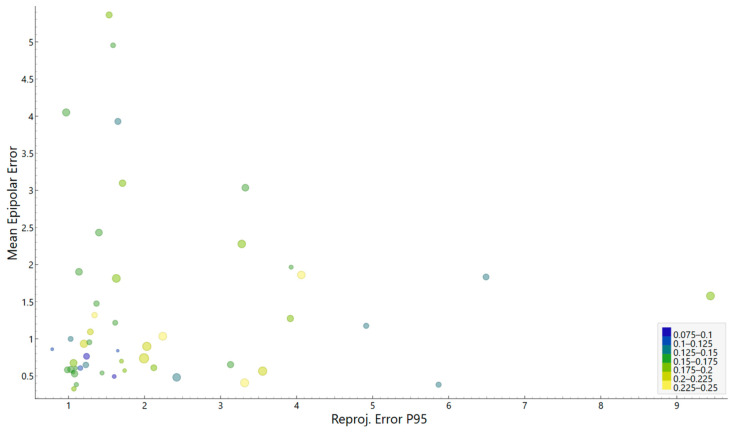
Joint distribution of reprojection error and mean epipolar error across stereo pairs. Marker color: mean coverage, and marker size: normalized left–right span difference.

This scatter plot (Figure 13) indicates that reproj_p95 and epipolar_mean are nearly independent on this dataset; the correlation was only 0.034. Poor reprojection performance therefore does not imply poor epipolar consistency, or vice versa. The lower-left quadrant contains the highest-scoring pairs, and according to the hard-filter thresholds: 16 pairs pass both tests, 12 fail reprojection only, 6 fail epipolar error only, and 15 fail both. This breakdown supports the decision to consider both criteria separately during filtering. The color and size encodings also indicate that high-coverage pairs with low left–right span difference can still populate both the good and bad regions of this space, so while useful, coverage amounts and geometric symmetry fail to explain all poor calibration behavior by themselves.

Next, hard filtering eliminated pairs that did not meet preset thresholds on reprojection, epipolar consistency, or left–right footprint asymmetry. Only nine pairs passed this test, many weak pairs were eliminated for various reasons. For example, pair_0014 was rejected based on reprojection p95 of 9.443, epipolar mean of 1.577, and left–right span difference of 0.119. Pair_0023 was also rejected with reprojection p95 of 1.534, epipolar mean of 5.364, and left–right span difference of 0.090.

The retained pairs became the pool from which candidates would be generated. Where created 19 candidate subsets, each consisting of six stereo pairs. Each stereo subset reported the same diversity summary: 1 unique image bin, 3 unique depth bins, and 2 unique angle bins. Bins refer to discrete categories that were used to promote pose diversity in chosen calibration subsets. Unique bins can be in terms of image (where the checkerboard is in the image), angle (grouping similar orientations of the board), or z-bin (grouping together boards that have similar depth or distance from the cameras).

Each candidate subset was then tuned and scored with a more extensive metric set than that used by the baseline methods. These metrics included stereo RMS, estimated baseline, mean epipolar error, mean reconstructed square length, square-length standard deviation, mean square absolute error, and stability metrics. Top scoring candidates produced stereo RMS estimates below 0.7 px. Run 15 resulted in stereo RMS of 0.6895, mean epipolar error of 0.5120, mean square absolute error of 0.1097 mm, square-length standard deviation of 0.1140 mm, and baseline standard deviation of 0.6105 mm (Table 12).

**Figure 14:**
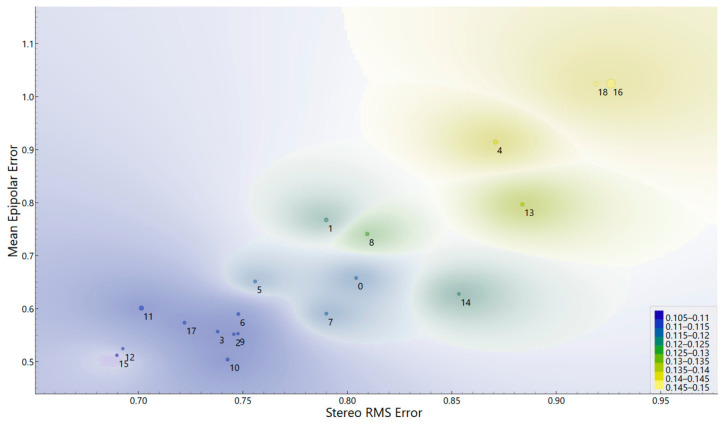
Overall quality of candidate stereo calibration subsets based on stereo rms and mean epipolar error. Marker color: mean square absolute error, and marker size: baseline standard deviation, label: run id.

Figure 14 depicts the stereo RMS error versus mean epipolar error. Can be seen a strong positive correlation between these two measures with correlation coefficient near 0.890. This makes sense because poor reprojection performance would also imply poor stereo rectification consistency on average. The best runs fall in the lower-left quadrant of the plot: runs 15, 12, 2, 3, and 10 rank well by both criteria of low stereo RMS error and low mean epipolar error, while runs 16 and 18 are easily identified as poor quality outliers by their location in the upper-right quadrant. Both runs have a maximum mean square absolute error as well.

The color scale also validates that geometric reconstruction error gets worse towards the upper-right region, and larger marker size for certain runs denotes greater baseline variability, so overall this figure validates the author’s intuition that subsets towards the lower-left corner are better-balanced subsets to choose from for final calibration.

**Figure 15:**
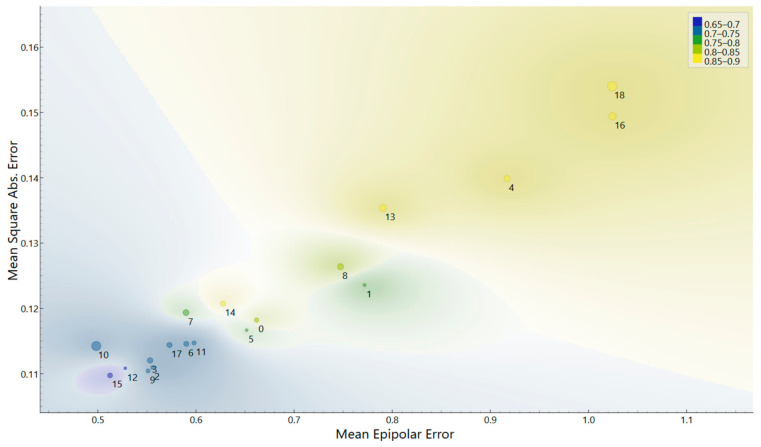
Relationship between epipolar consistency and 3d reconstruction error in candidate calibration subsets. Marker color: stereo RMS error, and marker size: mean square length, label: run id.

This plot (Figure 15) demonstrates very tight correlation between mean epipolar error and mean square absolute error (correlation ~0.976), implying that subsets which result in poorer stereo rectification also tend to lead to a less accurate reconstruction of the 3D square. Runs that produce lower errors fall into the lower-left corner of this graph, particularly runs 15, 12, 9, 2, 3, and 10, while runs 18 and 16 appear to be the two worst subset candidates (top-right).

The color encoding shows that higher Stereo RMS Error generally accompanies worse geometry and 3D accuracy, and the point sizes indicate only small variation in mean square length around the nominal 25 mm value, so the main differentiator among subsets is not the average reconstructed square size itself but the associated error and consistency measures.

**Figure 16:**
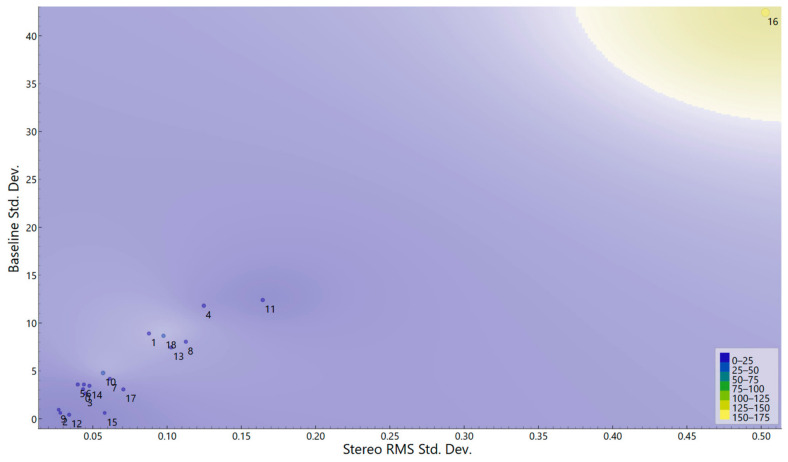
Bootstrap stability of candidate calibration subsets based on stereo rms and baseline variability. Marker color: fx2 standard deviation, and marker size: fy2 standard deviation, label: run id.

The scatter plot from Figure 16 depicts a strong positive relationship between stereo_rms_std and baseline_std (correlation ~0.988). This indicates subsets that create unstable stereo reprojection errors also produce unstable baseline estimates when bootstrap resampling is applied. Runs tend to aggregate in the bottom left corner of the graph. These represent relatively stable solutions. Run 16, however, is an outlier (having stereo_rms_std: 0.503, baseline_std: 42.44, fx2_std: 161.96, and fy2_std: 163.20), suggesting a noisy and unstable calibration subset.

Runs are mostly concentrated in the lower-left quadrant. This indicates that these runs tend to have stable calibration results. Run 16 is a clear outlier since stereo RMS values as well as values for baseline and focal length are much more spread out indicating that this calibration subset is unstable.

Table 13 shows the five highest scoring OutAw candidate subsets. Once scored, all candidate subsets were ranked based on the weighted OutAw score. Lower scores indicate higher quality calibration and robustness. The best performing subset uses not just low stereo RMS but also low epipolar error, low square error and low baseline variation. It does not try to optimize a single metric which justifies ranking with multiple criteria rather than reprojection accuracy alone.

Therefore run 15 was chosen as the best subset. It included pair_0004, pair_0007, pair_0010, pair_0030, pair_0045 and pair_0048. It had low reprojection and epipolar error, yet had high triangulation-based consistency, as well as low parameter variability.

The retained pool was rescoring using the calibration found from the best subset. Pair_0050, pair_0007, pair_0004 and pair_0030 tied for lowest epipolar mean in the retained pool with epipolar means of 0.2761, 0.2848, 0.2909 and 0.3086 respectively.

**Figure 17:**
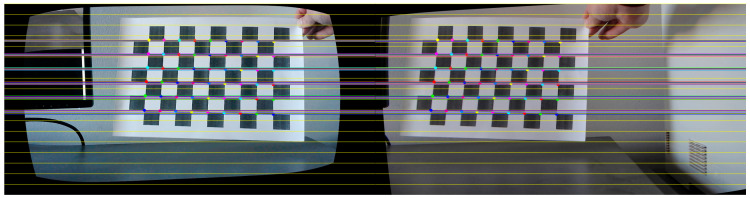
Rectified stereo pair for OutAw method with checkerboard corner overlays and horizontal epipolar guides.

After rectification, corresponding points should be in the same row, as can be seen in Figure 17. The colored markers and horizontal lines indicate that the geometry has been aligned correctly.

**Figure 18:**
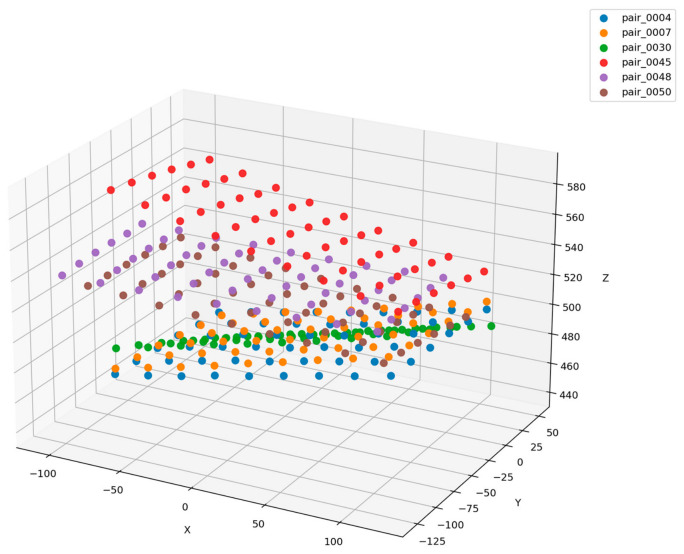
3D scatter plot of triangulated checkerboard corner positions grouped by stereo pair.

The plot from Figure 18 shows the reconstructed 3D points of checkerboard corner points after stereo rectification, using the OutAw algorithm. X and Y represents the planar coordinates of the checkerboard and Z is the depth. Each color corresponds to one stereo pair. The color points denote that the board was captured from multiple poses and distances. The points form ordered grids, the shape is smooth, and there is no sign of major triangulation failure.

OutAw performed the best of all tested algorithms. Aside from having significantly reduced stereo RMS and epipolar error, OutAw also had very low triangulation-based square absolute error and square length standard deviation showing good metric consistency in checkerboard geometry.

### 4.4. Comparative Analysis

Table 14 reports a comparison between the final outcome of BSC, InterFil and OutAw algorithms in terms of the main performance indicators.

Performance gain of algorithms BSC → InterFil → OutAw: InterFil decreases calibration error by filtering out low quality pairs and OutAw increases performance with hard filtering, diversity-aware subset generation, multi-criteria ranking and final refinement for producing top stereo and geometric consistency results.

**Figure 19:**
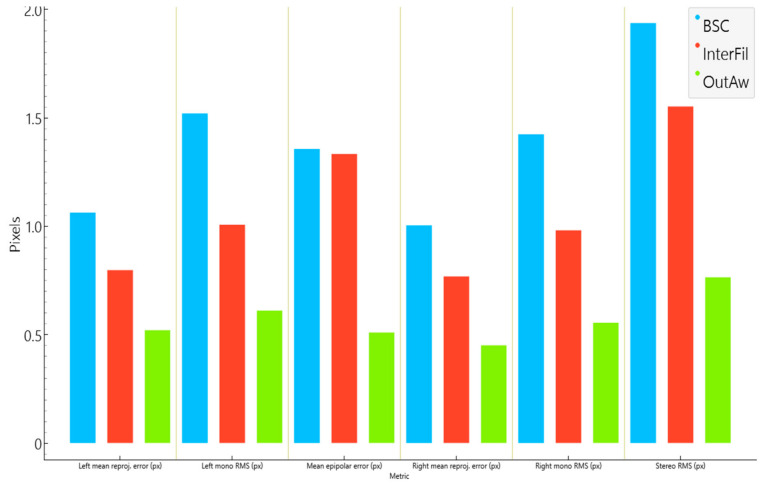
Stereo calibration accuracy across baseline and filtered algorithms.

According to presented results from Figure 19, OutAw decreased stereo RMS by 60.5% and mean epipolar error by 62.3% when compared to BSC. Compared to InterFil OutAw decreased stereo RMS by 0.7873 pixels (50.7% reduction) and mean epipolar error by 0.8234 pixels (61.7% reduction).

### 4.5. Bootstrap Stability Analysis Results

Table 15 reports the mean and standard deviation of stereo RMS, baseline, and focal length after 20 runs of bootstrap stability testing of the baseline calibration. The parameters exhibit measurable variability as there is a standard deviation of 0.0961 pixels for stereo RMS and 0.8242 mm for baseline. These results suggest that baseline calibration is not perfectly stable between resampled datasets.

Noticeable variation from run to run in the calculated parameters of the baseline calibration shows that the computed stereo model is not entirely stable but depends heavily on which images were chosen for inclusion in the bootstrap sample. Since the baseline solution appears to work but simply changes when given slightly different samples of the same dataset, this evidence advocates for more robust calibration methods which attempt to discount poor, unstable, or outlier image pairs.

Bootstrap analysis is not employed identically in the three methods. For BSC and InterFill, a bootstrap is used as a post-calibration robustness test, while for OutAw, a bootstrap is part of the subset-evaluation score for selecting the best calibration subset.

### 4.6. Robustness Under Contamination Results

Table 16 shows the output of the contamination study. Stereo calibration performance is tested as a function of the percentage of contaminated image pairs from 0% up to 30% while holding the total number of pairs constant at 34 pairs per test condition. The stereo RMS, mean epipolar error, estimated baseline and level of contamination are reported so the impact of incrementally corrupted input may be analyzed.

Calibration quality is seen to degrade smoothly as the contamination level increases. Stereo RMS error increases from 1.4078 px when there is no contamination to 1.5665 px at 10% contamination, 1.6455 px at 20% contamination and 1.9748 px at 30% contamination. Mean epipolar error does not change much from 0% to 20% contamination, bouncing around 1.28 to 1.31 px but is 1.4255 px at 30% contamination. Stereo RMS appears more sensitive to low and moderate contamination compared to mean epipolar error. However, as stronger contamination is introduced mean epipolar error begins to degrade as well, which suggests calibration quality decreases on a wider scale as more outlier pairs are introduced.

### 4.7. Comparative Analysis of BSC, InterFil, and OutAw Across Two Test Configurations

To further validate the OutAw method, a second test was performed. This test used 200 pairs. For the BSC method, after stereo pair checkerboard corner detection, only 155 pairs remains. After InterFill pair ranking, the number of pairs was reduced to 144.

Regarding OutAw algorithm, the starting point was 200 pairs, after weak pair rejection was kept 169 pairs. From the filtered pool was constructed subsets of 39 pairs with diversity constraints fixed at unique_ang_bins = 2, unique_img_bins = 4, and unique_z_bins = 3. The best ranked subset is refined into a final selection of 51 pairs. The 39 pair seeds were first ranked, and the best one was run_0029.

It was then refined into a 51 pair final set by keeping the strongest core pairs and adding a few complementary ones like pair_0027, pair_0051, pair_0161, pair_0178, and pair_0192 to improve overall stability and calibration quality. Table 17 shows a comparison between the outcome of BSC, InterFil and OutAw algorithms in for the two performed tests. The full data for the two tests are included in the Supplementary Materials, File S1 containing TEST1 results and File S2 containing TEST2 results.

The table compares three calibration methods (BSC, InterFil, and OutAw) across two tests, showing that OutAw consistently uses fewer but better-quality pairs and gives the best calibration accuracy. For example, in the second test it reduces stereo RMS to 0.8001 px and mean epipolar error to 0.4247 px, compared with 1.7868 px and 1.0862 px for the baseline values.

The OutAw workflow was deliberately developed to be quality-sensitive. The method only retains pairs that satisfy the criteria (sharpness, coverage, and geometric-consistency), and does not suppose that more pairs always guarantee better calibration. So, a smaller acquisition set containing 12–15 pairs may still be sufficient if the retained pairs are diverse and of high quality. In contrast, in poor-lighting experiments it was observed that all 70 pairs were rejected, highlighting the fact that the workflow was developed to halt when the data do not meet the minimum quality requirements. The next stage cannot be executed (if no pairs remain after hard filtering) and the workflow associated script triggers RuntimeError (“Not enough kept pairs for subset generation.”) to prevent calibration on an under constrained set.

Subset generation (in practice) needs a minimum of five retained pairs to ensure geometric diversity. For scenarios with only 12–15 initial pairs, it is recommended the verification that at least five image pairs pass the hard filtering before proceeding, and also the acquisition of additional pairs (if necessary).

## 5. Discussion

In terms of the main results, stereo calibration robustness relies less on the number but rather on the quality of the selected calibration pairs. Despite using all 49 valid pairs, Baseline stereo calibration (BSC) had a stereo RMS of 1.9385 pixels and an epipolar error of 1.3687 pixels, where OutAw reached 0.7666 pixels and 0.5119 pixels with only nine pairs which are improvements of 60.5% and 62.3%, respectively. This trend can be seen when observing the pair-level epipolar error distribution where a significant geometric inconsistency was observed with some of the rejected pairs; pair_0023 had a mean epipolar error of 5.3560 pixels which was over 17× larger than that of pair_0048 which only had 0.3108 pixels.

The results of subset-ranking show that the best calibration came from optimizing several quality measures simultaneously instead of one measure at a time. Run 15 had the lowest overall score (0.01296) and had good scores across all measures (stereo RMS, epipolar error, triangulation-based square error, square-length consistency, baseline stability). This points to the need to consider multiple criteria when ranking, in order to find the best geometrically consistent calibration subset for the OutAw algorithm.

The results obtained show that stereo calibration quality affects the digital twin quality since stereo calibration errors are transferred into disparity estimation and triangulation which result in inconsistencies in the geometry of the reconstructed virtual replica. Baseline stereo calibration (BSC) from all 49 valid stereo pairs resulted in a stereo RMS value of 1.9385 pixels with mean epipolar error of 1.3687 pixels, numbers that put baseline stereo quality in the weak range for accurate stereo reconstruction.

The intermediate filtered method (InterFil), which dropped seven noisy pairs from BSC, further lowered stereo RMS (1.5539 pixels) and epipolar error (1.3354 pixels). This shows that even simple data filtering improves calibration convergence, but epipolar performance was still above 1.0 pixel, indicating poor stereo correspondence.

OutAw produced the best results overall. It selected nine pairs left after hard filtering, multi-criteria subset ranking, and final refinement produced a stereo RMS of 0.7666 pixels and mean epipolar error of 0.5119 pixels. These results are improvements of 60.5% and 62.3% over BSC, respectively, and 50.7% and 61.7% over InterFil.

Stereo calibration analysis further supports the need for outlier-aware calibration for digital twin use cases. Synthetic contamination from 0% to 30% caused stereo RMS to increase by 40.3% while the estimated baseline also varied by over 4 mm. These results indicate that outliers, if not removed, progressively corrupt the calibration model even before performing 3D reconstruction.

Overall, the results validate OutAw approach of utilizing outlier rejection, stereo geometry validation, and triangulation-based metric validation as being more applicable to digital twin pipelines where geometric validity, scale consistency, and reconstruction stability are of utmost importance.

In Table 18 an OutAw algorithm performance comparison with existing calibration methods is presented.

OutAw should be seen primarily as an outlier-aware augmentation of traditional Zhang style, checkerboard-based stereo calibration, not as an independent family of calibrations. It maintained traditional OpenCV workflow reliant on checkerboard corner detection, subpixel refinement, monocular calibration, stereo calibration and rectification, but incorporated hard threshold filtering, diversity-aware subset generation, multi-criteria ranking, iterative refinement, bootstrap-based stability analysis, and triangulation-based geometric validation. This decision was validated by our results.

Compared to vanishing-point and other target-free methods, OutAw operates under a different calibration setting. Target-free methods appeal to in situ calibration settings where no dedicated pattern is necessary. OutAw assumes the checkerboard was captured under laboratory conditions and enabled stereo-specific evaluation of epipolar consistency, triangulated square size error, and square length variation.

Compared with camera-LiDAR calibration methods, OutAw solves a smaller but simpler problem. Camera-LiDAR methods are meant to handle heterogeneous sensor alignment for multimodal fusion systems, while OutAw is meant to be used on pure stereo camera systems, and so it focuses on stereo dataset quality control instead of inter-sensor registration. Compared with machine-learning approaches to stereo calibration, OutAw also has a different design goal as it utilizes deterministic statistical criteria rather than learned correction models, allowing for easy interpretation of its decisions without reliance on training data.

OutAw also differs from real-time recalibration and calibration monitoring approaches conceptually in use case as well as implementation. Recalibration approaches are meant to be used online to regain lost accuracy/fidelity during deployment, while monitoring approaches such as StOCaMo are designed to determine if a current calibration has lost accuracy. OutAw is instead an offline calibration generation methodology meant to output a robust reference calibration from a dedicated stereo dataset. For this reason, OutAw should be viewed as operating alongside methods such as ROCHADE: while ROCHADE provides improvements to checkerboard detection in challenging image conditions, OutAw provides improvements to downstream robustness after detection has occurred through pair rejection, subset ranking, and refinement.

Its contribution lies more in improving calibration robustness against imperfect stereo datasets through hard pair filtering, diversity-aware subset generation, multi-criteria ranking, and iterative refinement, not replacing Zhang-style calibration.

Regarding the computational cost, the OutAw algorithm uses an independent Python script for each step (corner detection, pair-features extraction, hard filtering, candidate subset generation, subset calibration, multicriteria ranking, iterative refinement, bootstrap stability). Each step has different complexities; for example, corner detection and other pre-processing steps have computational requirements linear with the number of pairs, while repeated calibrations, rankings, and other evaluations have an expensive computational cost.

In Table 19 the recorded runtimes are compared on the 49 valid stereo pairs. The stage mapping separates shared preprocessing from method specific stages.

BSC largest costs come from monocular calibration at 52.014 s and rectification at 33.582 s, InterFil reuses the shared detection and monocular calibration stages, the added runtime comes from outlier scoring, filtering, and recalibration. For the OutAw, the largest contributors are feature extractions at 65.731 s and subset evaluation at 28.590 s.

For computational complexity the notation meanings are:

- N is the number of processed stereo pairs;
- P is the number of pixels per image;
- C represents the number of checkerboard corners per image pair;
- I is the optimization iterations number used by the calibration routines;
- R is the number of candidate runs or evaluated candidate subsets;
- S is the subset size (the number of image pairs in one candidate subset);
- G is the diversity groups number used during subset generation;
- K is the number of kept pairs entering refinement;
- F is the final pairs number used in recalibration.

For the computational complexity (described in Table 20), BSC combines pixel image processing with calibration over all valid pairs. InterFill adds scoring, sorting and filtering and the dominant extra cost that comes from recalibration. OutAw is more selective, but the complexity cost depends on number of candidate subsets and respectively on their size. The heavy computational cost for OutAw is reduced compared to BSC because it is applied only on the remaining subsets (not on all pairs).

For the reported dataset (49 image pairs), the full OutAw pipeline required approximately 129 s on NVidia Jetson Nano (quad-core ARM Cortex-A57 CPU at up to 1.43 GHz, a 128-core Maxwell GPU, and 4 GB LPDDR4 memory with 25.6 GB/s bandwidth).

Comparing the OutAw (129 s) runs with the one of InterFil (131 s) and BSC (117 s), it can be stated that the computational cost (introduced by the multi-stage workflow) remains negligible in practice (despite its complexity).

There are a few limitations to note. First, the contamination experiments were based on artificial outliers and did not represent true degradation. As such, the 0%, 10%, 20%, and 30% contamination rates tested should be thought of as controlled robustness tests, rather than scenarios representative of online deployment. Second, OutAw is more computationally expensive than baseline because it considers multiple candidate subsets and iteratively refines subsets as implemented currently; this number can be decreased when computational budget is limited, at the expense of robustness. The third limitation is the comparison between the proposed solution and the OpenCV baseline, but a fair benchmarking cannot be made using only one or two indicators, without the methods’ reproducibility.

These limitations can suggest a few directions for future work. The first direction can be to further test the contamination experiments, using real challenging conditions, such as variable illumination and alternative calibration target geometries. For the second direction, an optimization of ranking and filtering stages with clear execution times. Third, to implement a comparison with other reproducible emerging methods. Another future work can be a direct benchmark using an implemented Digital Twin.

## 6. Conclusions

This paper reports a robust stereo calibration experiment targeting digital twin data acquisition under a regular checkerboard-based capture setup and applying three calibration methods: a baseline using all pairs strategy, an intermediary filtered approach, and the outlier-aware proposed calibration selection strategy. The reported results show that calibration quality consistently improves as it moves from baseline to more selective calibration selection strategies, with the last algorithm (OutAw) obtaining the highest calibration accuracy.

Results indicate that calibration robustness correlates more tightly with the geometric quality of selected image pairs, rather than the overall quantity of available pairs. Blindly collecting more calibration images will not necessarily provide a better solution if some images are blurry, weakly informative, or geometrically inconsistent. Conversely, a smaller subset will result in a calibration that is more stable, accurate, and useful downstream for 3D reconstruction.

OutAw provides an advantage by assessing calibration quality against richer criteria than reprojection error alone. Image-plane fit is supplemented with stereo alignment, triangulation-based geometric consistency, and stability under resampling. This means OutAw can provide a more holistic view of calibration reliability for use in digital twin workflows that require preservation of scale, geometric credibility, and reconstruction consistency in addition to low reprojection error.

While OutAw is compatible with existing camera models, its primary contribution is not a novel camera model but an explainable outlier rejection framework for stereo calibration. OutAw produces a calibration with increased reliability without sacrificing OpenCV-based workflow compatibility. As such, it can be recommended for use in digital twin acquisition pipelines where reliable geometry is valued over simply calibrating with all available images.

Optimization is relevant to digital twin data acquisition because camera data needs to enable visualization as well as geometry quality reconstruction, measurement, alignment, and downstream analytic use cases. Poor stereo calibration affects depth estimation and 3D reconstruction accuracy with scale, spatial inconsistency, and dimensional inaccuracies.

Future work could also tie OutAw more directly to the deployment of digital twins. This could be realized through assimilation to real-time data acquisition, integration with calibration monitoring approaches, and analysis of downstream 3D reconstruction accuracy with respect to real-world measurement tasks beyond checkerboard square consistency.

The current study was just focused on the digital twin calibration stage of data acquisition as opposed to a fully integrated digital twin. By limiting the scope, the paper concentrated on confirming the OutAw algorithm’s specific benefits as an offline stereo calibration solution. Since calibration robustness enhancement was the main goal of this paper, studying this contribution strictly within the bounds of a controlled refinement methodology applied to a standard checkerboard-based calibration workflow allowed for fewer system-level variables. In addition, real-time calibration, health monitoring, and full end-to-end digital twin deployment would have expanded the scope of this work past algorithm development and depreciated the clarity of the comparison.

## Figures and Tables

**Table 1:** Short description of camera calibration methods and techniques.

Calibration Method	Key Technique(s)/Tools	Performance/Accuracy	Applicability Domain	Citations
Planar pattern calibration	Zhang’s method, Levenberg–Marquardt optimization	Benchmark calibration performance	General calibration	[18,19,20,21]
GCC tools	BabelCalib, Basalt, Camodocal, Kalibr, MATLAB R2021a calibration toolbox, ROS calibration	DAOV < 100°; DAOV > 100°; KB-8	Ultra-wide cameras	[9]
Vanishing point-based calibration	Multi-stage Hough voting, fine-tuned vanishing points, collinearity constraints	30% RMSE decrease; 0.64% vertical error	Field calibration, 3D reconstruction	[18]
LiDAR Camera MLO	Edge matching, MLO, robust loss	0.3° rotation error; 5 cm translation error	AVs, sensor fusion	[22,23,24]
LiDAR-Camera Self-Calibration	SVD, iterative optimization, CNN projection	Real-time error detection and correction	AVs, robotics	[22,23]
Camera-LiDAR RSS	VOQ metrics, smart sampling	1–1.2 cm reprojection error; ~90 s calibration	AVs, multi-sensor integration	[24]
Binocular Structured Light Calibration	Zhang stereo calibration, light plane estimation, phase shifting decoding	0.053 mm reconstruction error (36.33% improvement)	3D reconstruction, precision manufacturing	[27]
On-the-Fly Stereo Recalibration	GD, disparity maximization, IMU scale	Depth error: 0.9182 ± 0.7464 mm; 7 iterations	AVs, real-time correction	[25,28]
ML-Based Stereo Calibration	RT, NN, U-V disparity planes	Very low RMSE/RAE; 300,000 training set	Pitch correction, 3D reconstruction	[26]
ROCHADE	Surface fitting, subpixel refinement, 7-step processing pipeline	80% detection rate > OpenCV; 0.21 mm error	Multi-camera setups, extreme poses, lens distortion	[21]
Subframe Synchronization	Time-calibrated video markers, interpolation algorithms	<17 ms error (<25 ms frame level limit)	Multi-camera systems, temporal precision	[29]
StOCaMo	Kernel correlation, variance-based resampling variance, SLAM error correlation	91–94% recall; 0.78 SLAM correlation	Real-time autonomous systems, de-calibration detection	[8]
Symbolic Regression for Calibration	GP, knowledge-guided discovery, interpretable models	Superior accuracy with limited data	General calibration, low data scenarios	[12]
Heterogeneous Lens Calibration	Chromatic checkerboard patterns, distortion models, and plane mapping	Zhang’s method accuracy with heterogeneous FOV handling	Wide/narrow-angle stereo systems	[18,19]
Dual-Calibration for Scene Reconstruction	Two parameter sets, one for 2D matching, one for 3D reconstruction	Superior dual-stage precision	Wide/narrow-angle stereo systems	[18,20]

**Table 2:** OutAw method, pair-level descriptors.

Feature Group	Specific Features
Geometric coverage	Checkerboard coverage ratio based on the detected area in the image
Stereo consistency	Normalized LR extent difference that measures coverage imbalance between two views
Image quality	Sharpness descriptors obtained from the stereo images (used in OutAw to reject out-of-focus images)
Pose descriptors	Translational and rotational descriptors used for populating multi-dimensional pose bins during viable subset generation
Reprojection statistics	Reprojection-error descriptors obtained from observed vs. projected checkerboard corners (the summary statistics include mean, median, 95th percentile, maximum error)
Epipolar statistics	Epipolar-error descriptors based on y-disparity after rectification (as defined: mean epipolar error and threshold-based)
Image-plane descriptors	Checkerboard centroid information used for image-plane binning in the diverse-pose subset generation stage

**Table 3:** OutAw hard filtering thresholds used in Stage 2.

Feature	Threshold	Action
Sharpness	≥40	Out-of-focus images rejection
Coverage	≥0.04	Insufficient coverage patterns rejection
Reprojection 95th percentile	≤1.5 px	Reject high reprojection error
Epipolar error	≤1.0 px	Stereo alignment error rejection
LR span difference	≤0.08	Reject coverage asymmetry

**Table 4:** Sensitivity of hard filtering to the sharpness threshold.

Sharpness	Coverage	Reproj_p95	Epipolar Error	LR_Span Difference	Number of Retained Pairs	Number of Rejected Pairs
40	0.04	1.5	1	0.08	9	40
100	0.04	1.5	1	0.08	9	40
200	0.04	1.5	1	0.08	9	40
300	0.04	1.5	1	0.08	9	40
400	0.04	1.5	1	0.08	7	42
500	0.04	1.5	1	0.08	3	46
600	0.04	1.5	1	0.08	1	48
700	0.04	1.5	1	0.08	0	49
800	0.04	1.5	1	0.08	0	49
900	0.04	1.5	1	0.08	0	49

**Table 5:** Sensitivity of hard filtering to the coverage threshold.

Sharpness	Coverage	Reproj_p95	Epipolar Error	LR_Span Difference	Number of Retained Pairs	Number of Rejected Pairs
40	0	1.5	1	0.08	9	40
40	0.02	1.5	1	0.08	9	40
40	0.04	1.5	1	0.08	9	40
40	0.08	1.5	1	0.08	9	40
40	0.1	1.5	1	0.08	9	40
40	0.12	1.5	1	0.08	7	42
40	0.14	1.5	1	0.08	6	43
40	0.18	1.5	1	0.08	0	49
40	0.2	1.5	1	0.08	0	49
40	0.24	1.5	1	0.08	0	49

**Table 6:** Weights and justification are used in the multi-criteria ranking score for candidate OutAw subsets.

Metric	Weight	Justification
Reprojection RMS	0.20	Image-plane reprojection quality
Epipolar error	0.25	Rectification quality
Triangulation error	0.25	3D geometric accuracy
Square variance	0.15	Intrinsic consistency
Stability	0.10	Robustness to data variations
Diversity bonus	0.05	Favors diverse image and pose

**Table 7:** Compared stereo calibration method descriptions.

Calibration Method	Description	Filtering	Sample Selection	Subset Optimization
BSC	Standard OpenCV with all-image pairs	No	No	No
InterFil	Pre-filtered data	Yes	No	No
OutAw	Outlier-aware robust sampling	Yes	Yes	Yes

**Table 8:** Stereo calibration quality assessment evaluation metrics.

Metric	Symbol	Interpretation
Mean epipolar error	e_epi_	Stereo rectification quality (px)
Stereo RMS reprojection error	e_rms_	Overall calibration accuracy (px)
Square lengths SD	s	Geometric robustness (mm)
Square absolute error	e_s_	Metric board consistency (mm)
Estimated baseline	b	Extrinsic consistency (mm)
Bootstrap parameter SD	-	Parameter repeatability

**Table 9:** Centralized results of the Baseline stereo calibration (BSC) algorithm.

Indicator	Value
Number of stereo pairs used for calibration	49
Number of pairs evaluated post-rectification	48
Image resolution	1280 × 720
Stereo RMS	1.9385 px
Baseline	134.9002 mm
Mean vertical epipolar error	1.3687 px
Median vertical epipolar error	0.8483 px
RMS Monocular Left Camera	1.5217 px
RMS Monocular Right Camera	1.4259 px
Intrinsic parameters of the left camera (fx1, fy1, cx1, cy1)	1262.2224, 1255.4895, 582.3646, 363.5142
Intrinsic parameters of the right camera (fx2, fy2, cx2, cy2)	1085.3653, 1078.9389, 560.0632, 343.5532
Lowest average epipolar error per pair	pair_0048: 0.3107 px
Second lowest mean epipolar error	pair_0050: 0.3846 px
Third lowest mean epipolar error	pair_0038: 0.3861 px
Highest average epipolar error per pair	pair_0023: 5.3560 px
Second largest mean epipolar error	pair_0020: 4.9750 px
Third largest mean epipolar error	pair_0022: 4.0305 px

**Table 10:** Centralized results of the InterFil algorithm.

Indicator	Value
Total number of pairs	49
Number of pairs retained	42
Rejected pairs	pair_0014, pair_0031, pair_0032, pair_0033, pair_0034, pair_0036, pair_0037
Ranking features	cov_mean/lr_span_diff_norm/reproj_mean
Baseline (mm)	132.9996
Stereo RMS (px)	1.5539
Mean vertical epipolar error (px)	1.3354
Left monocular RMS (px)	1.0088
Right monocular RMS (px)	0.9833
Left mean reprojection error (px)	0.7994
Right mean reprojection error (px)	0.7704
Best-ranked pair (name)	pair_0010
Best-ranked pair (score)	0.1788
Best-ranked pair (features)	0.1106/0.0381/0.3790
Lowest-ranked retained pair (name)	pair_0041
Lowest-ranked retained pair (score)	0.7365
Lowest-ranked retained pair (features)	0.1627/0.0946/1.4814
First rejected pair (name)	pair_0037
First rejected pair (score)	1.0094
Worst-ranked pair (name)	pair_0014
Worst-ranked pair (score)	1.9260
Worst-ranked pair (features)	0.1795/0.1194/3.8522

**Table 11:** Centralized results of the OutAw algorithm.

Indicator	Value
Candidate subsets	19
Subset size	6 pairs
Diversity per subset	unique_ang_bins = 2; unique_img_bins = 1; unique_z_bins = 3
Best initial subset	pair_0004, pair_0007, pair_0010, pair_0030, pair_0045, pair_0048
Best initial subset score	0.0129
Best subset fx1 std	9.0047
Best subset fy1 std	7.8935
Best subset fx2 std	4.8686
Best subset fy2 std	4.9959
Best subset stereo RMS std	0.0575
Final baseline	132.6164 mm
Final stereo RMS	0.7666 px
Final mean epipolar error	0.5119 px
Final mean square absolute error	0.1097 mm
Final mean square length std	0.1140 mm
Lowest per-pair epipolar mean	pair_0050: 0.2761 px
Highest per-pair epipolar mean	pair_0019: 1.1608 px
Lowest per-pair epipolar p95	pair_0004: 0.6418 px
Highest per-pair epipolar p95	pair_0047: 1.8909 px
Lowest triangulated z mean	pair_0004: 481.814
Highest triangulated z mean	pair_0010: 601.574

**Table 12:** The extracted pair-level features for each stereo pair.

Pair	Sharpness Mean	Coverage Mean	LR Span Diff	Reproj p95	Epipolar Mean	Pose Left z	Pose Right z
pair_0004	617.530	0.1684	0.0538	1.0942	0.6051	494.969	519.271
pair_0007	584.300	0.1654	0.0541	1.0618	0.5519	500.224	525.916
pair_0010	425.500	0.1106	0.0381	0.7859	0.8597	623.044	652.403
pair_0050	500.697	0.1545	0.0646	1.1031	0.3831	501.384	523.825

**Table 13:** Top five ranked OutAw candidate subsets.

Rank	Run	Score	Stereo RMS	Epipolar	Square Abs	Square Std	Baseline Std
1	15	0.01296	0.68953	0.51197	0.10974	0.11403	0.61054
2	12	0.03774	0.69238	0.52743	0.11109	0.11088	0.65847
3	9	0.07834	0.74857	0.55317	0.11046	0.11044	0.97048
4	2	0.08594	0.74524	0.55481	0.11121	0.11197	0.85919
5	3	0.10623	0.73919	0.55605	0.11198	0.11924	2.58939

**Table 14:** Comparative analysis of BSC, InterFil, and OutAw.

Metric	BSC	InterFil	OutAw
Number of pairs	49	42	9
Stereo RMS (px)	1.938538	1.553984	0.766671
Mean epipolar error (px)	1.3687	1.335440	0.511970
Estimated baseline (mm)	134.900222	132.999637	132.616423
Left mono RMS (px)	1.521796	1.008873	0.613386
Right mono RMS (px)	1.425922	0.983337	0.556867
Left mean reproj. error (px)	1.065098	0.799453	0.522820
Right mean reproj. error (px)	1.005837	0.770416	0.453040

**Table 15:** Bootstrap stability analysis (20 runs, baseline configuration).

Metric	Value
Stereo RMS mean	1.9217 pixels
Stereo RMS std	0.0961 pixels
Baseline mean	134.6410 mm
Baseline std	0.8242 mm
fx_1_ std	17.1312
fy_1_ std	17.5721
fx_2_ std	15.6590
fy_2_ std	15.0052

**Table 16:** Contamination study results.

Contamination	Pairs	Stereo RMS (px)	Epipolar (px)	Baseline (mm)	Contamination
0.0	34	1.4078	1.3040	131.4370	0.0
0.1	34	1.5665	1.2755	135.6261	0.1
0.2	34	1.6455	1.3096	132.9698	0.2
0.3	34	1.9748	1.4255	135.5329	0.3

**Table 17:** Comparative analysis of BSC, InterFil, and OutAw between the two tests.

Metric	BSC TEST1	BSC TEST2	InterFil TEST1	InterFil TEST2	OutAw TEST1	OutAw TEST2
Number of pairs	49	155	42	144	9	51
Stereo RMS (px)	1.9385	1.7868	1.5539	1.4386	0.7666	0.8001
Mean epipolar error (px)	1.3687	1.086161	1.335440	1.000038	0.511970	0.424658
Left mono RMS (px)	1.521796	1.312771	1.008873	0.991256	0.613386	0.609883
Right mono RMS (px)	1.425922	1.149412	0.983337	0.797427	0.556867	0.502707
Left mean reproj. error (px)	1.065098	0.966020	0.799453	0.795805	0.522820	0.514830
Right mean reproj. error (px)	1.005837	0.807778	0.770416	0.629822	0.453040	0.424507

**Table 18:** OutAw algorithm performance comparison with existing calibration methods.

Method Family	Relation to OutAw	Comparison to OutAw
Zhang-style planar calibration/standard checkerboard workflows	Same family; extended workflow	OutAw still employs checkerboard detection, subpixel refinement, mono calibration, stereo calibration and rectification; the new contribution is the data selection logic: hard filtering, subset search, ranking and refinement.
Wide-angle GCC tools	Different scope	GCC methods work to select appropriate camera models, targets and software to deal with wide angle and fisheye imaging. OutAw attempts to improve robustness within a standard stereo OpenCV pipeline.
Vanishing-point calibration	Alternative target strategy	Vanishing-point approaches allow target-less in situ calibration. In contrast OutAw aims for robust checkerboard-based stereo calibration through curation of the data used for calibration and validation.
Camera-LiDAR calibration	Different problem domain	LiDAR-based approaches solve heterogeneous sensor alignment within sensor fusion systems. OutAw is only concerned with stereo camera calibration, making it simpler and better suited to pure stereo cameras.
On-the-fly stereo recalibration	Complementary operational role	Real-time recalibration method allows degraded calibration to recover while the system is running. OutAw is an offline calibration generation approach that places importance on curation of the dataset and quality of reference calibration.
ML-based stereo calibration	Different design philosophy	ML-based methods learn correction models from data, whereas OutAw relies on deterministic geometric and statistical criteria. As a result, OutAw is more interpretable, but less adaptive.
ROCHADE checkerboard detection	Complementary stage	ROCHADE aims to improve checkerboard detection in challenging imaging conditions, where OutAw improves robustness after detection by rejecting weak pairs and ranking candidate calibration subsets.
Calibration monitoring methods	Different objective	Monitoring approaches such as StOCaMo can observe decalibration during operation. OutAw is designed to provide high-quality initial calibration, not actively monitor a calibration that may exist.

**Table 19:** Stage-level runtime identification for each method.

Method	Stage	Script Runtime (s)	Role in Pipeline
BSC	Corner detection	28.312	Detect checkerboard corners and assemble the valid stereo correspondence set.
BSC	Monocular calibration	52.014	Estimate left and right intrinsics and compute reprojection statistics.
BSC	Stereo calibration	3.226	Estimate stereo extrinsics and rectification transforms.
BSC	Rectification and evaluation	33.582	Rectify all valid pairs and compute epipolar evaluation metrics.
InterFil	Corner detection	28.312	Shared detection for building the input dataset.
InterFil	Monocular calibration	52.014	Shared initialization before pair-quality filtering.
InterFil	Outlier scoring, filtering, and recalibration	50.791	Score views, reject lower-quality pairs, and recalibrate on the retained subset.
OutAw	Corner detection	28.312	Shared detection for building the input dataset.
OutAw	Feature extraction	65.731	Compute quality, geometric, pose, and sharpness features for each pair.
OutAw	Hard filtering	0.563	Apply threshold-based rules to reject unsuitable pairs.
OutAw	Candidate subset generation	0.652	Build diverse candidate calibration subsets from the retained pool.
OutAw	Subset calibration	3.149	Calibrate each candidate subset.
OutAw	Subset evaluation	28.590	Evaluate each candidate subset with geometric performance criteria.
OutAw	Ranking	0.145	Aggregate metrics, compute final scores, and select the best candidate subset.
OutAw	Refinement	3.233	Re-score the kept pairs and run final recalibration on the selected refined subset.

**Table 20:** The total runtime and computational complexity for each method.

Method	Total Runtime (s)	Overall Complexity
BSC	117.136	Approximately O(N⋅P) + O(N⋅C⋅I).
InterFil	131.118	Approximately O(N⋅P) + O(N⋅C⋅I) + O (N⋅log N)
OutAw	129.017	Approximately O(N⋅P) + O(N⋅C⋅I) + O(R⋅(NlogN + G + S)) + O(R⋅S⋅C⋅I) + O(R⋅N⋅C) + O(K⋅C + F⋅C⋅I).

## Data Availability

The original contributions presented in this study are included in the article’s Supplementary Materials. Further inquiries can be directed to the corresponding author.

## Supplementary Materials

The following supporting information can be downloaded at https://www.mdpi.com/article/10.3390/jimaging12070280/s1. File S1 (first test): contains the image pairs and the results for the initial test with 50 captured pairs; File S2 (second test):contains the image pairs and the results for the final test with 200 captured pairs.

## Abbreviations

The following abbreviations are used in this manuscript:

AVs	Autonomous Vehicles
BSC	Baseline Stereo Calibration
CAD	Computer-Aided Design
CNN	Convolutional Neural Network
DAOV	Diagonal Angle of View
DT	Digital Twin
FOV	Field of View
GCC	Geometric Camera Calibration
GD	Gradient descent optimization
GP	Genetic programming
IDs	Identifiers
IMU	Inertial Measurement Unit
IMU scale	External sensor scale correction
InterFil	Intermediate Filtered Method
IoT	Internet of Things
KB-8	Kannala-Brandt model with eight parameters
LiDAR	Light Detection and Ranging
LR	Left–Right
ML	Machine Learning
MLO	Multi-Level Parameter Optimization
NN	Neural Networks
OpenCV	Open Source Computer Vision Library
OutAw	Outlier-Aware Method
RAE	Relative Absolute Error
RMS	Root Mean Square
RMSE	Root Mean Square Error
ROCHADE	Robust Checkerboard Advanced Detection
ROS	Robot Operating System
RQ	Research Question
RSS	Robust Sample Selection
RT	Regression Trees
SD	Standard Deviation
SLAM	Simultaneous Localization and Mapping
StOCaMo	Stereo Online Calibration Monitoring
SVD	Singular Value Decomposition
USB	Universal Serial Bus
VOQ	Variability of Quality
