# Supplementary figures and images for: Quantifying and Improving Stereo Camera Calibration Robustness: An Outlier-Aware Algorithm for Digital Twin Data Acquisition (part 2 of 2)
Source: J Imaging. 2026 Jun 25;12(7):280. doi: 10.3390/jimaging12070280 (PMC13413186; doi:10.3390/jimaging12070280)

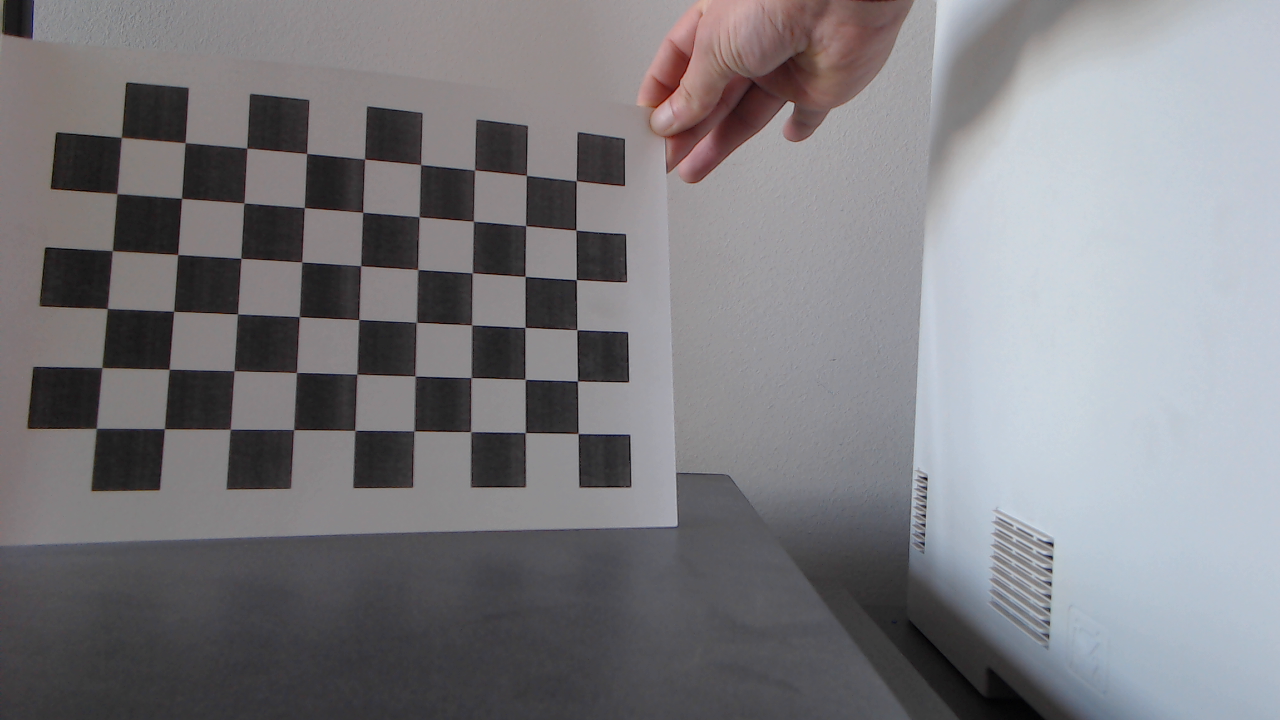

Supplement: Supplementary file 1 [file jimaging-12-00280-s001.zip › Supplementary Materials/first test/Pairs/raw/right/pair_0029_right.png]

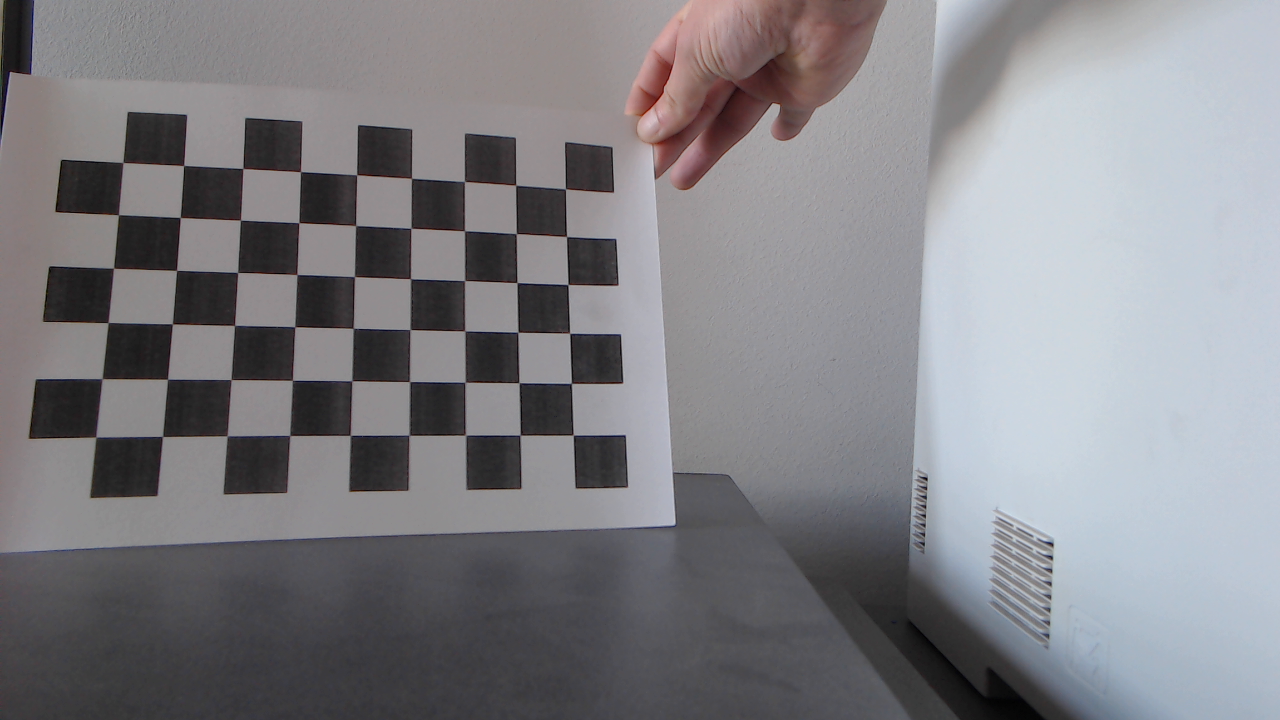

Supplement: Supplementary file 1 [file jimaging-12-00280-s001.zip › Supplementary Materials/first test/Pairs/raw/right/pair_0030_right.png]

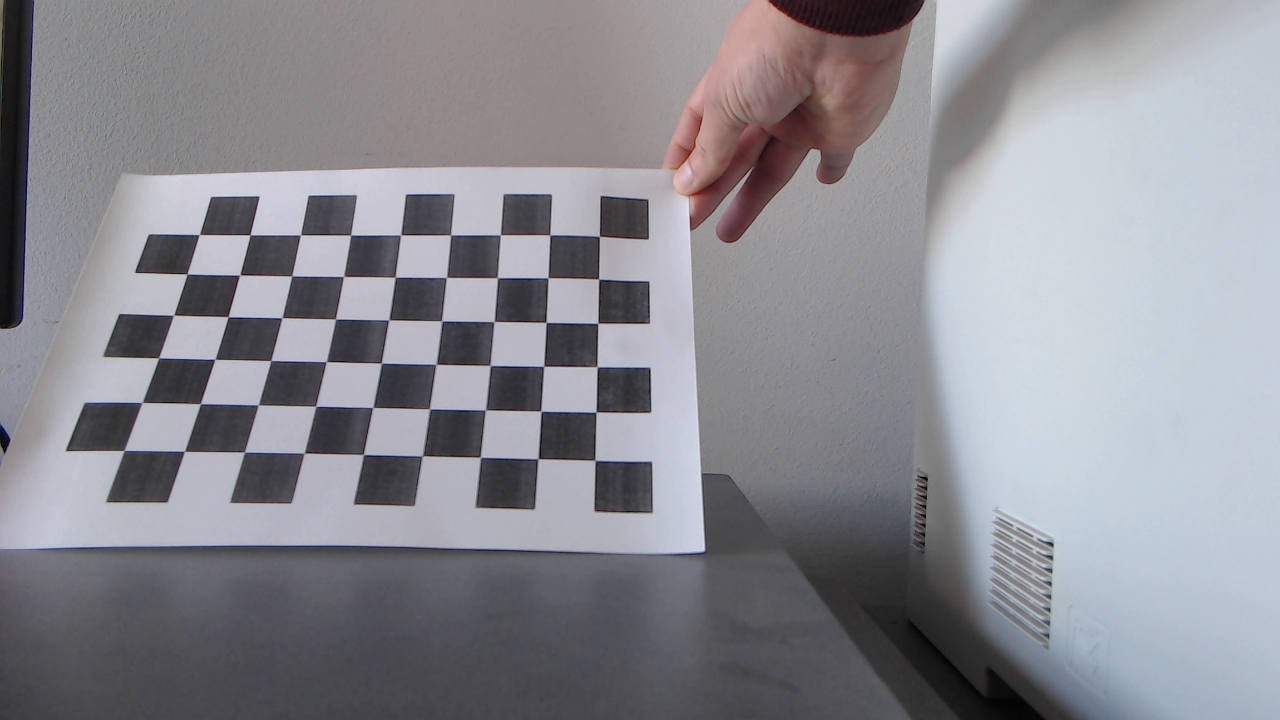

Supplement: Supplementary file 1 [file jimaging-12-00280-s001.zip › Supplementary Materials/first test/Pairs/raw/right/pair_0031_right.png]

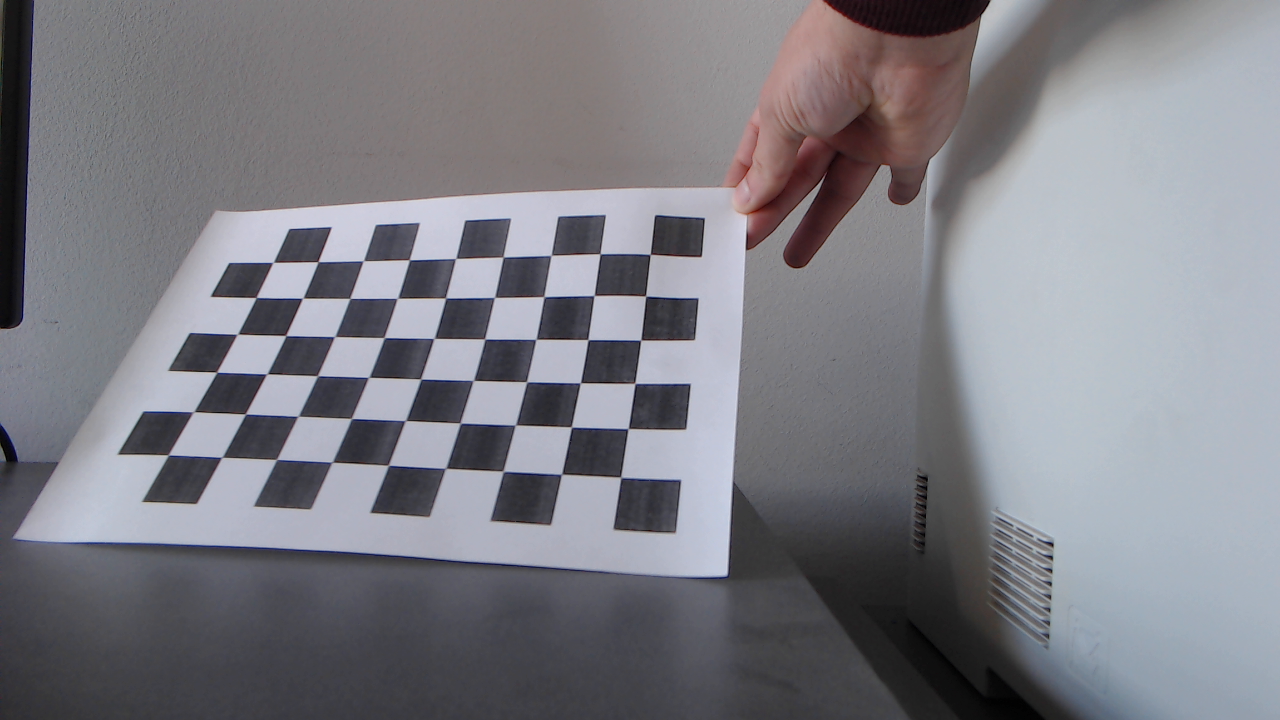

Supplement: Supplementary file 1 [file jimaging-12-00280-s001.zip › Supplementary Materials/first test/Pairs/raw/right/pair_0032_right.png]

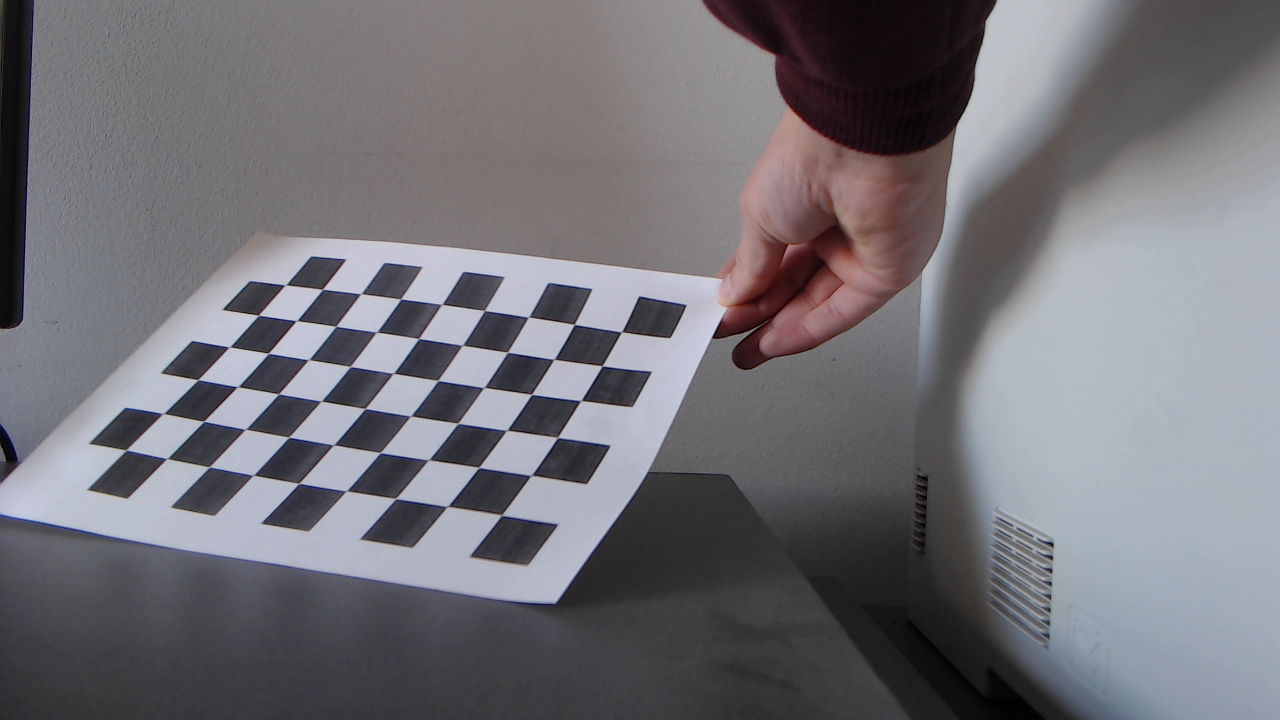

Supplement: Supplementary file 1 [file jimaging-12-00280-s001.zip › Supplementary Materials/first test/Pairs/raw/right/pair_0033_right.png]

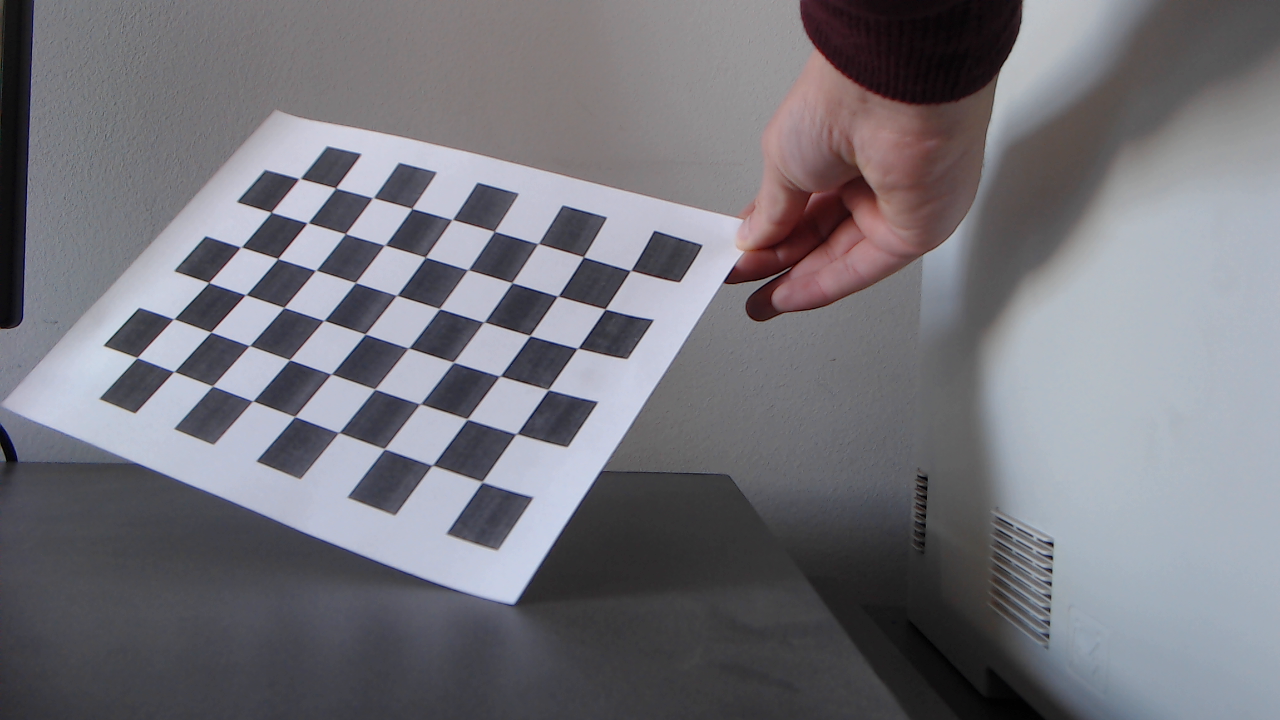

Supplement: Supplementary file 1 [file jimaging-12-00280-s001.zip › Supplementary Materials/first test/Pairs/raw/right/pair_0034_right.png]

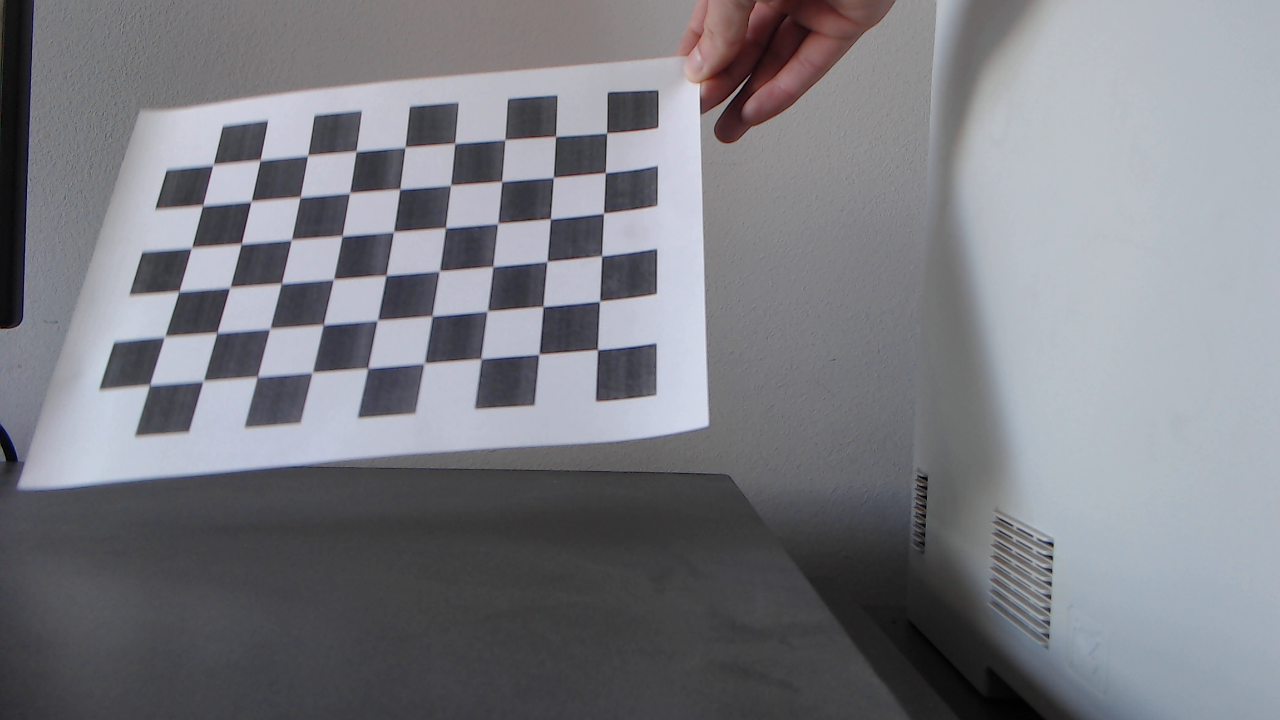

Supplement: Supplementary file 1 [file jimaging-12-00280-s001.zip › Supplementary Materials/first test/Pairs/raw/right/pair_0035_right.png]

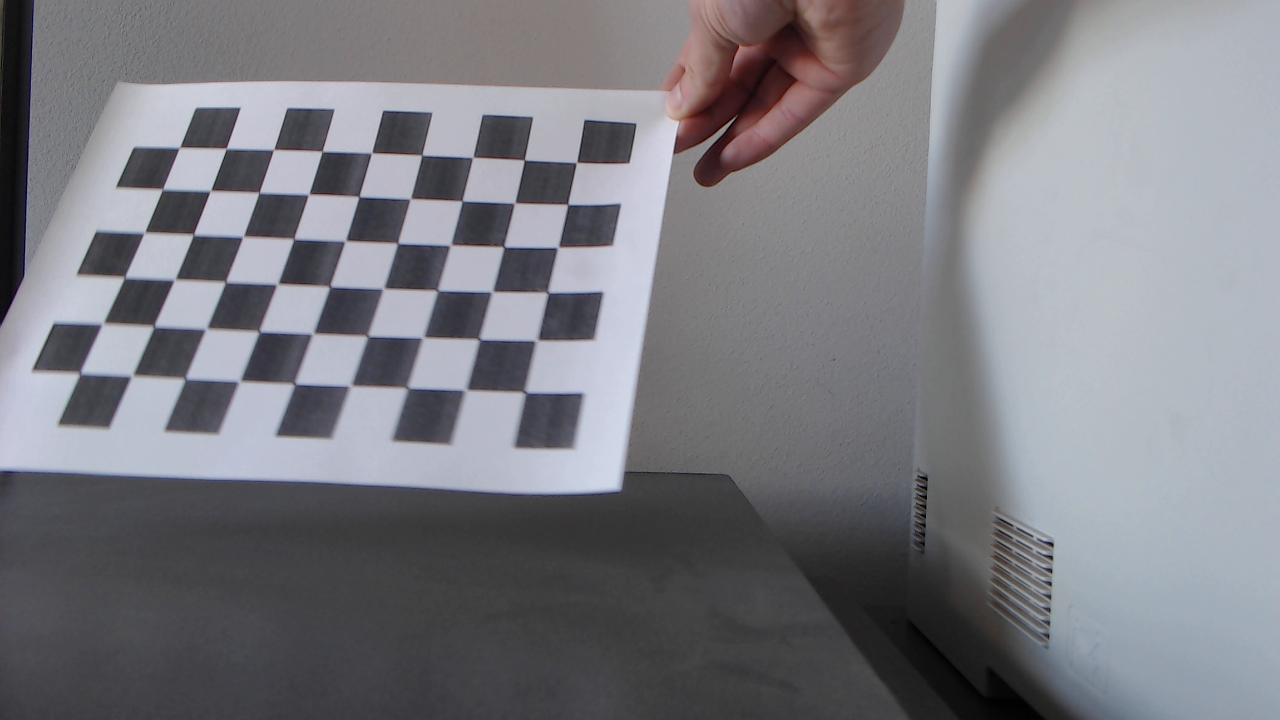

Supplement: Supplementary file 1 [file jimaging-12-00280-s001.zip › Supplementary Materials/first test/Pairs/raw/right/pair_0036_right.png]

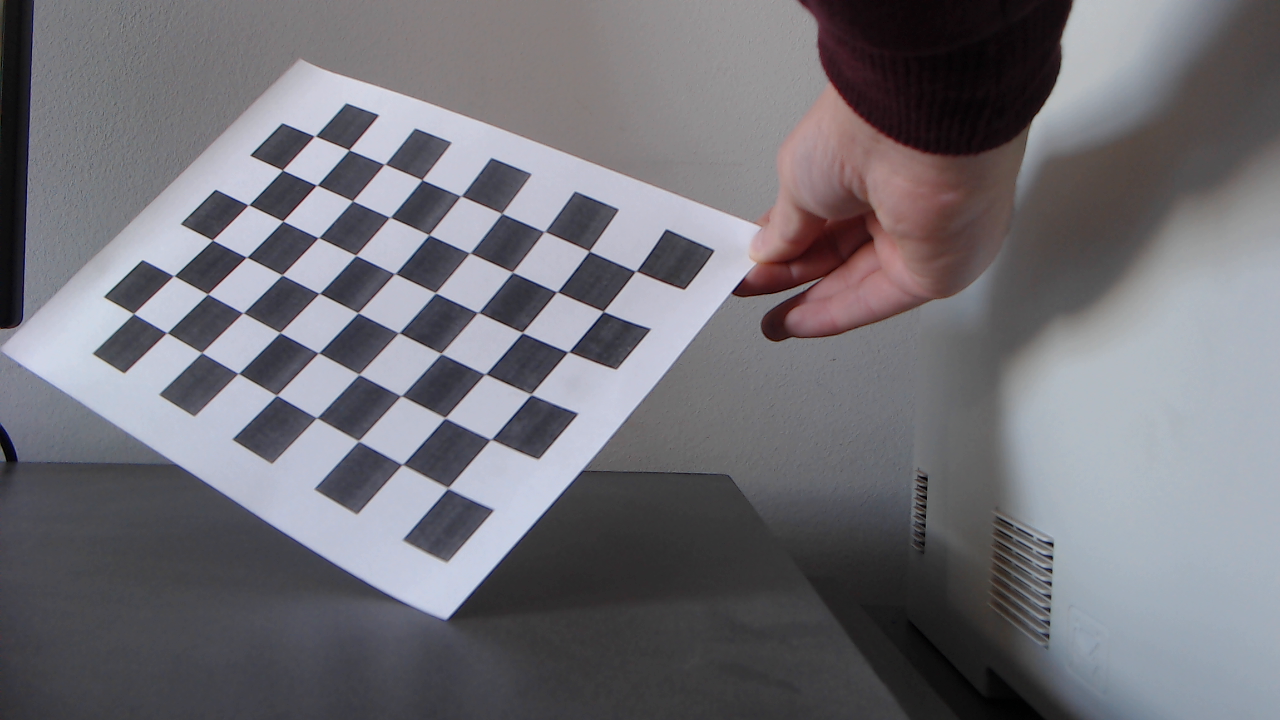

Supplement: Supplementary file 1 [file jimaging-12-00280-s001.zip › Supplementary Materials/first test/Pairs/raw/right/pair_0037_right.png]

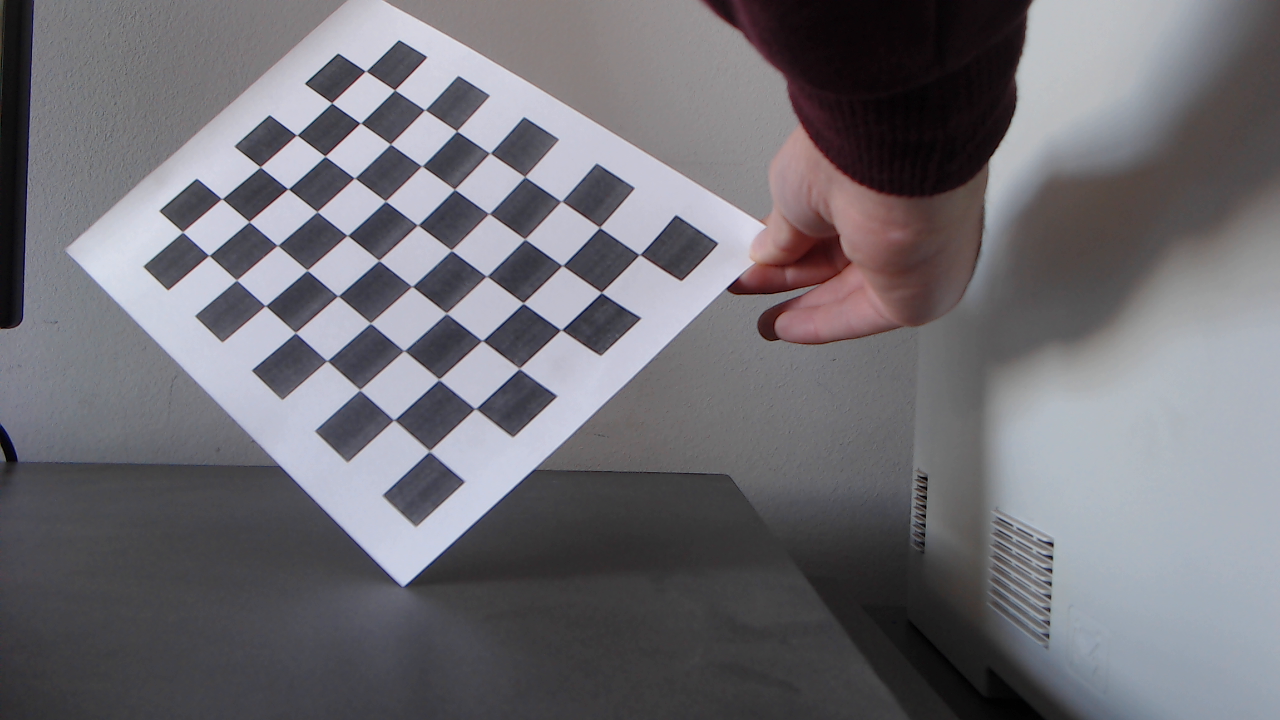

Supplement: Supplementary file 1 [file jimaging-12-00280-s001.zip › Supplementary Materials/first test/Pairs/raw/right/pair_0038_right.png]

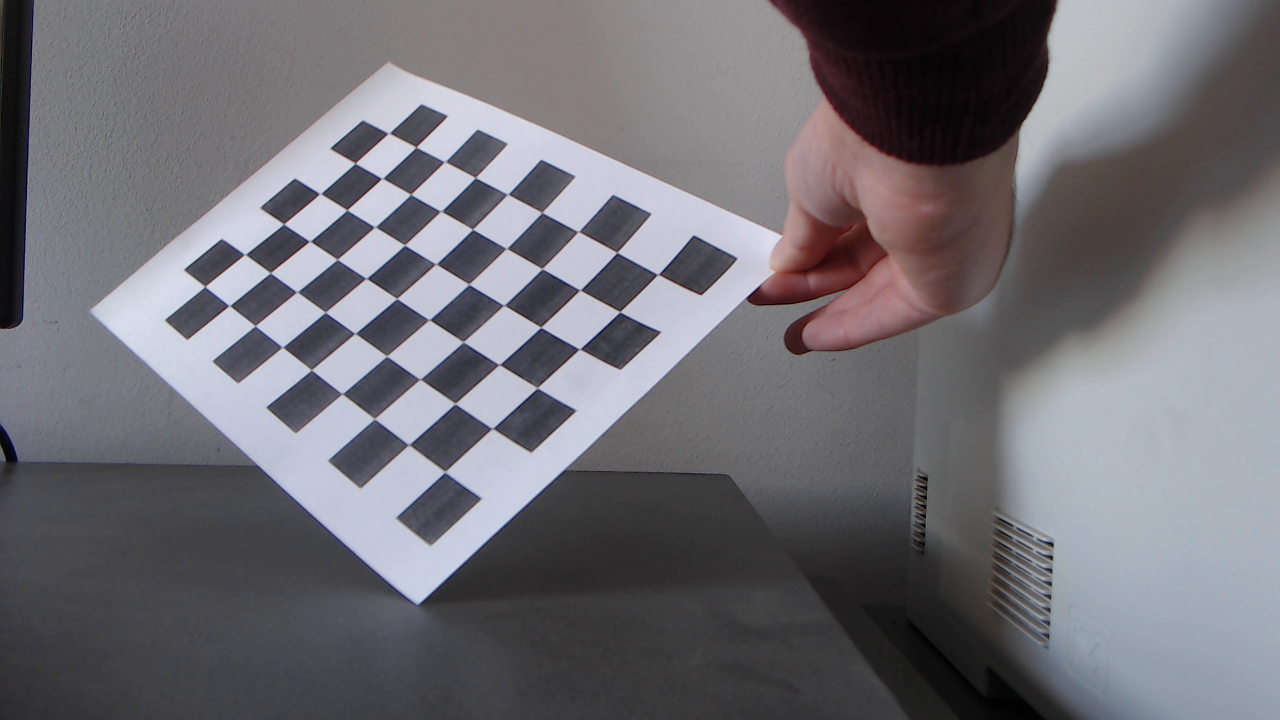

Supplement: Supplementary file 1 [file jimaging-12-00280-s001.zip › Supplementary Materials/first test/Pairs/raw/right/pair_0039_right.png]

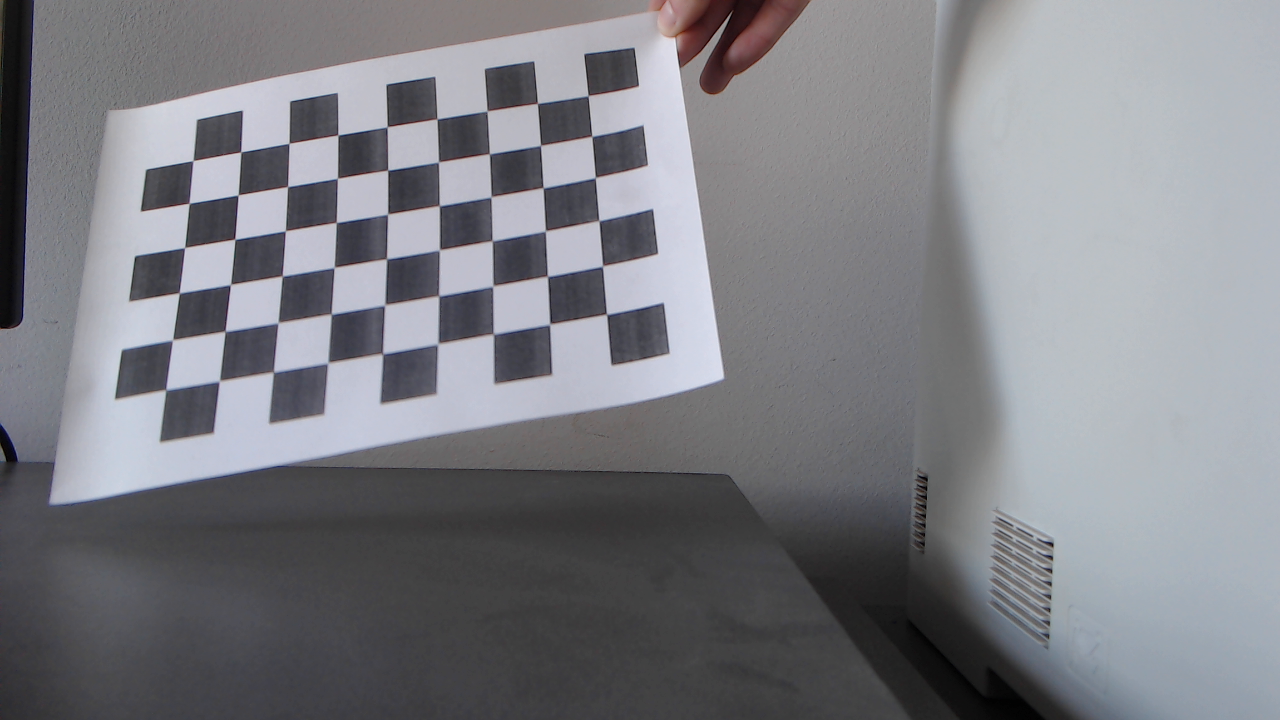

Supplement: Supplementary file 1 [file jimaging-12-00280-s001.zip › Supplementary Materials/first test/Pairs/raw/right/pair_0040_right.png]

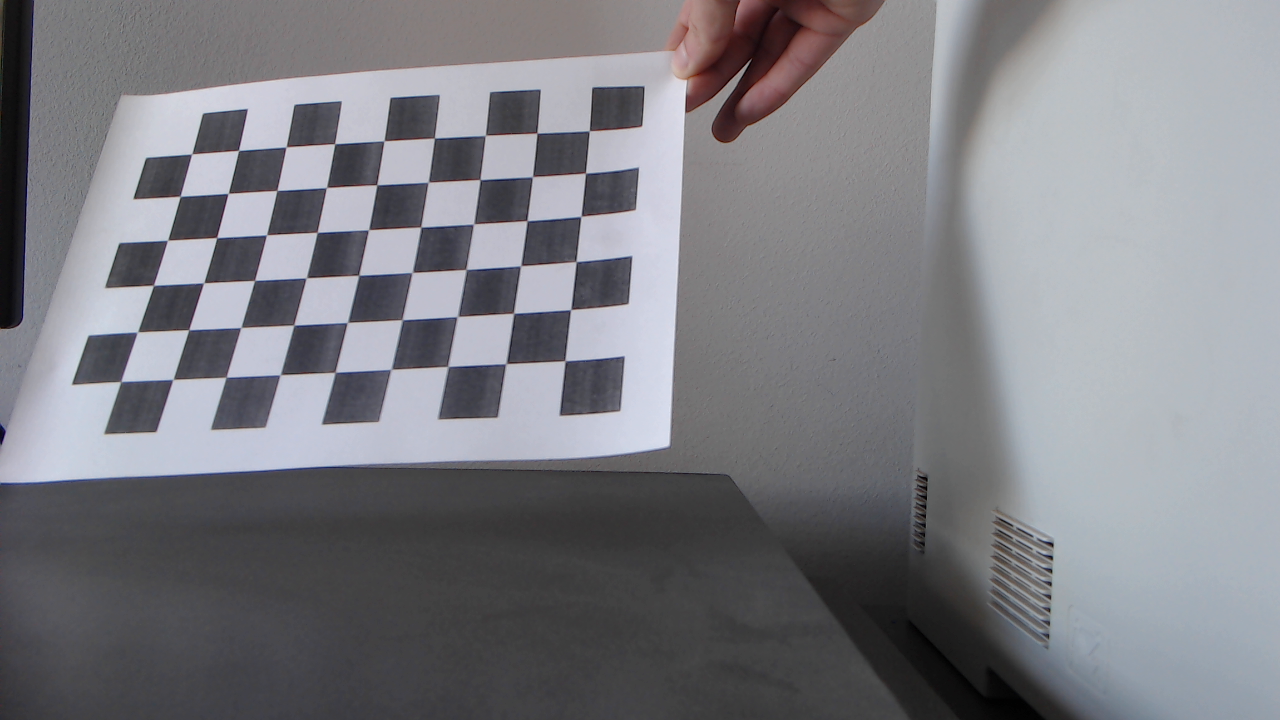

Supplement: Supplementary file 1 [file jimaging-12-00280-s001.zip › Supplementary Materials/first test/Pairs/raw/right/pair_0041_right.png]

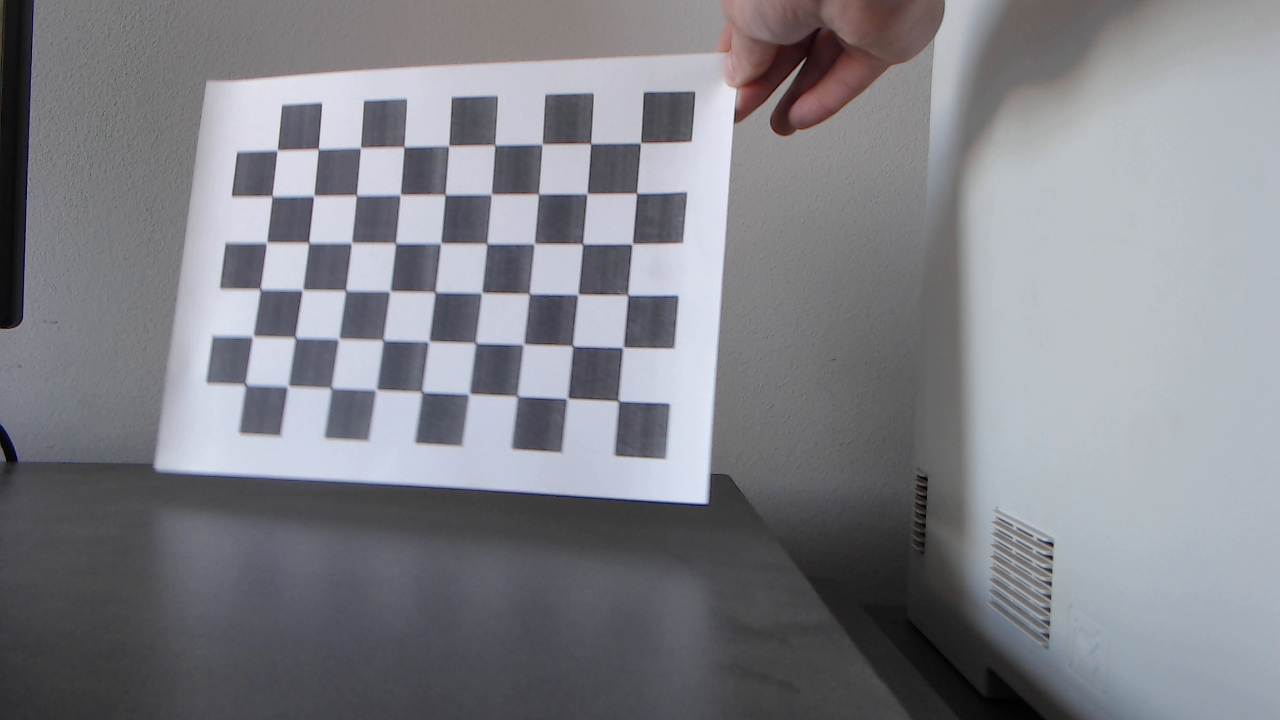

Supplement: Supplementary file 1 [file jimaging-12-00280-s001.zip › Supplementary Materials/first test/Pairs/raw/right/pair_0042_right.png]

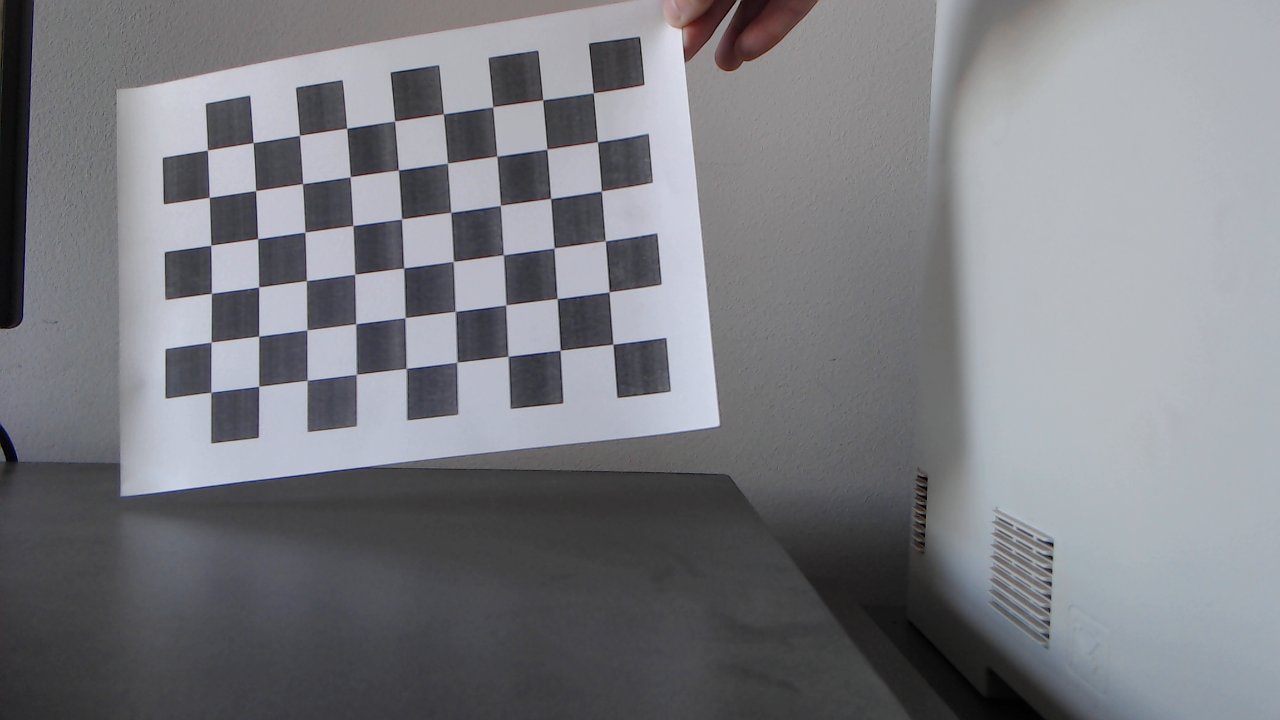

Supplement: Supplementary file 1 [file jimaging-12-00280-s001.zip › Supplementary Materials/first test/Pairs/raw/right/pair_0043_right.png]

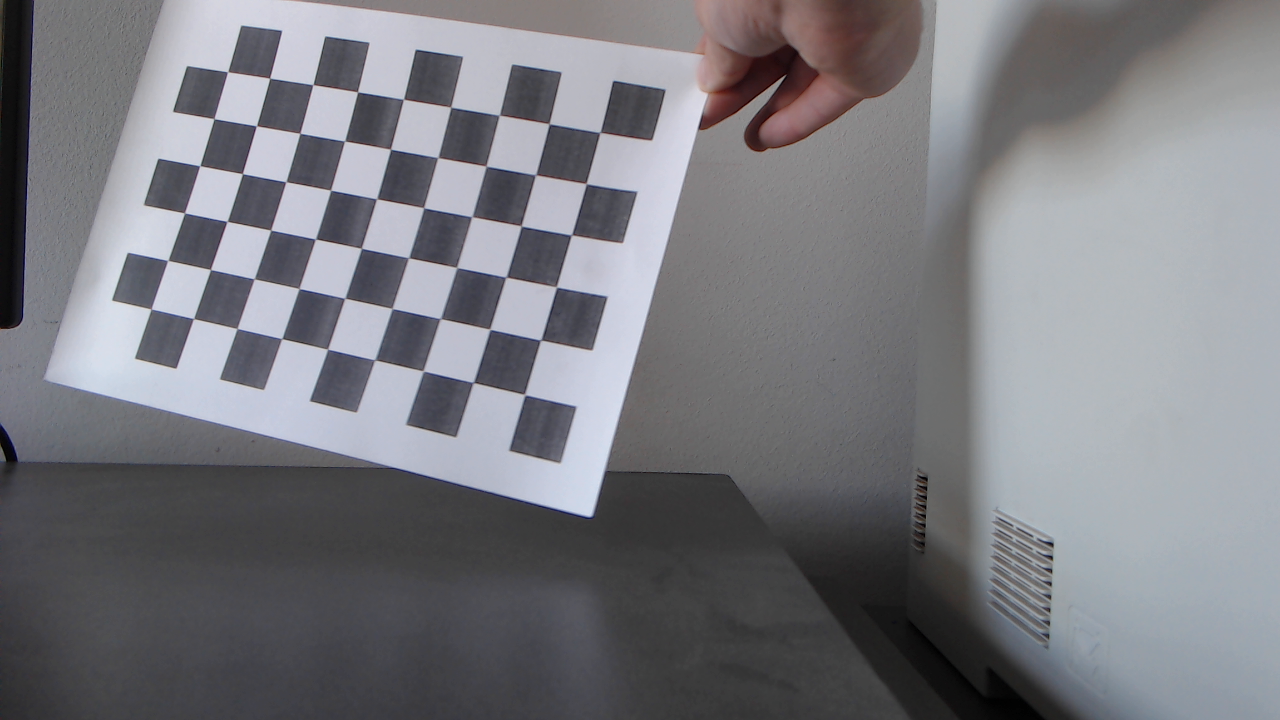

Supplement: Supplementary file 1 [file jimaging-12-00280-s001.zip › Supplementary Materials/first test/Pairs/raw/right/pair_0044_right.png]

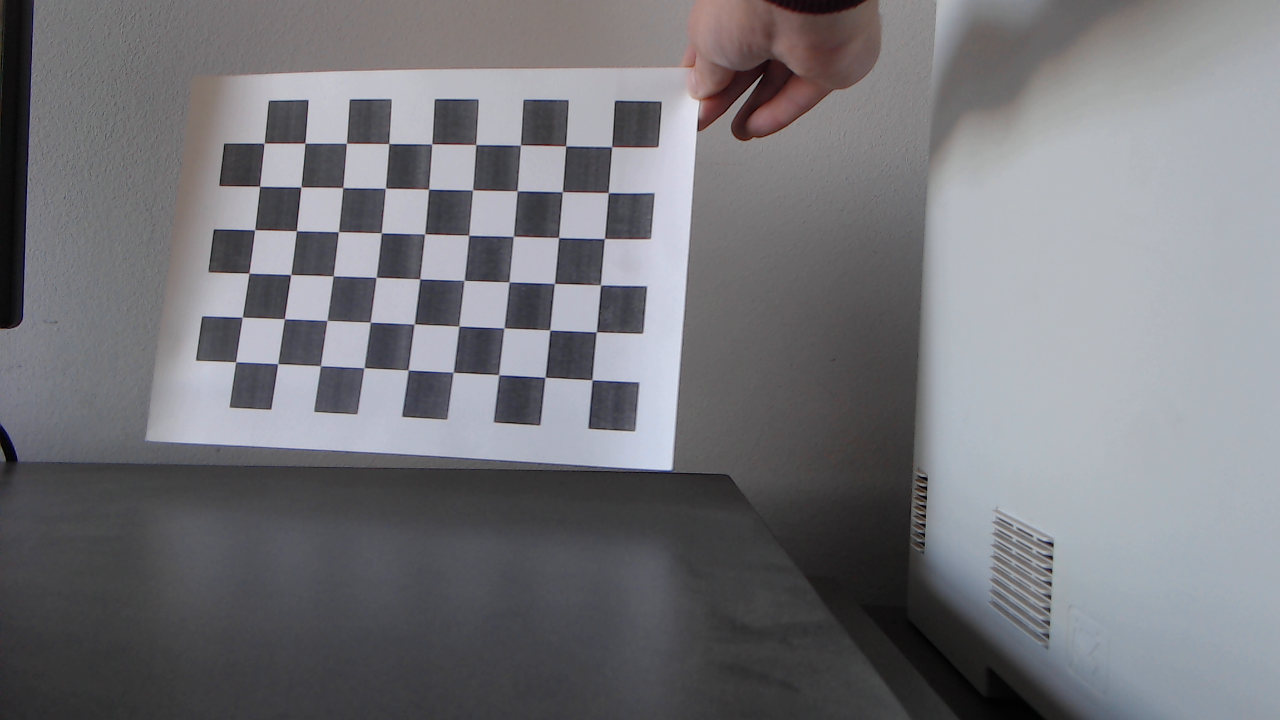

Supplement: Supplementary file 1 [file jimaging-12-00280-s001.zip › Supplementary Materials/first test/Pairs/raw/right/pair_0045_right.png]

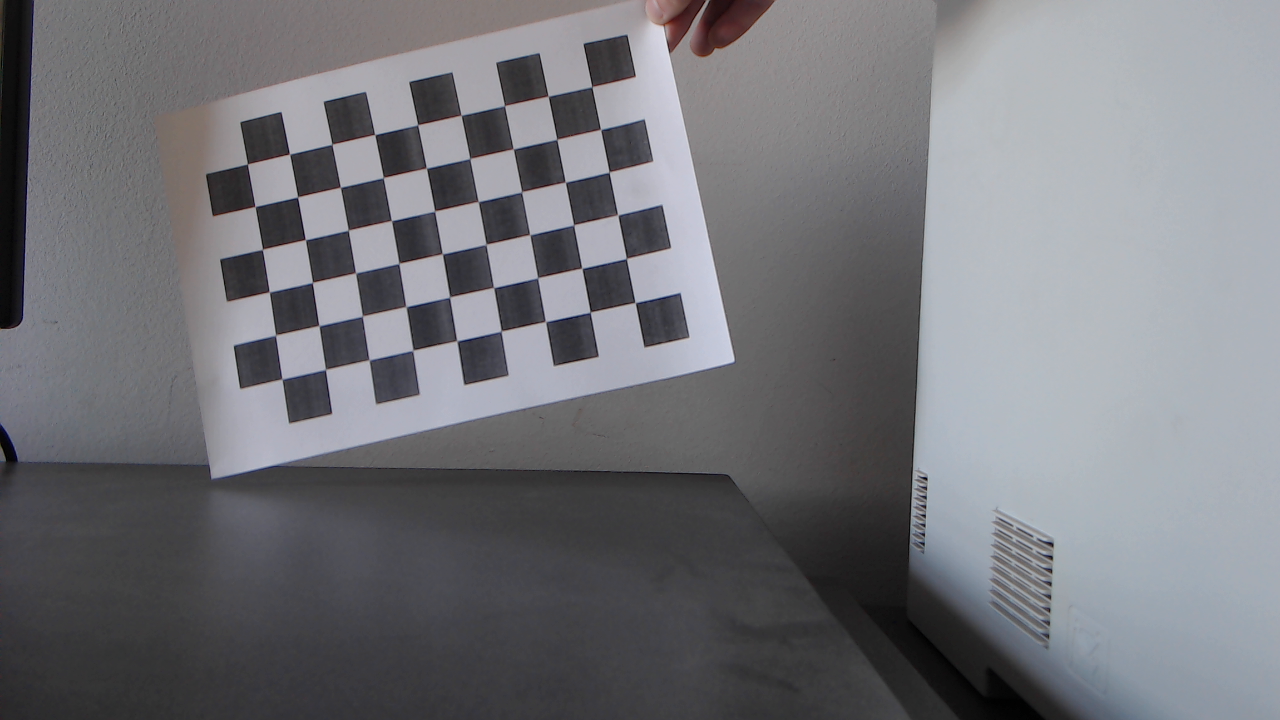

Supplement: Supplementary file 1 [file jimaging-12-00280-s001.zip › Supplementary Materials/first test/Pairs/raw/right/pair_0046_right.png]

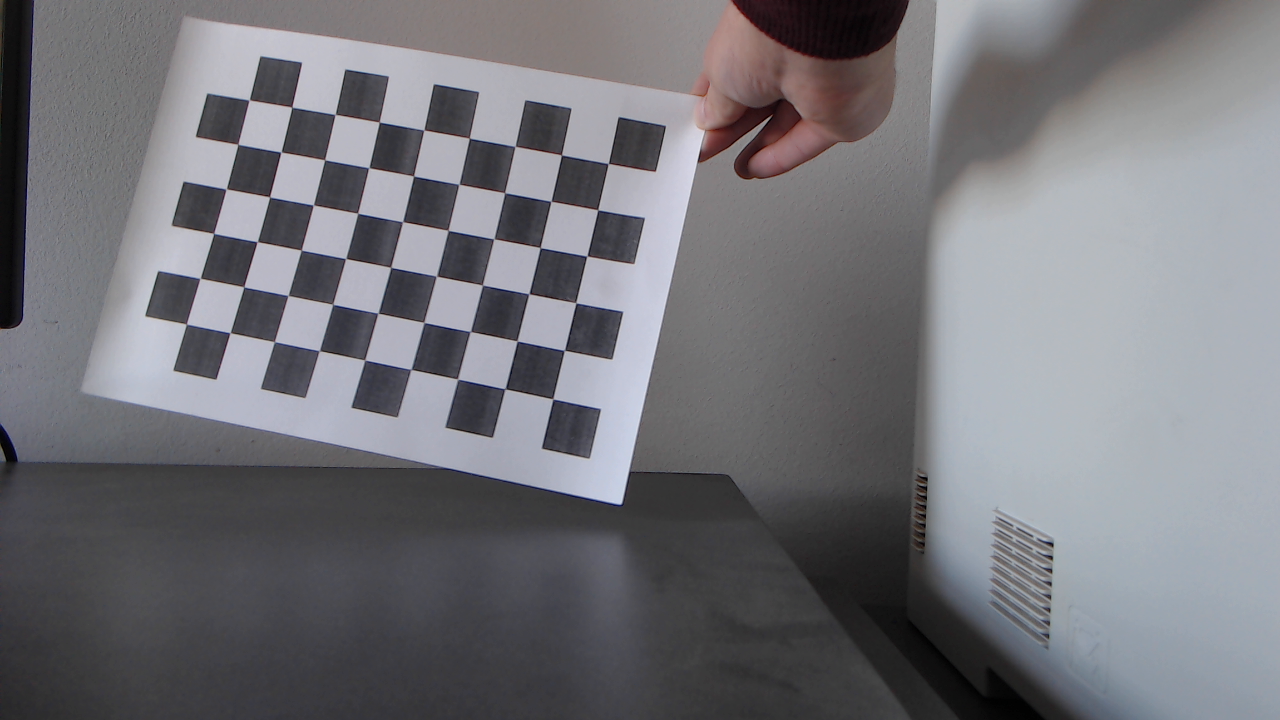

Supplement: Supplementary file 1 [file jimaging-12-00280-s001.zip › Supplementary Materials/first test/Pairs/raw/right/pair_0047_right.png]

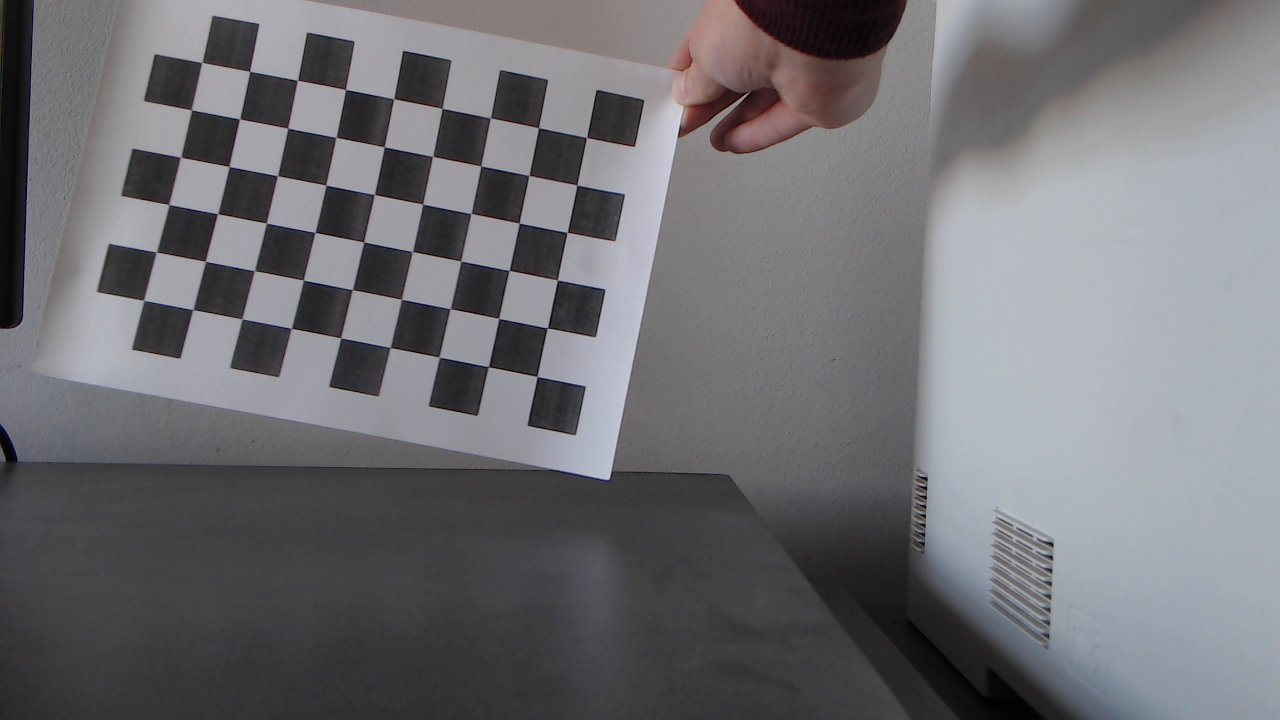

Supplement: Supplementary file 1 [file jimaging-12-00280-s001.zip › Supplementary Materials/first test/Pairs/raw/right/pair_0048_right.png]

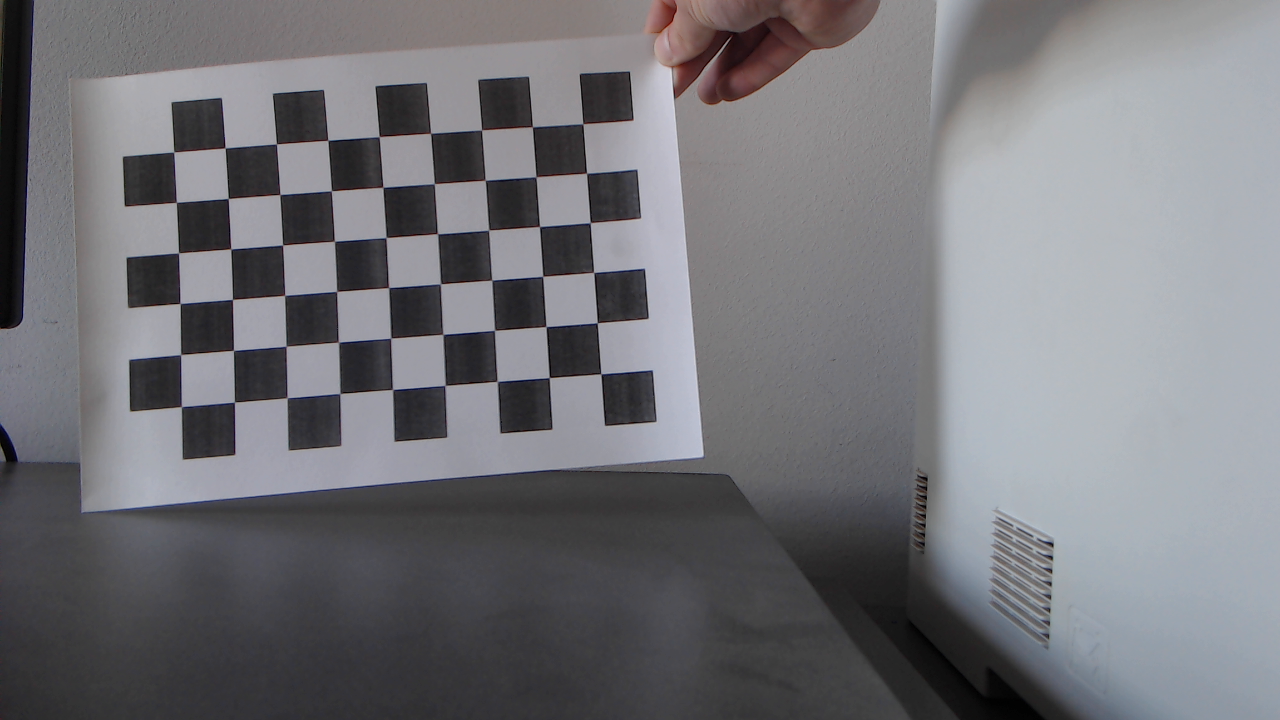

Supplement: Supplementary file 1 [file jimaging-12-00280-s001.zip › Supplementary Materials/first test/Pairs/raw/right/pair_0049_right.png]

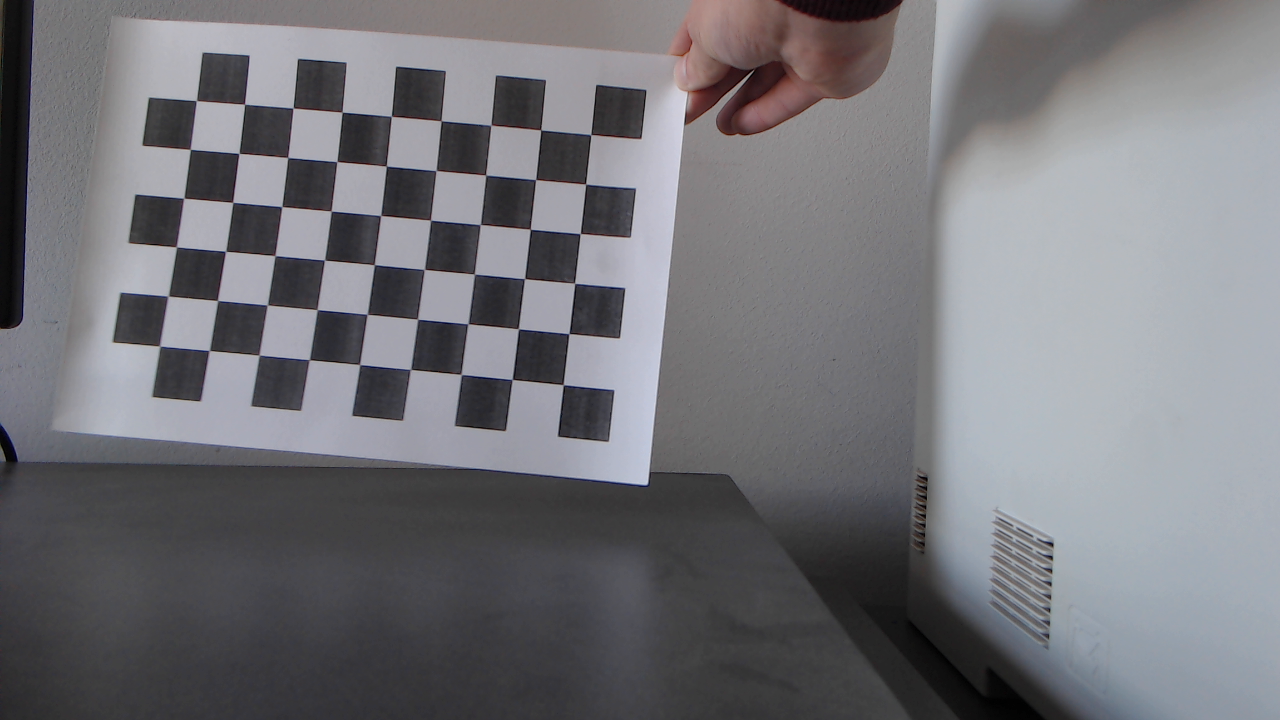

Supplement: Supplementary file 1 [file jimaging-12-00280-s001.zip › Supplementary Materials/first test/Pairs/raw/right/pair_0050_right.png]

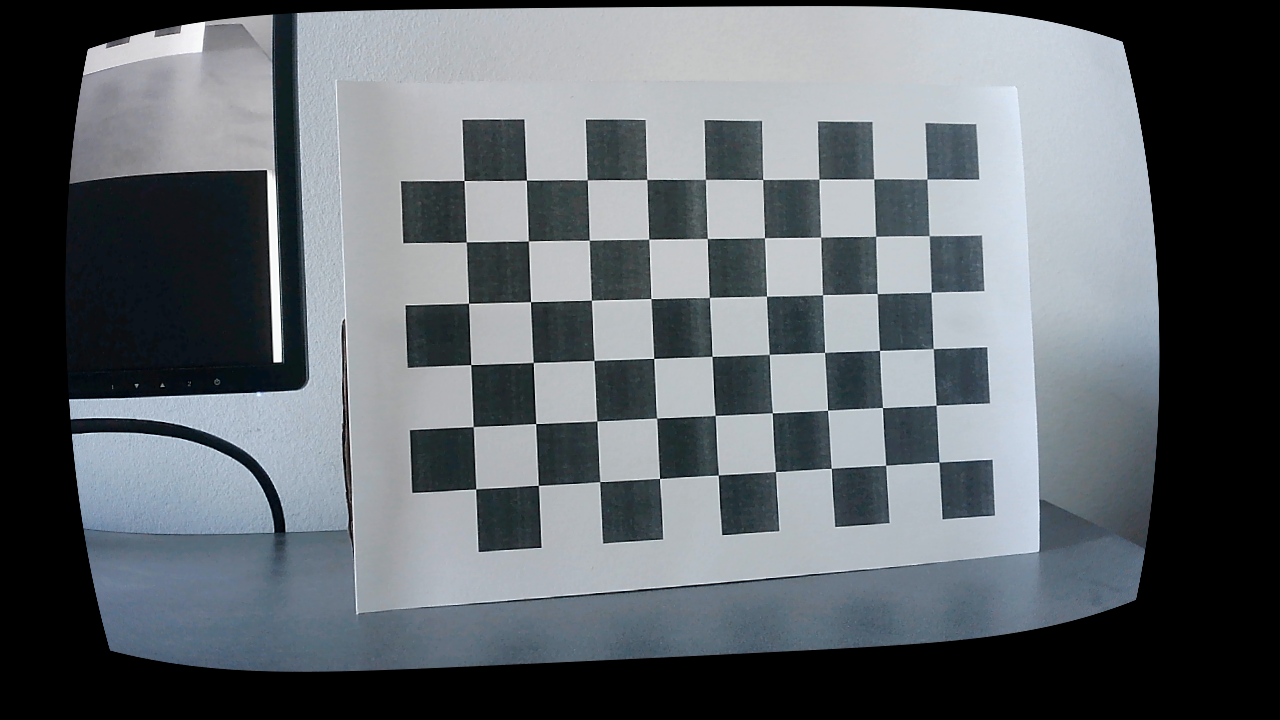

Supplement: Supplementary file 1 [file jimaging-12-00280-s001.zip › Supplementary Materials/first test/results/OutAw_runs/rectified_left/pair_0004_left.png]

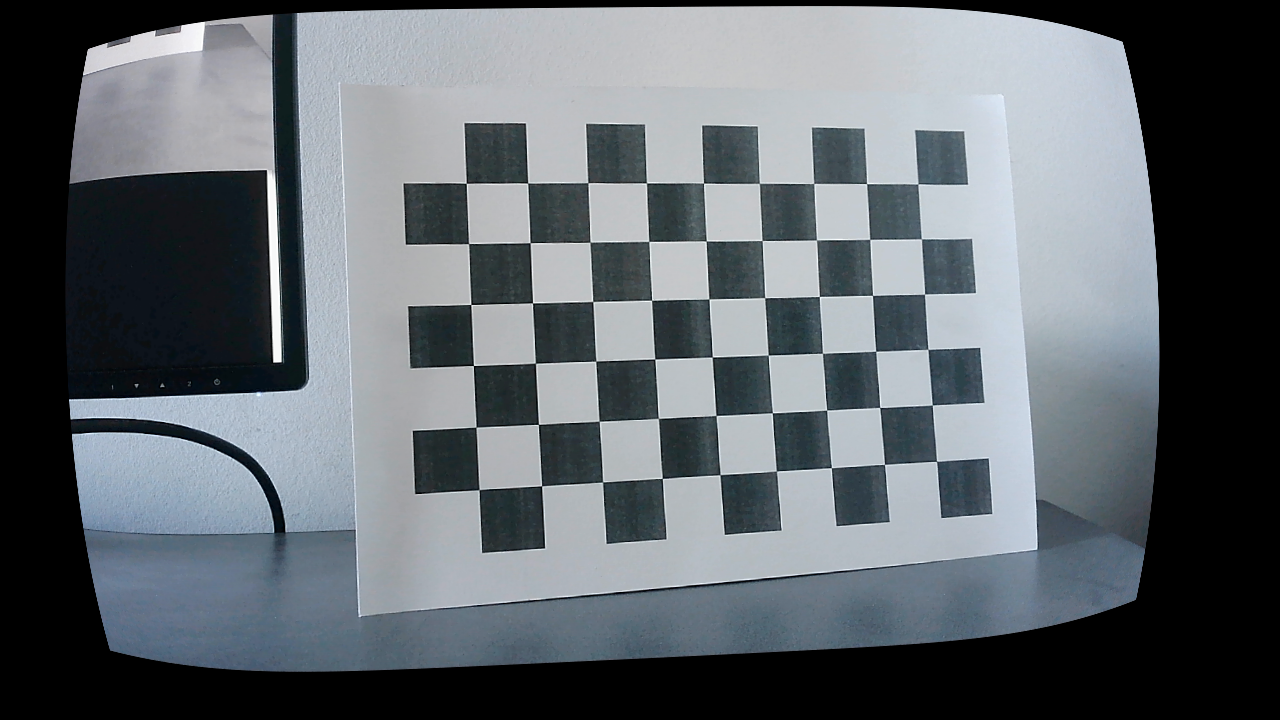

Supplement: Supplementary file 1 [file jimaging-12-00280-s001.zip › Supplementary Materials/first test/results/OutAw_runs/rectified_left/pair_0007_left.png]

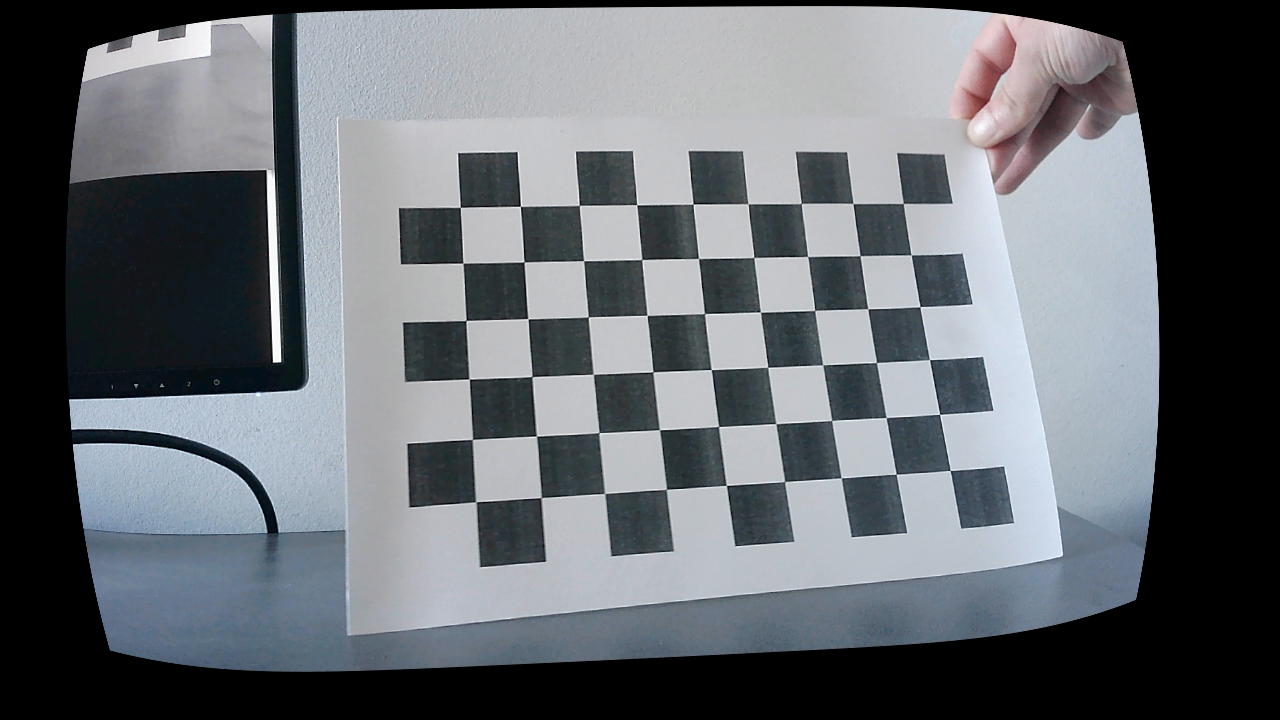

Supplement: Supplementary file 1 [file jimaging-12-00280-s001.zip › Supplementary Materials/first test/results/OutAw_runs/rectified_left/pair_0030_left.png]

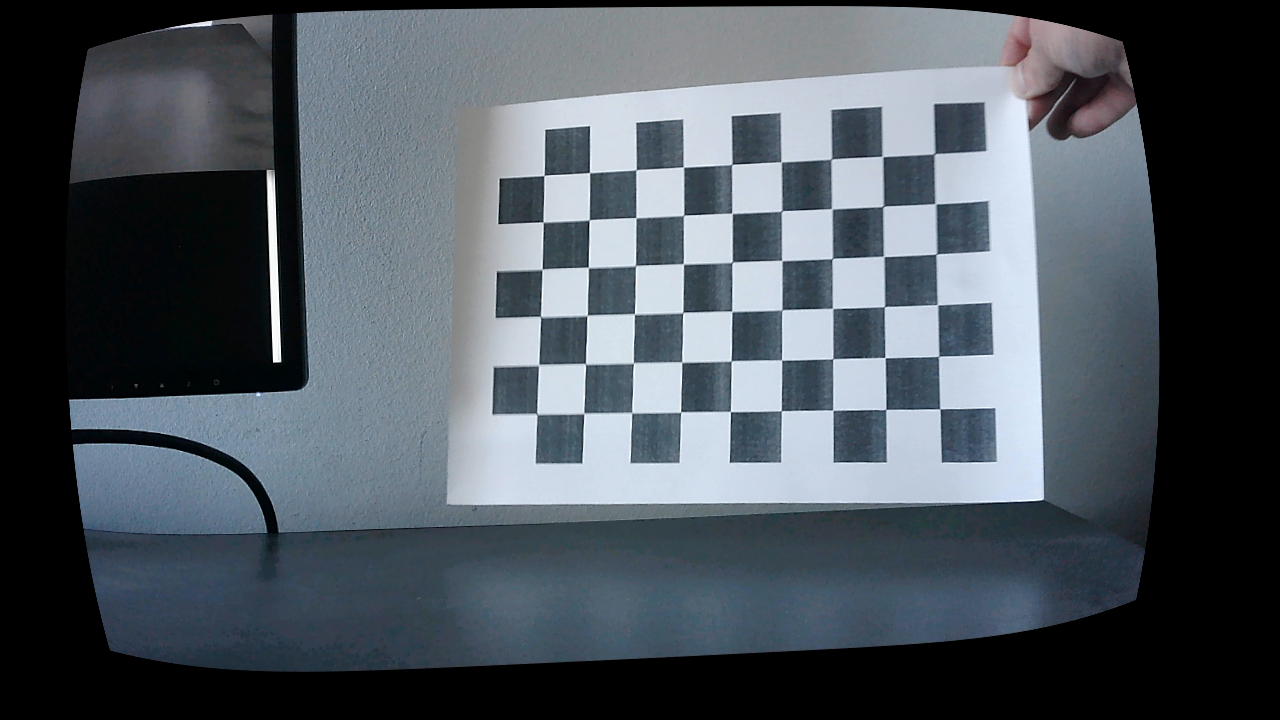

Supplement: Supplementary file 1 [file jimaging-12-00280-s001.zip › Supplementary Materials/first test/results/OutAw_runs/rectified_left/pair_0045_left.png]

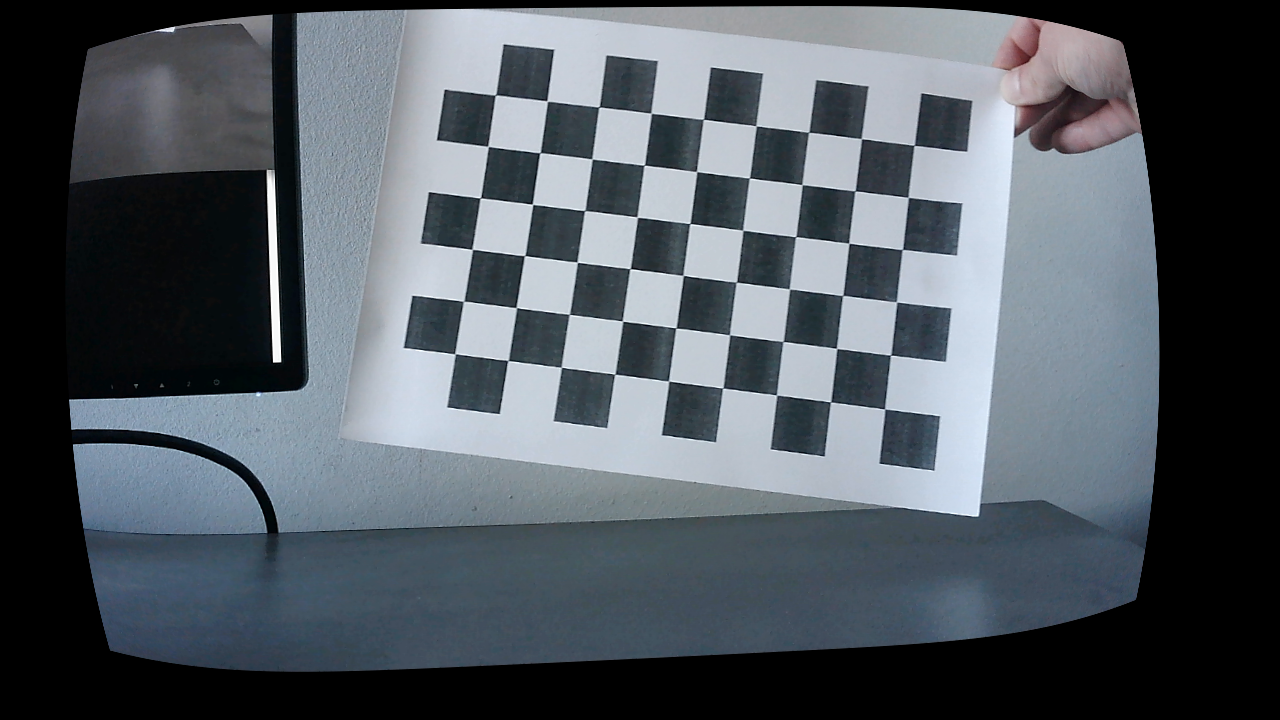

Supplement: Supplementary file 1 [file jimaging-12-00280-s001.zip › Supplementary Materials/first test/results/OutAw_runs/rectified_left/pair_0048_left.png]

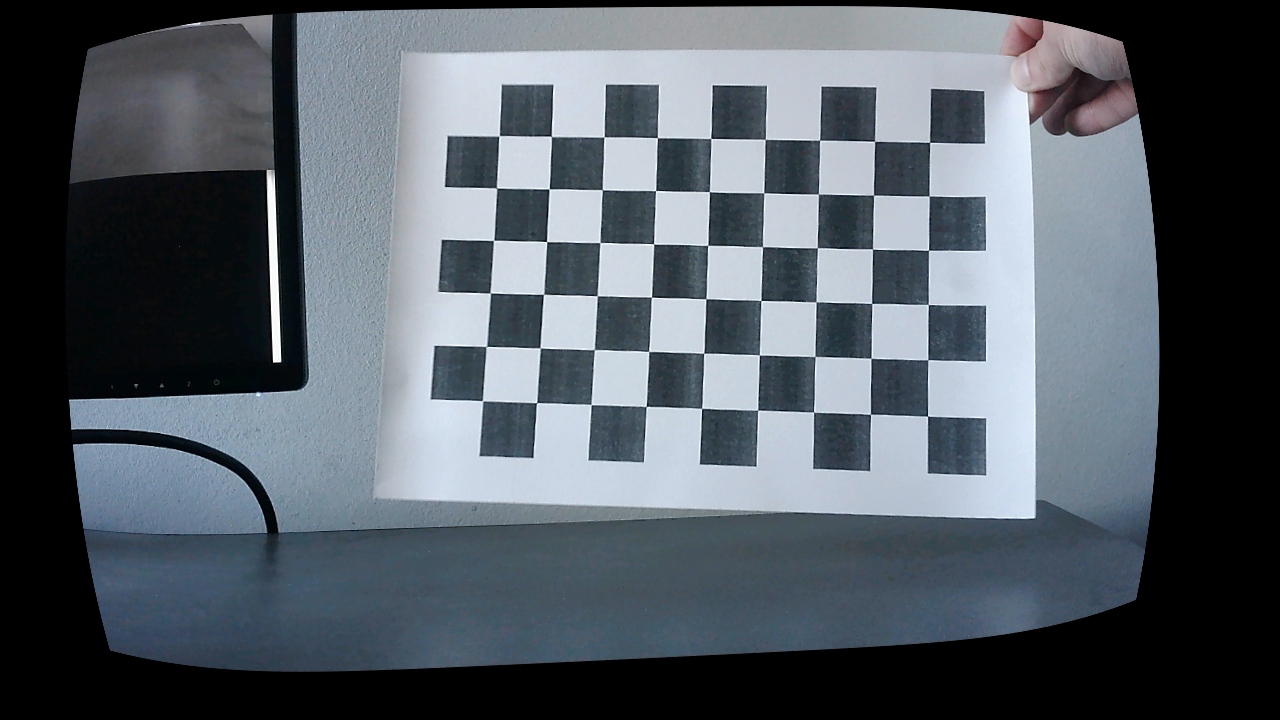

Supplement: Supplementary file 1 [file jimaging-12-00280-s001.zip › Supplementary Materials/first test/results/OutAw_runs/rectified_left/pair_0050_left.png]

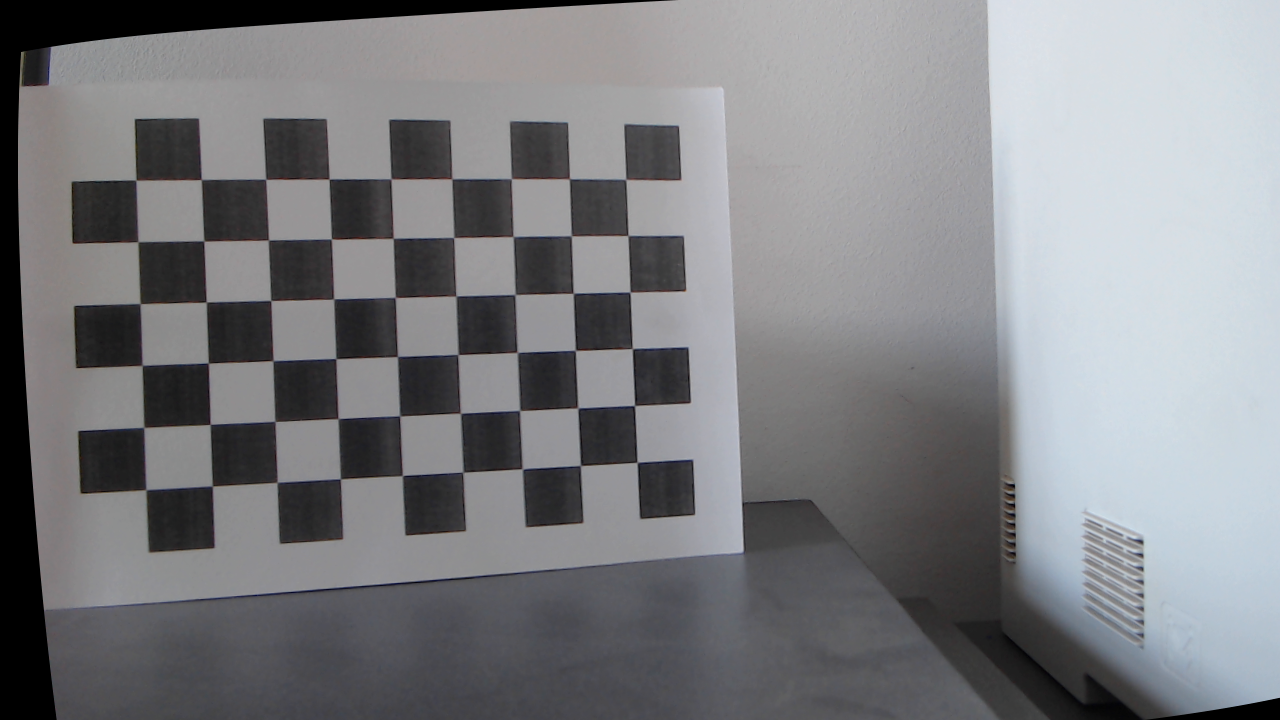

Supplement: Supplementary file 1 [file jimaging-12-00280-s001.zip › Supplementary Materials/first test/results/OutAw_runs/rectified_right/pair_0004_right.png]

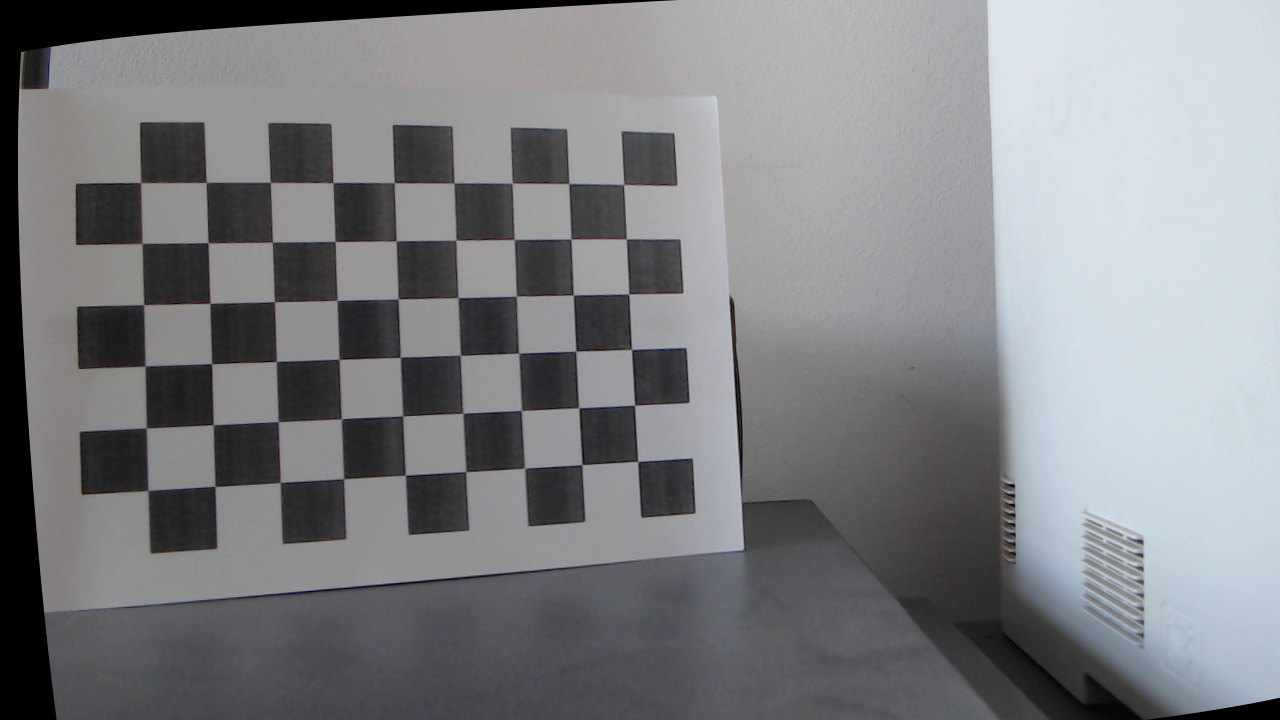

Supplement: Supplementary file 1 [file jimaging-12-00280-s001.zip › Supplementary Materials/first test/results/OutAw_runs/rectified_right/pair_0007_right.png]

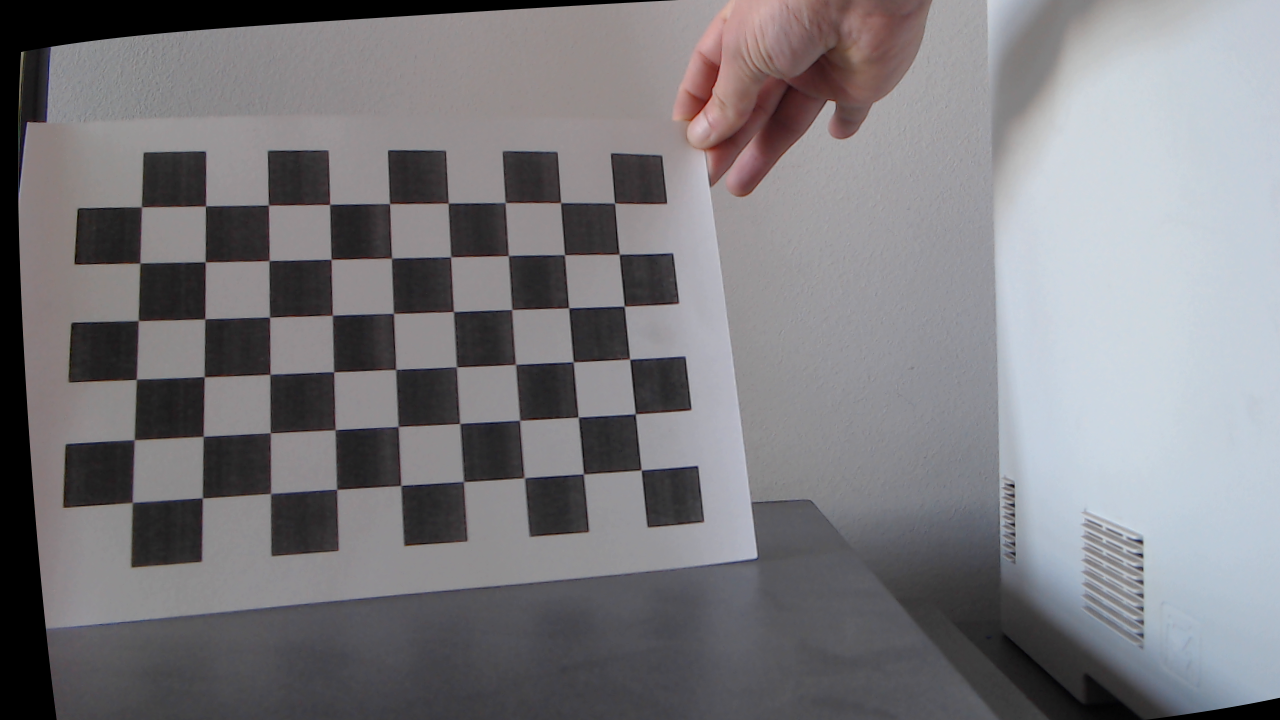

Supplement: Supplementary file 1 [file jimaging-12-00280-s001.zip › Supplementary Materials/first test/results/OutAw_runs/rectified_right/pair_0030_right.png]

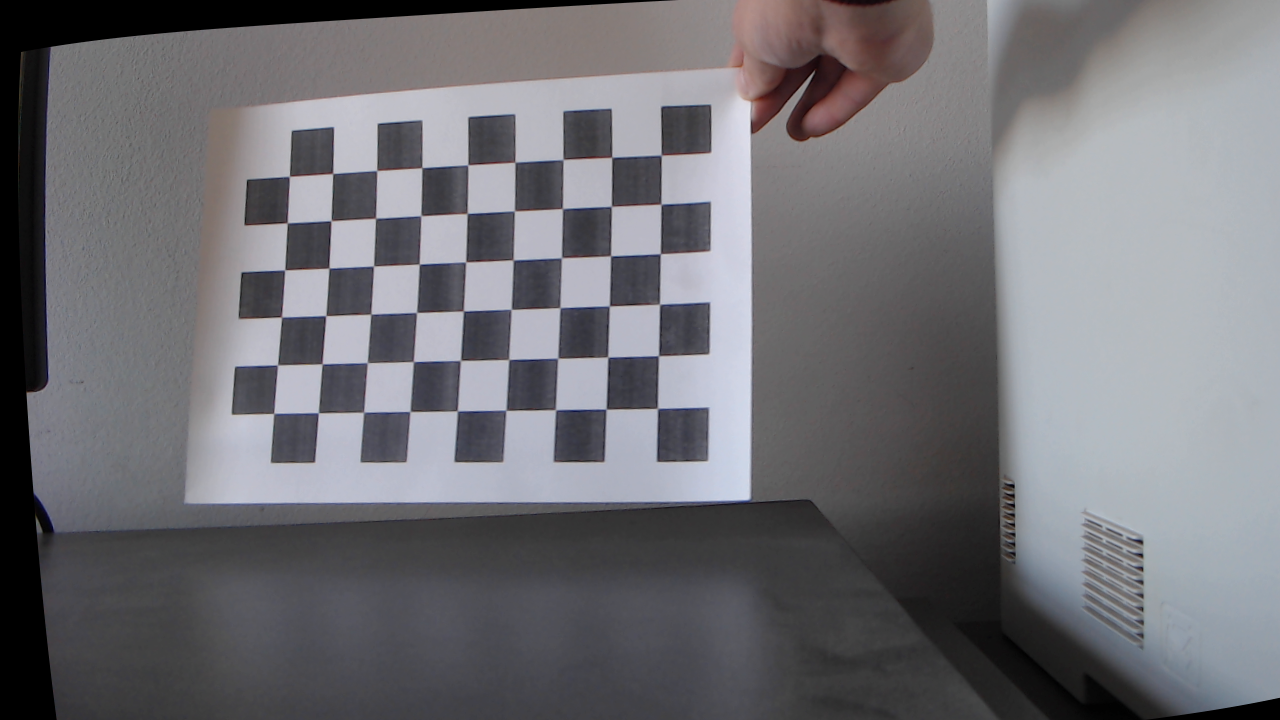

Supplement: Supplementary file 1 [file jimaging-12-00280-s001.zip › Supplementary Materials/first test/results/OutAw_runs/rectified_right/pair_0045_right.png]

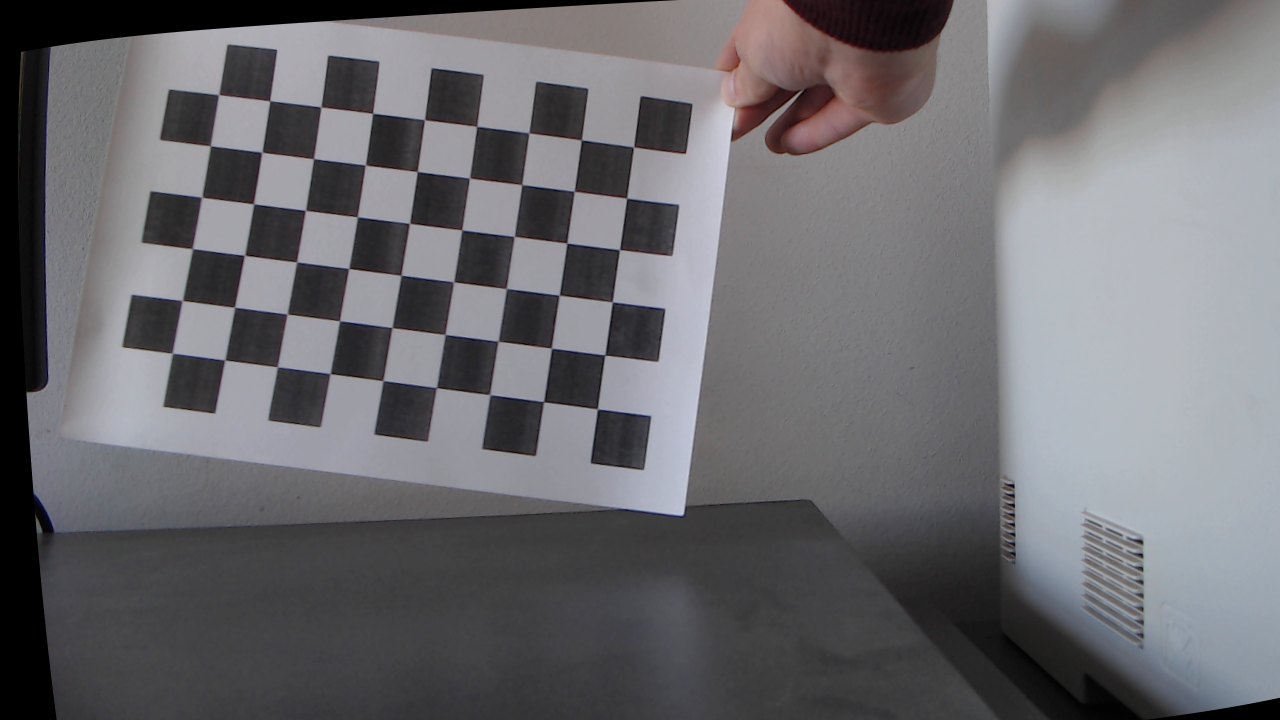

Supplement: Supplementary file 1 [file jimaging-12-00280-s001.zip › Supplementary Materials/first test/results/OutAw_runs/rectified_right/pair_0048_right.png]

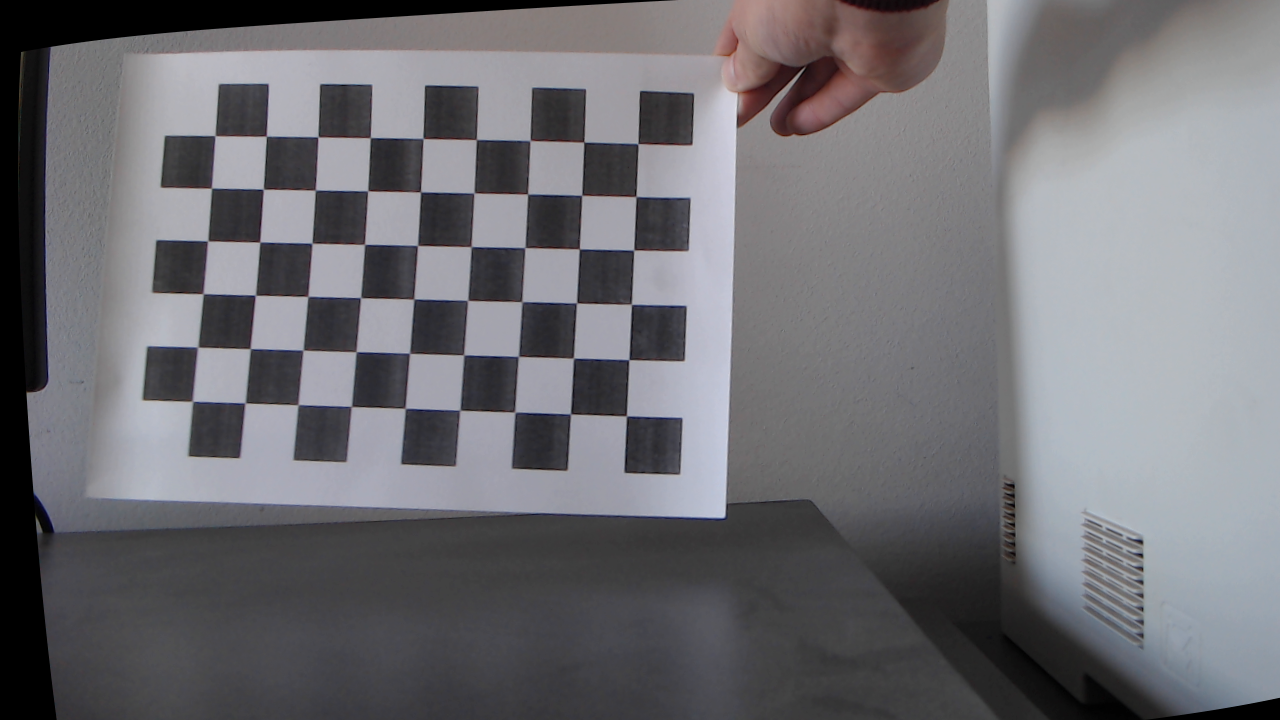

Supplement: Supplementary file 1 [file jimaging-12-00280-s001.zip › Supplementary Materials/first test/results/OutAw_runs/rectified_right/pair_0050_right.png]

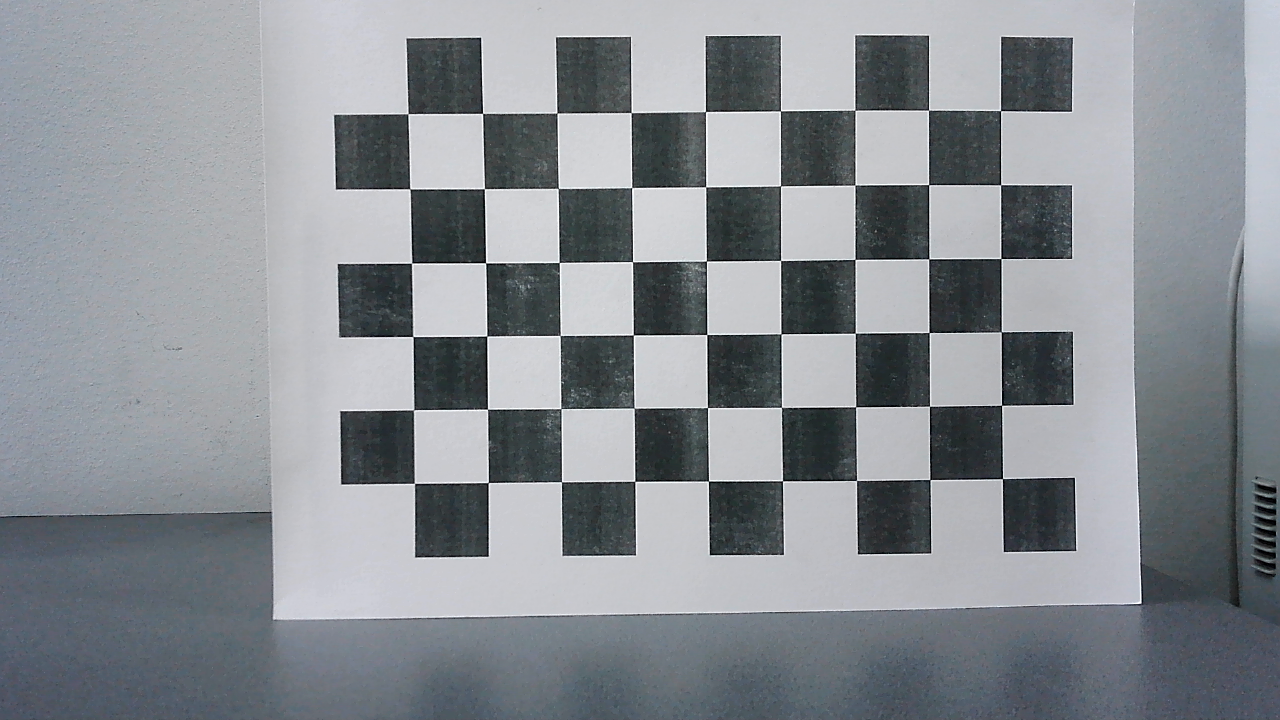

Supplement: Supplementary file 1 [file jimaging-12-00280-s001.zip › Supplementary Materials/second test/data/raw_selection/left/pair_0000_left.png]

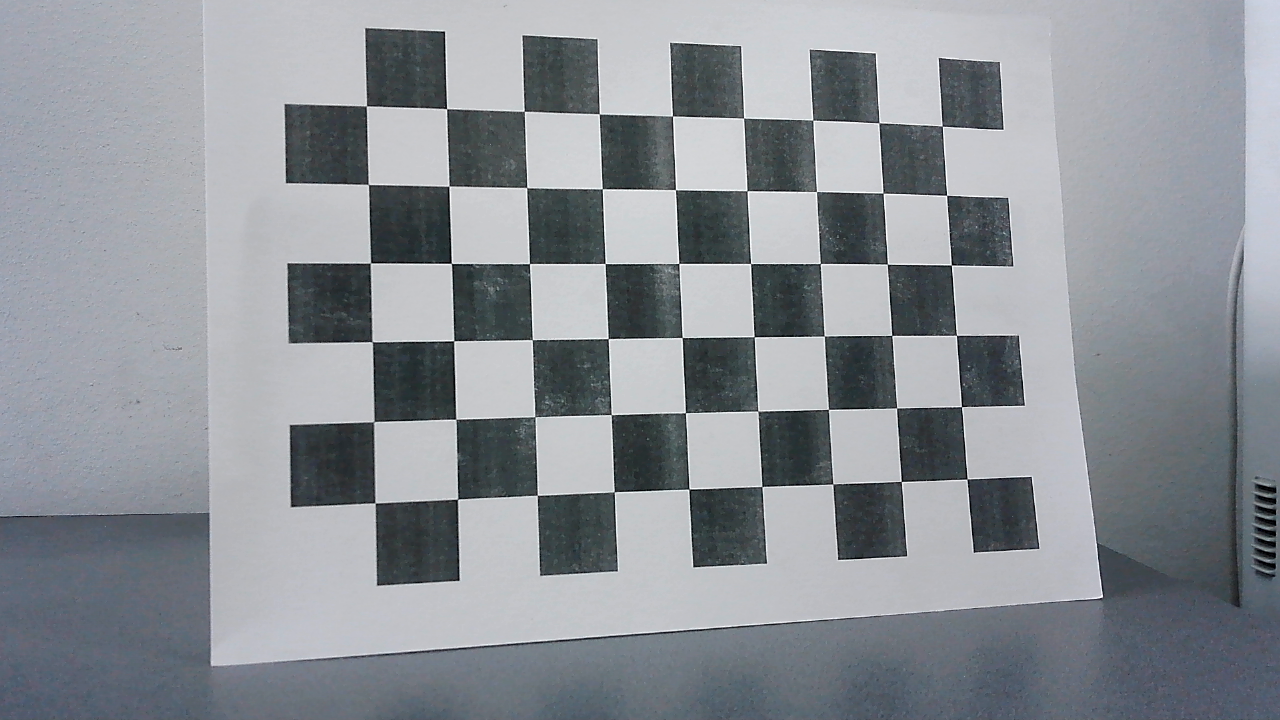

Supplement: Supplementary file 1 [file jimaging-12-00280-s001.zip › Supplementary Materials/second test/data/raw_selection/left/pair_0001_left.png]

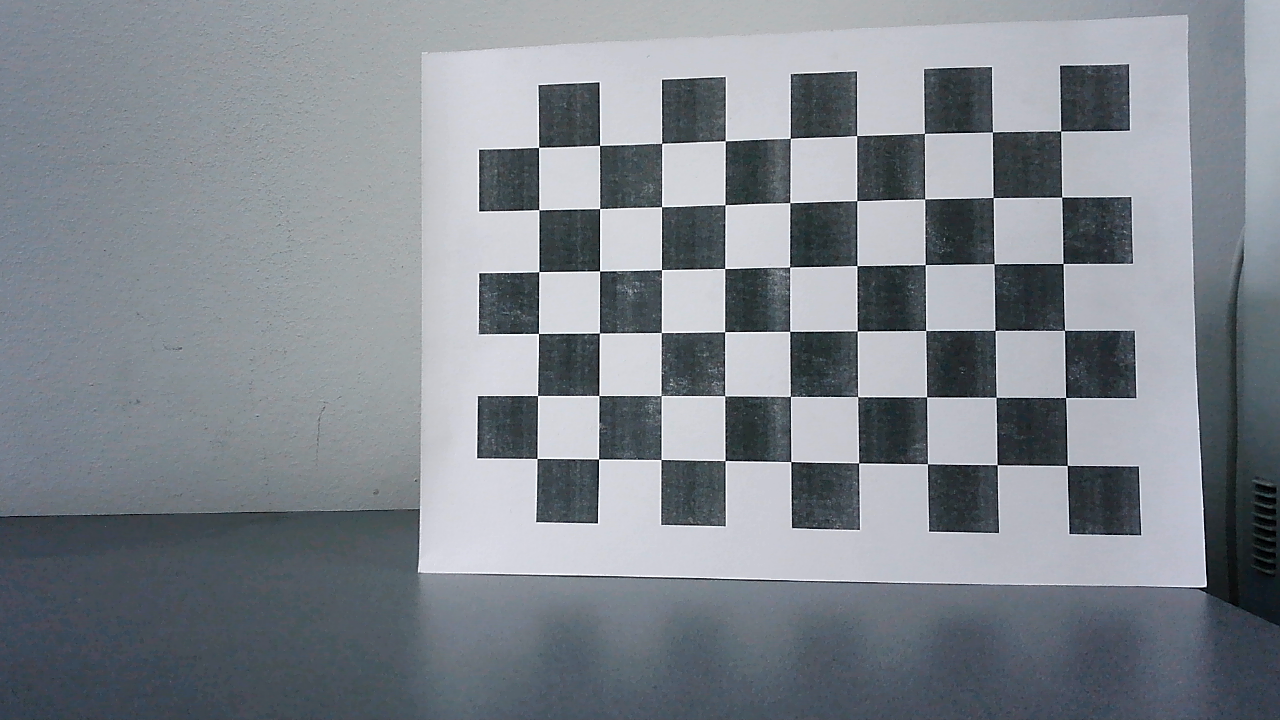

Supplement: Supplementary file 1 [file jimaging-12-00280-s001.zip › Supplementary Materials/second test/data/raw_selection/left/pair_0002_left.png]

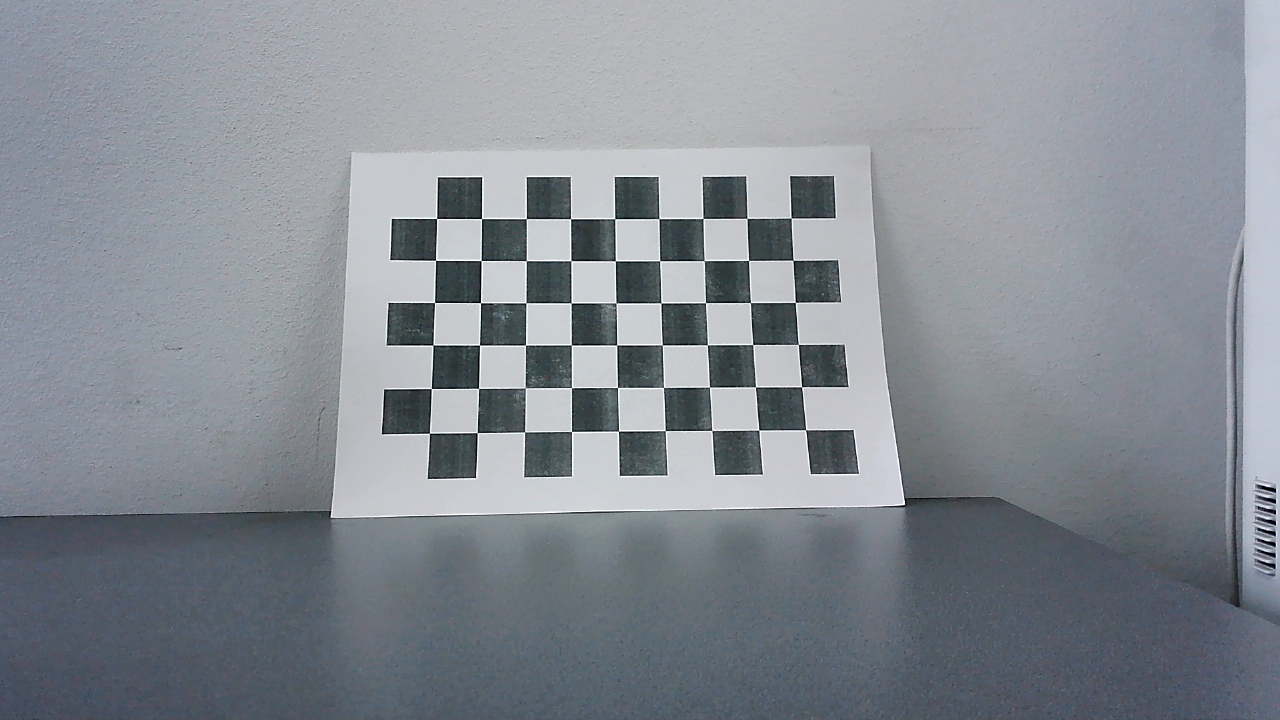

Supplement: Supplementary file 1 [file jimaging-12-00280-s001.zip › Supplementary Materials/second test/data/raw_selection/left/pair_0003_left.png]

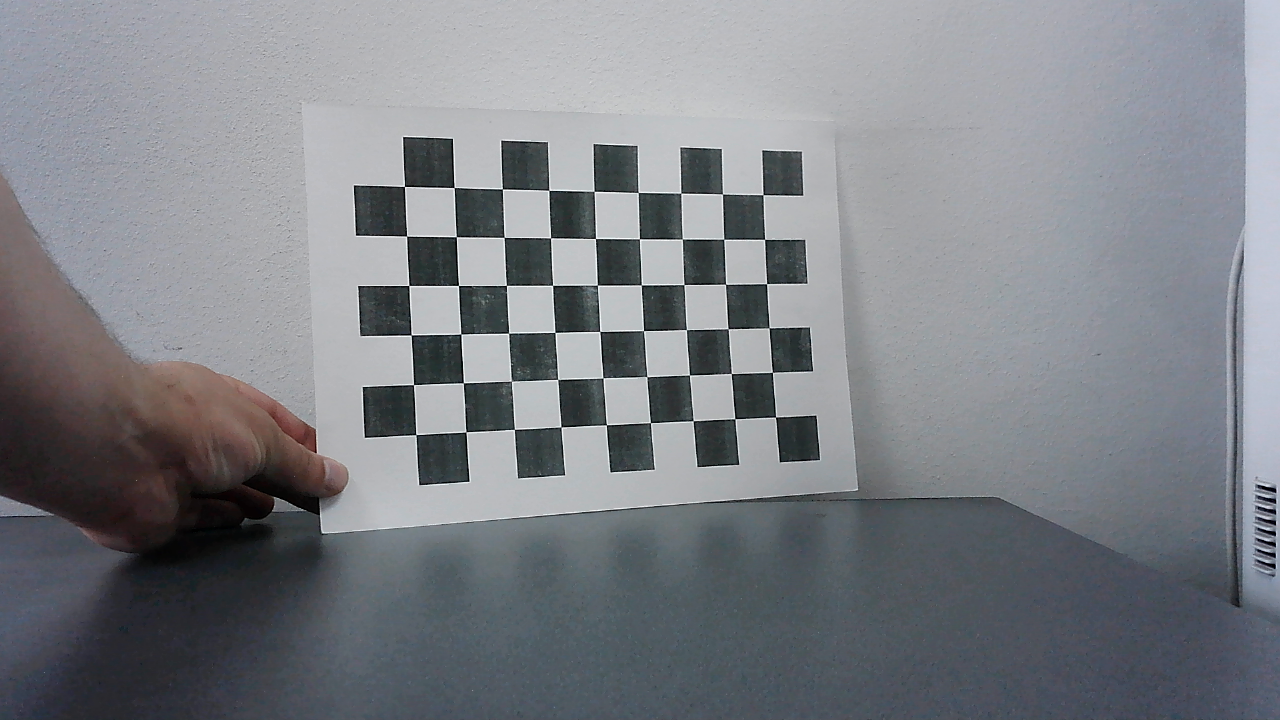

Supplement: Supplementary file 1 [file jimaging-12-00280-s001.zip › Supplementary Materials/second test/data/raw_selection/left/pair_0004_left.png]

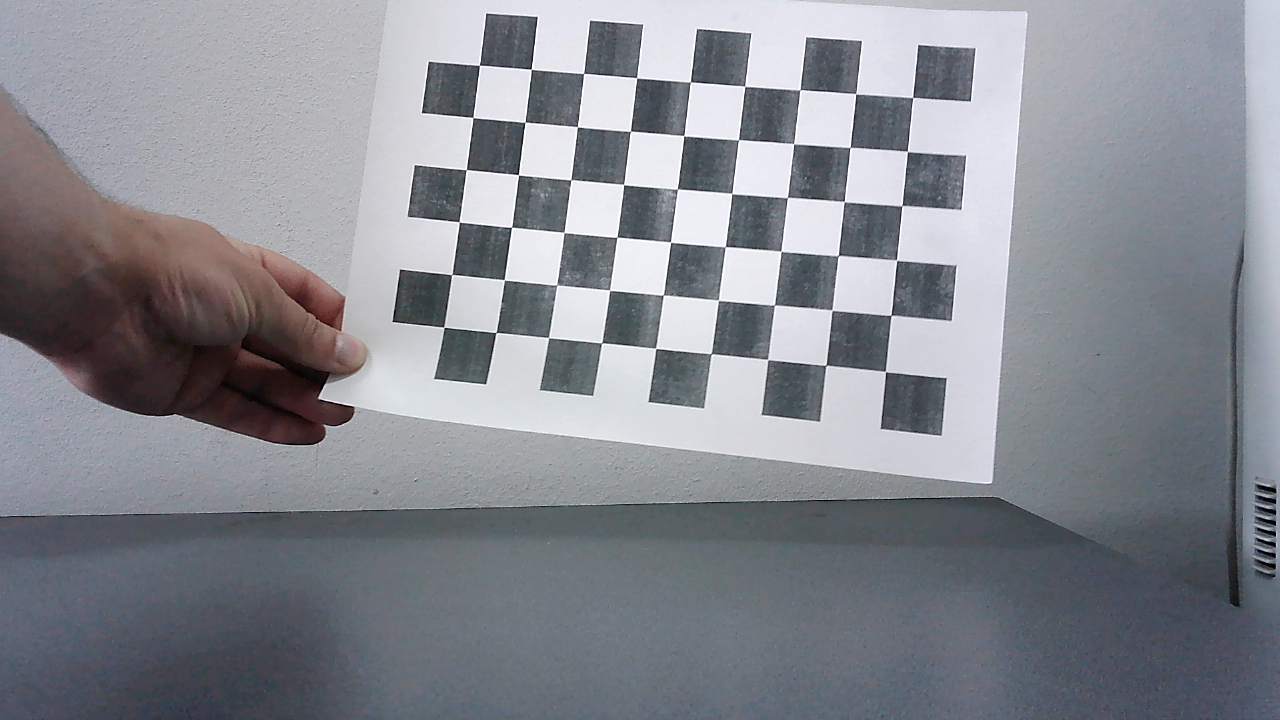

Supplement: Supplementary file 1 [file jimaging-12-00280-s001.zip › Supplementary Materials/second test/data/raw_selection/left/pair_0005_left.png]

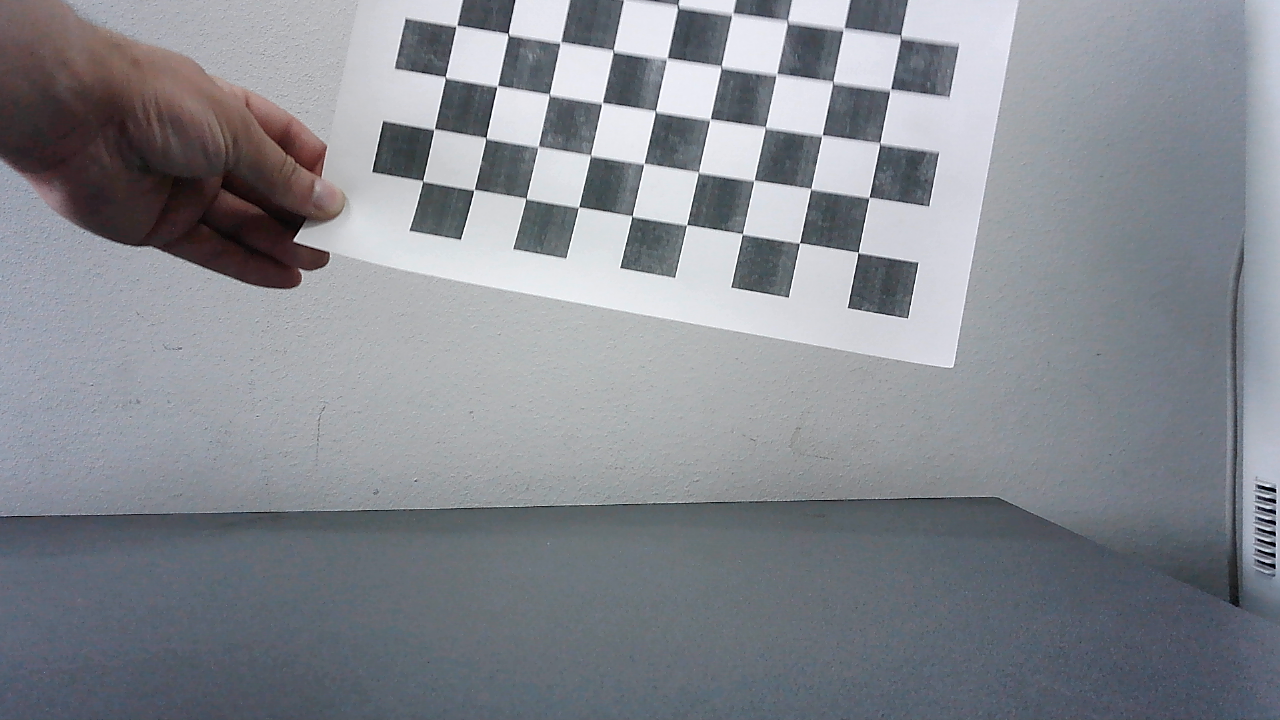

Supplement: Supplementary file 1 [file jimaging-12-00280-s001.zip › Supplementary Materials/second test/data/raw_selection/left/pair_0006_left.png]

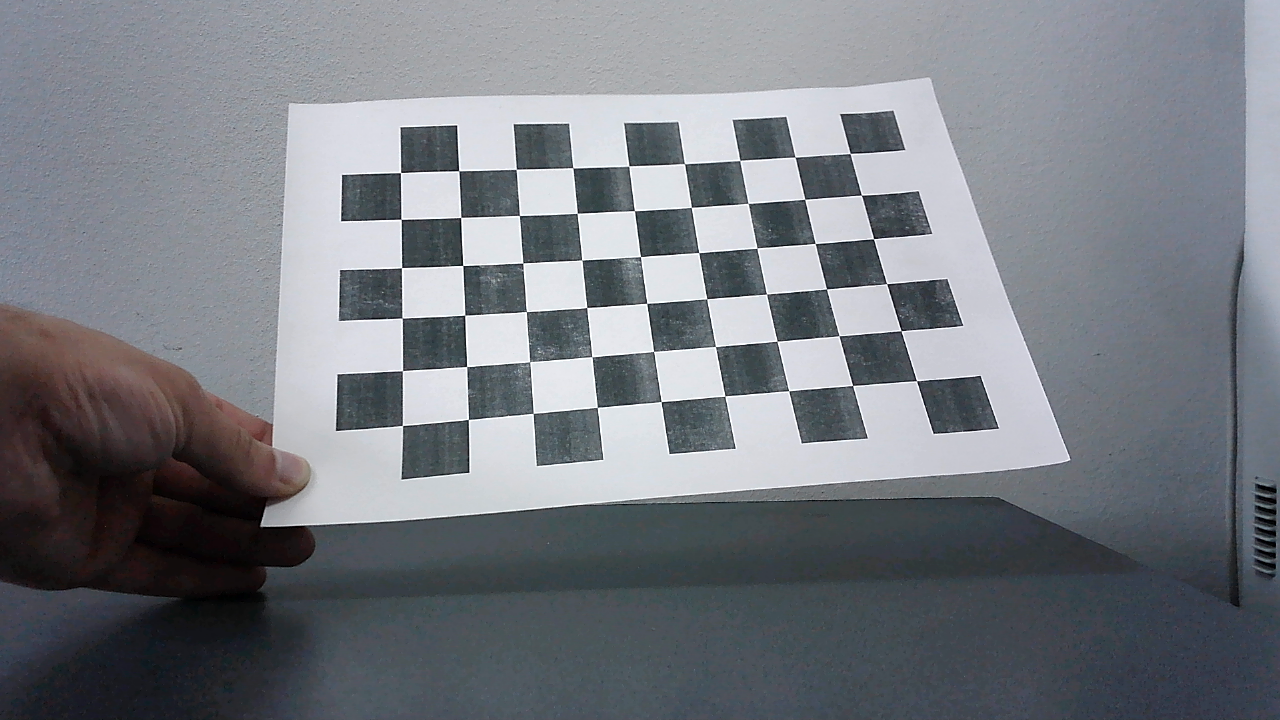

Supplement: Supplementary file 1 [file jimaging-12-00280-s001.zip › Supplementary Materials/second test/data/raw_selection/left/pair_0007_left.png]

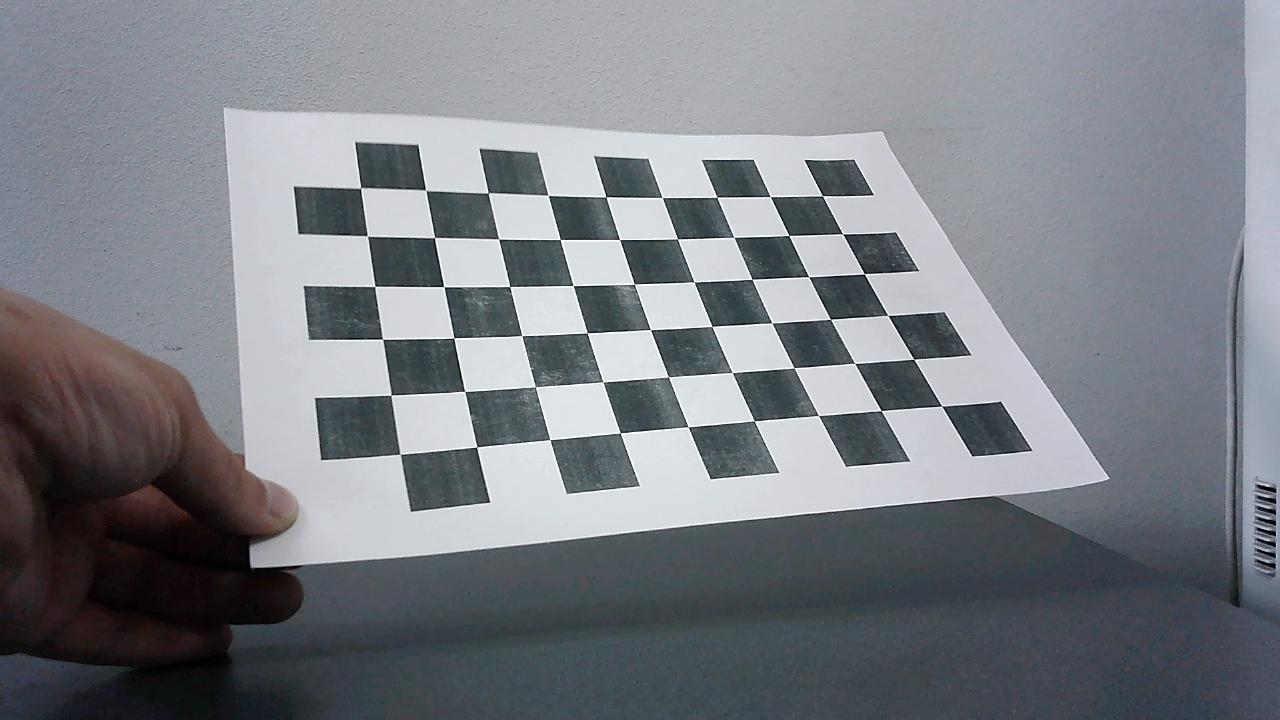

Supplement: Supplementary file 1 [file jimaging-12-00280-s001.zip › Supplementary Materials/second test/data/raw_selection/left/pair_0008_left.png]

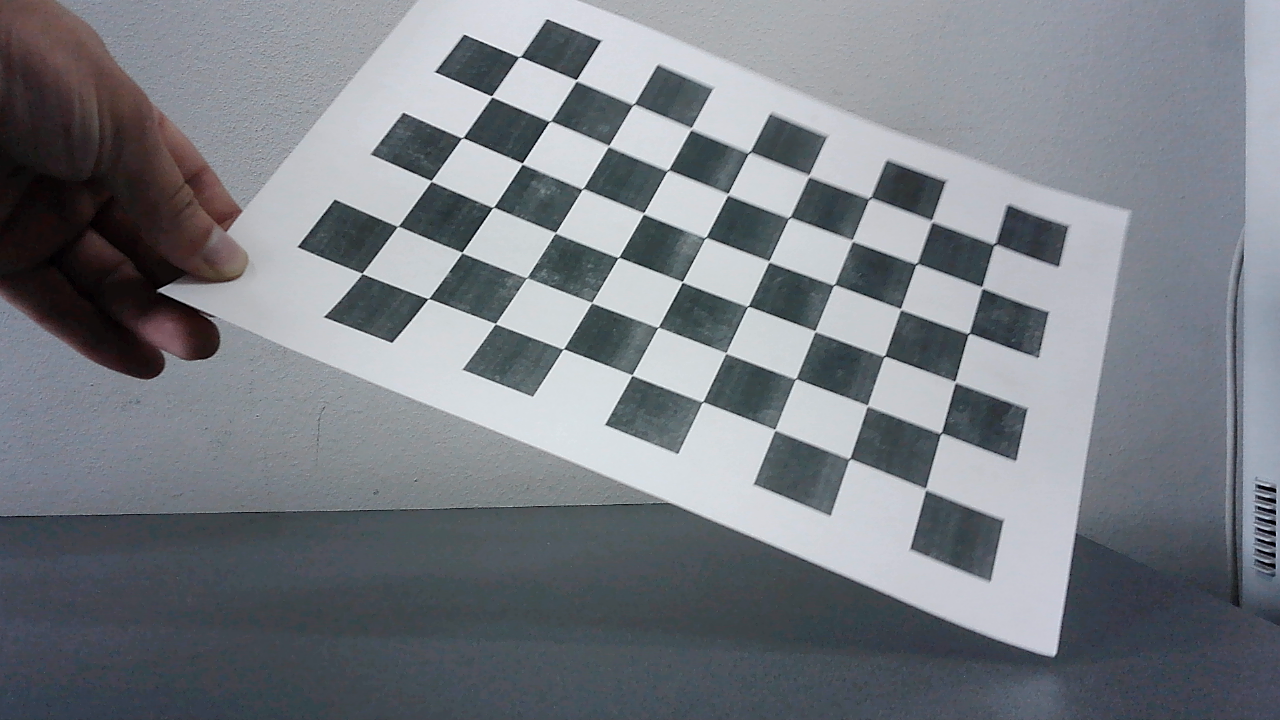

Supplement: Supplementary file 1 [file jimaging-12-00280-s001.zip › Supplementary Materials/second test/data/raw_selection/left/pair_0009_left.png]

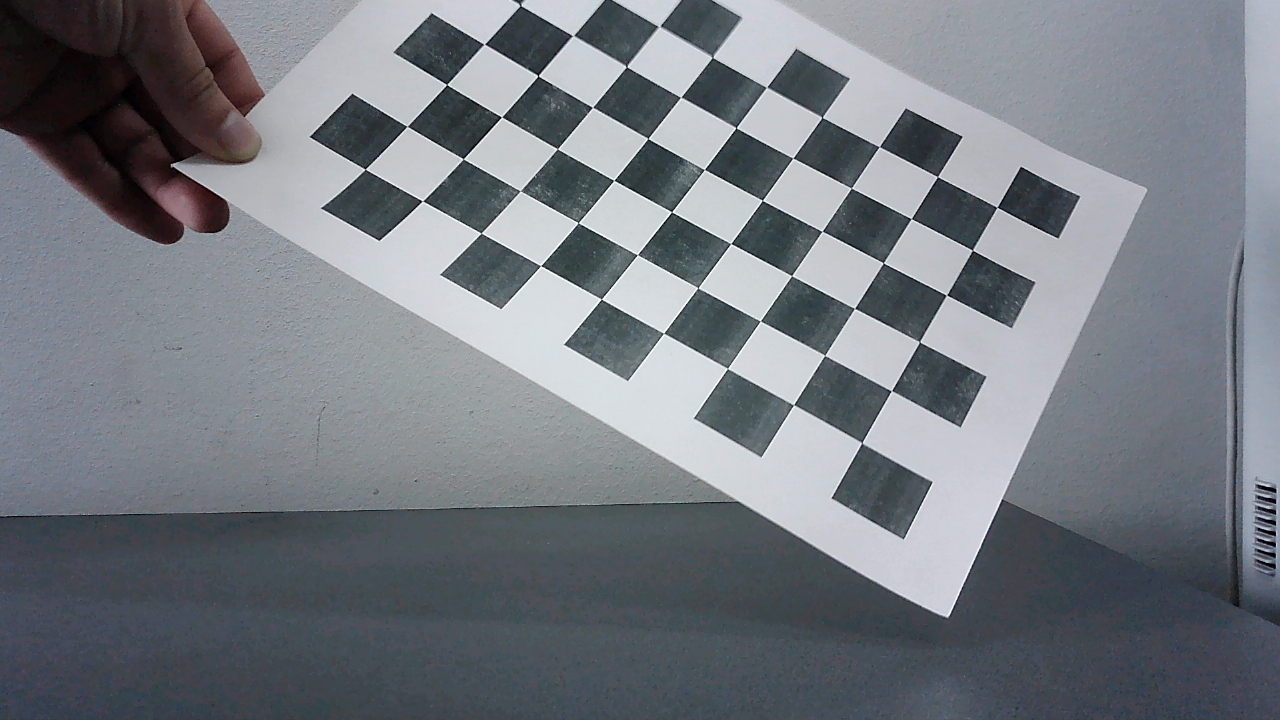

Supplement: Supplementary file 1 [file jimaging-12-00280-s001.zip › Supplementary Materials/second test/data/raw_selection/left/pair_0010_left.png]
