# Supplementary figures and images for: Quantifying and Improving Stereo Camera Calibration Robustness: An Outlier-Aware Algorithm for Digital Twin Data Acquisition (part 1 of 2)
Source: J Imaging. 2026 Jun 25;12(7):280. doi: 10.3390/jimaging12070280 (PMC13413186; doi:10.3390/jimaging12070280)

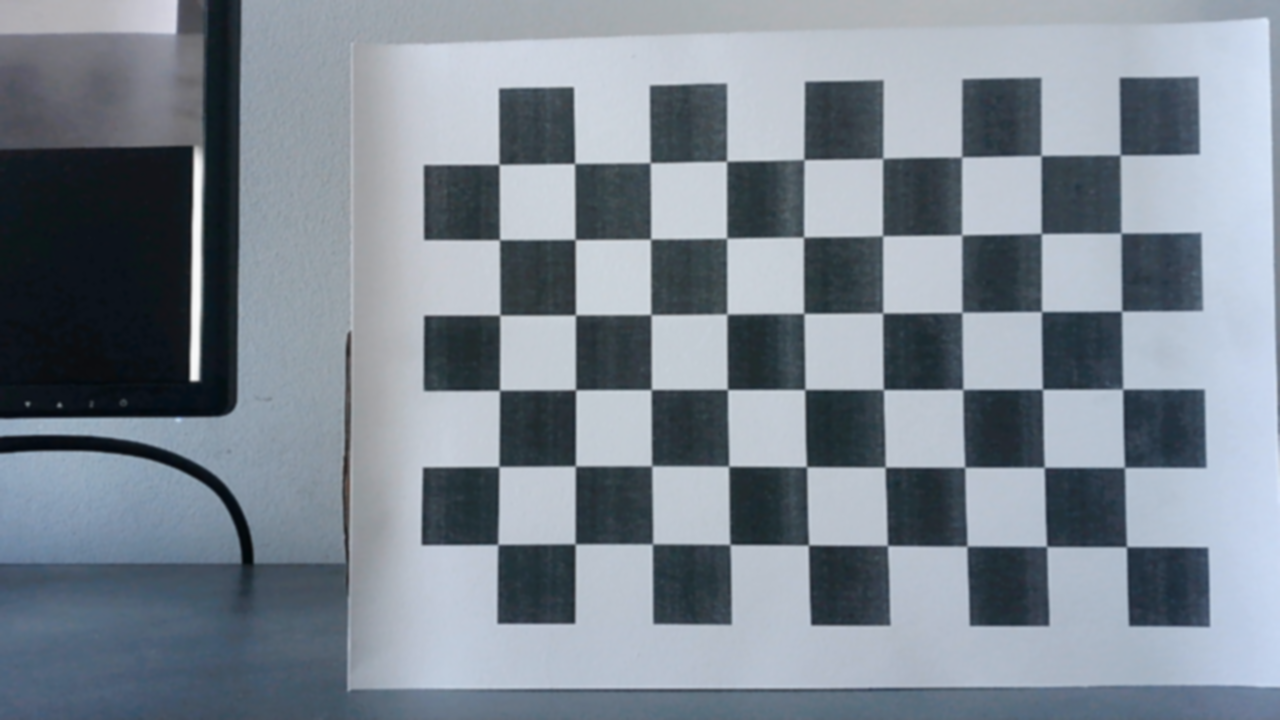

Supplement: Supplementary file 1 [file jimaging-12-00280-s001.zip › Supplementary Materials/first test/Pairs/corrupted/left/pair_0000_left.png]

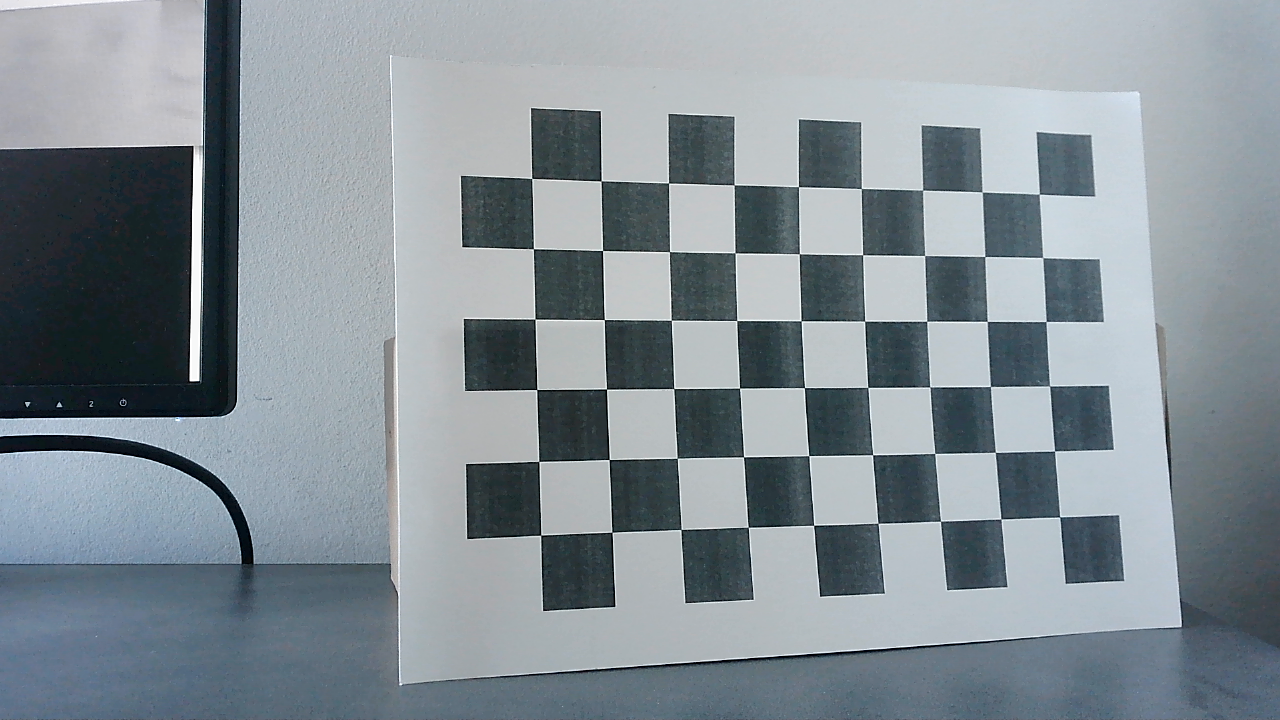

Supplement: Supplementary file 1 [file jimaging-12-00280-s001.zip › Supplementary Materials/first test/Pairs/corrupted/left/pair_0001_left.png]

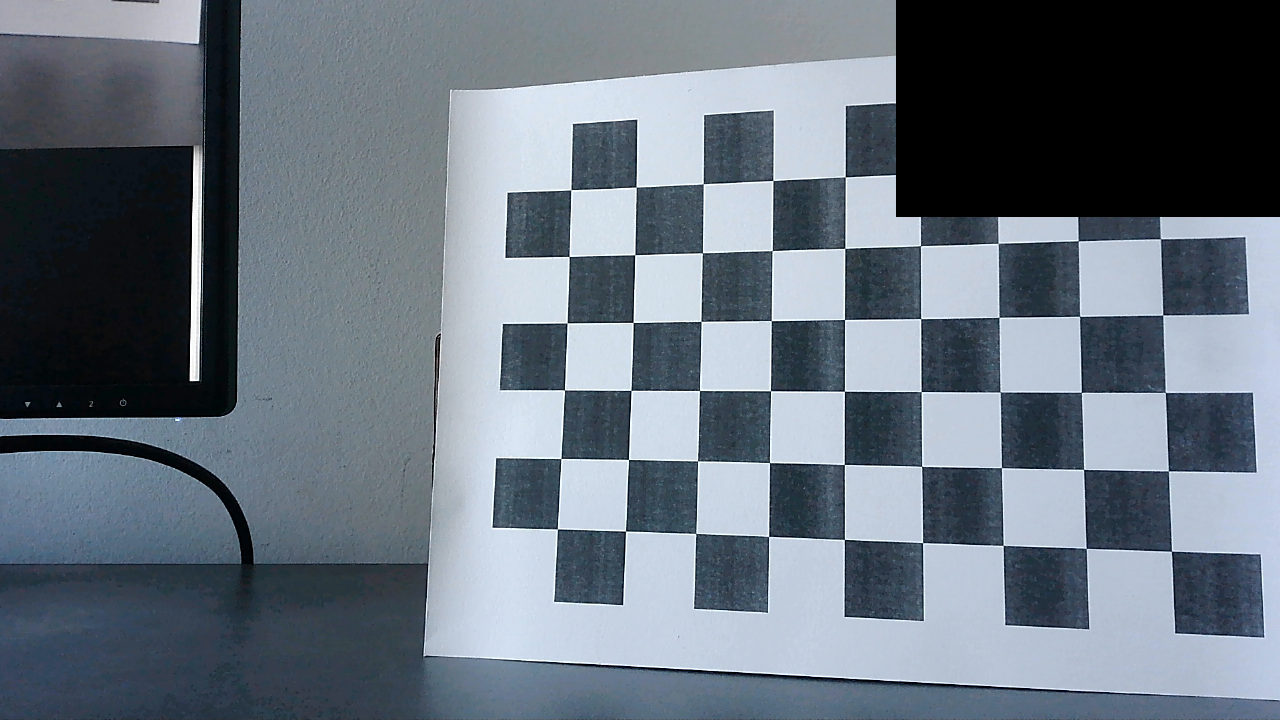

Supplement: Supplementary file 1 [file jimaging-12-00280-s001.zip › Supplementary Materials/first test/Pairs/corrupted/left/pair_0002_left.png]

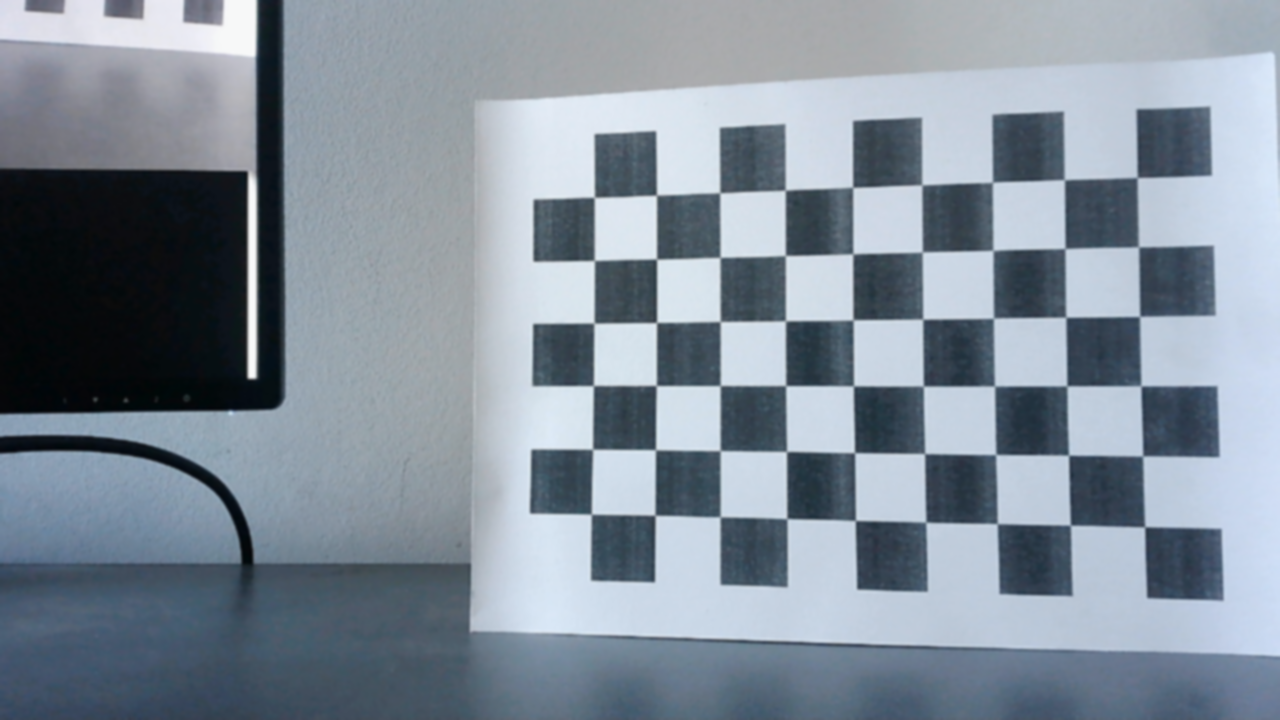

Supplement: Supplementary file 1 [file jimaging-12-00280-s001.zip › Supplementary Materials/first test/Pairs/corrupted/left/pair_0003_left.png]

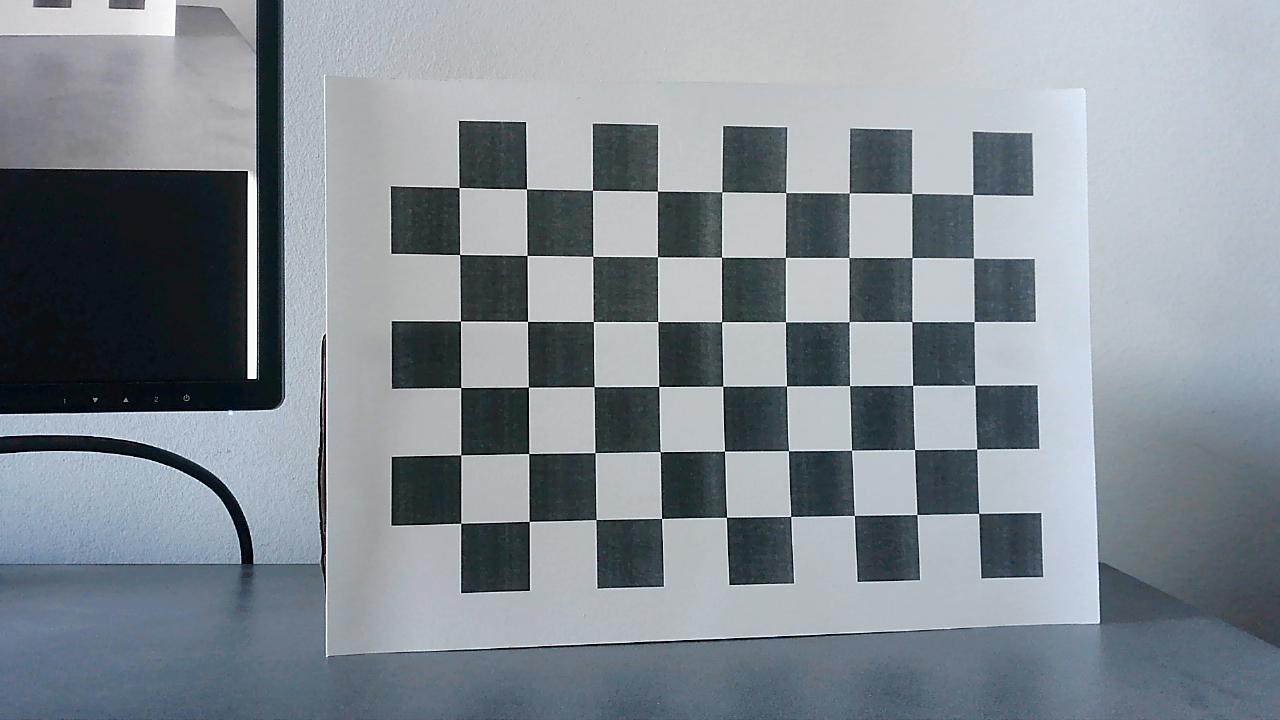

Supplement: Supplementary file 1 [file jimaging-12-00280-s001.zip › Supplementary Materials/first test/Pairs/corrupted/left/pair_0004_left.png]

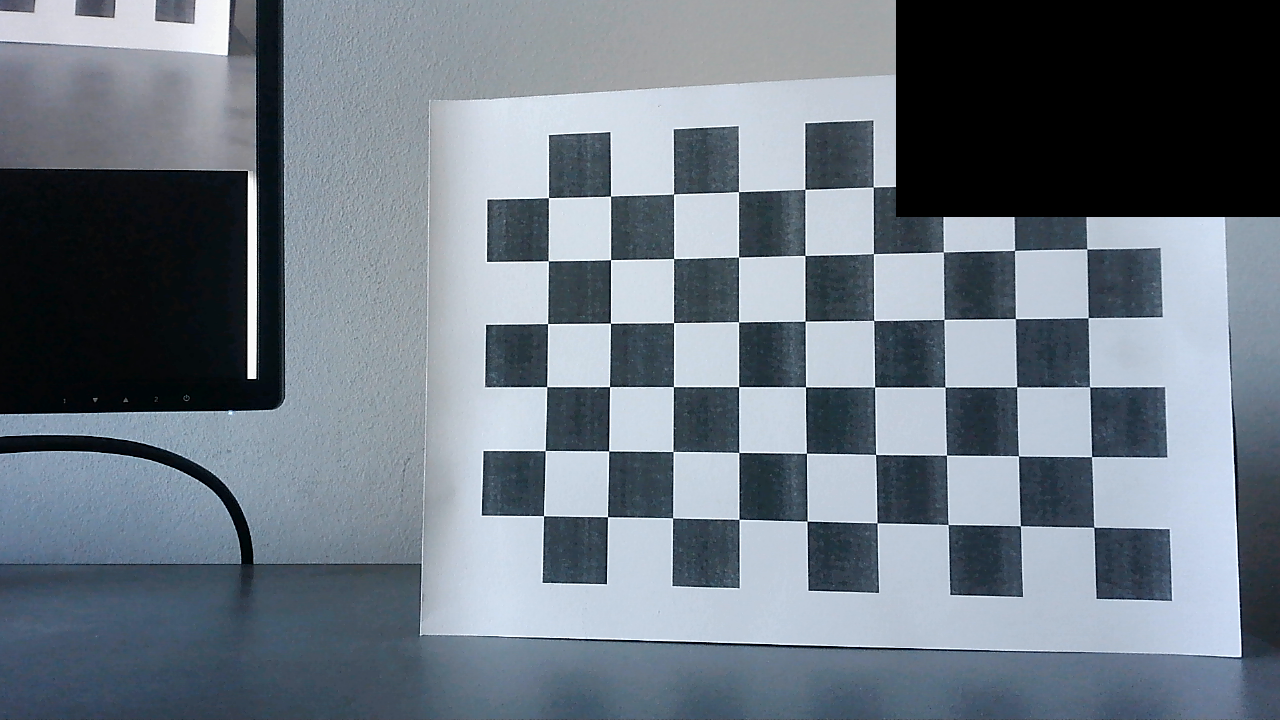

Supplement: Supplementary file 1 [file jimaging-12-00280-s001.zip › Supplementary Materials/first test/Pairs/corrupted/left/pair_0005_left.png]

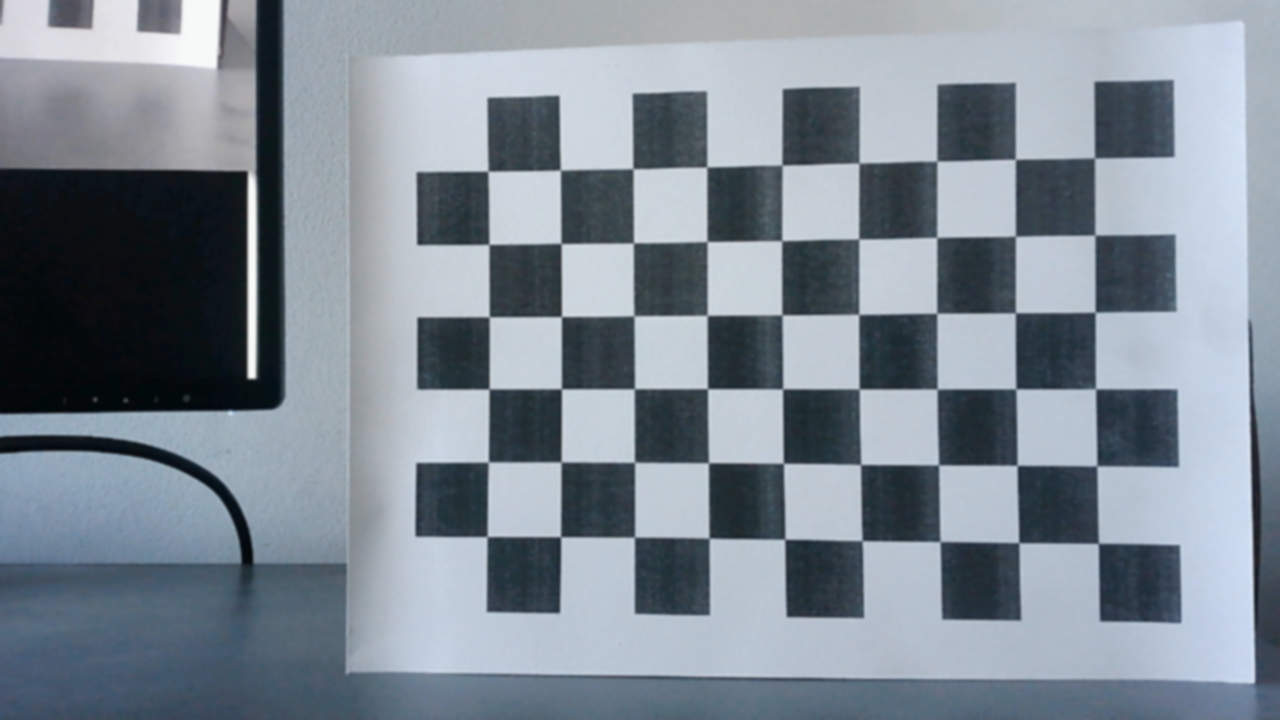

Supplement: Supplementary file 1 [file jimaging-12-00280-s001.zip › Supplementary Materials/first test/Pairs/corrupted/left/pair_0006_left.png]

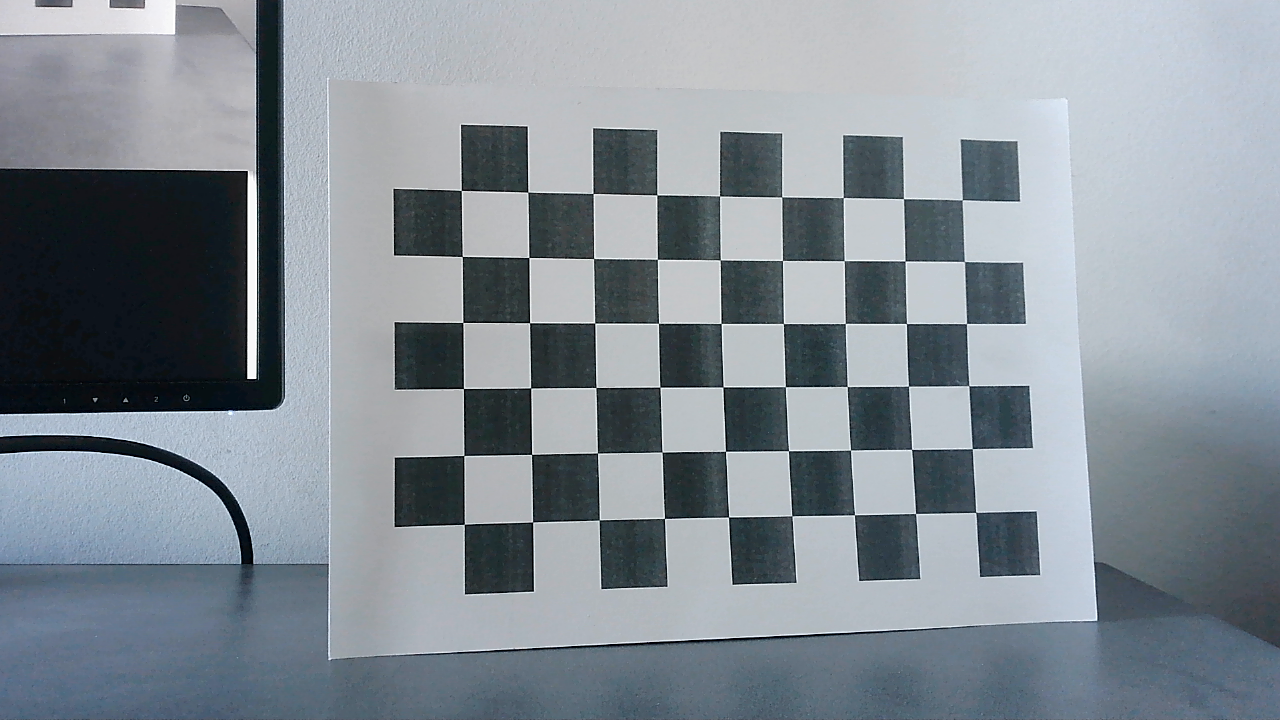

Supplement: Supplementary file 1 [file jimaging-12-00280-s001.zip › Supplementary Materials/first test/Pairs/corrupted/left/pair_0007_left.png]

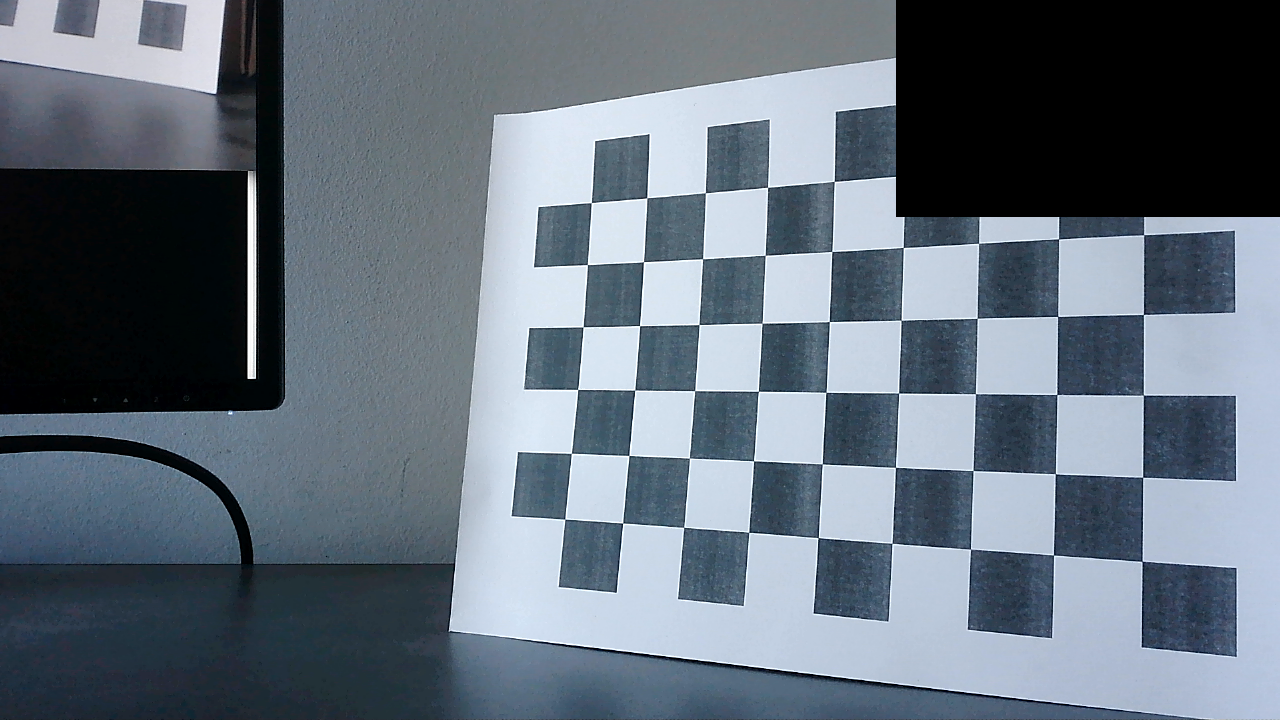

Supplement: Supplementary file 1 [file jimaging-12-00280-s001.zip › Supplementary Materials/first test/Pairs/corrupted/left/pair_0008_left.png]

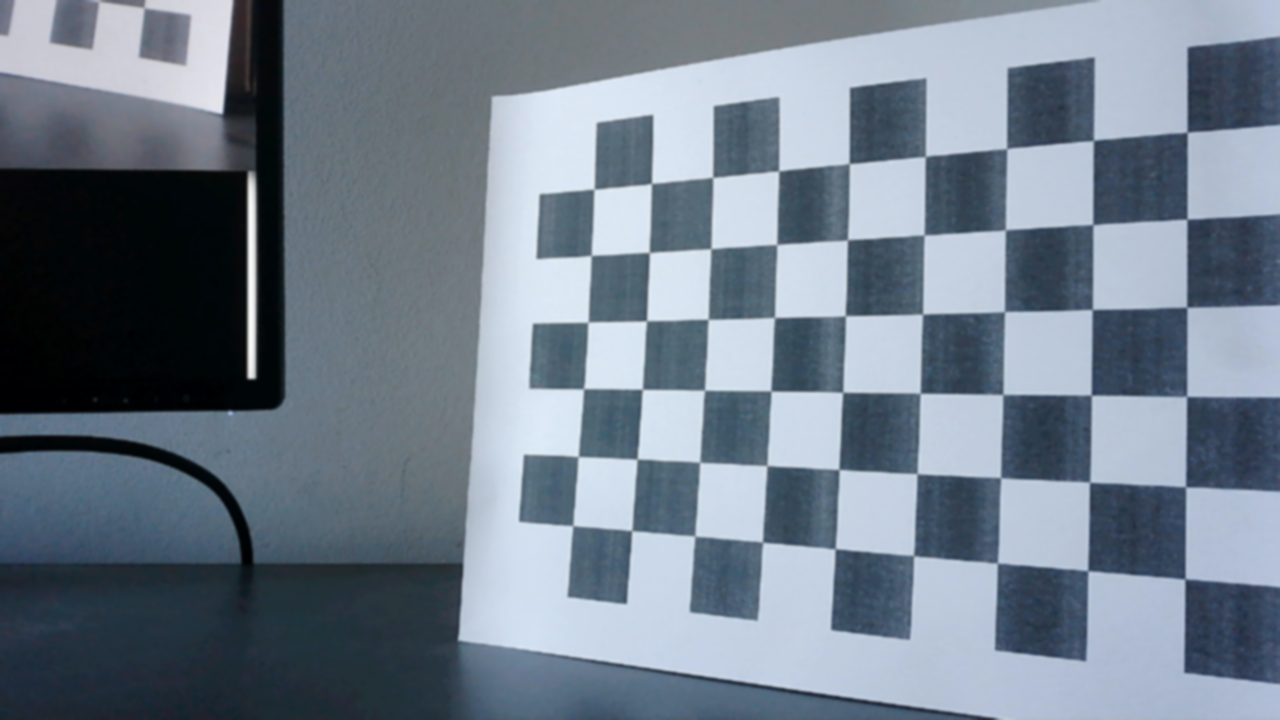

Supplement: Supplementary file 1 [file jimaging-12-00280-s001.zip › Supplementary Materials/first test/Pairs/corrupted/left/pair_0009_left.png]

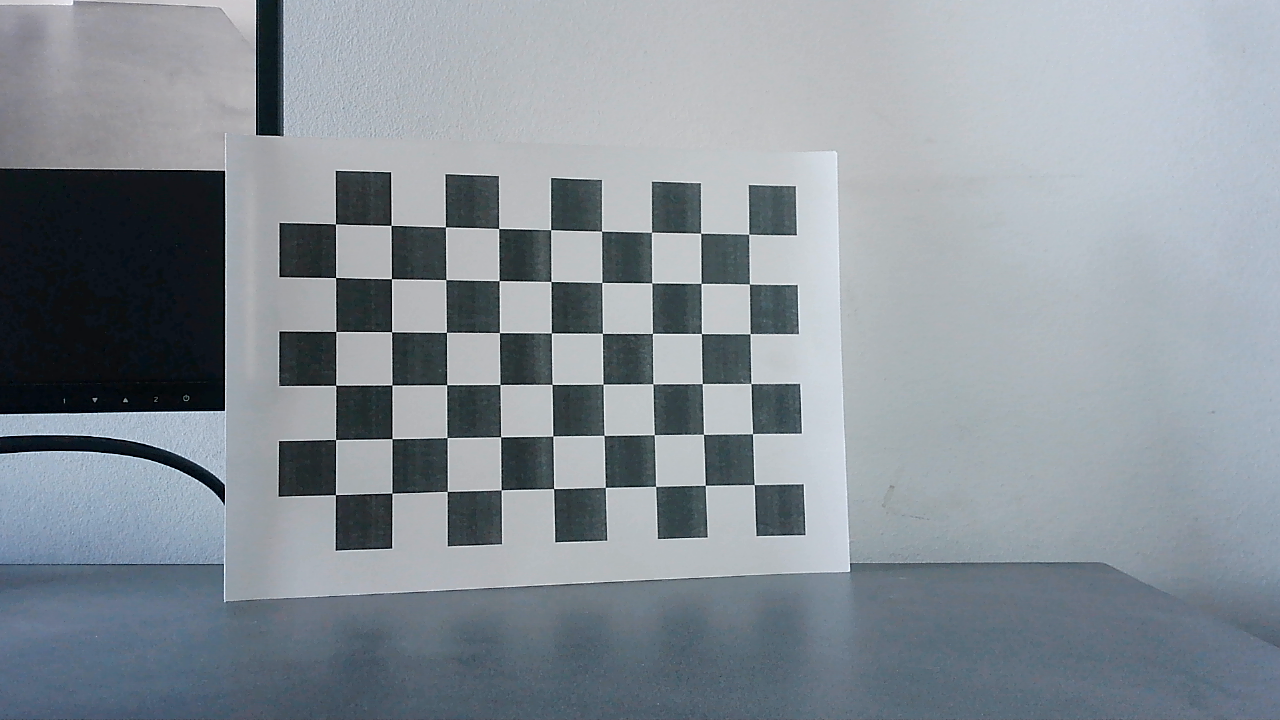

Supplement: Supplementary file 1 [file jimaging-12-00280-s001.zip › Supplementary Materials/first test/Pairs/corrupted/left/pair_0010_left.png]

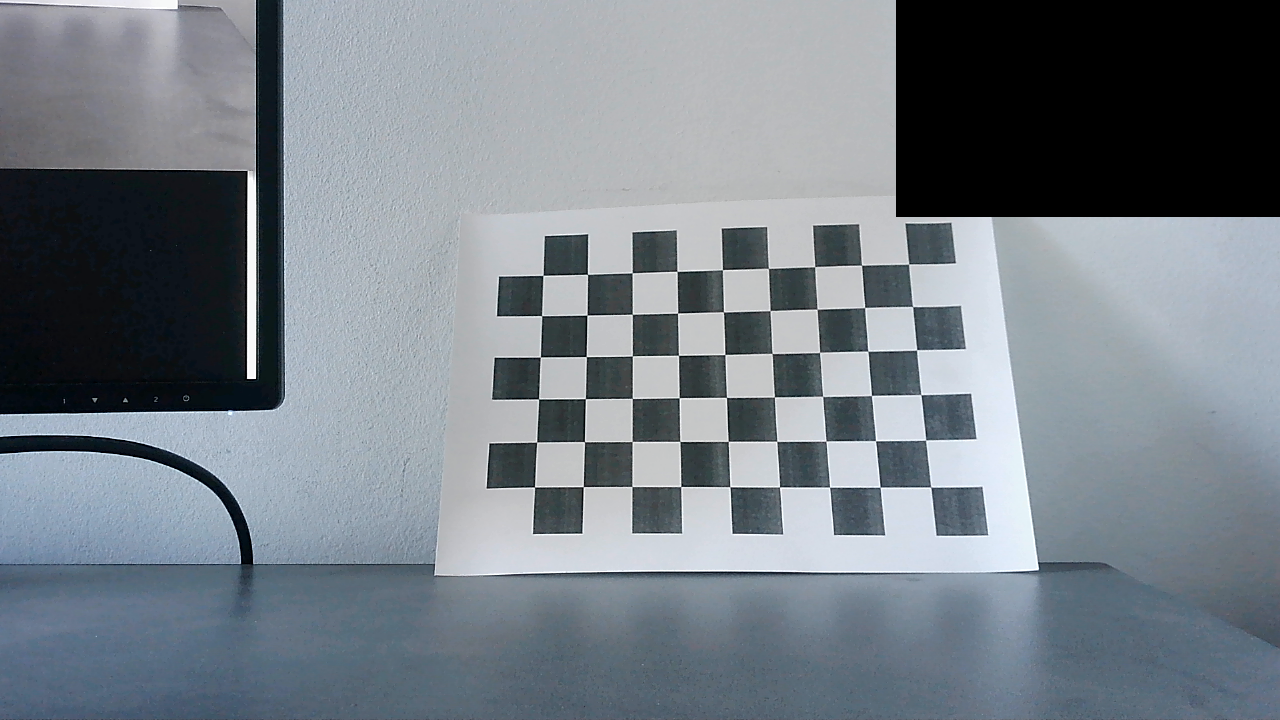

Supplement: Supplementary file 1 [file jimaging-12-00280-s001.zip › Supplementary Materials/first test/Pairs/corrupted/left/pair_0011_left.png]

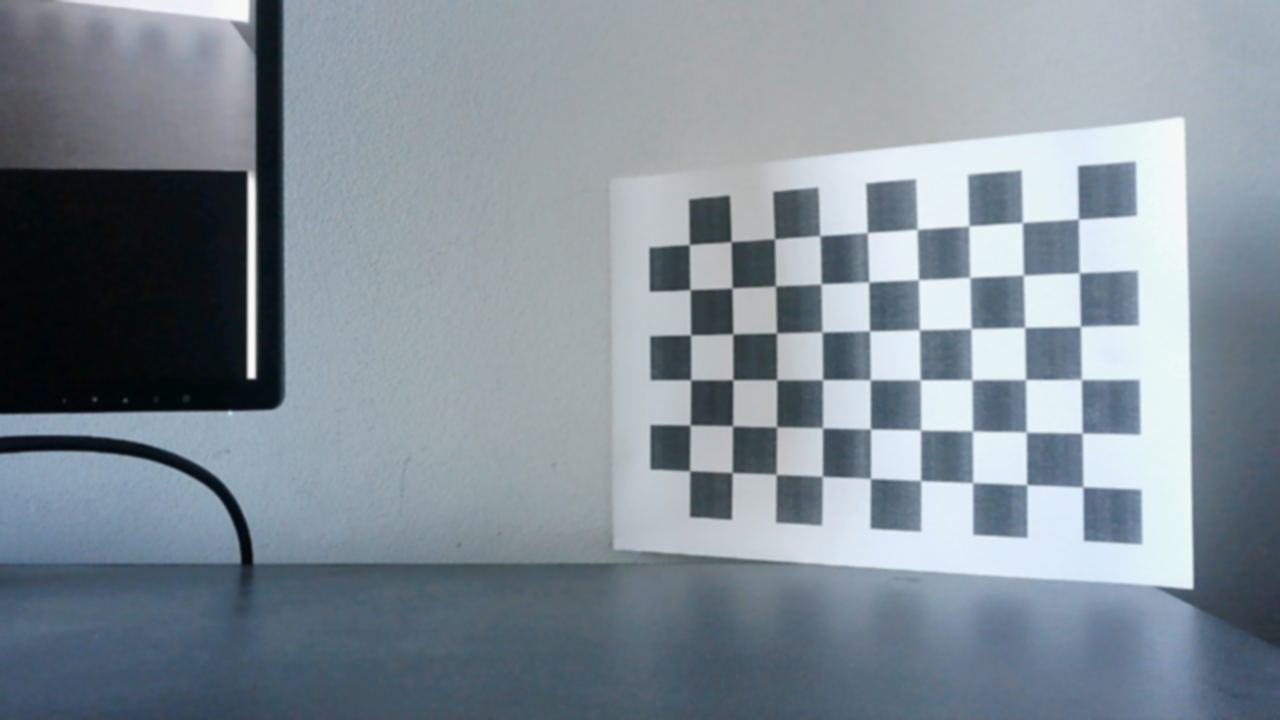

Supplement: Supplementary file 1 [file jimaging-12-00280-s001.zip › Supplementary Materials/first test/Pairs/corrupted/left/pair_0012_left.png]

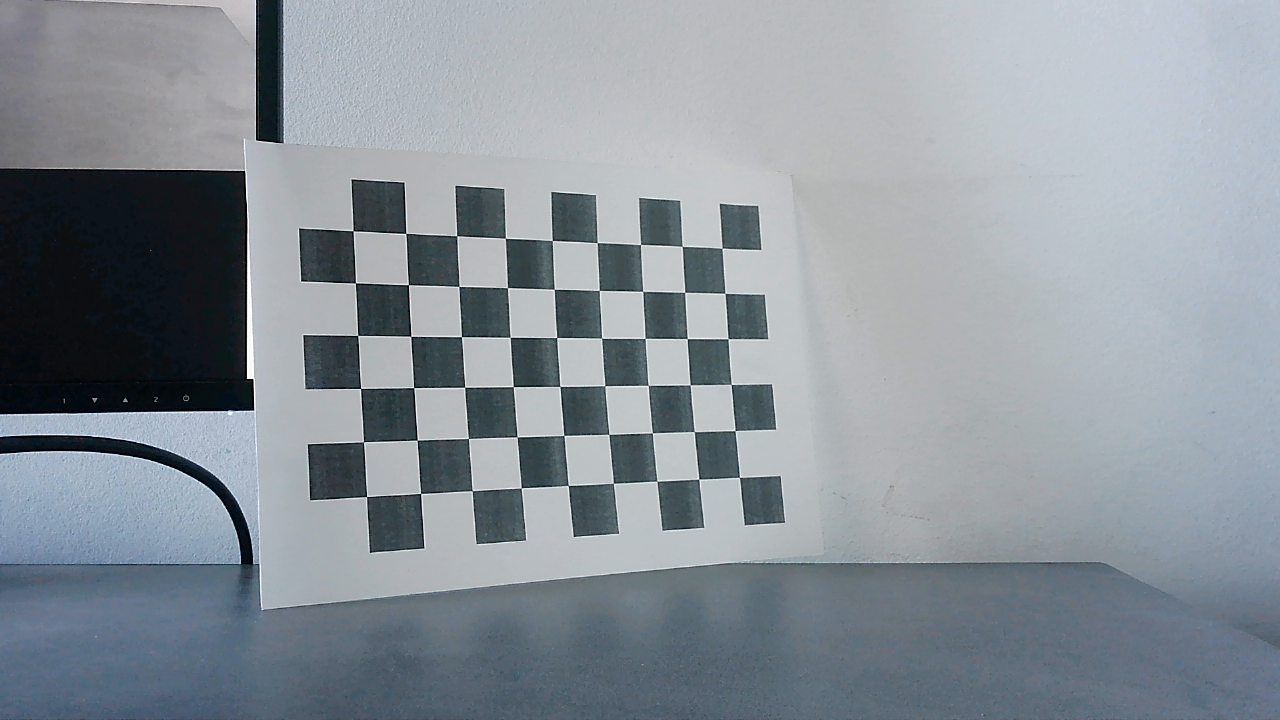

Supplement: Supplementary file 1 [file jimaging-12-00280-s001.zip › Supplementary Materials/first test/Pairs/corrupted/left/pair_0013_left.png]

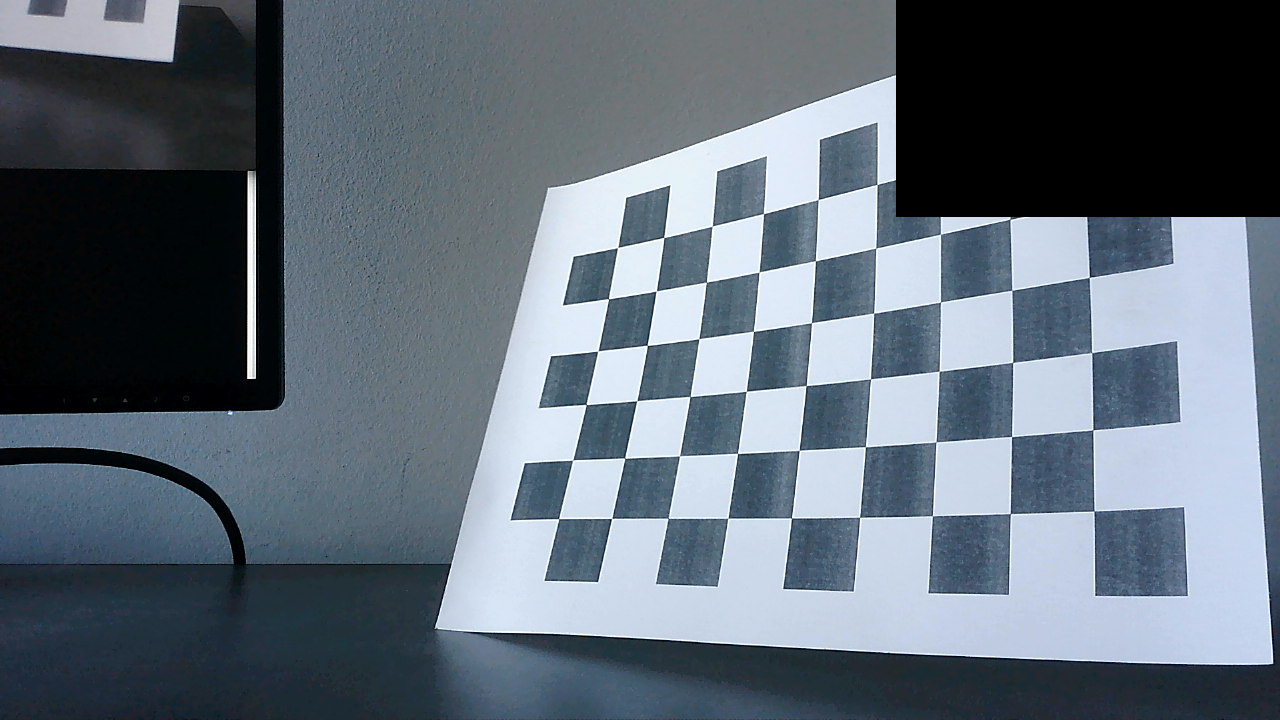

Supplement: Supplementary file 1 [file jimaging-12-00280-s001.zip › Supplementary Materials/first test/Pairs/corrupted/left/pair_0014_left.png]

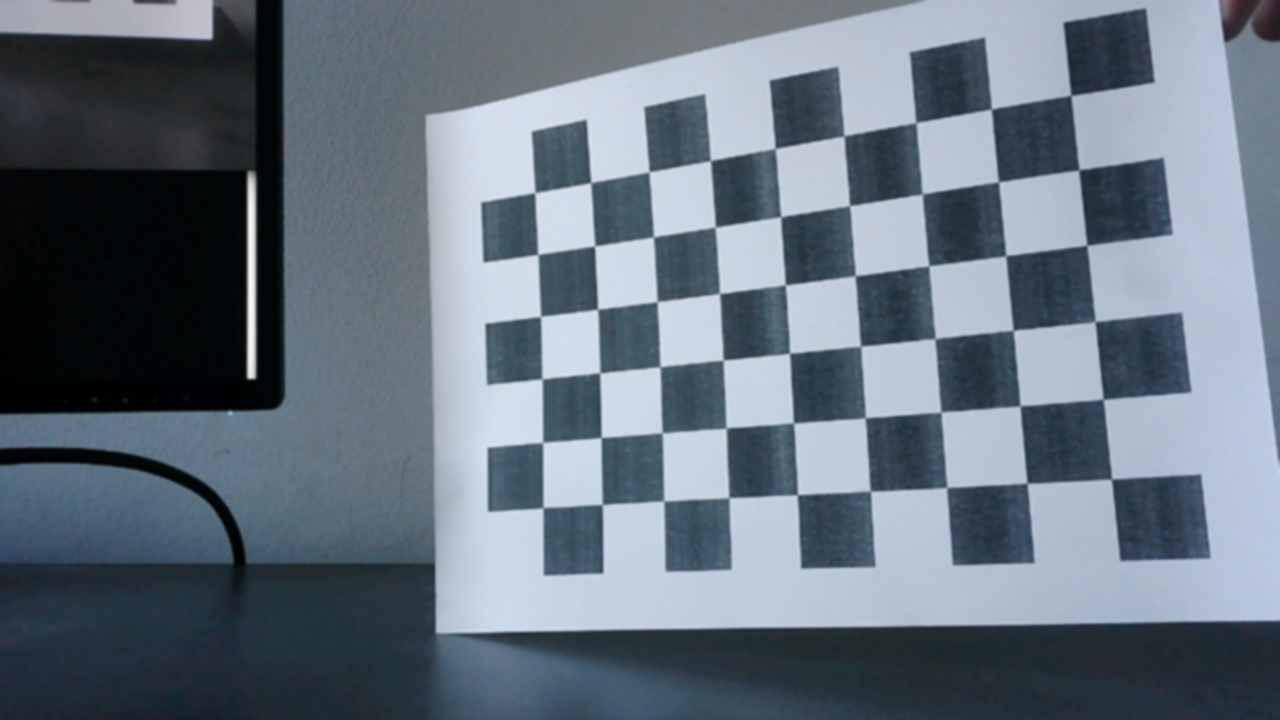

Supplement: Supplementary file 1 [file jimaging-12-00280-s001.zip › Supplementary Materials/first test/Pairs/corrupted/left/pair_0015_left.png]

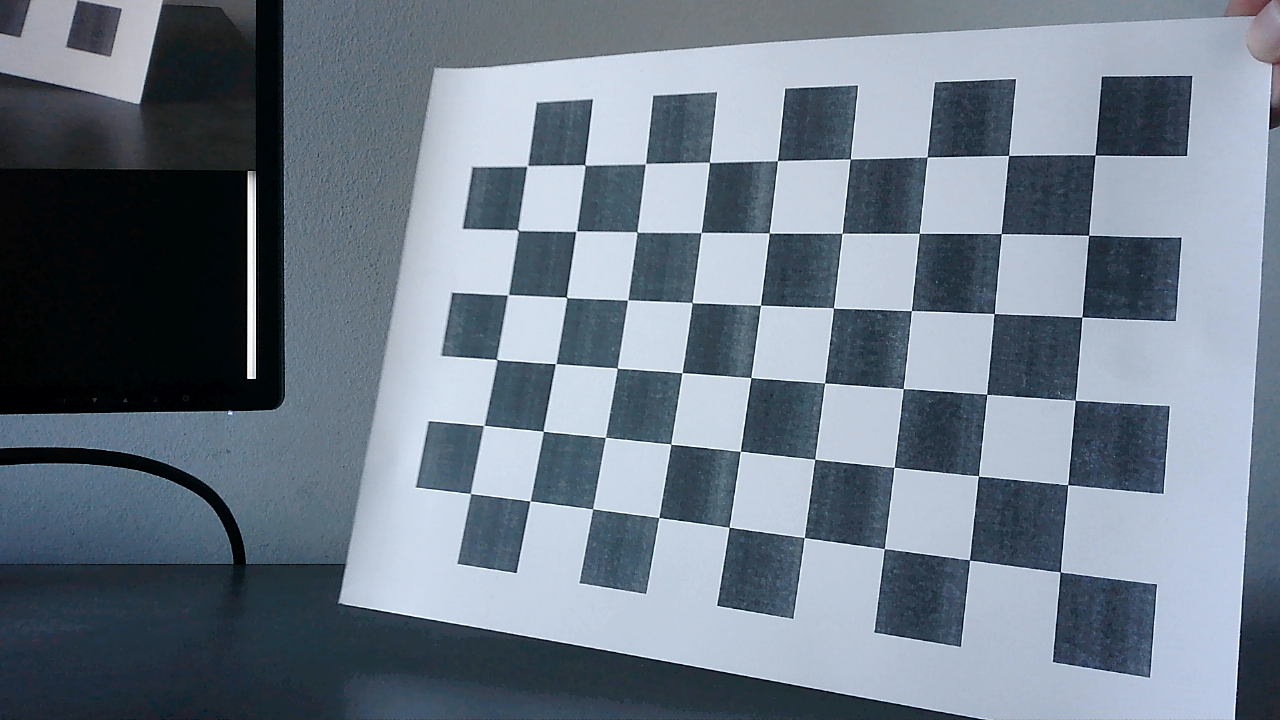

Supplement: Supplementary file 1 [file jimaging-12-00280-s001.zip › Supplementary Materials/first test/Pairs/corrupted/left/pair_0016_left.png]

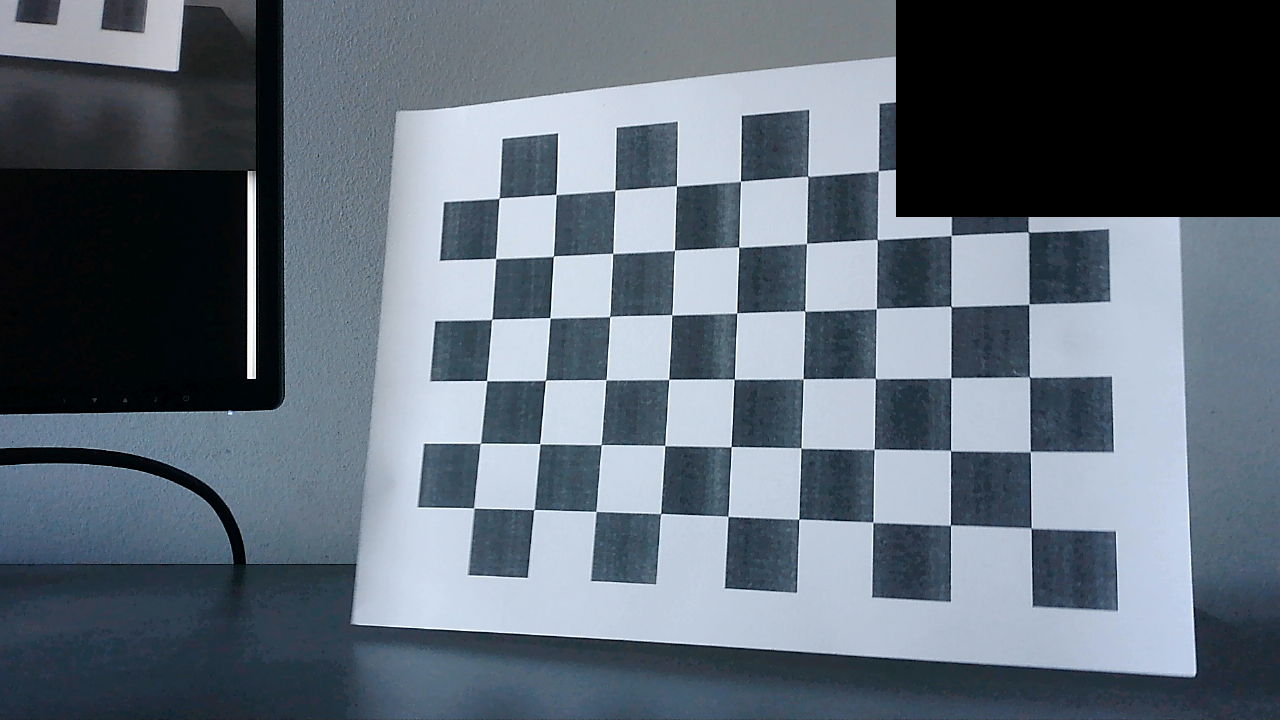

Supplement: Supplementary file 1 [file jimaging-12-00280-s001.zip › Supplementary Materials/first test/Pairs/corrupted/left/pair_0017_left.png]

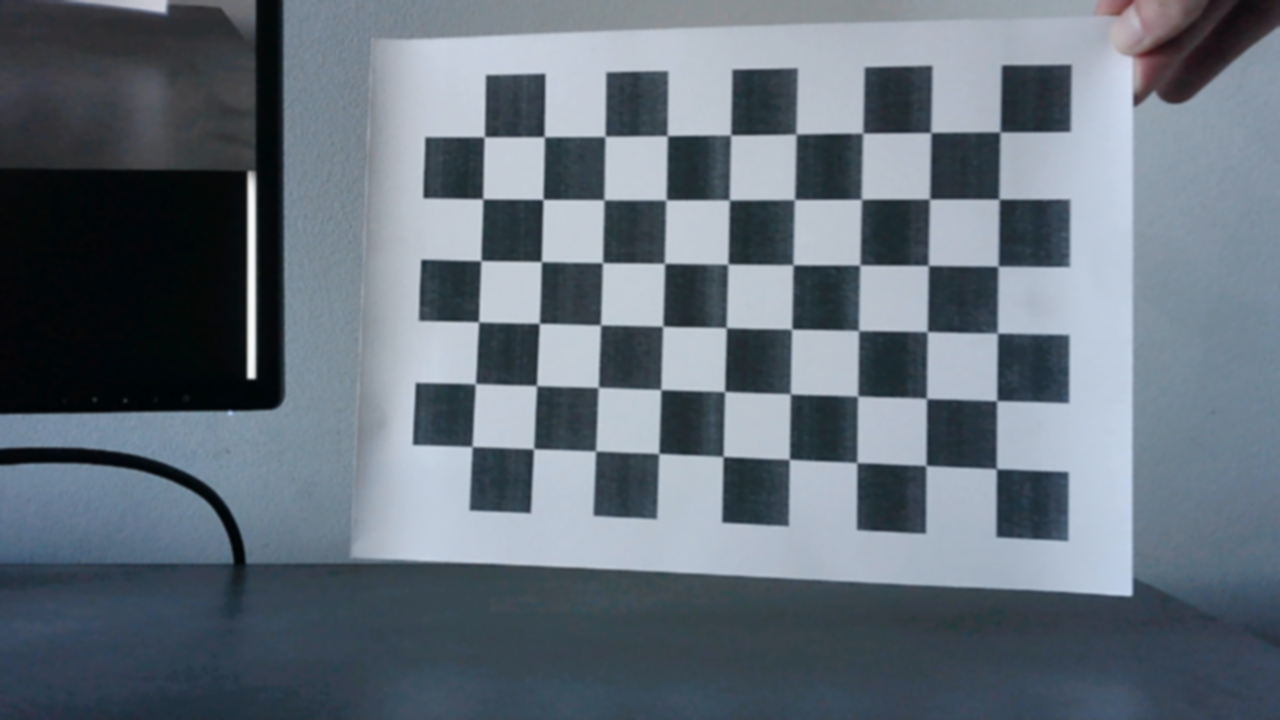

Supplement: Supplementary file 1 [file jimaging-12-00280-s001.zip › Supplementary Materials/first test/Pairs/corrupted/left/pair_0018_left.png]

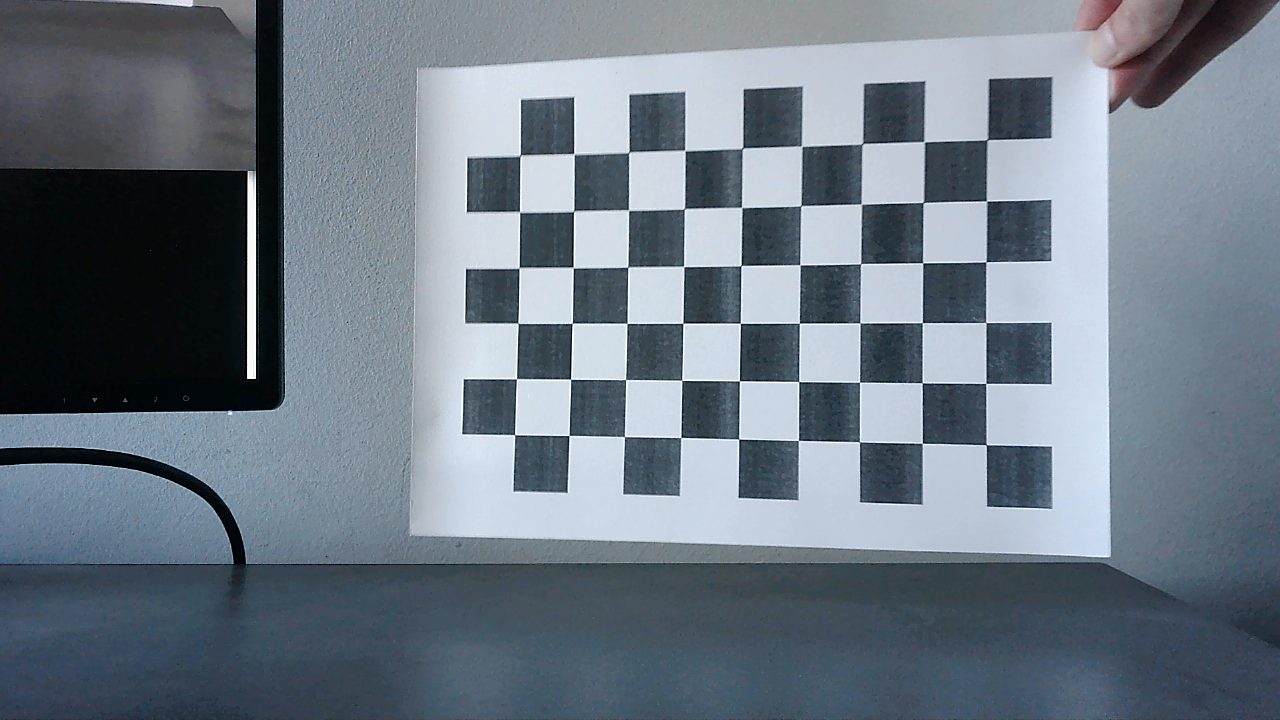

Supplement: Supplementary file 1 [file jimaging-12-00280-s001.zip › Supplementary Materials/first test/Pairs/corrupted/left/pair_0019_left.png]

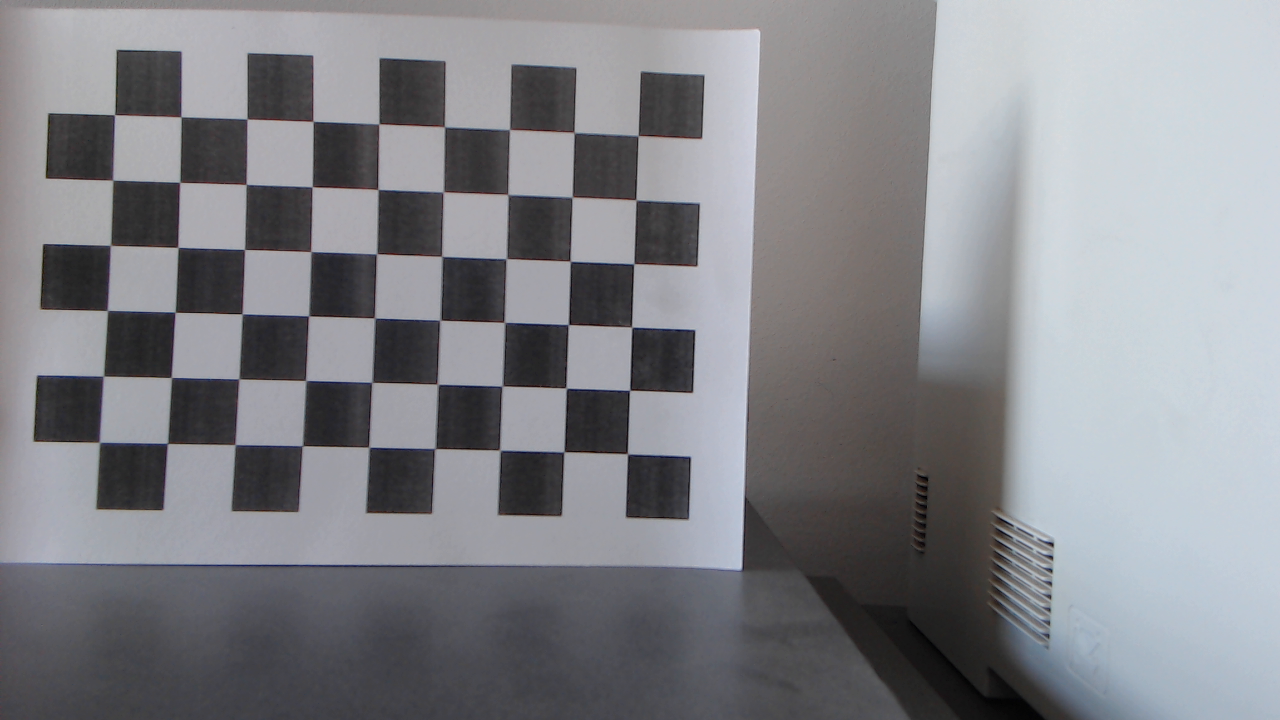

Supplement: Supplementary file 1 [file jimaging-12-00280-s001.zip › Supplementary Materials/first test/Pairs/corrupted/right/pair_0000_right.png]

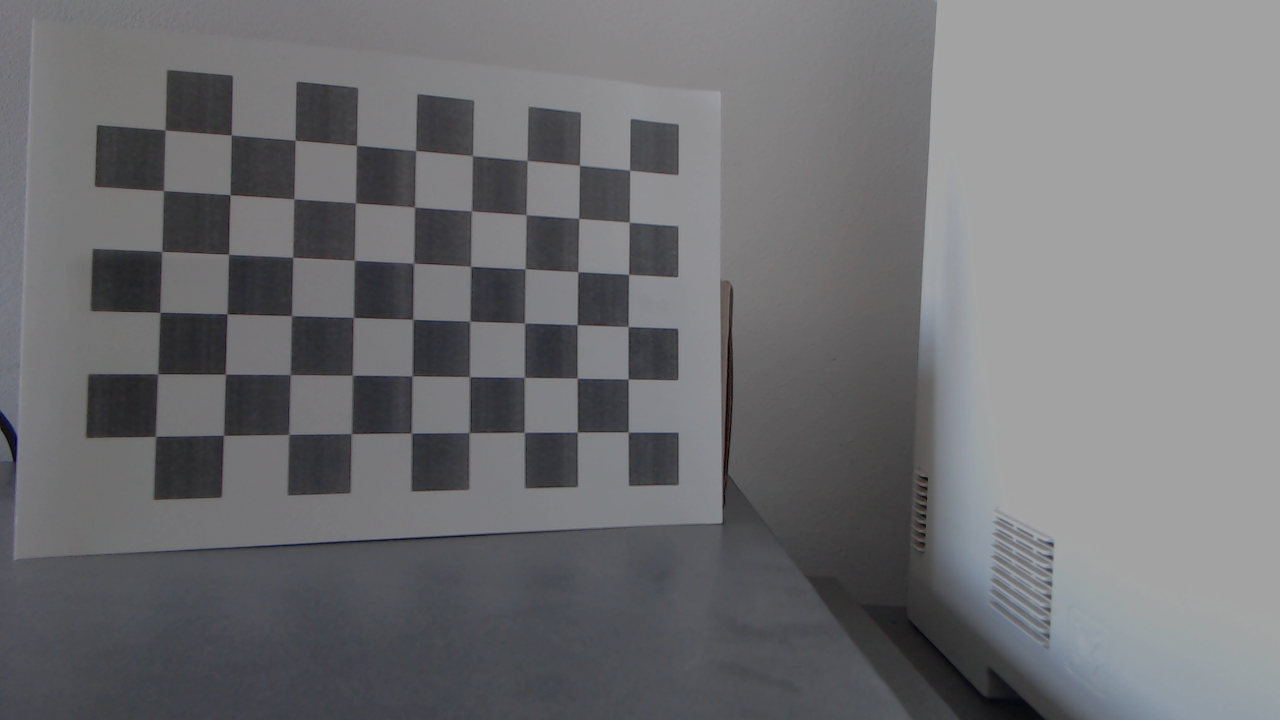

Supplement: Supplementary file 1 [file jimaging-12-00280-s001.zip › Supplementary Materials/first test/Pairs/corrupted/right/pair_0001_right.png]

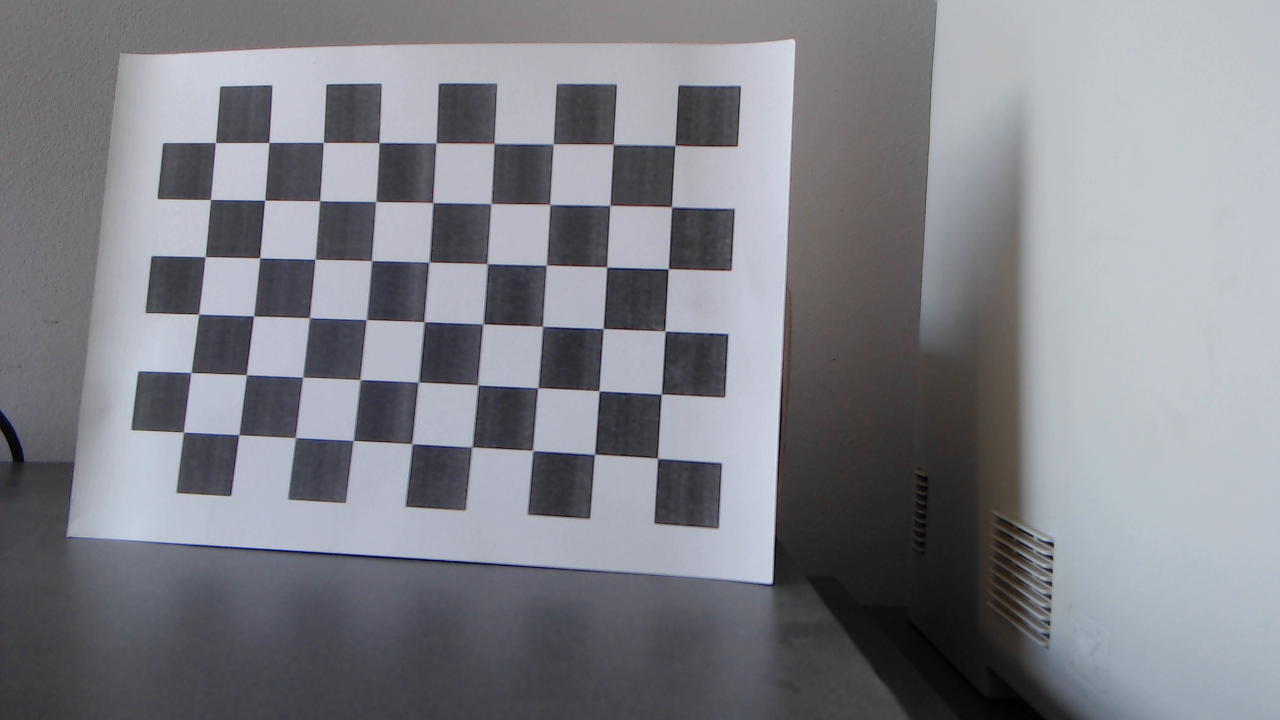

Supplement: Supplementary file 1 [file jimaging-12-00280-s001.zip › Supplementary Materials/first test/Pairs/corrupted/right/pair_0002_right.png]

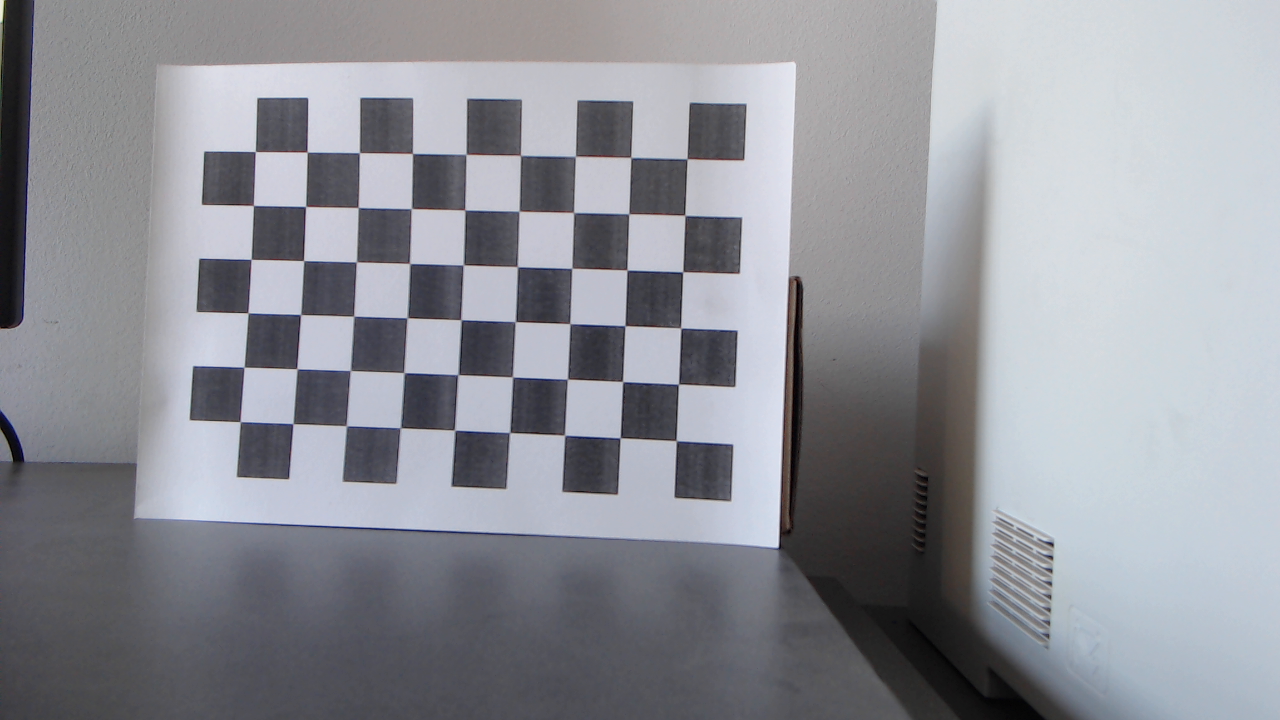

Supplement: Supplementary file 1 [file jimaging-12-00280-s001.zip › Supplementary Materials/first test/Pairs/corrupted/right/pair_0003_right.png]

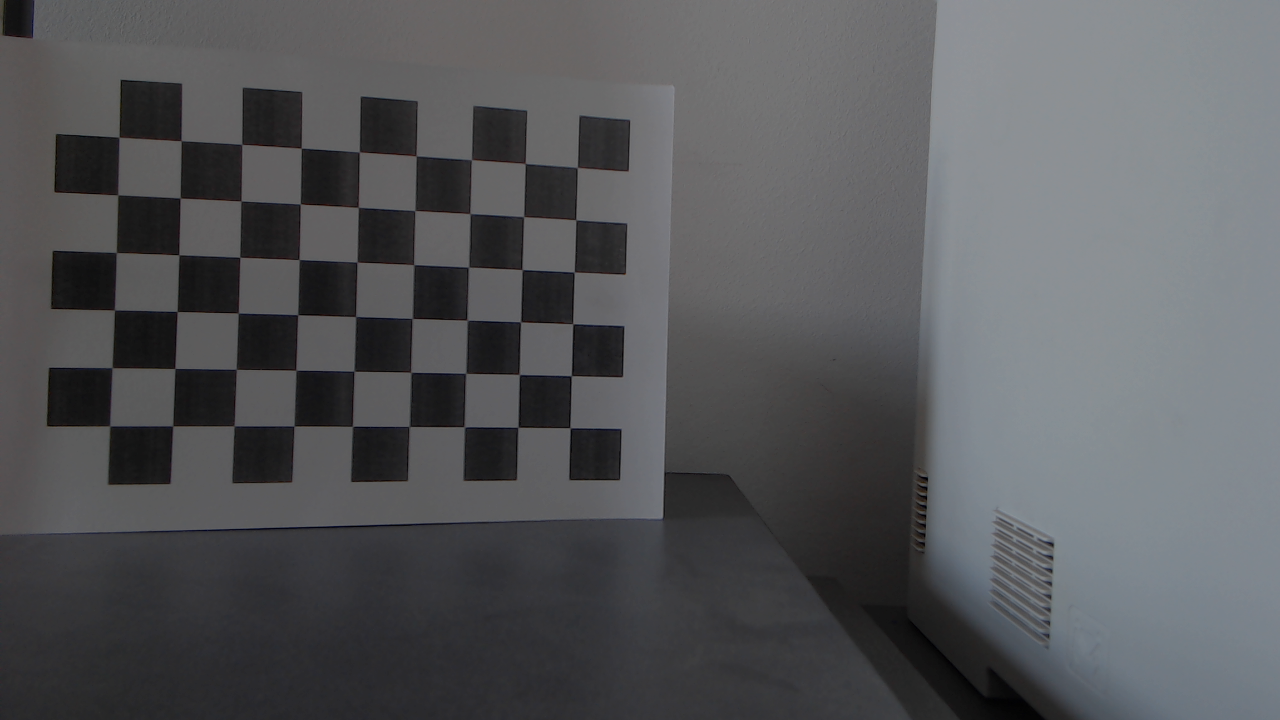

Supplement: Supplementary file 1 [file jimaging-12-00280-s001.zip › Supplementary Materials/first test/Pairs/corrupted/right/pair_0004_right.png]

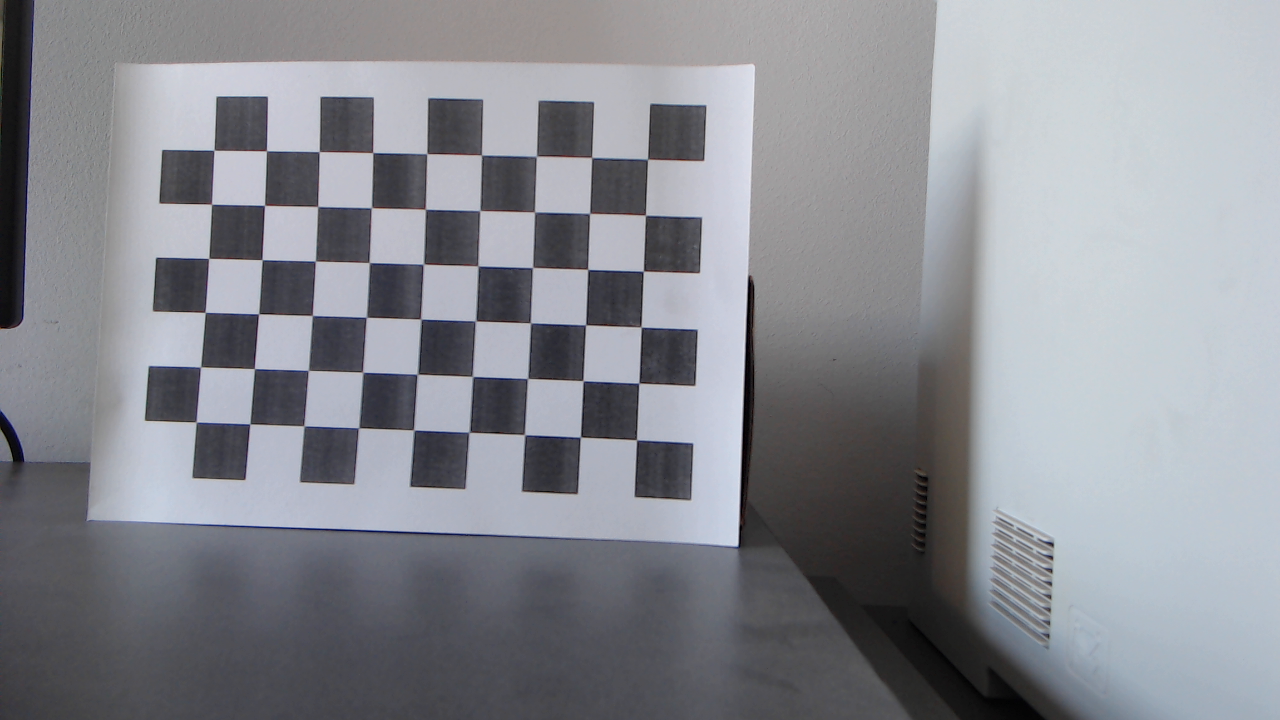

Supplement: Supplementary file 1 [file jimaging-12-00280-s001.zip › Supplementary Materials/first test/Pairs/corrupted/right/pair_0005_right.png]

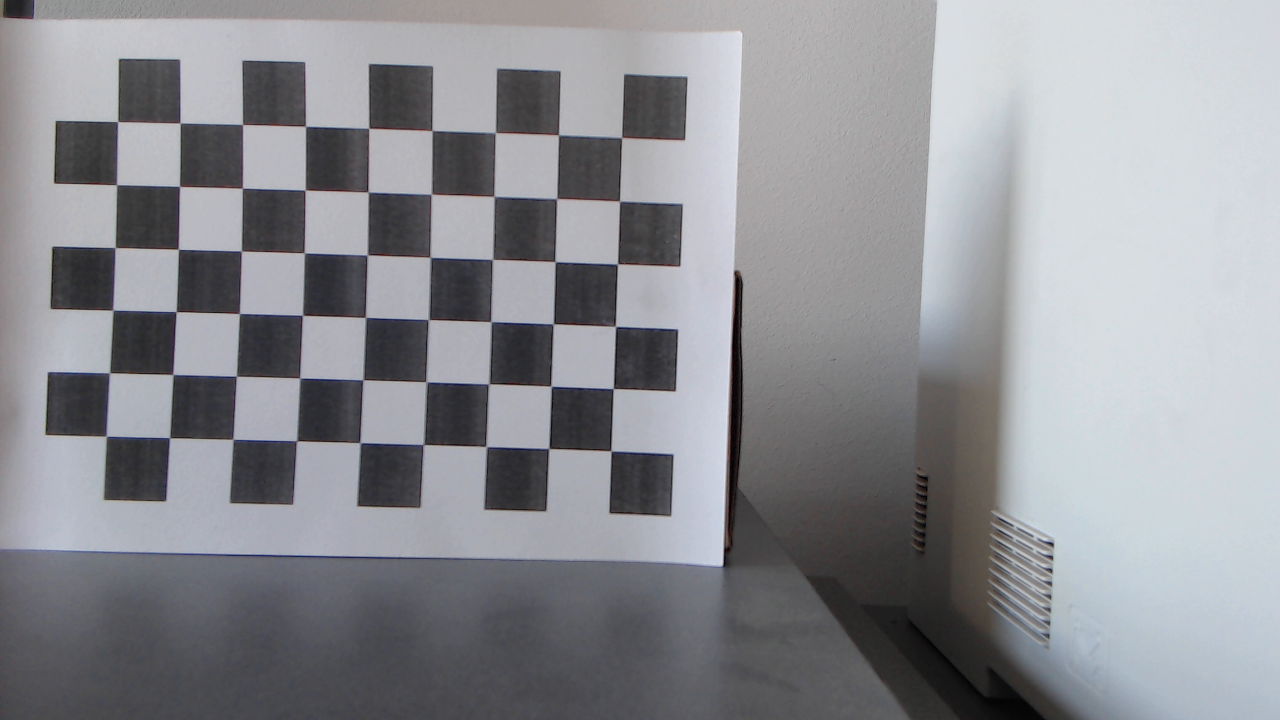

Supplement: Supplementary file 1 [file jimaging-12-00280-s001.zip › Supplementary Materials/first test/Pairs/corrupted/right/pair_0006_right.png]

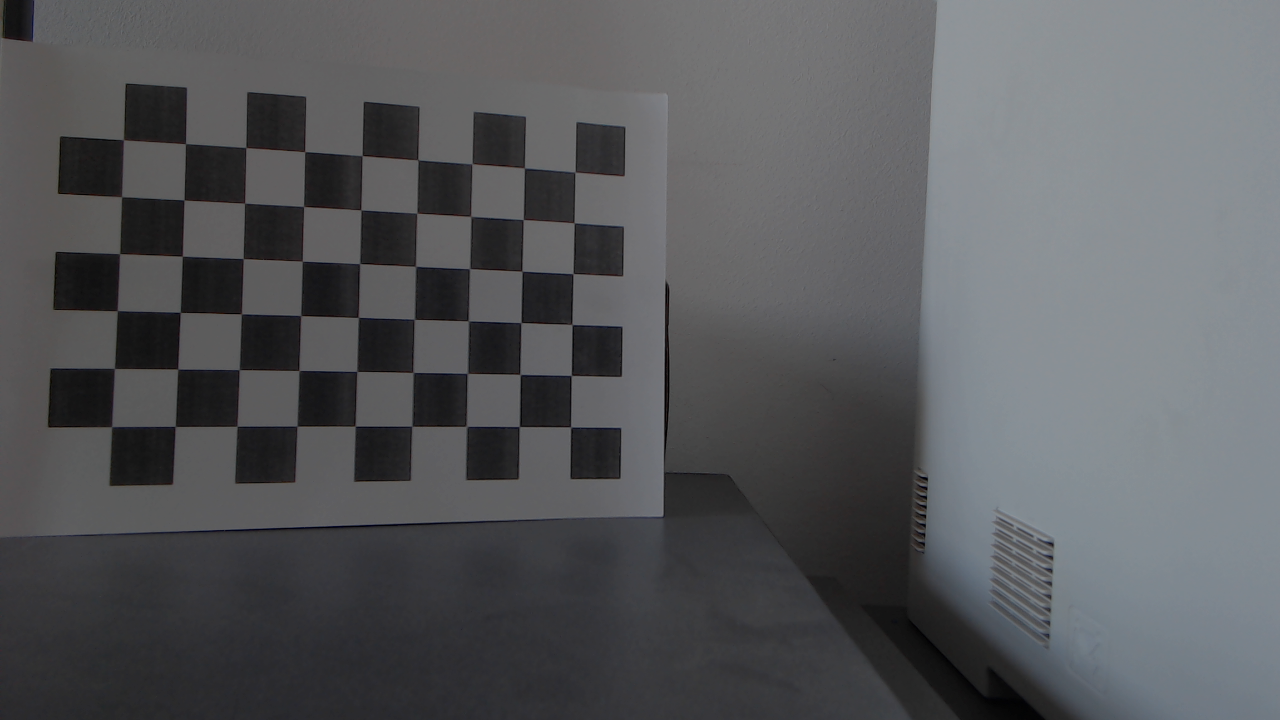

Supplement: Supplementary file 1 [file jimaging-12-00280-s001.zip › Supplementary Materials/first test/Pairs/corrupted/right/pair_0007_right.png]

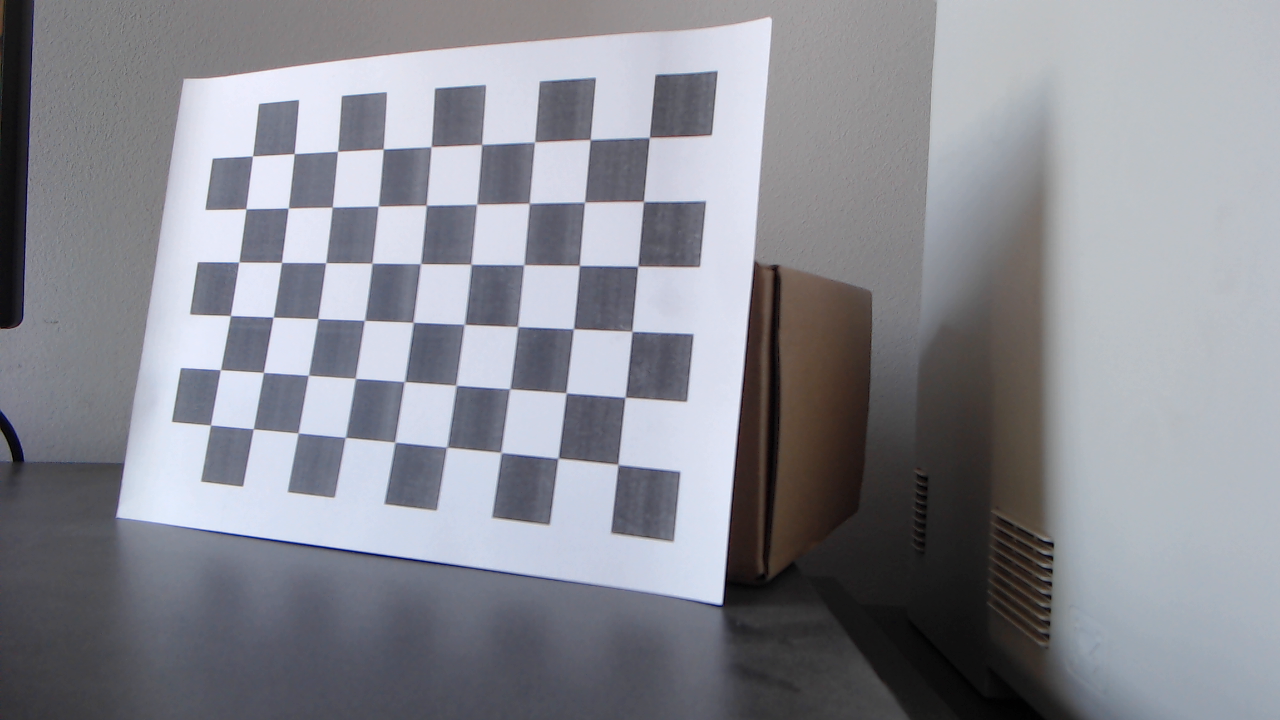

Supplement: Supplementary file 1 [file jimaging-12-00280-s001.zip › Supplementary Materials/first test/Pairs/corrupted/right/pair_0008_right.png]

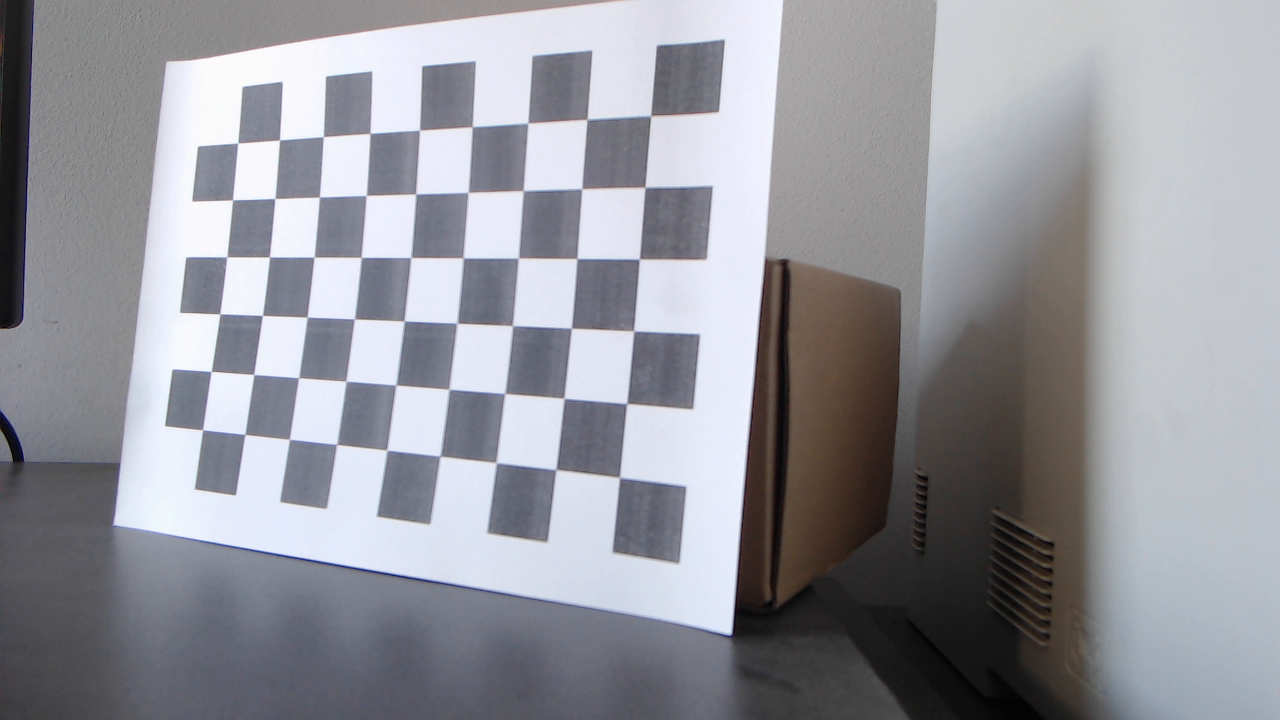

Supplement: Supplementary file 1 [file jimaging-12-00280-s001.zip › Supplementary Materials/first test/Pairs/corrupted/right/pair_0009_right.png]

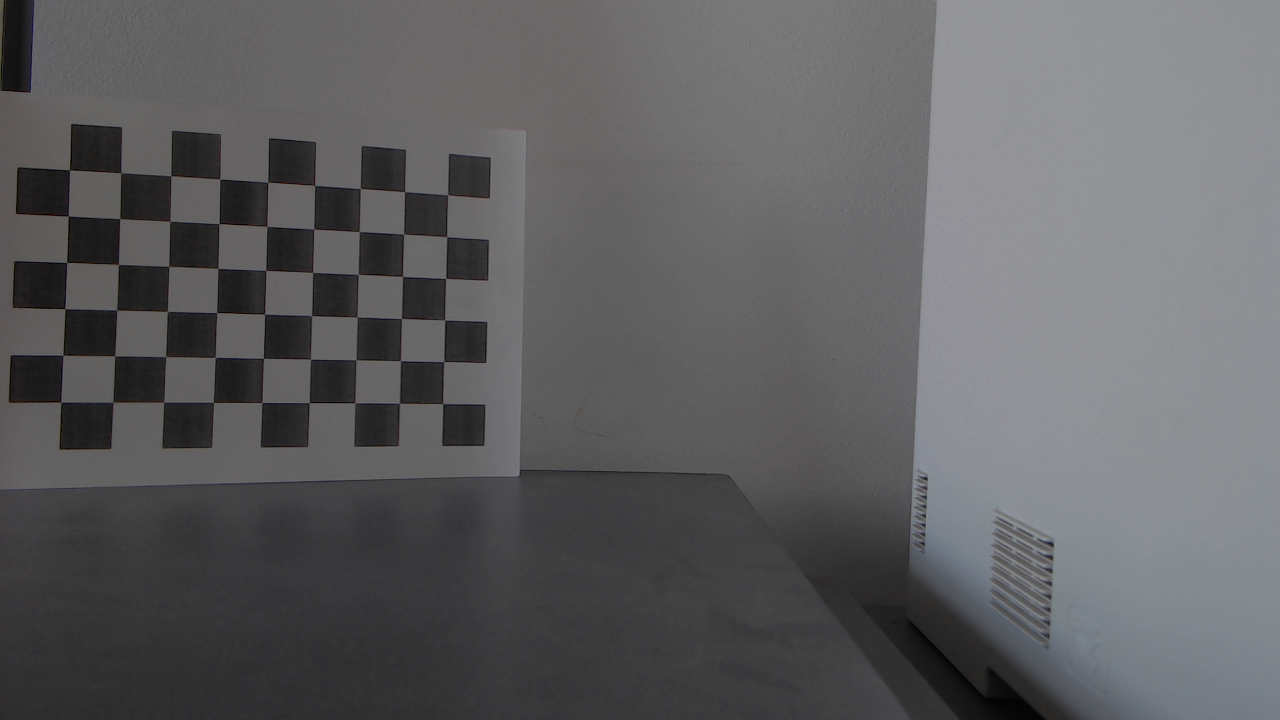

Supplement: Supplementary file 1 [file jimaging-12-00280-s001.zip › Supplementary Materials/first test/Pairs/corrupted/right/pair_0010_right.png]

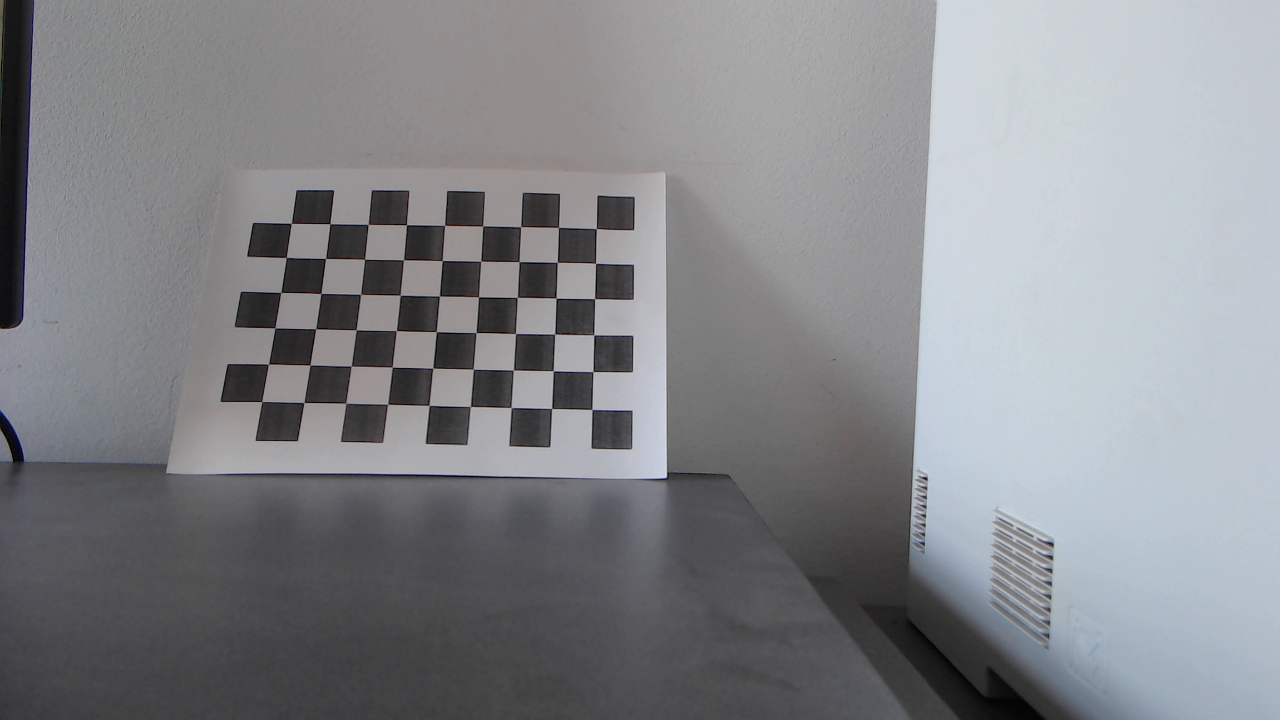

Supplement: Supplementary file 1 [file jimaging-12-00280-s001.zip › Supplementary Materials/first test/Pairs/corrupted/right/pair_0011_right.png]

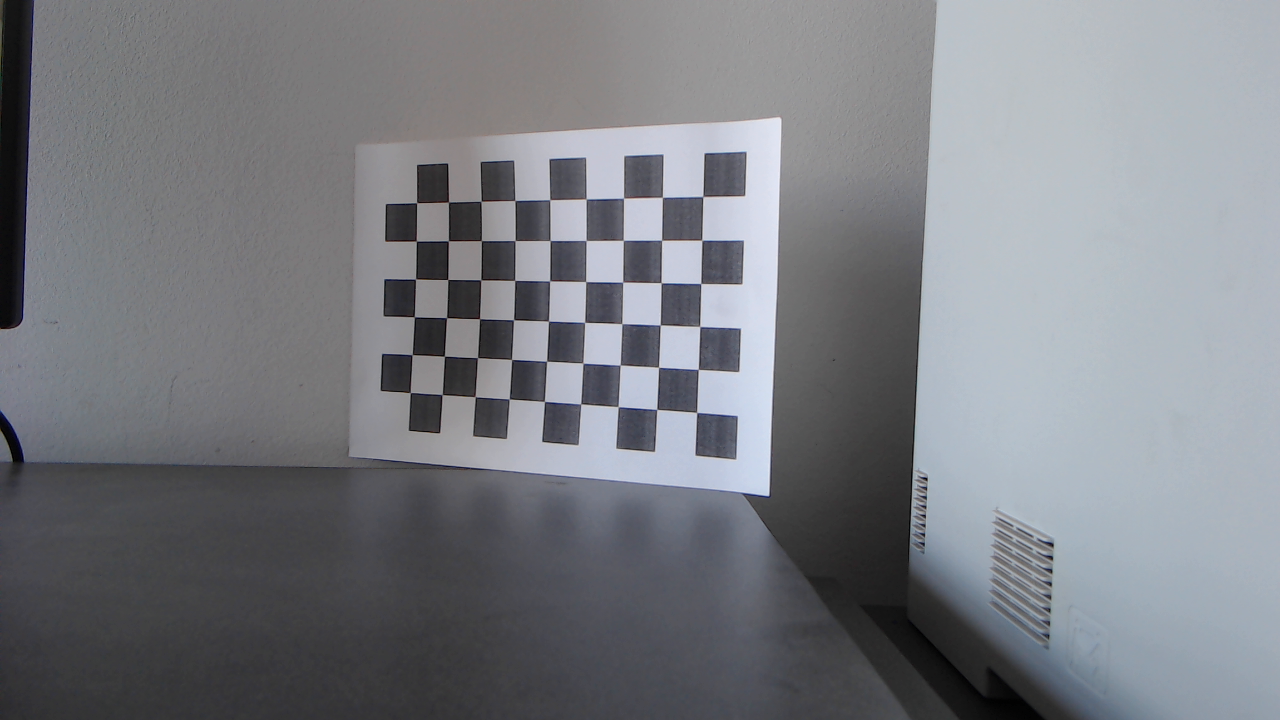

Supplement: Supplementary file 1 [file jimaging-12-00280-s001.zip › Supplementary Materials/first test/Pairs/corrupted/right/pair_0012_right.png]

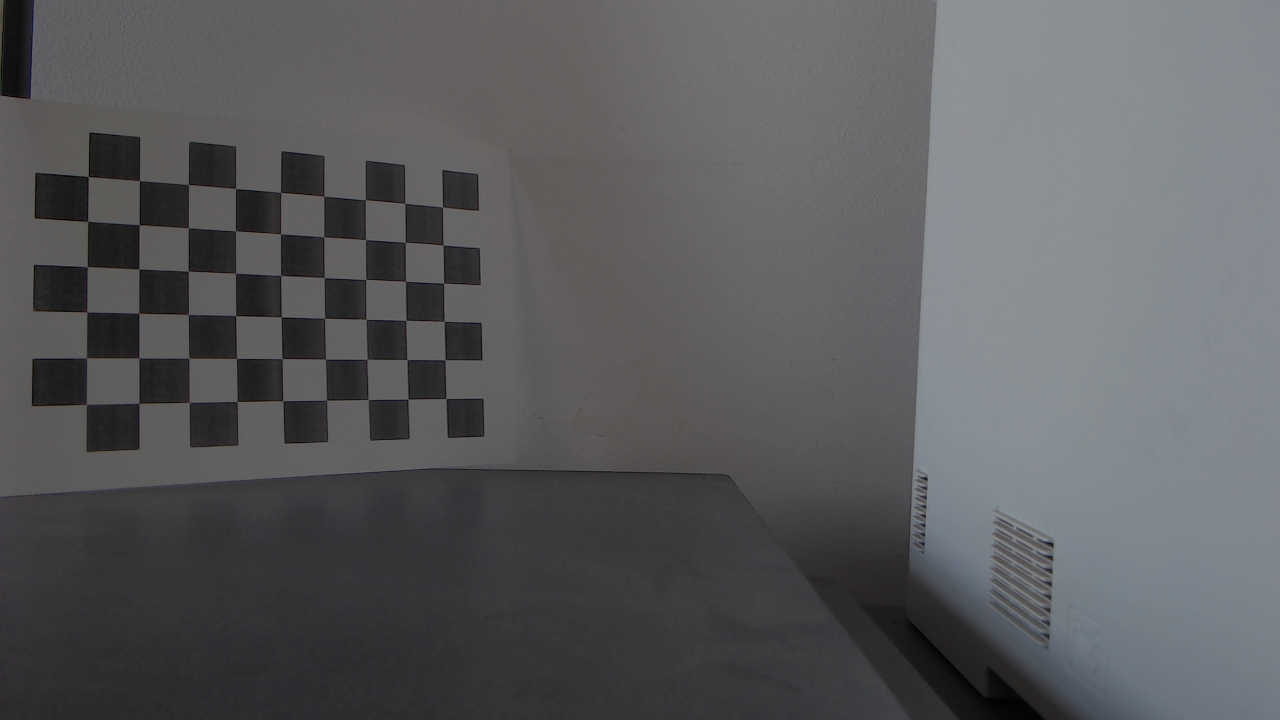

Supplement: Supplementary file 1 [file jimaging-12-00280-s001.zip › Supplementary Materials/first test/Pairs/corrupted/right/pair_0013_right.png]

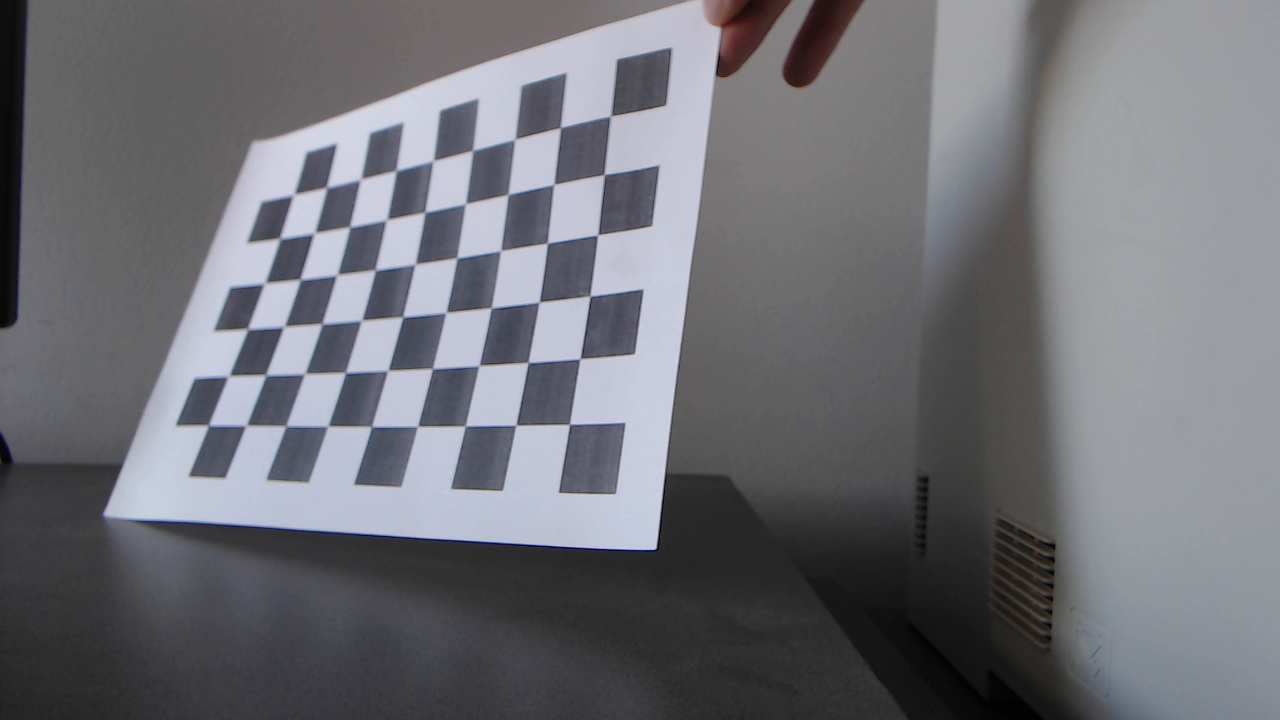

Supplement: Supplementary file 1 [file jimaging-12-00280-s001.zip › Supplementary Materials/first test/Pairs/corrupted/right/pair_0014_right.png]

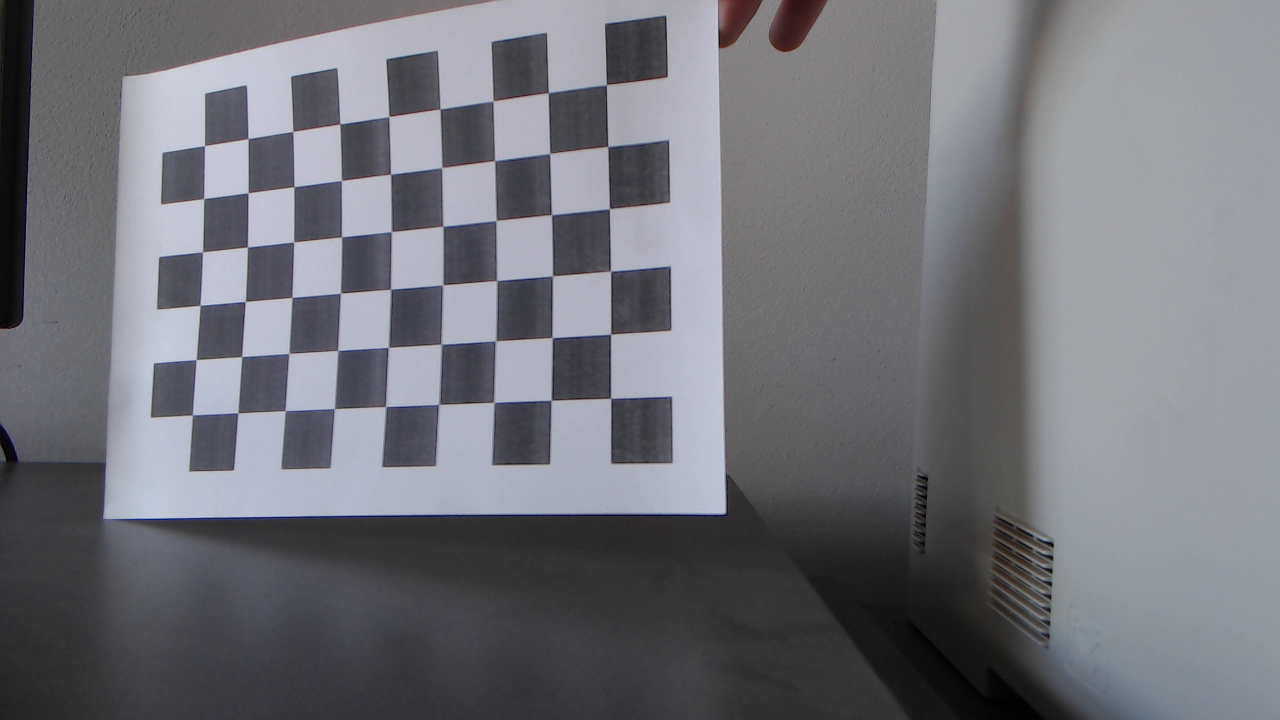

Supplement: Supplementary file 1 [file jimaging-12-00280-s001.zip › Supplementary Materials/first test/Pairs/corrupted/right/pair_0015_right.png]

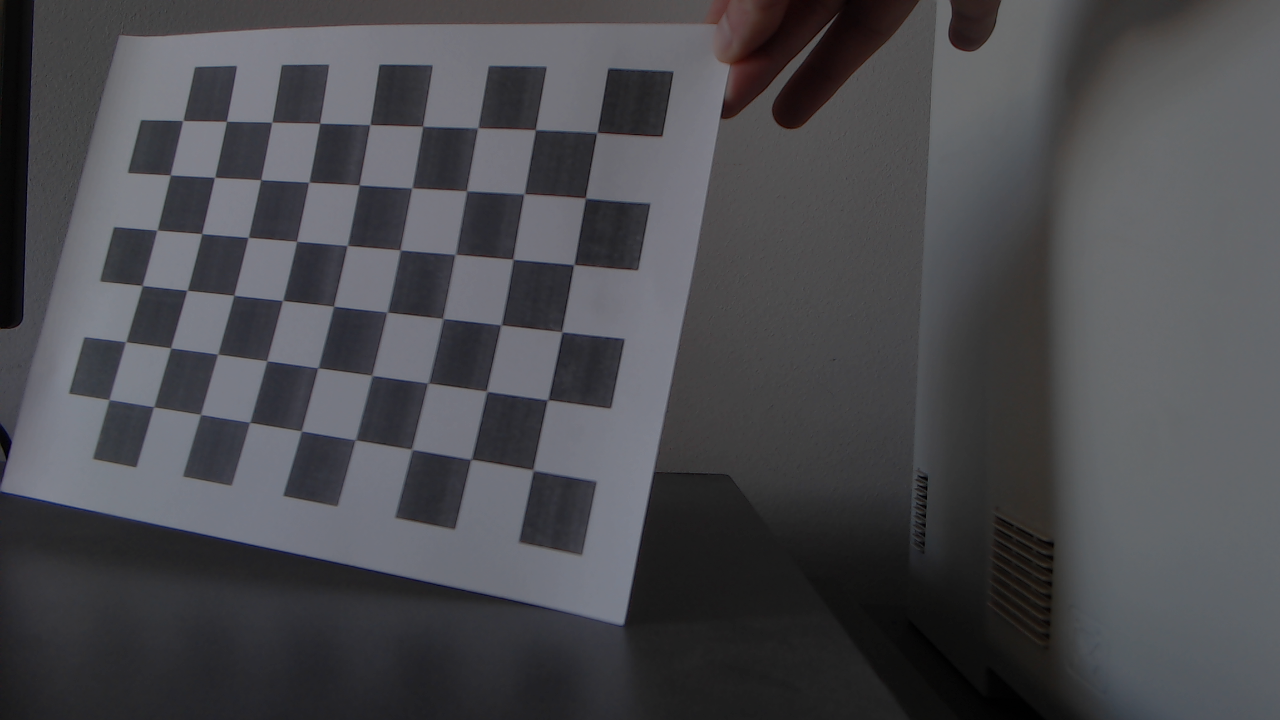

Supplement: Supplementary file 1 [file jimaging-12-00280-s001.zip › Supplementary Materials/first test/Pairs/corrupted/right/pair_0016_right.png]

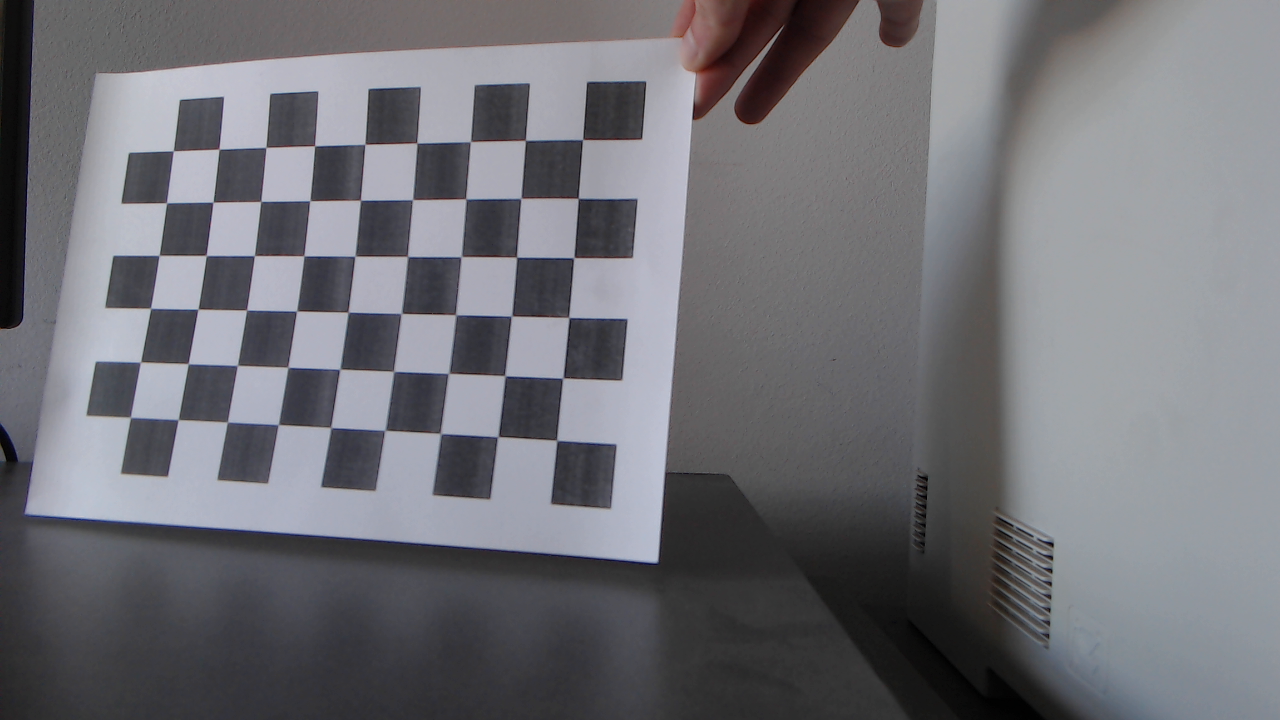

Supplement: Supplementary file 1 [file jimaging-12-00280-s001.zip › Supplementary Materials/first test/Pairs/corrupted/right/pair_0017_right.png]

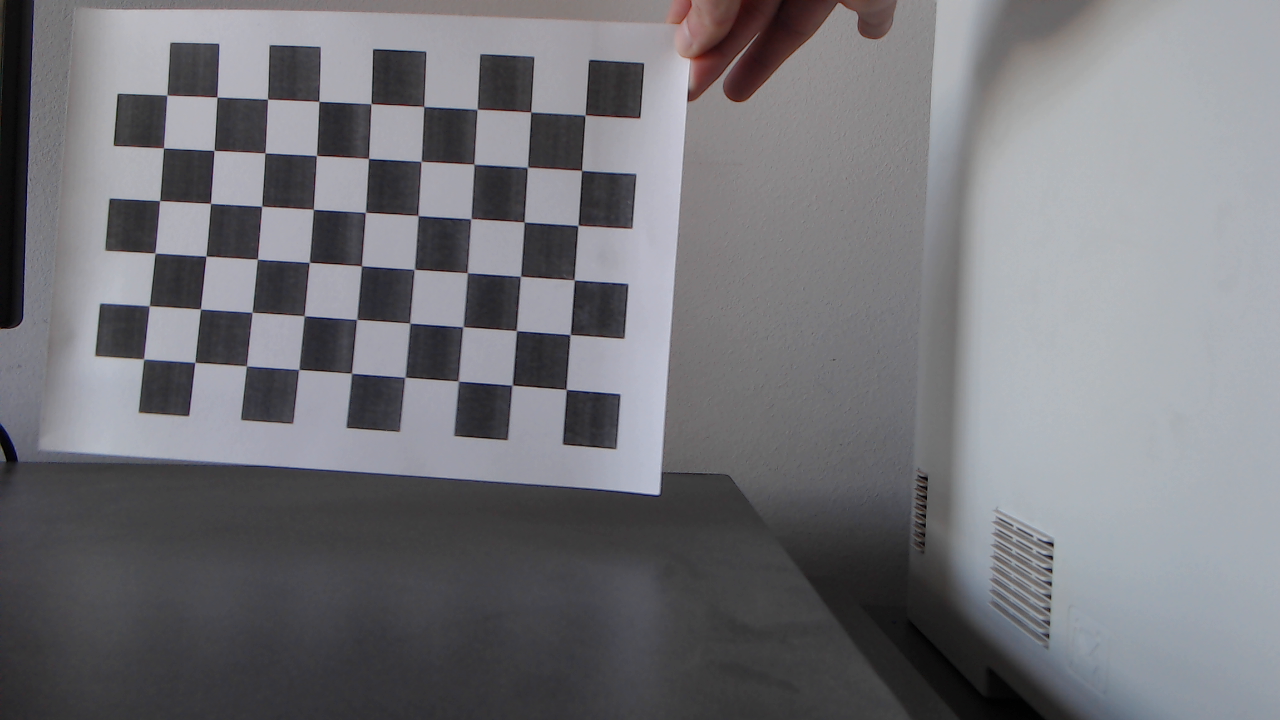

Supplement: Supplementary file 1 [file jimaging-12-00280-s001.zip › Supplementary Materials/first test/Pairs/corrupted/right/pair_0018_right.png]

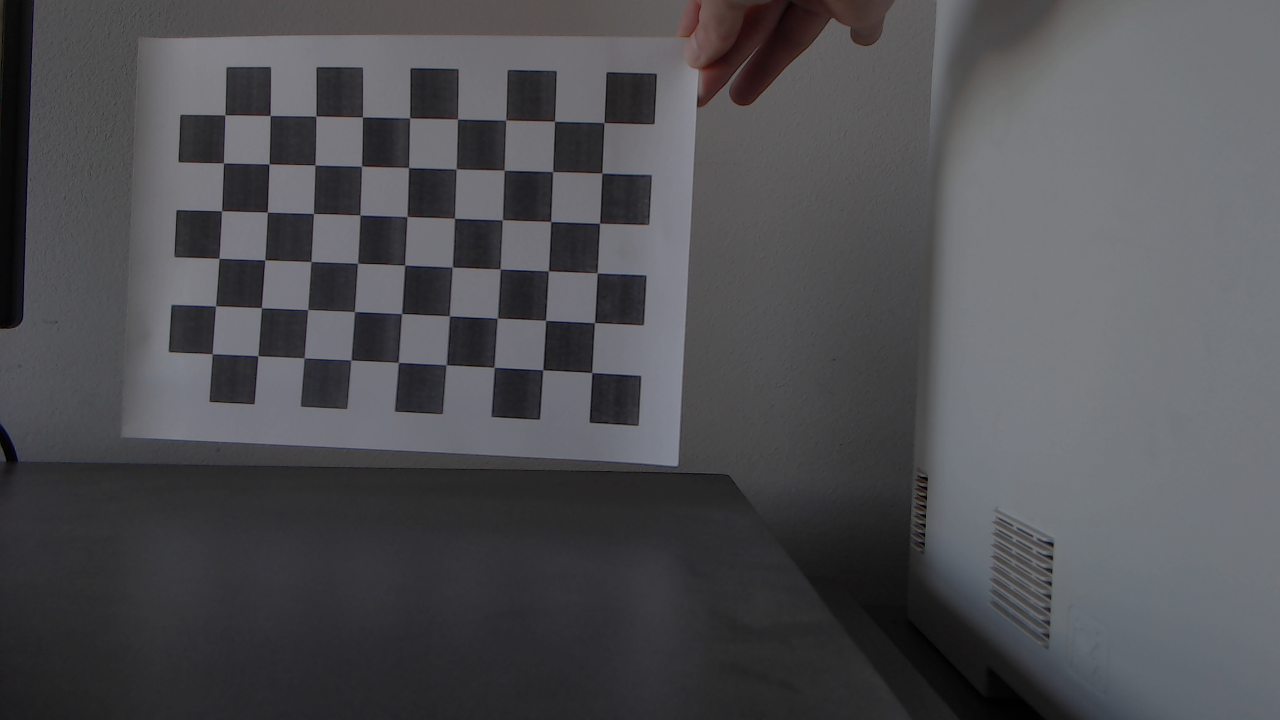

Supplement: Supplementary file 1 [file jimaging-12-00280-s001.zip › Supplementary Materials/first test/Pairs/corrupted/right/pair_0019_right.png]

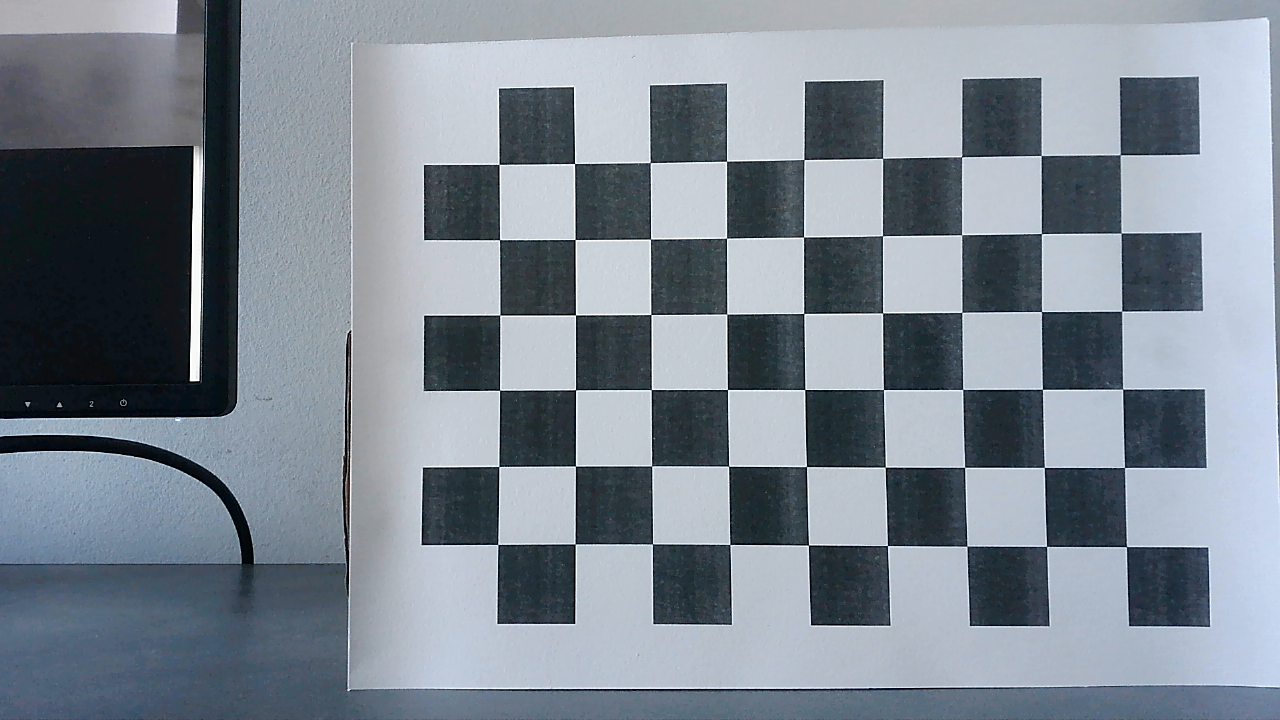

Supplement: Supplementary file 1 [file jimaging-12-00280-s001.zip › Supplementary Materials/first test/Pairs/raw/left/pair_0000_left.png]

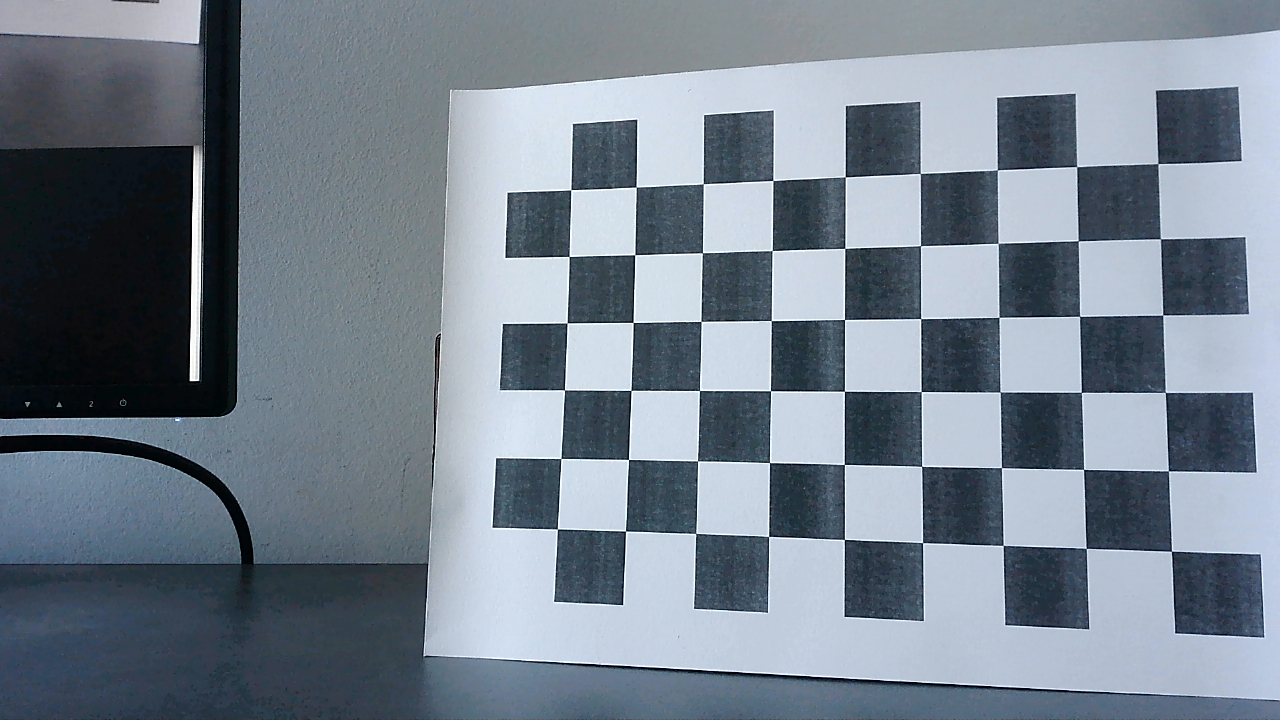

Supplement: Supplementary file 1 [file jimaging-12-00280-s001.zip › Supplementary Materials/first test/Pairs/raw/left/pair_0002_left.png]

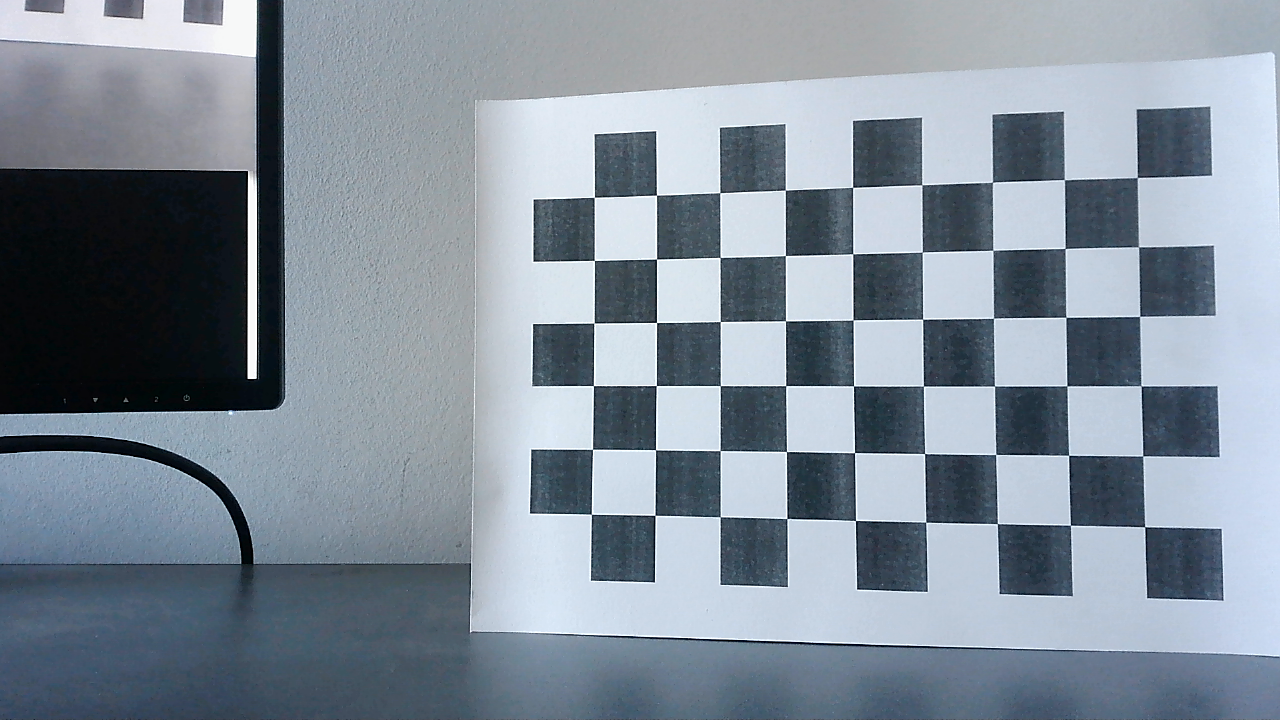

Supplement: Supplementary file 1 [file jimaging-12-00280-s001.zip › Supplementary Materials/first test/Pairs/raw/left/pair_0003_left.png]

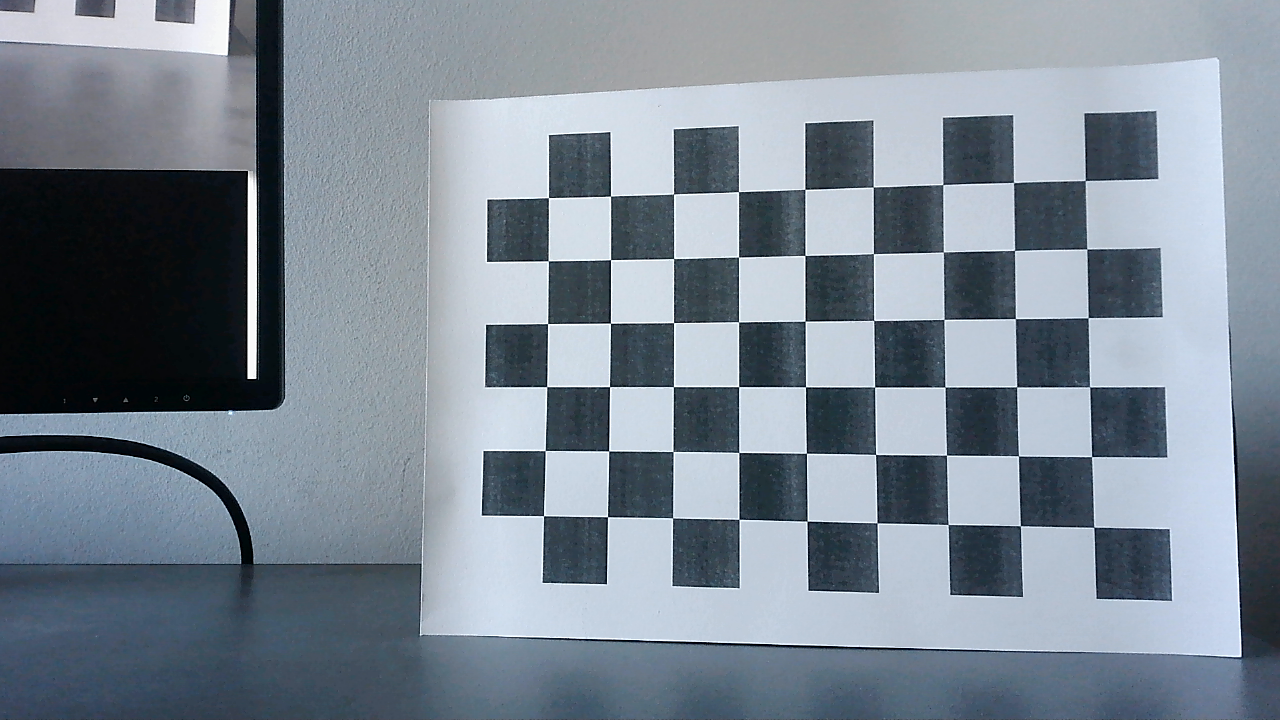

Supplement: Supplementary file 1 [file jimaging-12-00280-s001.zip › Supplementary Materials/first test/Pairs/raw/left/pair_0005_left.png]

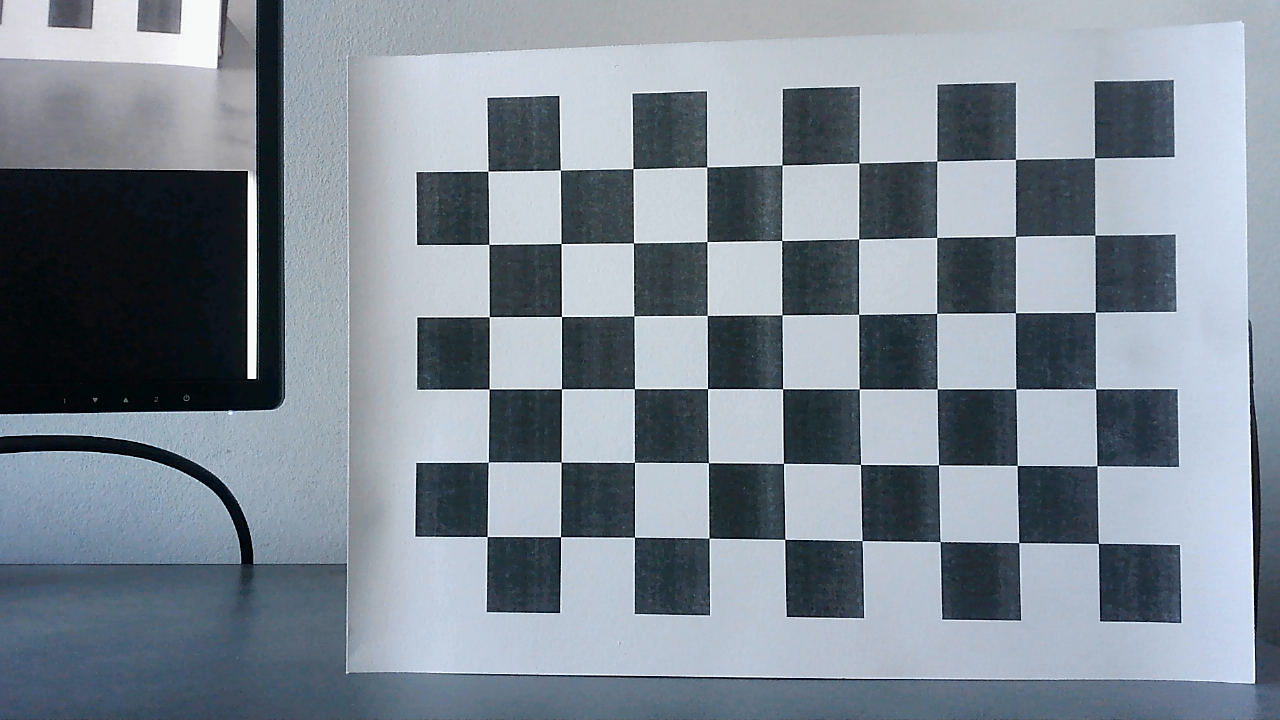

Supplement: Supplementary file 1 [file jimaging-12-00280-s001.zip › Supplementary Materials/first test/Pairs/raw/left/pair_0006_left.png]

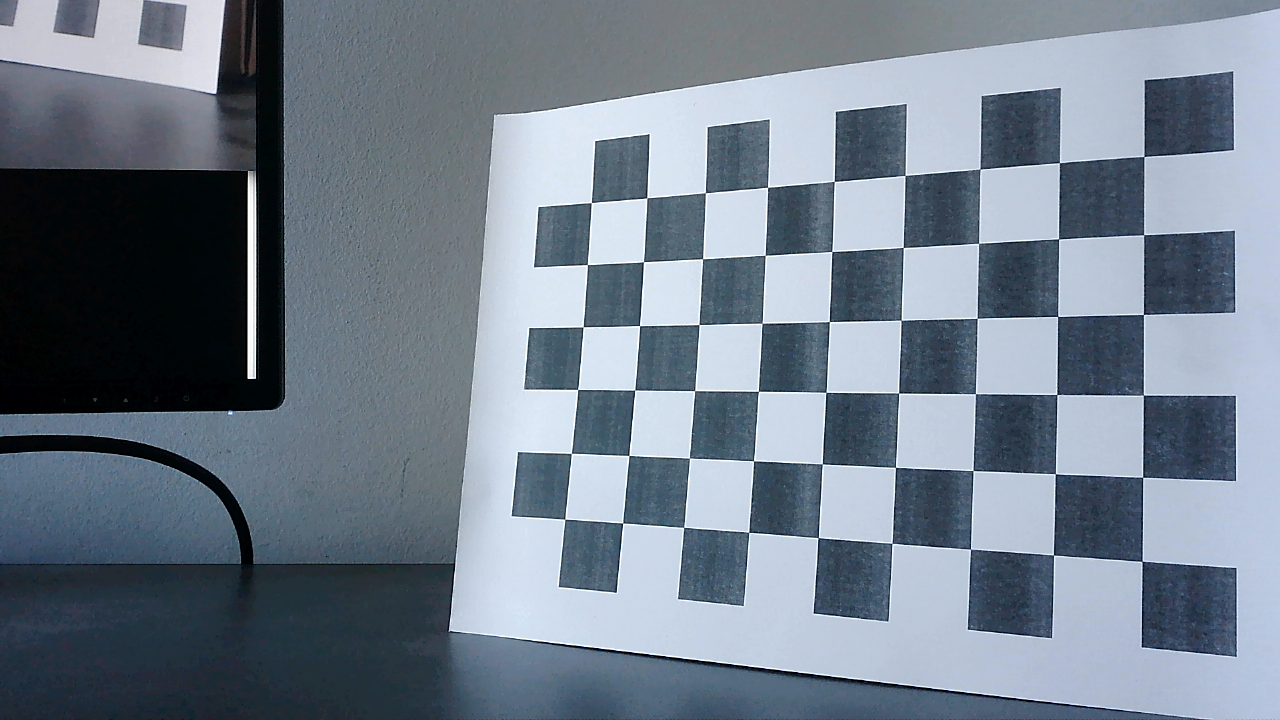

Supplement: Supplementary file 1 [file jimaging-12-00280-s001.zip › Supplementary Materials/first test/Pairs/raw/left/pair_0008_left.png]

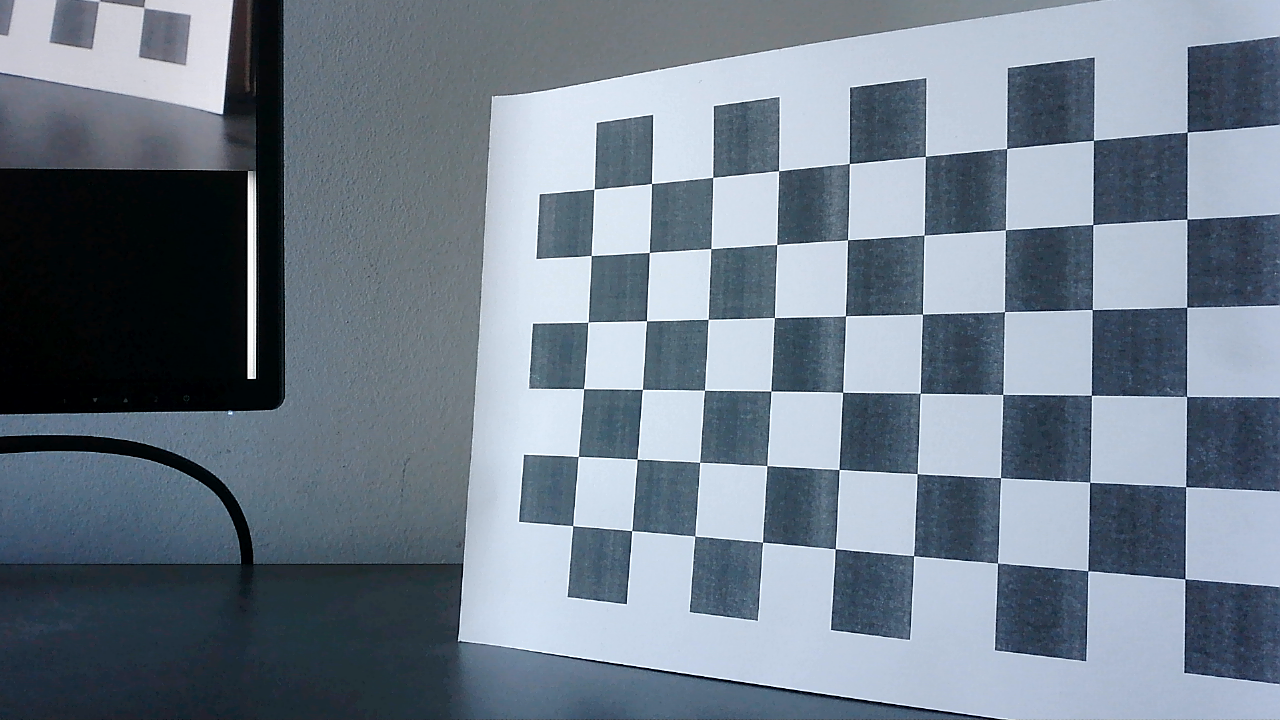

Supplement: Supplementary file 1 [file jimaging-12-00280-s001.zip › Supplementary Materials/first test/Pairs/raw/left/pair_0009_left.png]

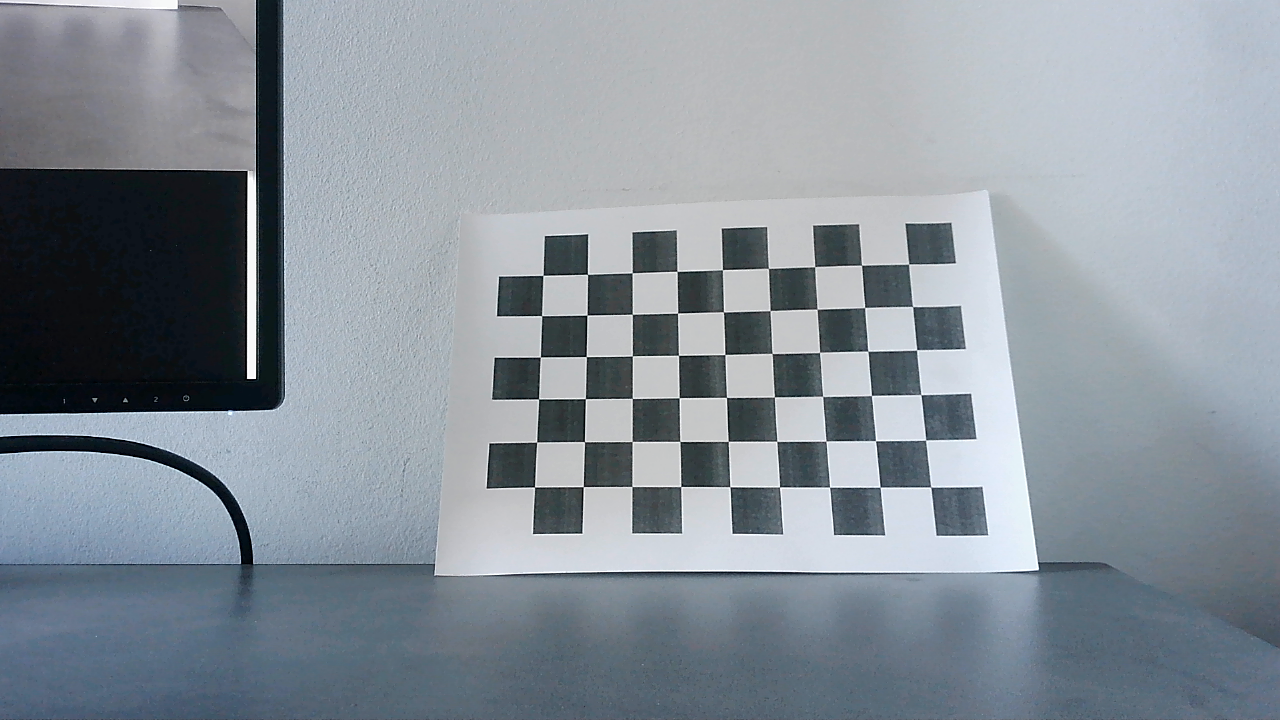

Supplement: Supplementary file 1 [file jimaging-12-00280-s001.zip › Supplementary Materials/first test/Pairs/raw/left/pair_0011_left.png]

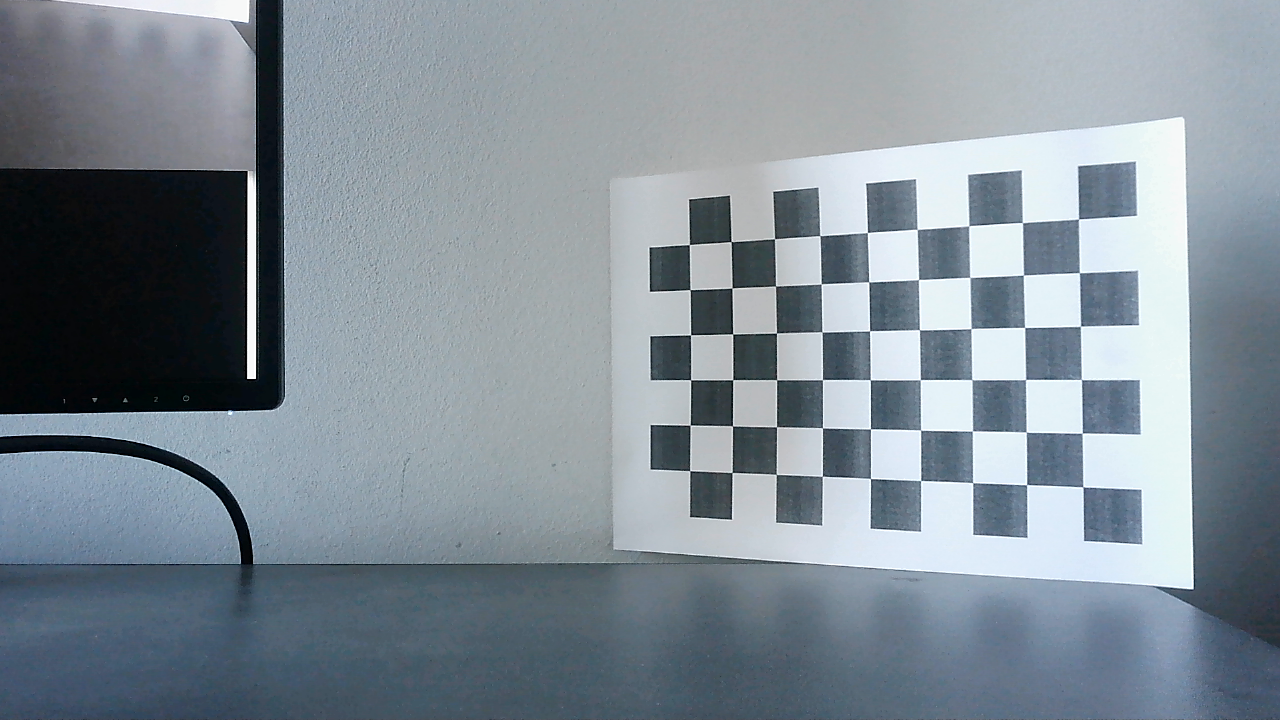

Supplement: Supplementary file 1 [file jimaging-12-00280-s001.zip › Supplementary Materials/first test/Pairs/raw/left/pair_0012_left.png]

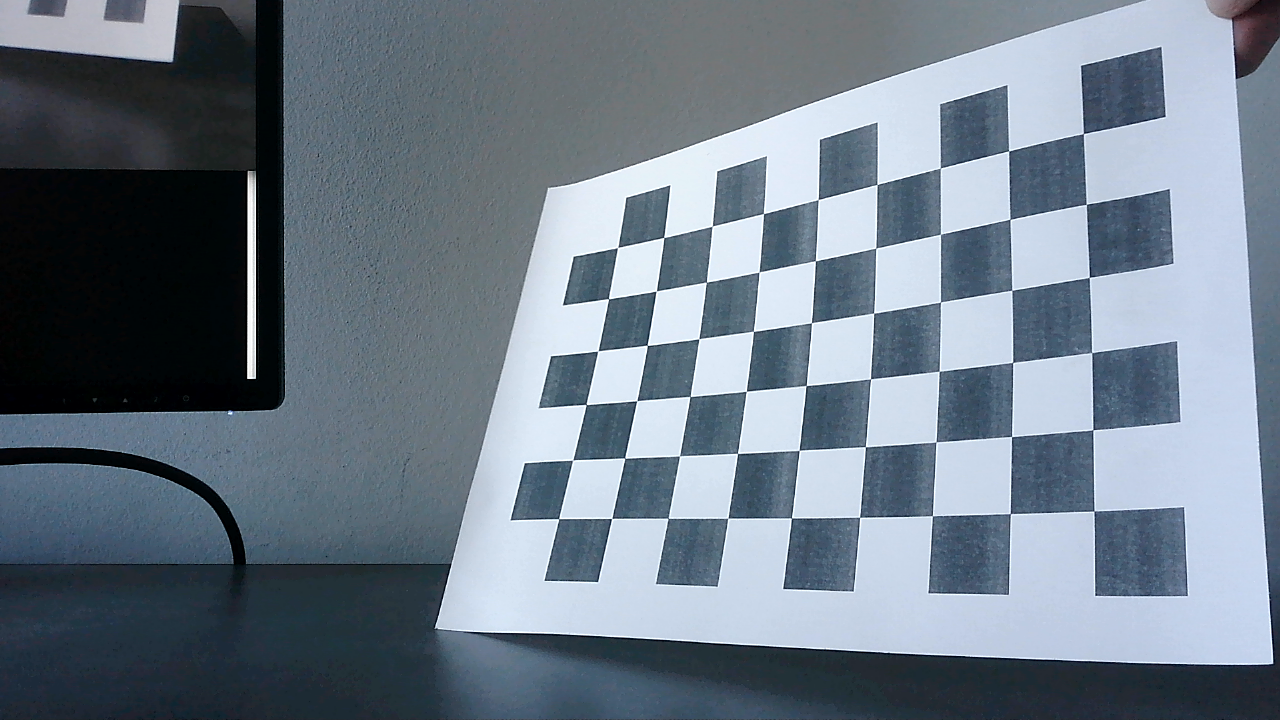

Supplement: Supplementary file 1 [file jimaging-12-00280-s001.zip › Supplementary Materials/first test/Pairs/raw/left/pair_0014_left.png]

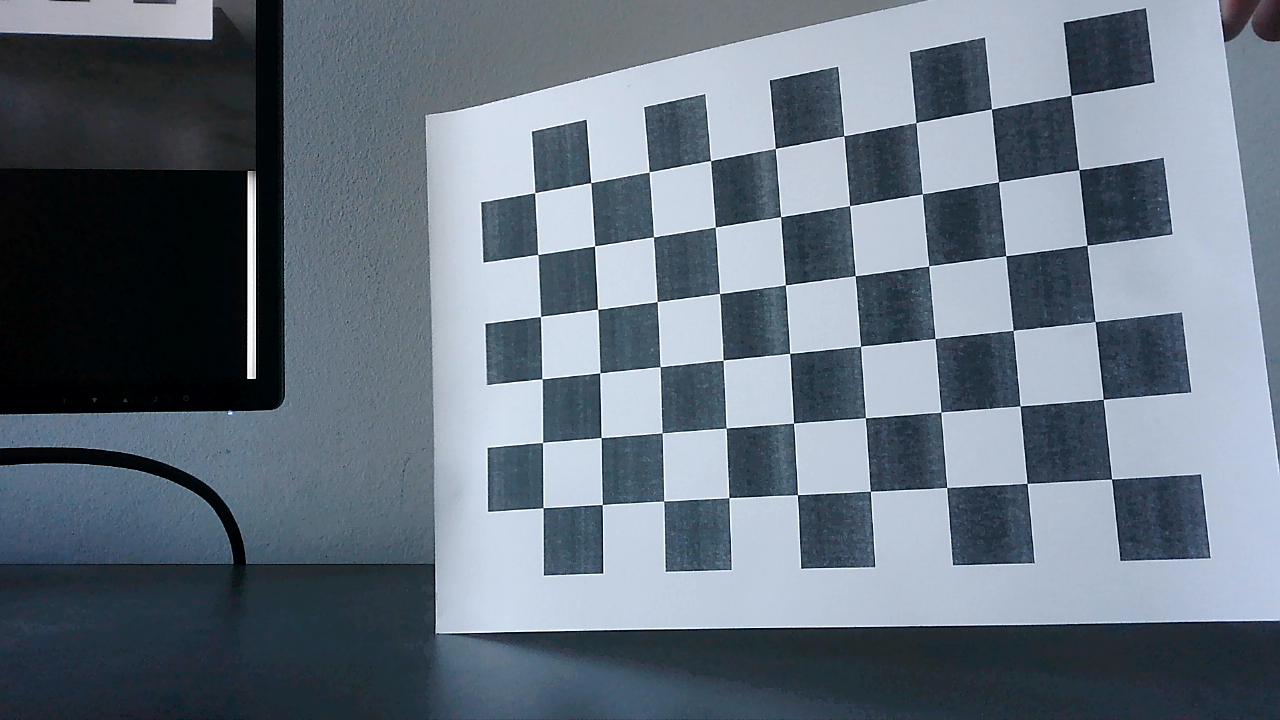

Supplement: Supplementary file 1 [file jimaging-12-00280-s001.zip › Supplementary Materials/first test/Pairs/raw/left/pair_0015_left.png]

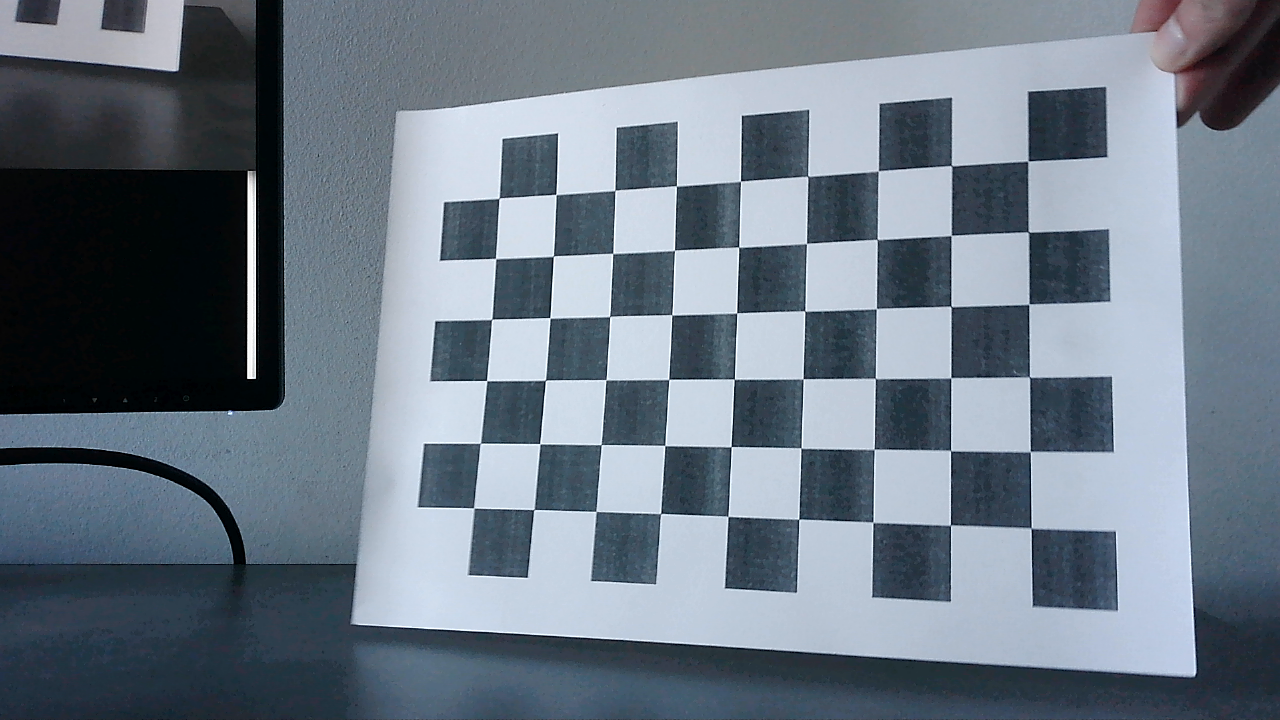

Supplement: Supplementary file 1 [file jimaging-12-00280-s001.zip › Supplementary Materials/first test/Pairs/raw/left/pair_0017_left.png]

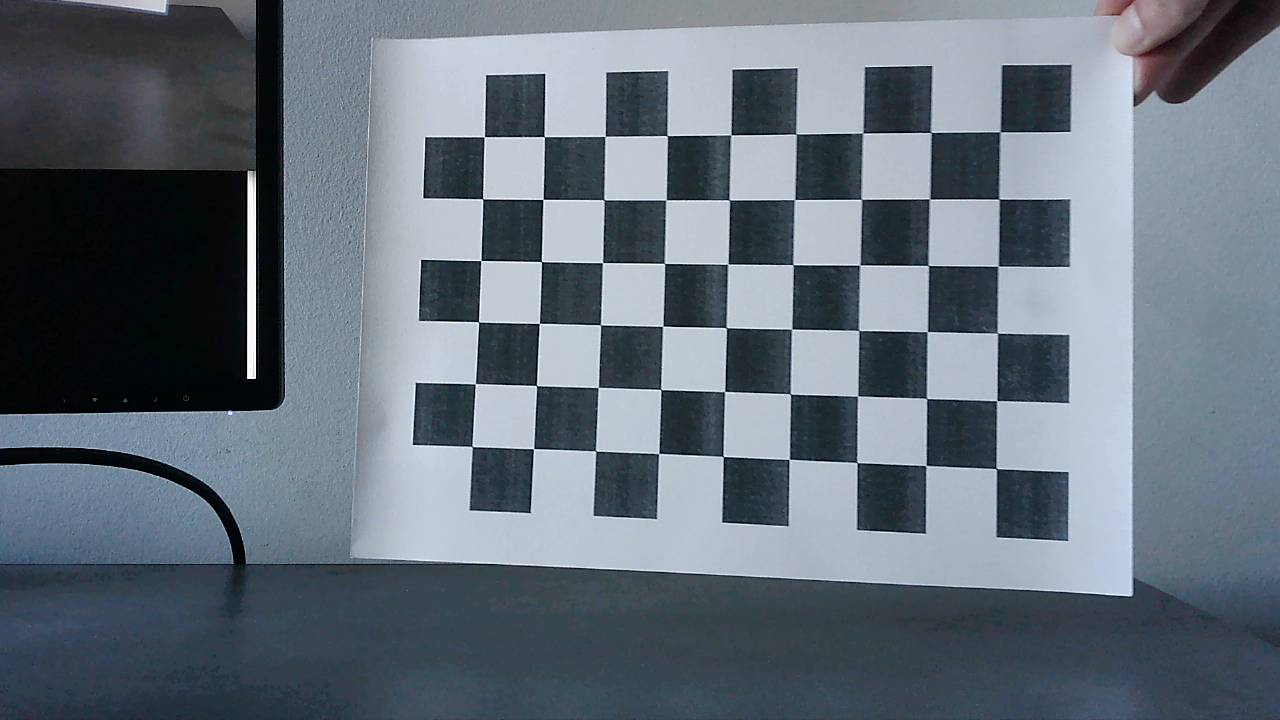

Supplement: Supplementary file 1 [file jimaging-12-00280-s001.zip › Supplementary Materials/first test/Pairs/raw/left/pair_0018_left.png]

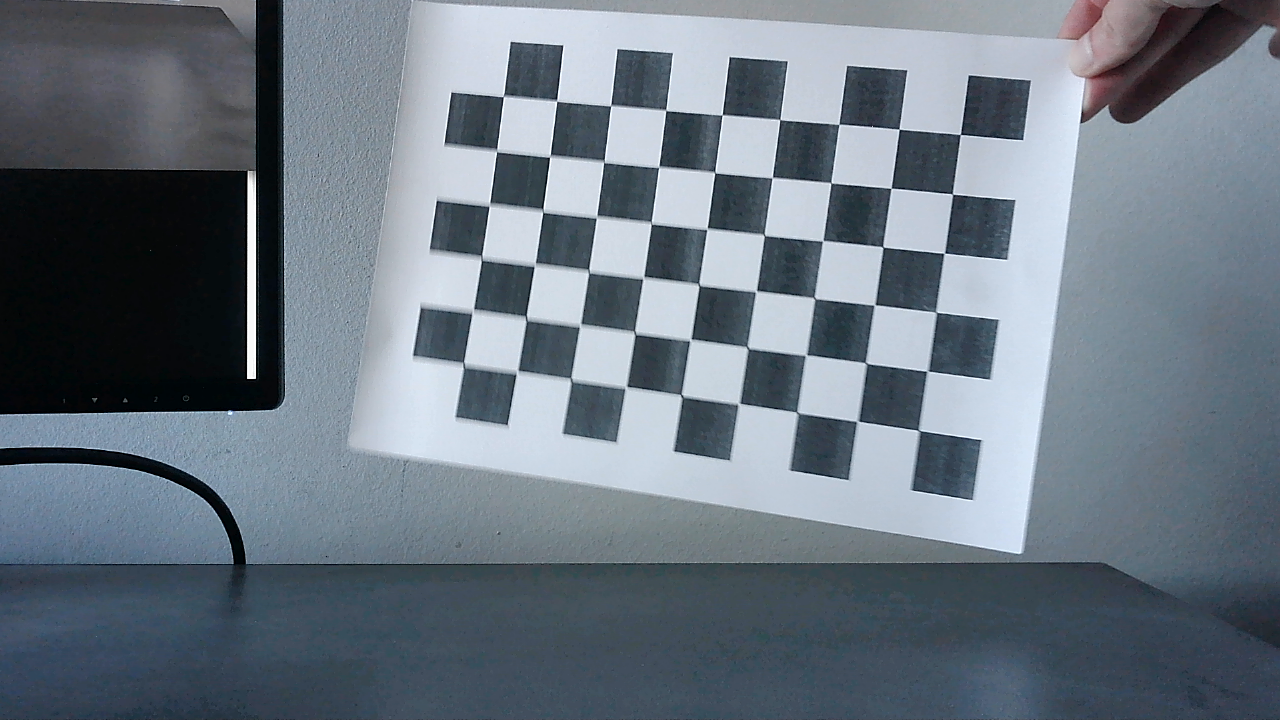

Supplement: Supplementary file 1 [file jimaging-12-00280-s001.zip › Supplementary Materials/first test/Pairs/raw/left/pair_0020_left.png]

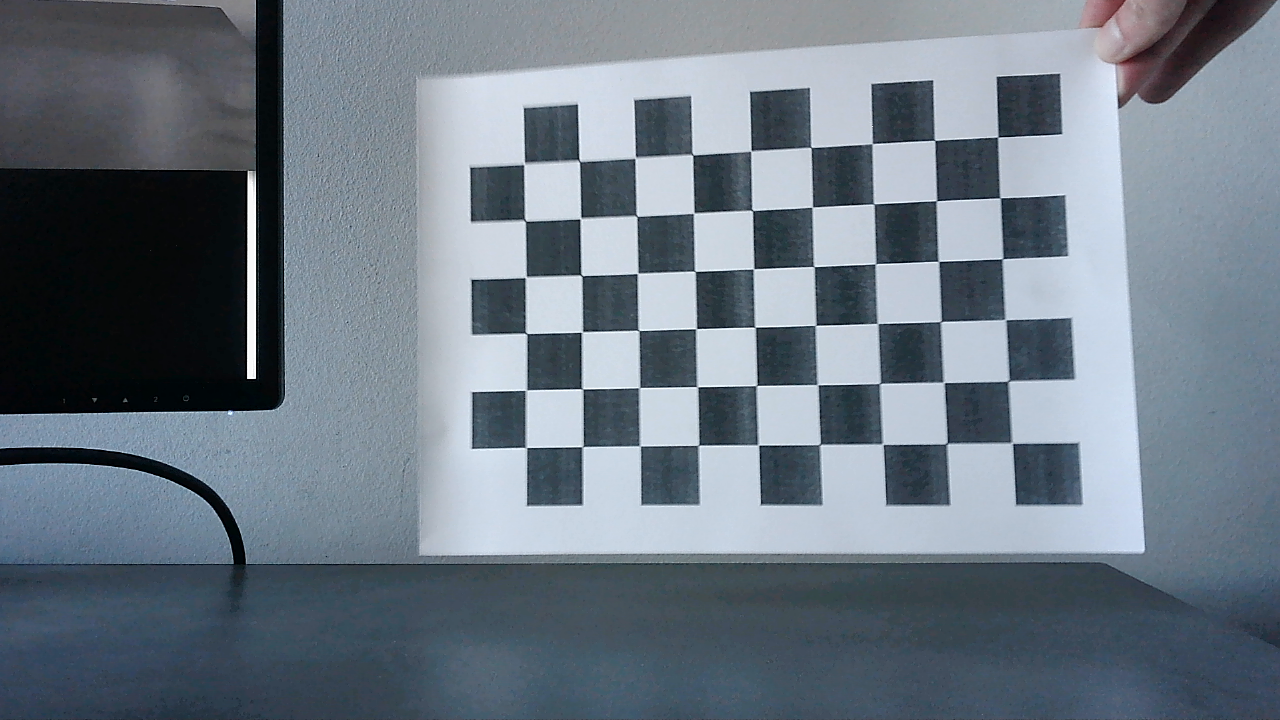

Supplement: Supplementary file 1 [file jimaging-12-00280-s001.zip › Supplementary Materials/first test/Pairs/raw/left/pair_0021_left.png]

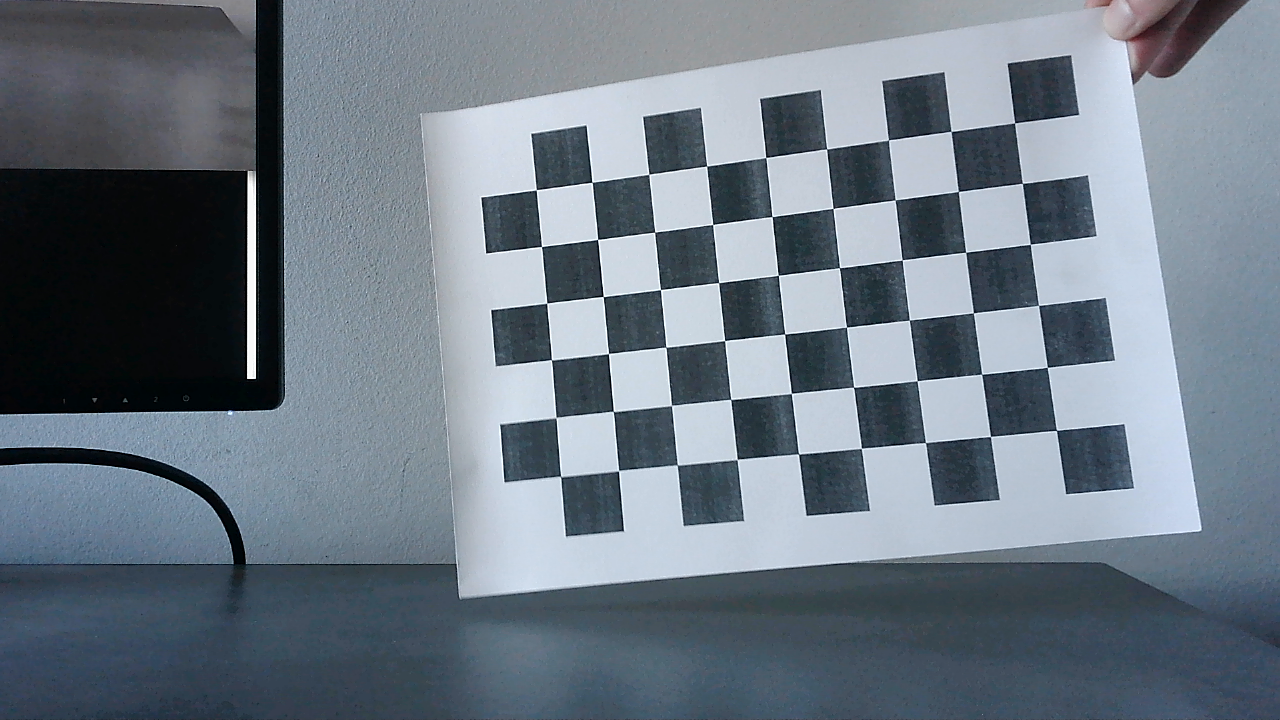

Supplement: Supplementary file 1 [file jimaging-12-00280-s001.zip › Supplementary Materials/first test/Pairs/raw/left/pair_0022_left.png]

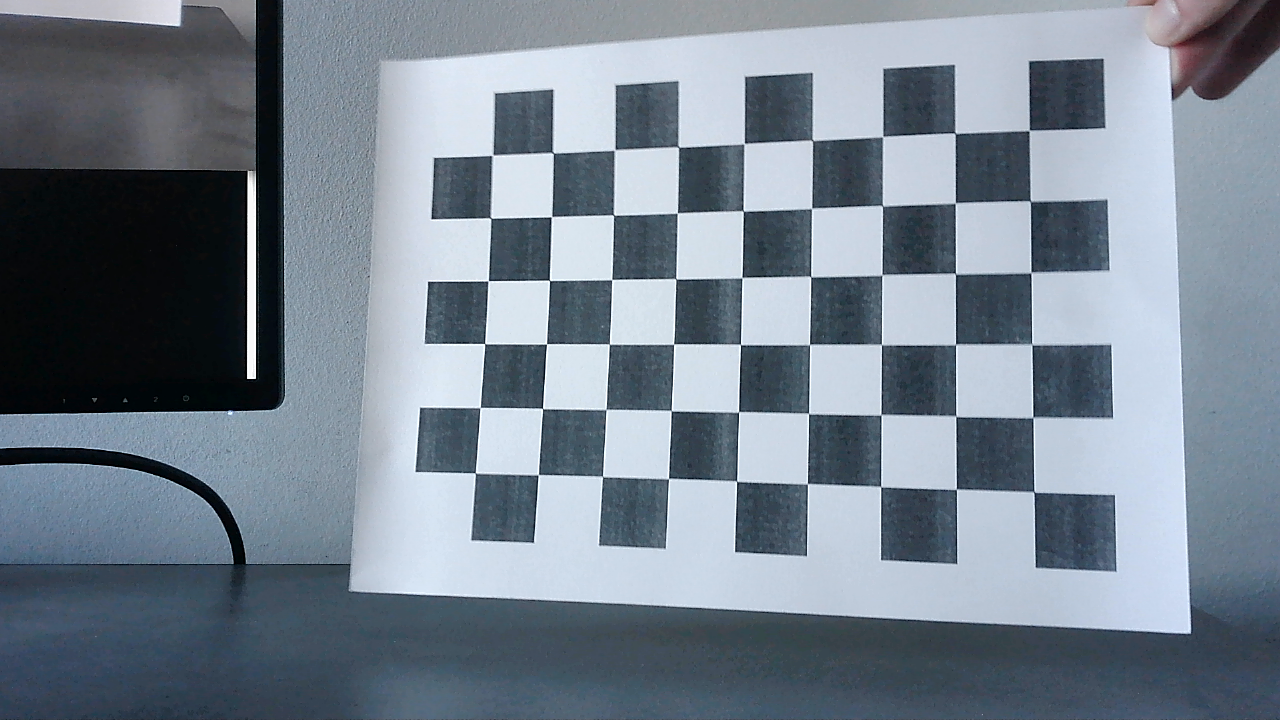

Supplement: Supplementary file 1 [file jimaging-12-00280-s001.zip › Supplementary Materials/first test/Pairs/raw/left/pair_0023_left.png]

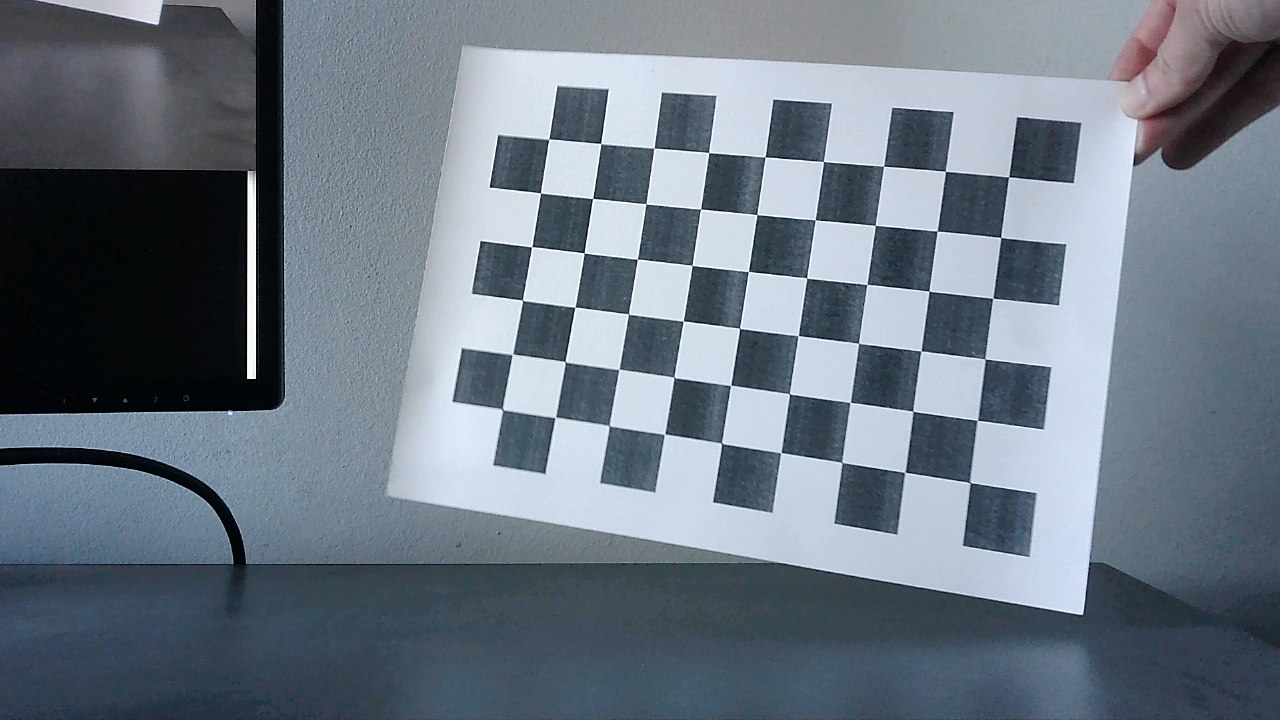

Supplement: Supplementary file 1 [file jimaging-12-00280-s001.zip › Supplementary Materials/first test/Pairs/raw/left/pair_0024_left.png]

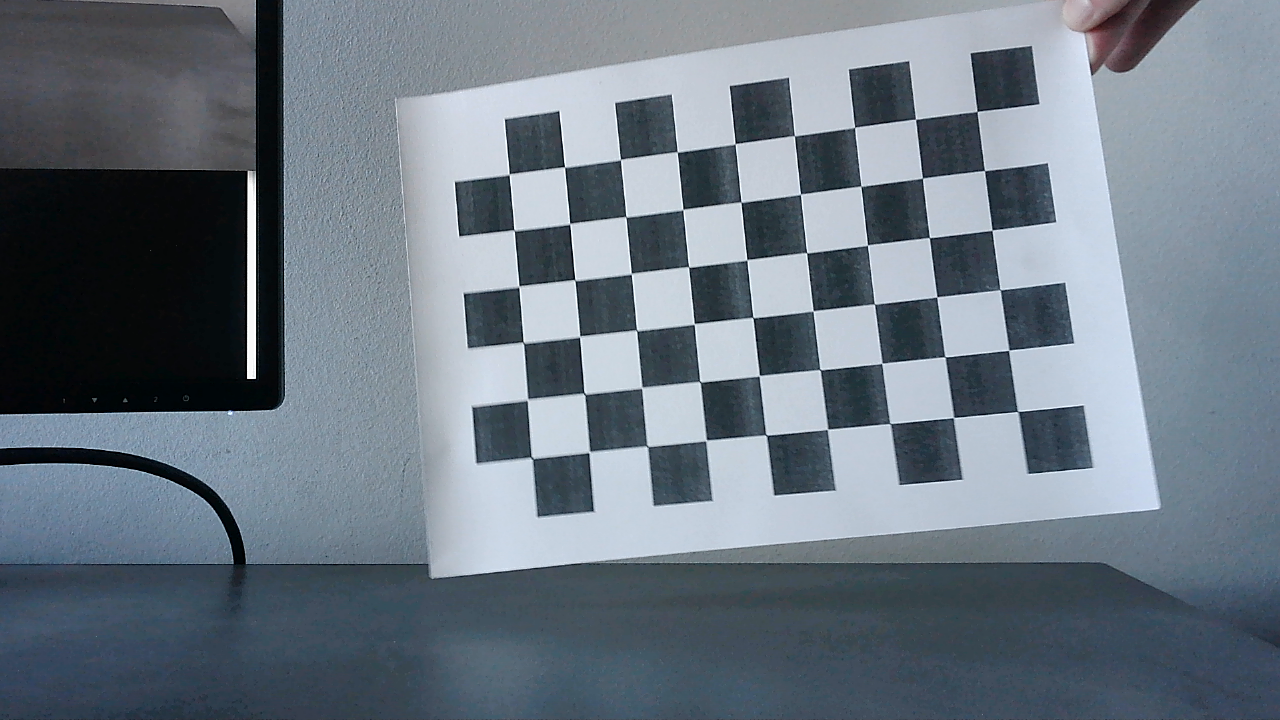

Supplement: Supplementary file 1 [file jimaging-12-00280-s001.zip › Supplementary Materials/first test/Pairs/raw/left/pair_0025_left.png]

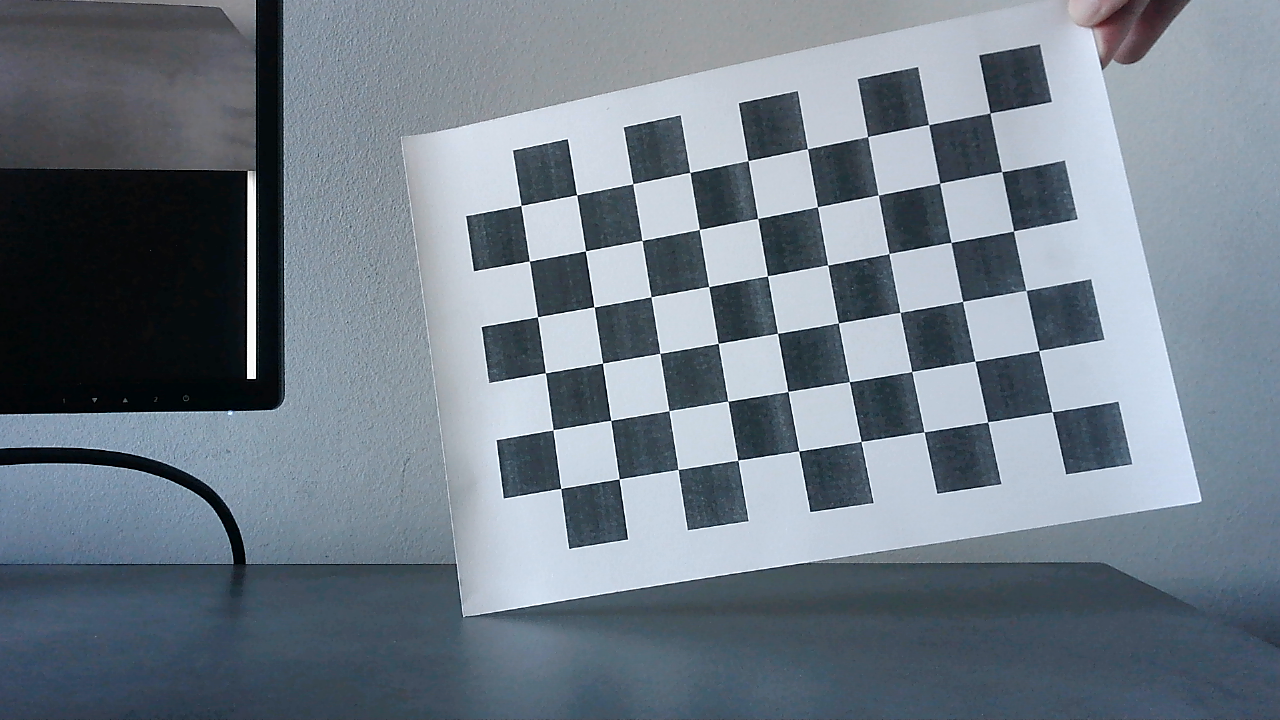

Supplement: Supplementary file 1 [file jimaging-12-00280-s001.zip › Supplementary Materials/first test/Pairs/raw/left/pair_0026_left.png]

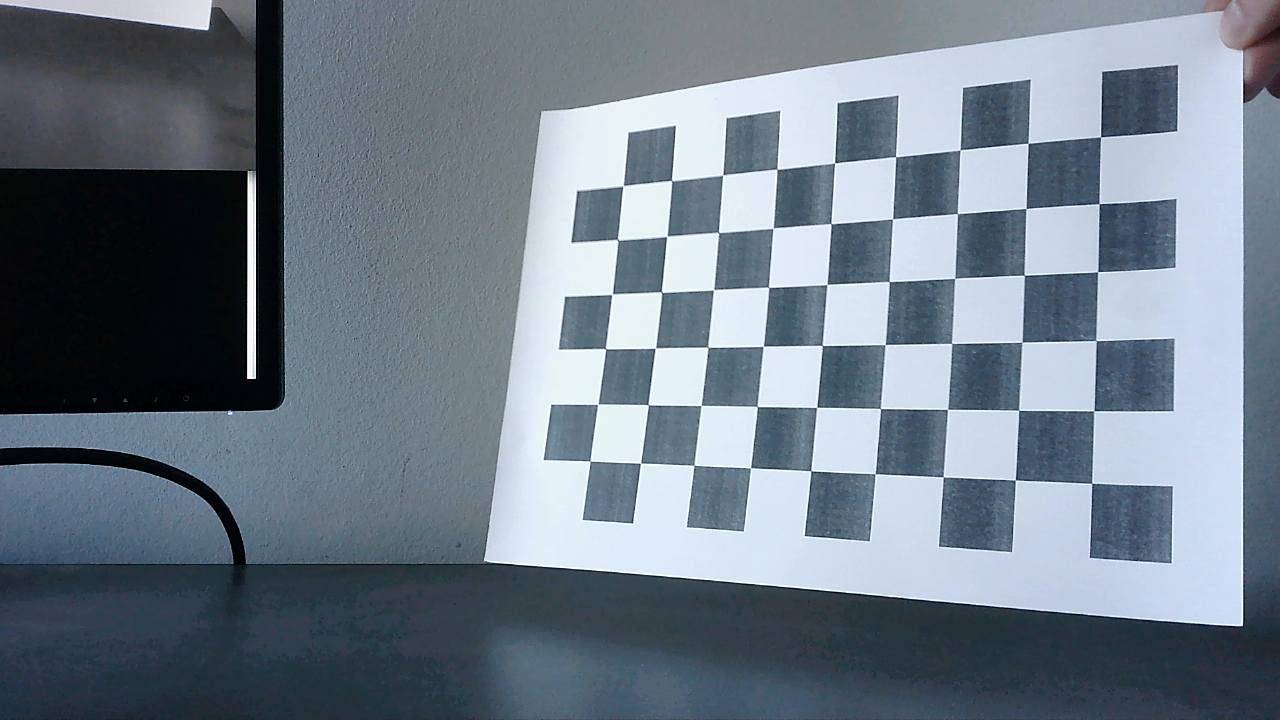

Supplement: Supplementary file 1 [file jimaging-12-00280-s001.zip › Supplementary Materials/first test/Pairs/raw/left/pair_0027_left.png]

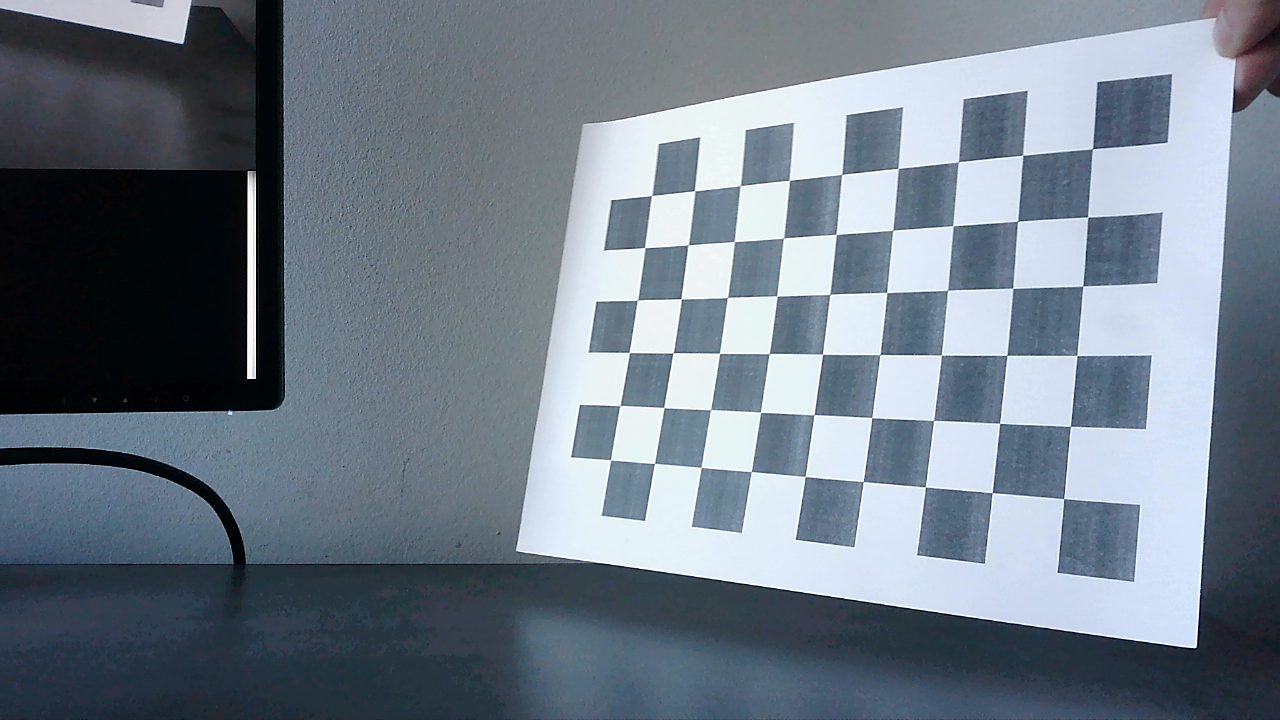

Supplement: Supplementary file 1 [file jimaging-12-00280-s001.zip › Supplementary Materials/first test/Pairs/raw/left/pair_0028_left.png]

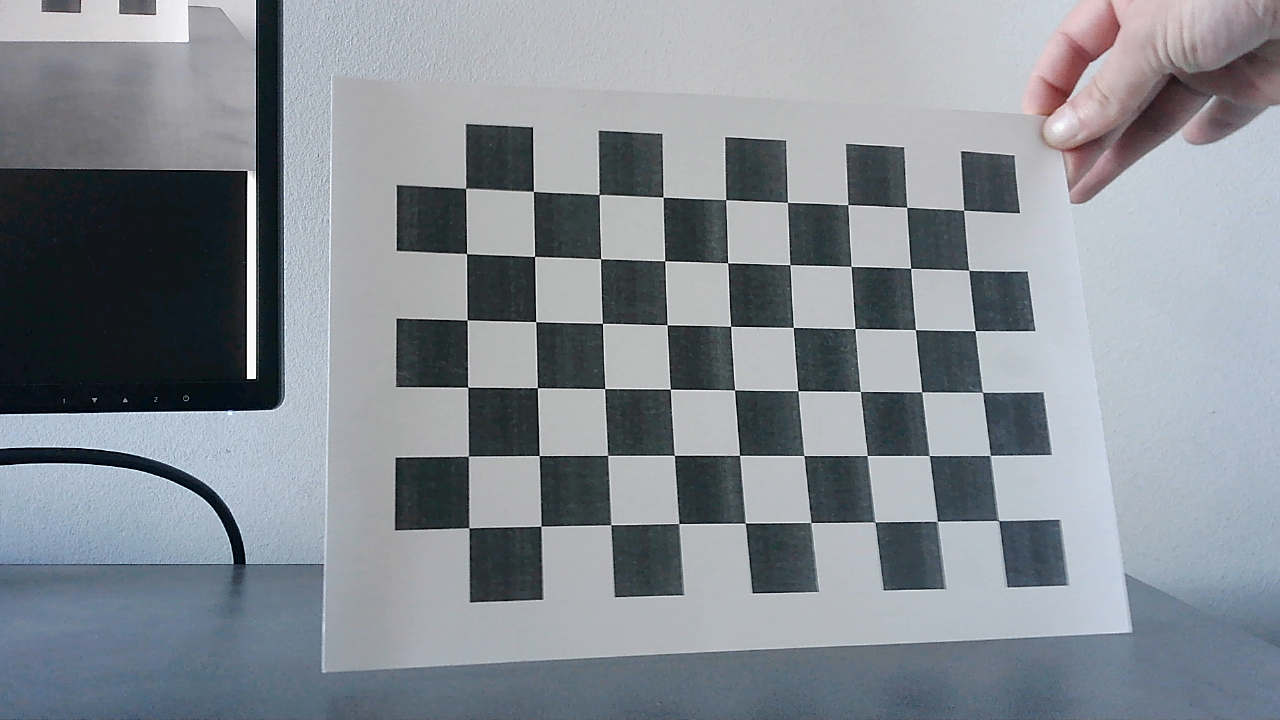

Supplement: Supplementary file 1 [file jimaging-12-00280-s001.zip › Supplementary Materials/first test/Pairs/raw/left/pair_0029_left.png]

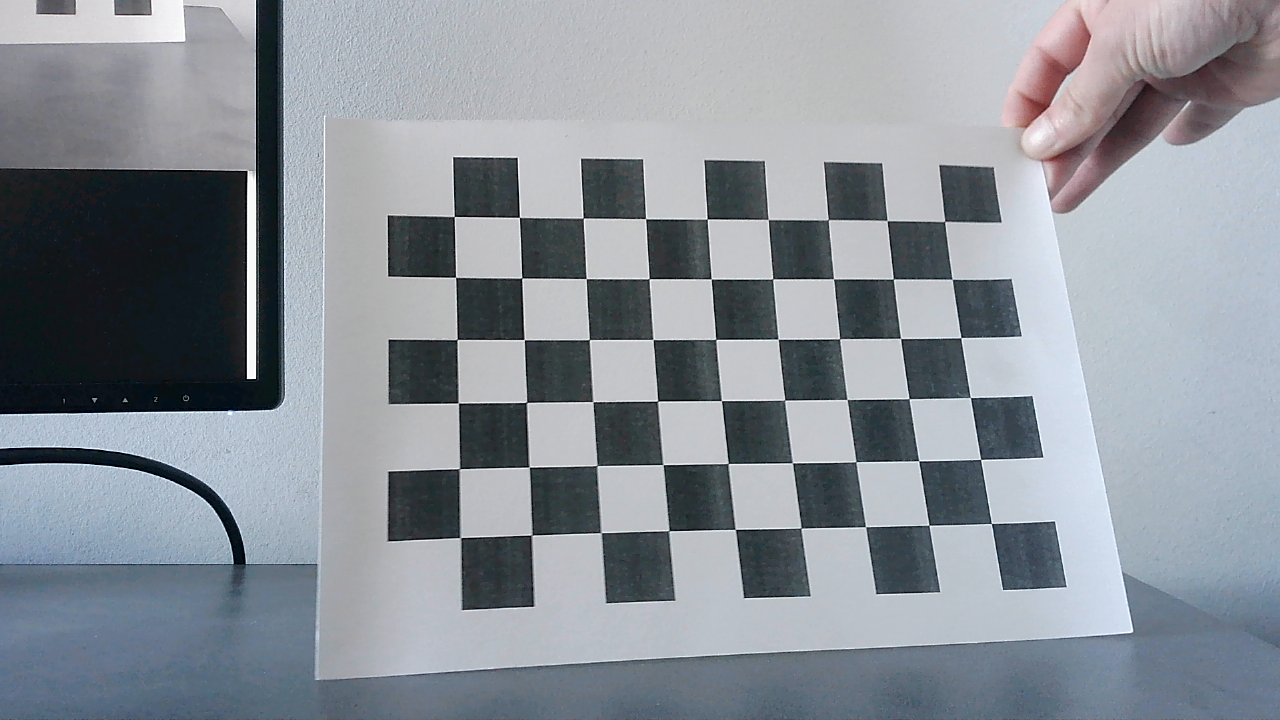

Supplement: Supplementary file 1 [file jimaging-12-00280-s001.zip › Supplementary Materials/first test/Pairs/raw/left/pair_0030_left.png]

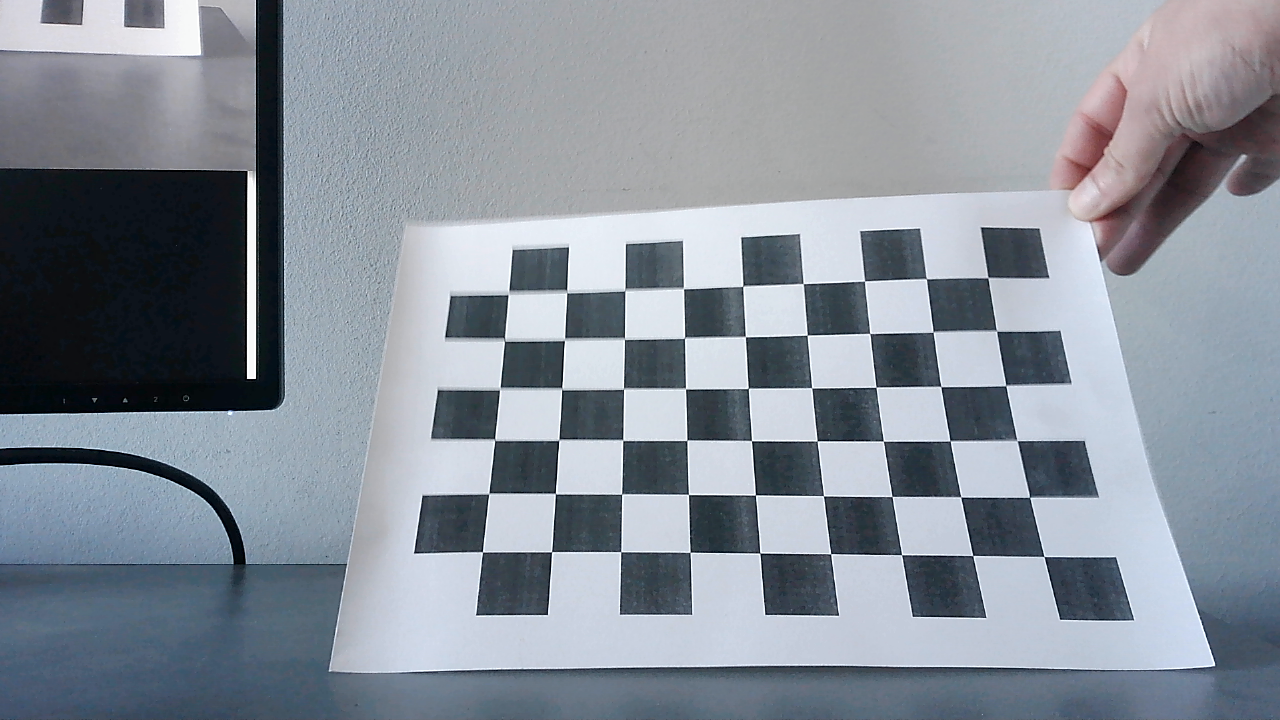

Supplement: Supplementary file 1 [file jimaging-12-00280-s001.zip › Supplementary Materials/first test/Pairs/raw/left/pair_0031_left.png]

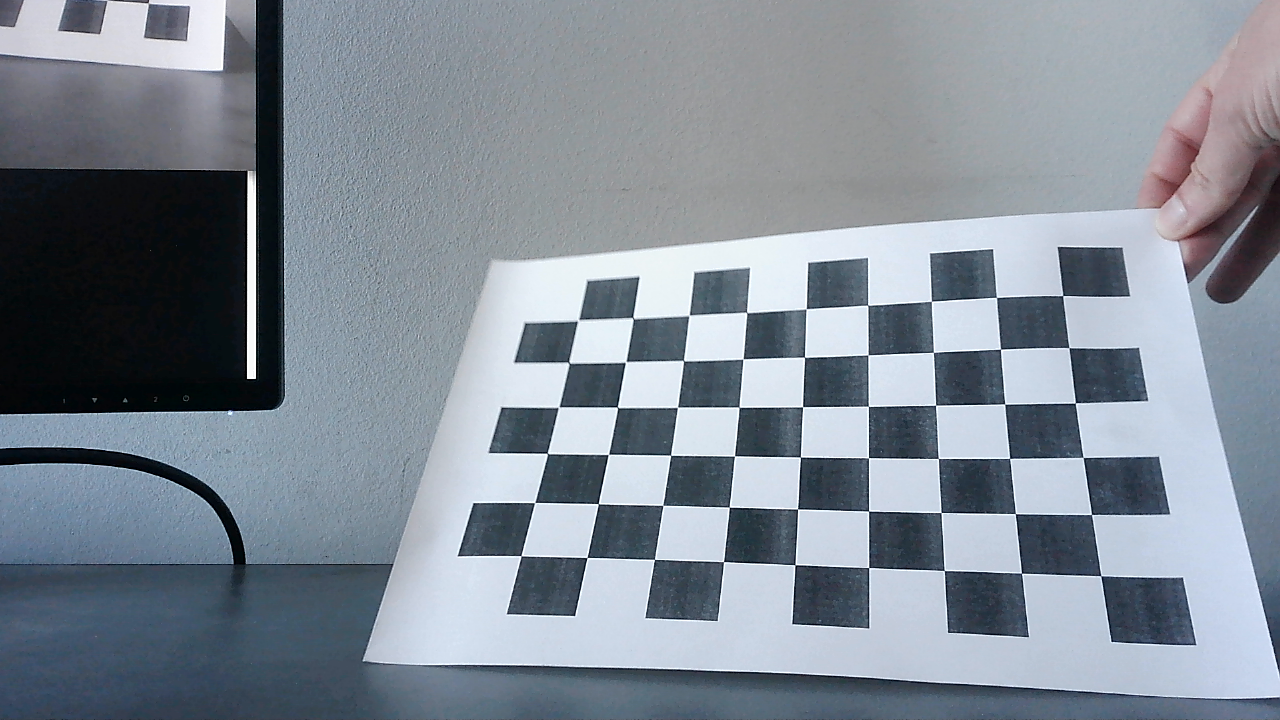

Supplement: Supplementary file 1 [file jimaging-12-00280-s001.zip › Supplementary Materials/first test/Pairs/raw/left/pair_0032_left.png]

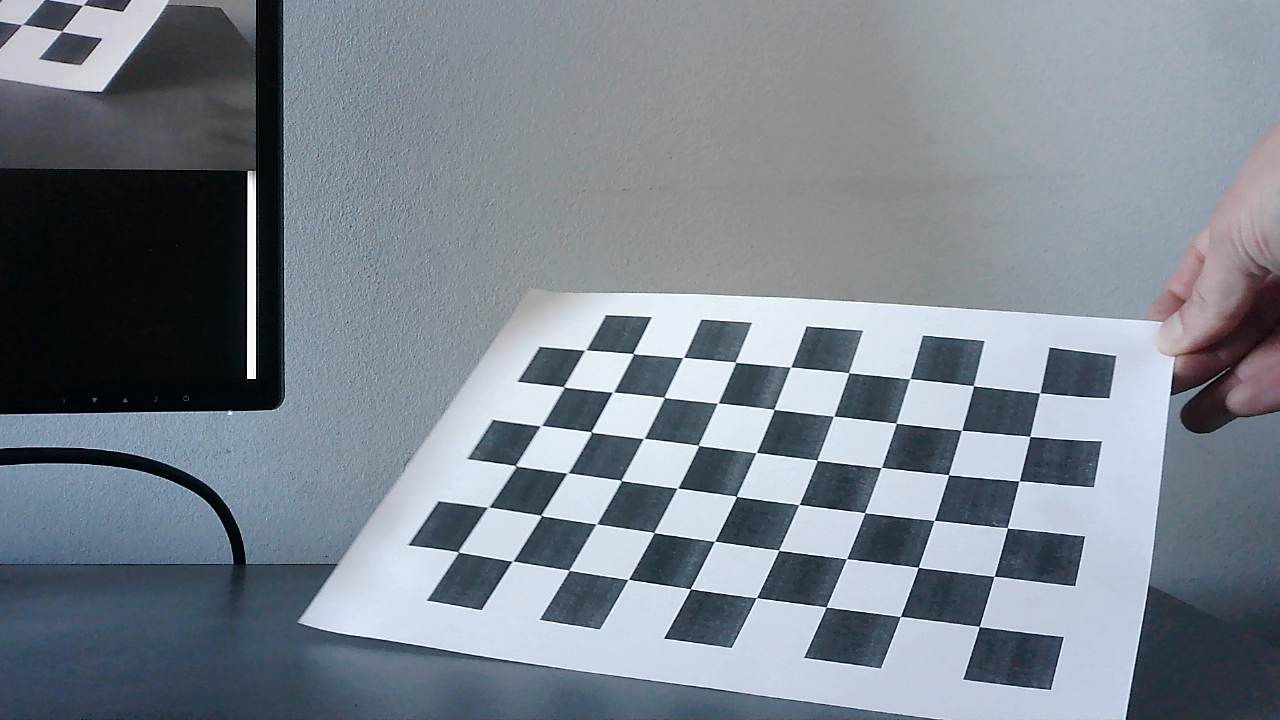

Supplement: Supplementary file 1 [file jimaging-12-00280-s001.zip › Supplementary Materials/first test/Pairs/raw/left/pair_0033_left.png]

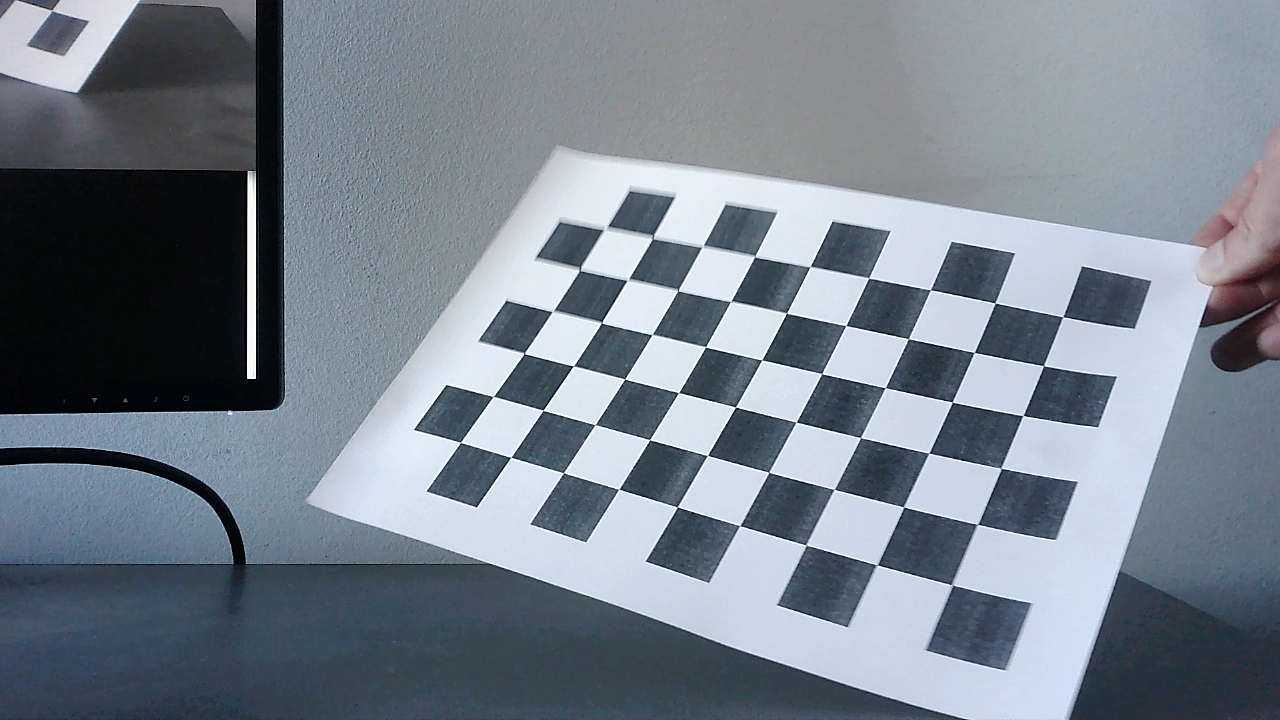

Supplement: Supplementary file 1 [file jimaging-12-00280-s001.zip › Supplementary Materials/first test/Pairs/raw/left/pair_0034_left.png]

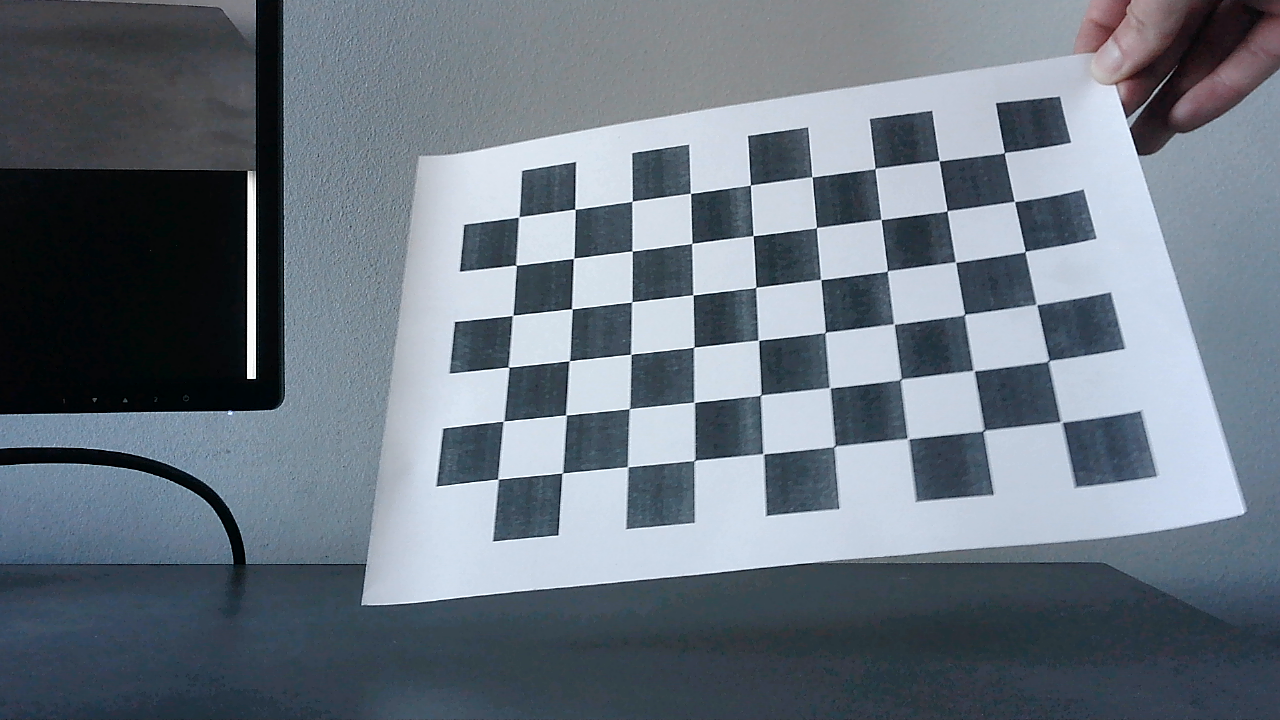

Supplement: Supplementary file 1 [file jimaging-12-00280-s001.zip › Supplementary Materials/first test/Pairs/raw/left/pair_0035_left.png]

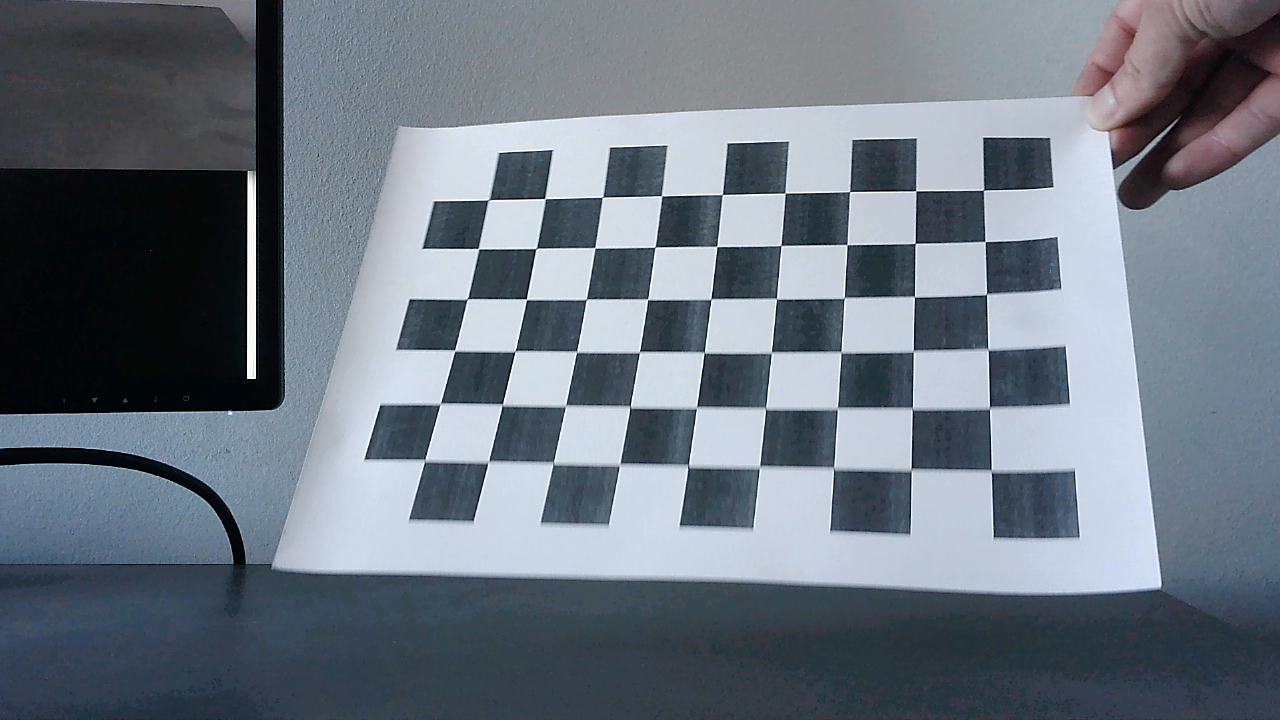

Supplement: Supplementary file 1 [file jimaging-12-00280-s001.zip › Supplementary Materials/first test/Pairs/raw/left/pair_0036_left.png]

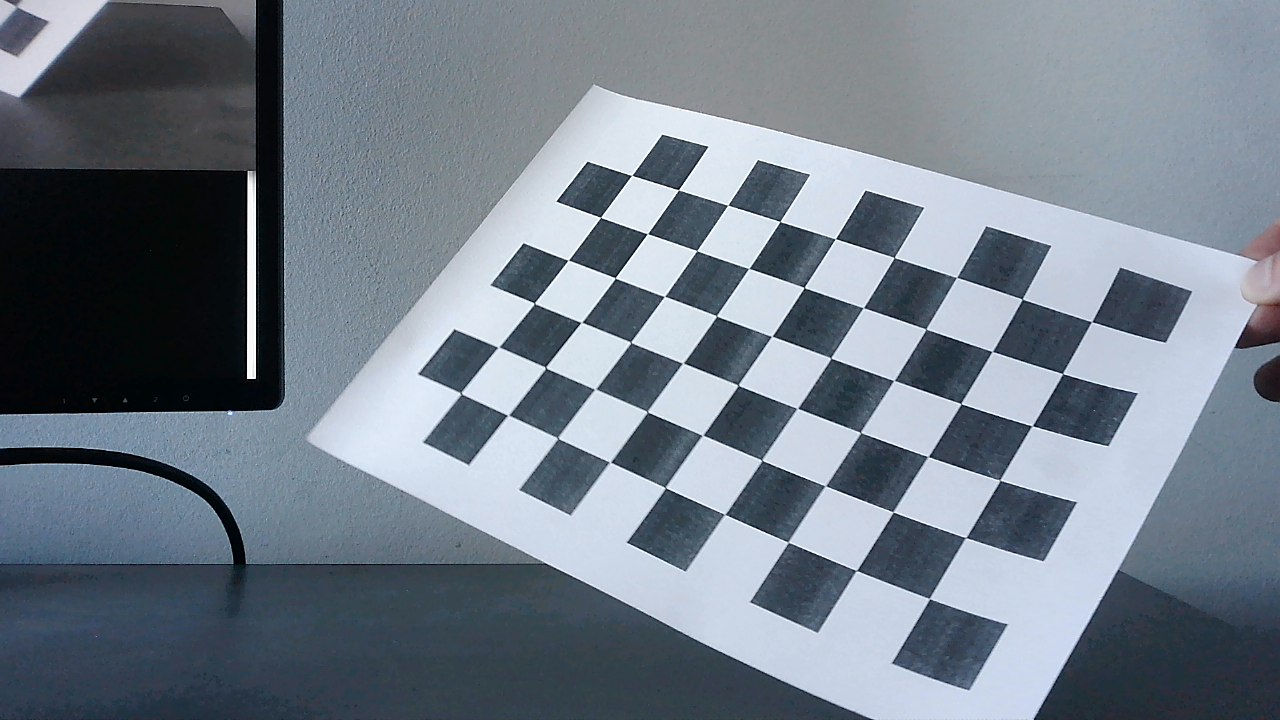

Supplement: Supplementary file 1 [file jimaging-12-00280-s001.zip › Supplementary Materials/first test/Pairs/raw/left/pair_0037_left.png]

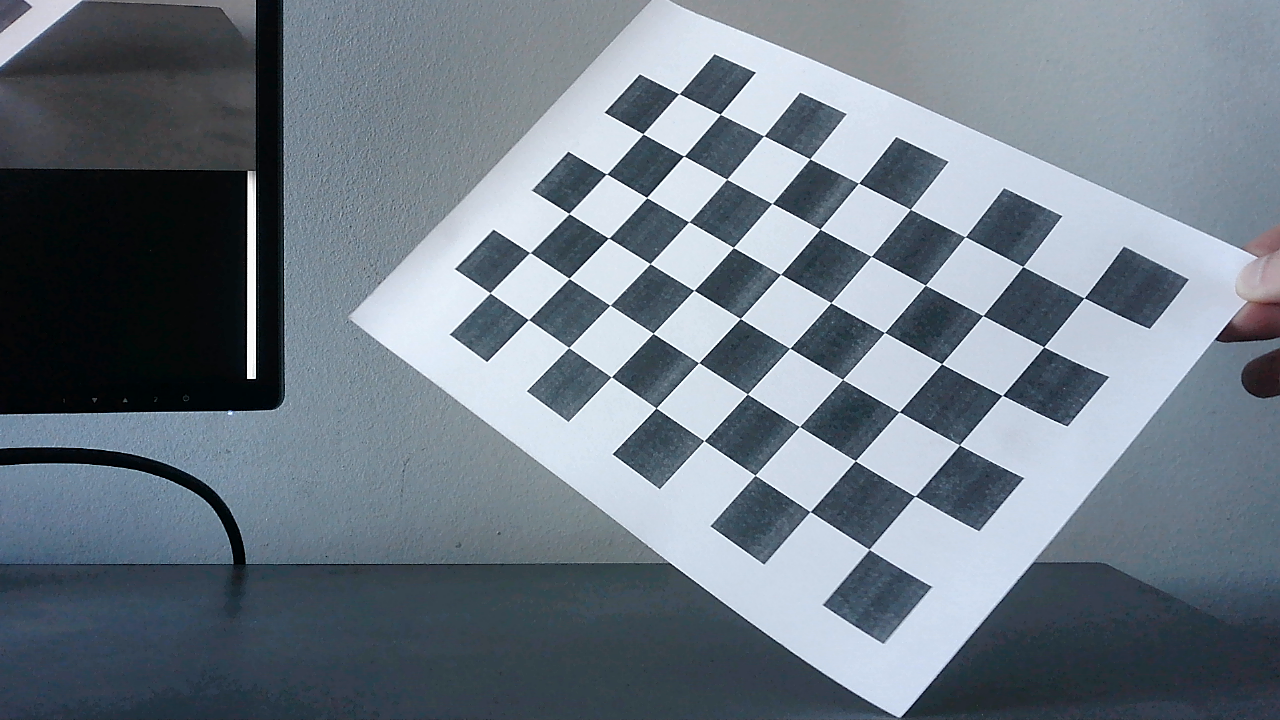

Supplement: Supplementary file 1 [file jimaging-12-00280-s001.zip › Supplementary Materials/first test/Pairs/raw/left/pair_0038_left.png]

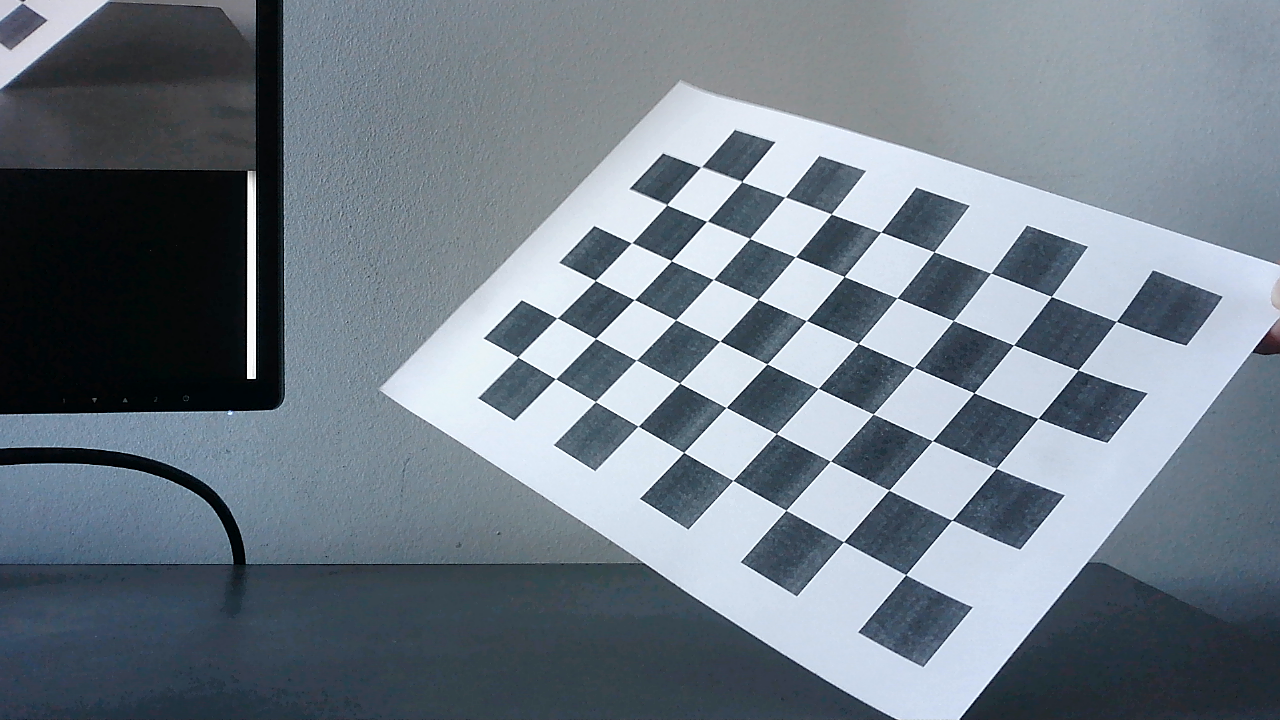

Supplement: Supplementary file 1 [file jimaging-12-00280-s001.zip › Supplementary Materials/first test/Pairs/raw/left/pair_0039_left.png]

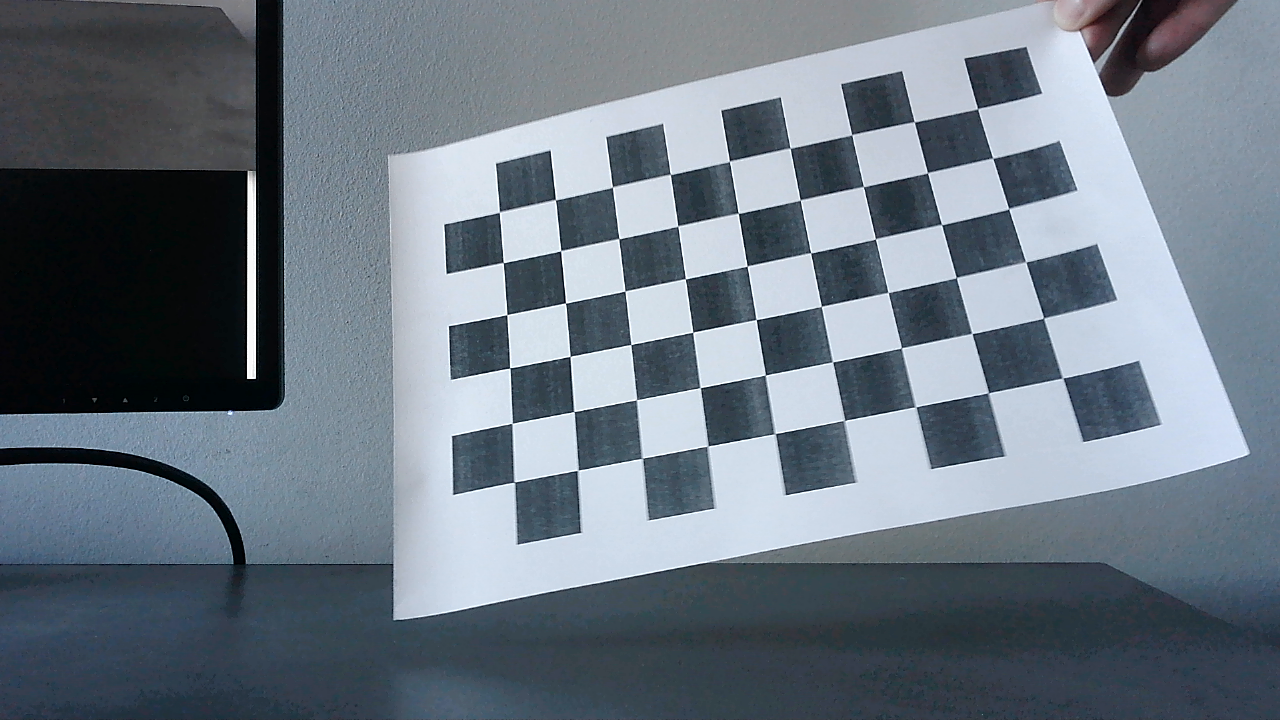

Supplement: Supplementary file 1 [file jimaging-12-00280-s001.zip › Supplementary Materials/first test/Pairs/raw/left/pair_0040_left.png]

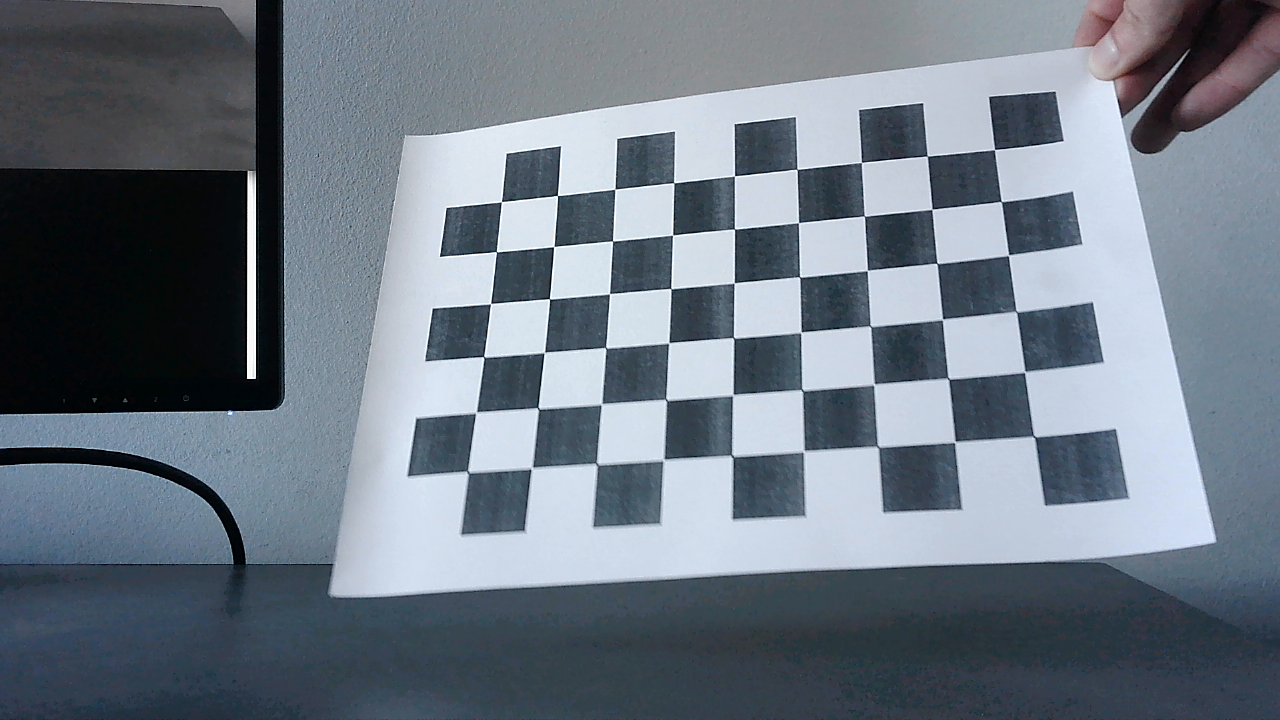

Supplement: Supplementary file 1 [file jimaging-12-00280-s001.zip › Supplementary Materials/first test/Pairs/raw/left/pair_0041_left.png]

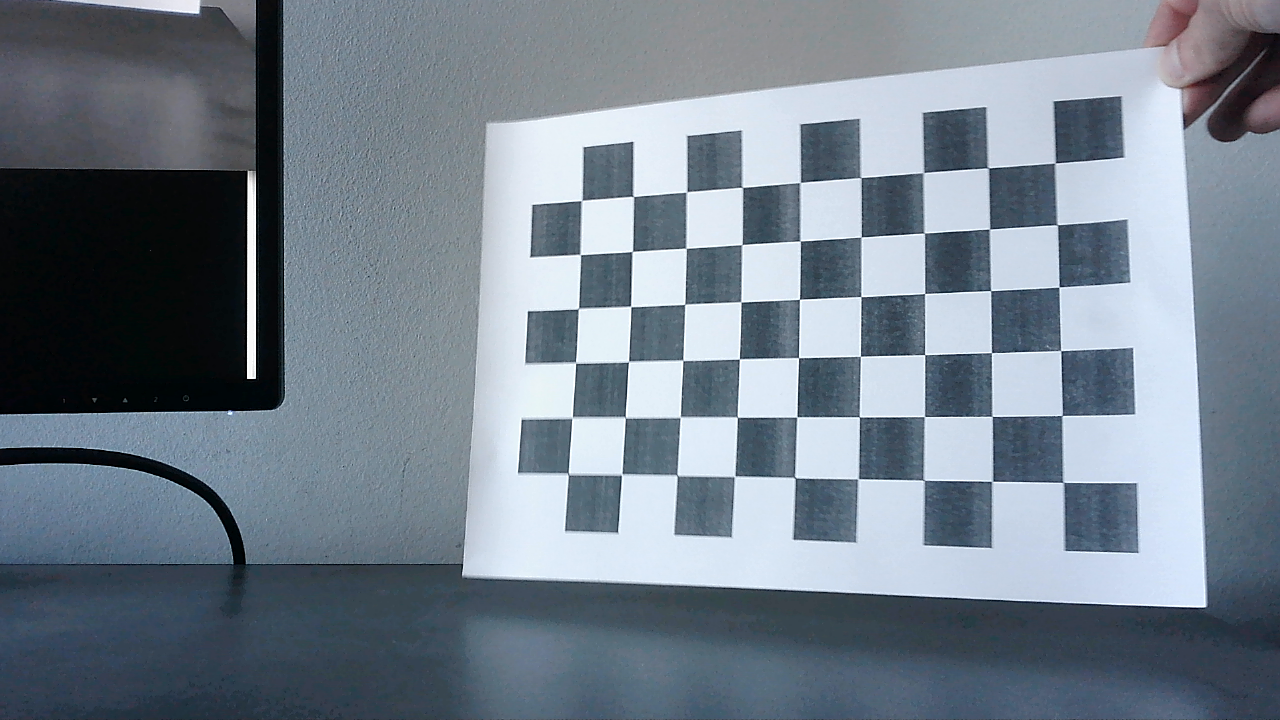

Supplement: Supplementary file 1 [file jimaging-12-00280-s001.zip › Supplementary Materials/first test/Pairs/raw/left/pair_0042_left.png]

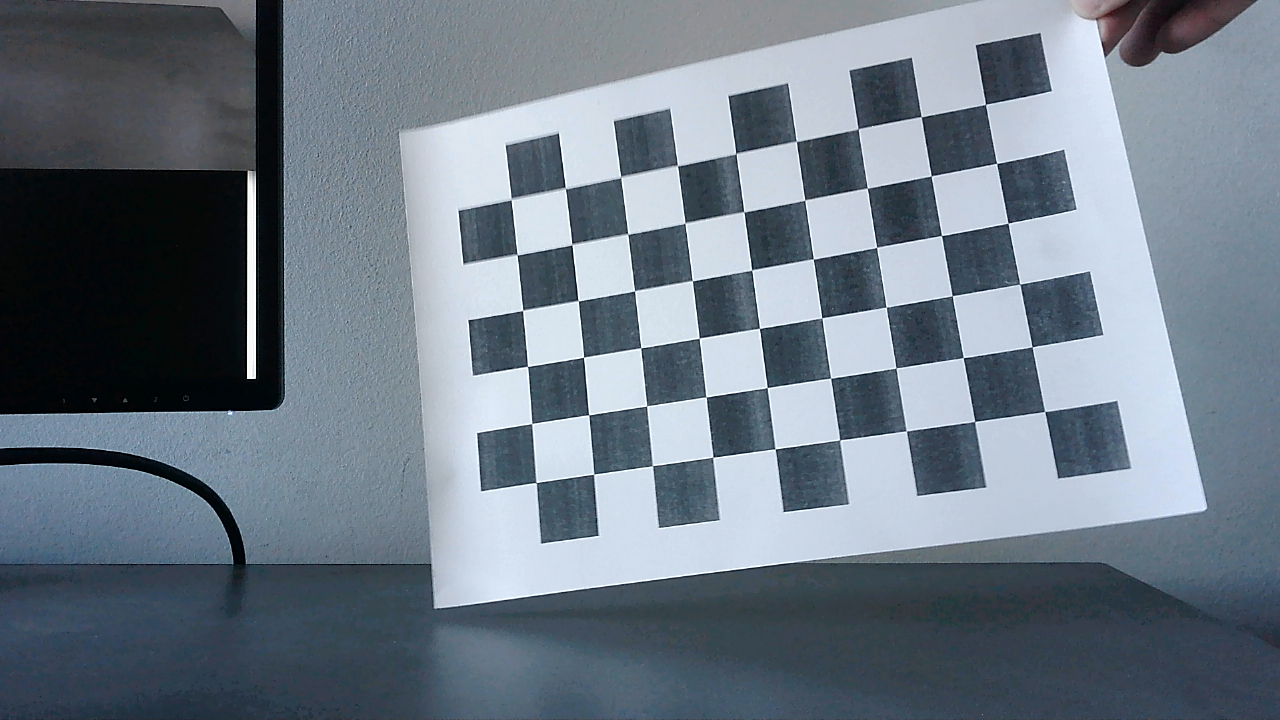

Supplement: Supplementary file 1 [file jimaging-12-00280-s001.zip › Supplementary Materials/first test/Pairs/raw/left/pair_0043_left.png]

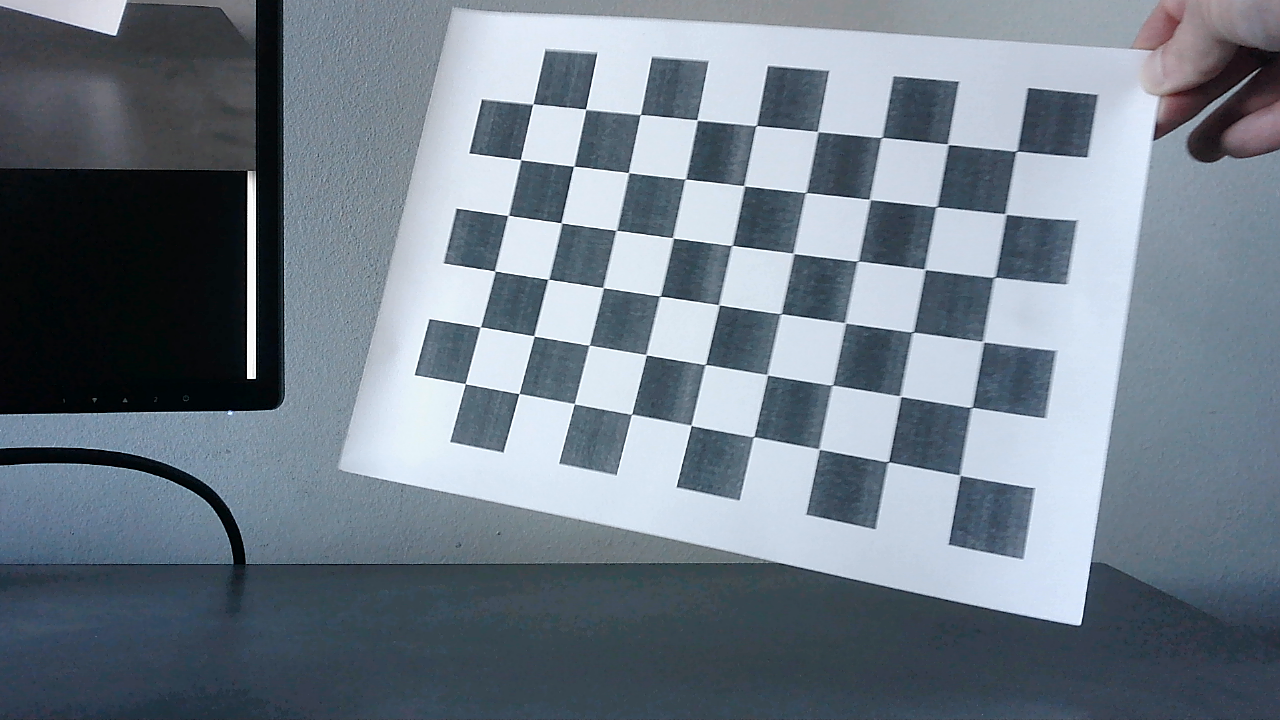

Supplement: Supplementary file 1 [file jimaging-12-00280-s001.zip › Supplementary Materials/first test/Pairs/raw/left/pair_0044_left.png]

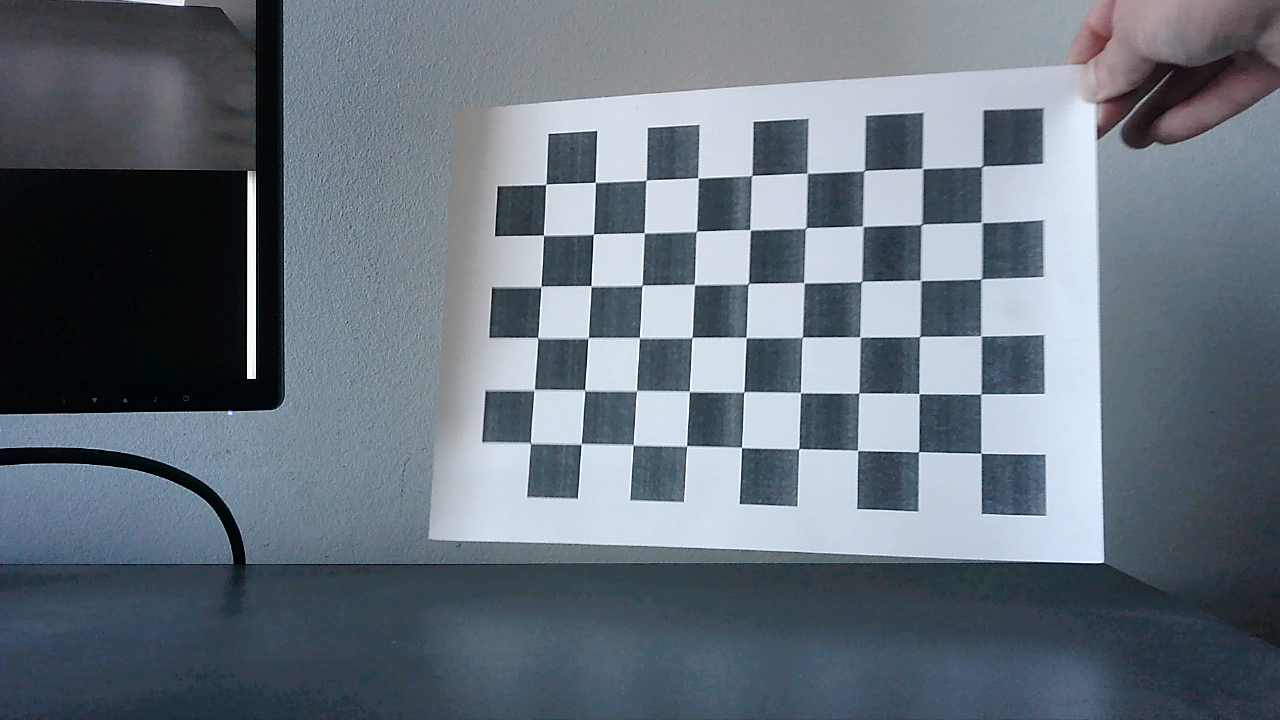

Supplement: Supplementary file 1 [file jimaging-12-00280-s001.zip › Supplementary Materials/first test/Pairs/raw/left/pair_0045_left.png]

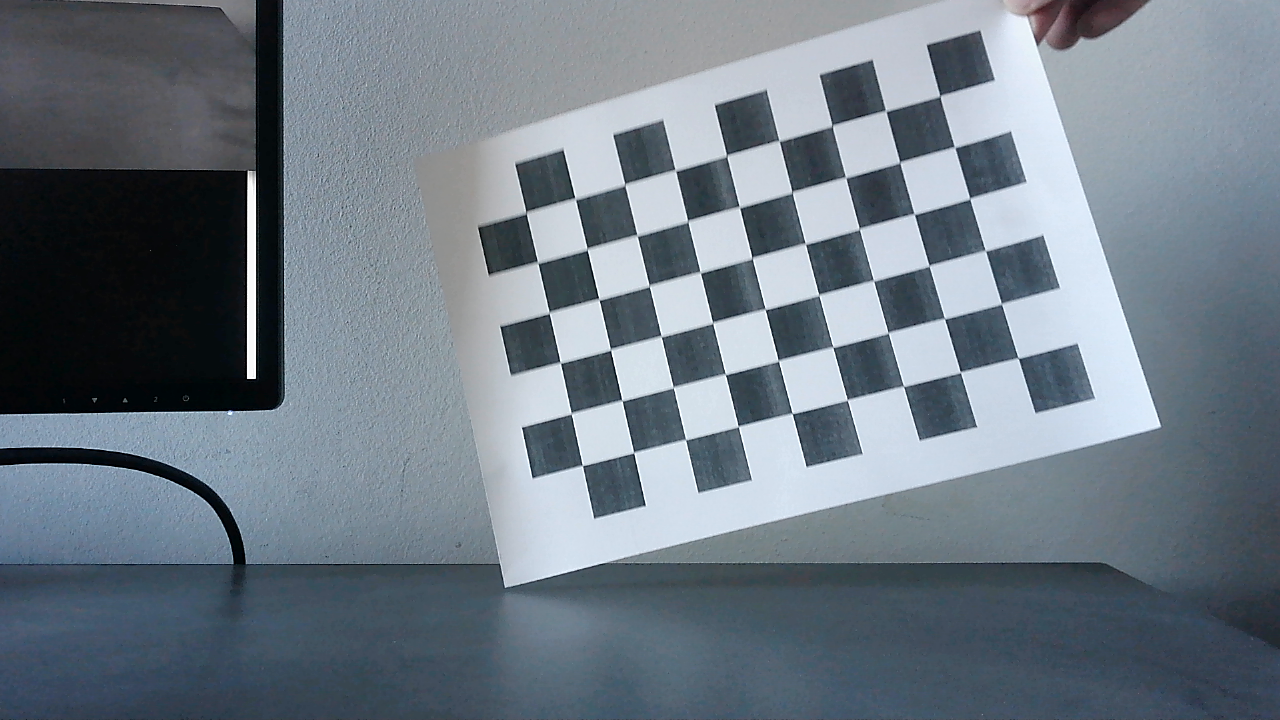

Supplement: Supplementary file 1 [file jimaging-12-00280-s001.zip › Supplementary Materials/first test/Pairs/raw/left/pair_0046_left.png]

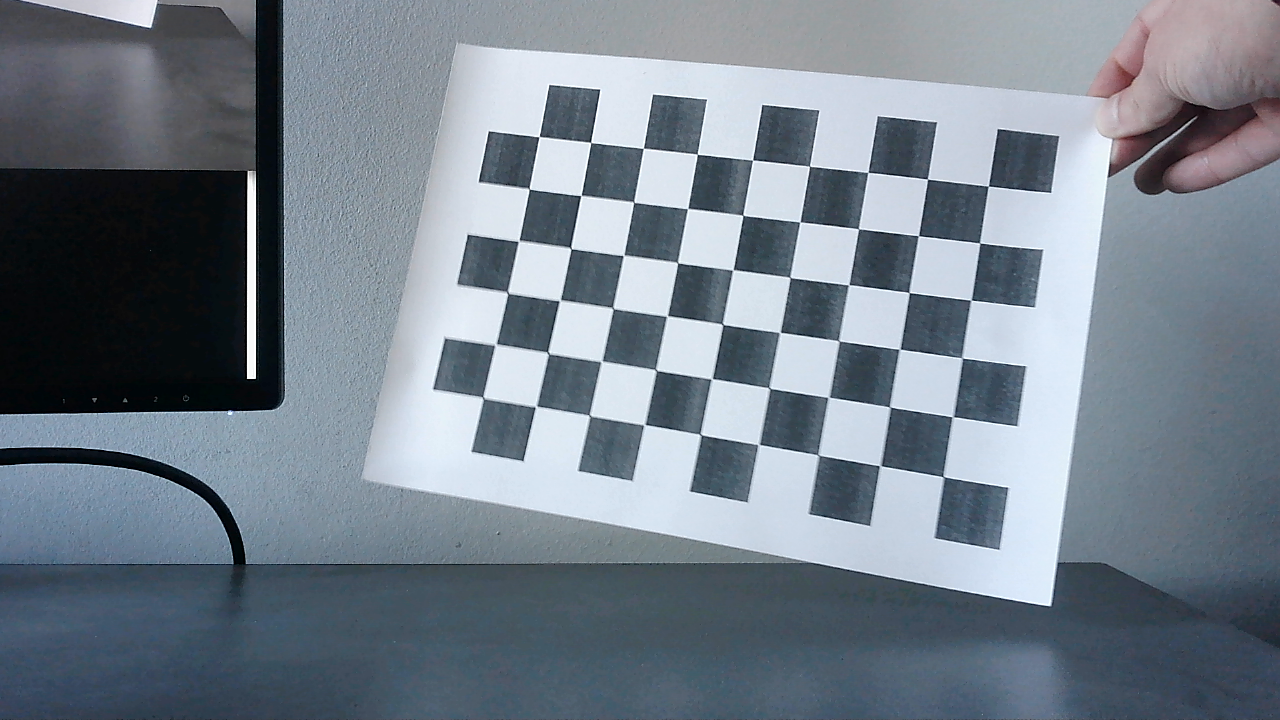

Supplement: Supplementary file 1 [file jimaging-12-00280-s001.zip › Supplementary Materials/first test/Pairs/raw/left/pair_0047_left.png]

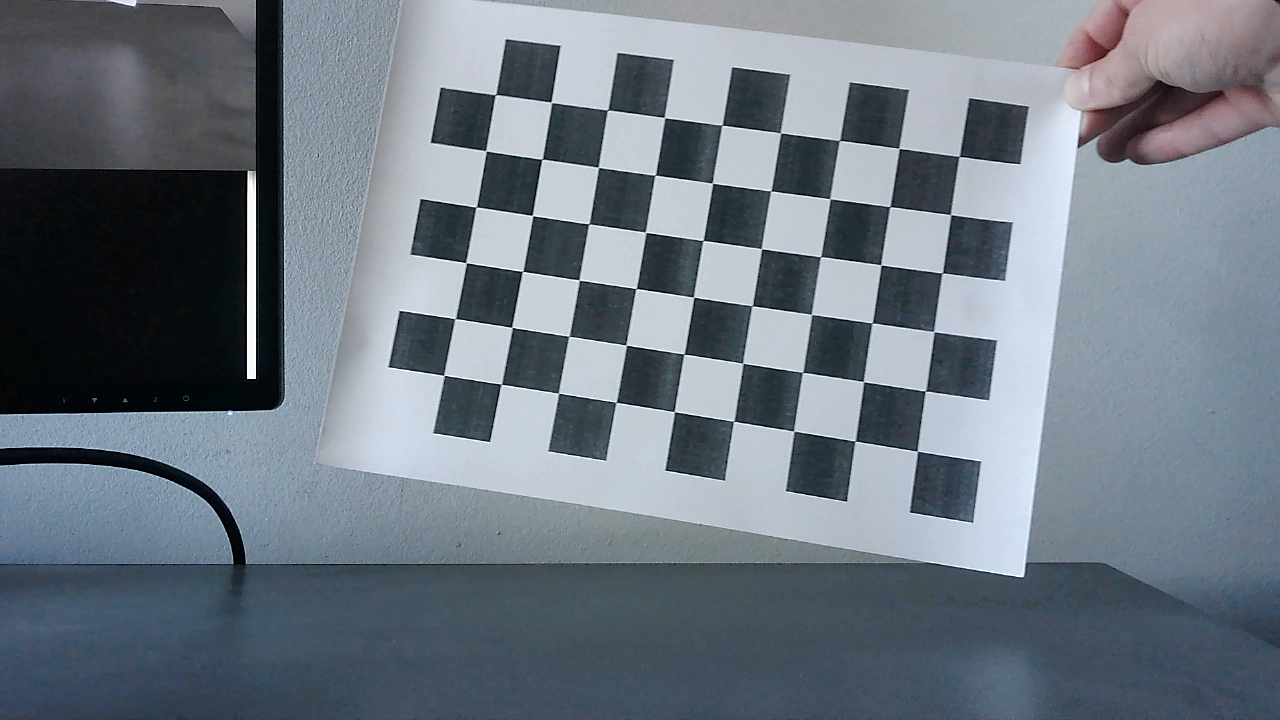

Supplement: Supplementary file 1 [file jimaging-12-00280-s001.zip › Supplementary Materials/first test/Pairs/raw/left/pair_0048_left.png]

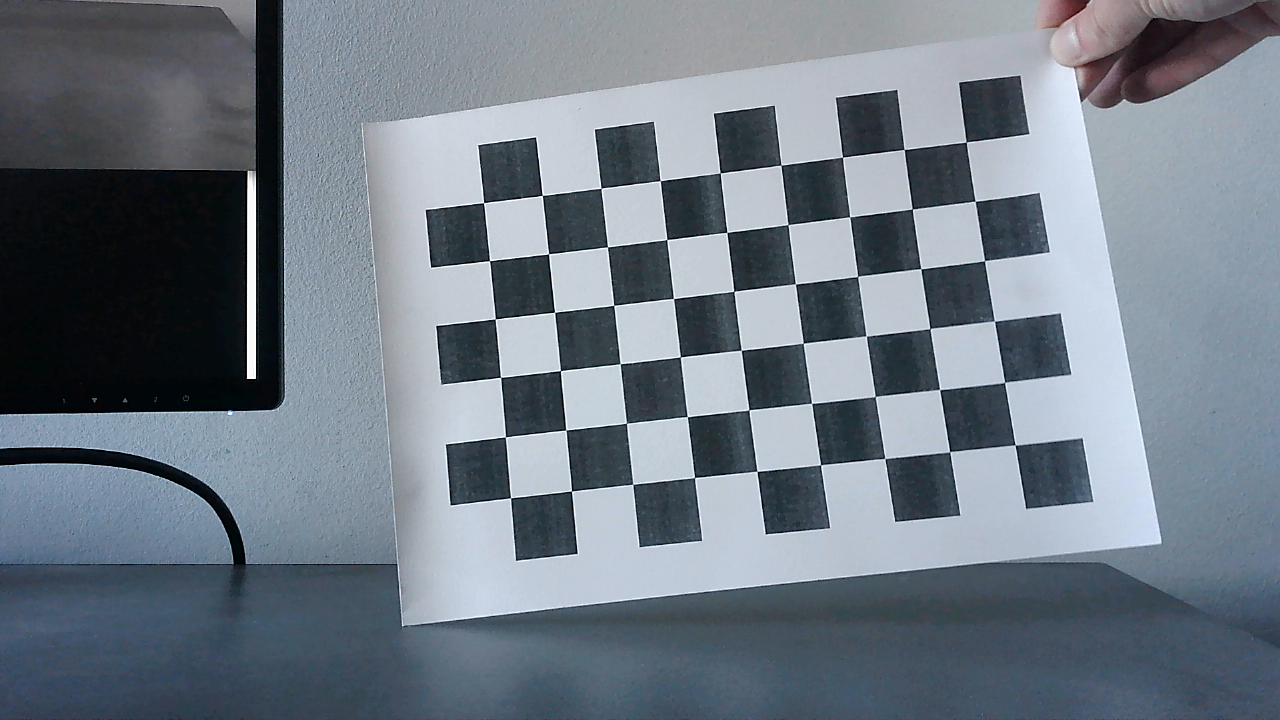

Supplement: Supplementary file 1 [file jimaging-12-00280-s001.zip › Supplementary Materials/first test/Pairs/raw/left/pair_0049_left.png]

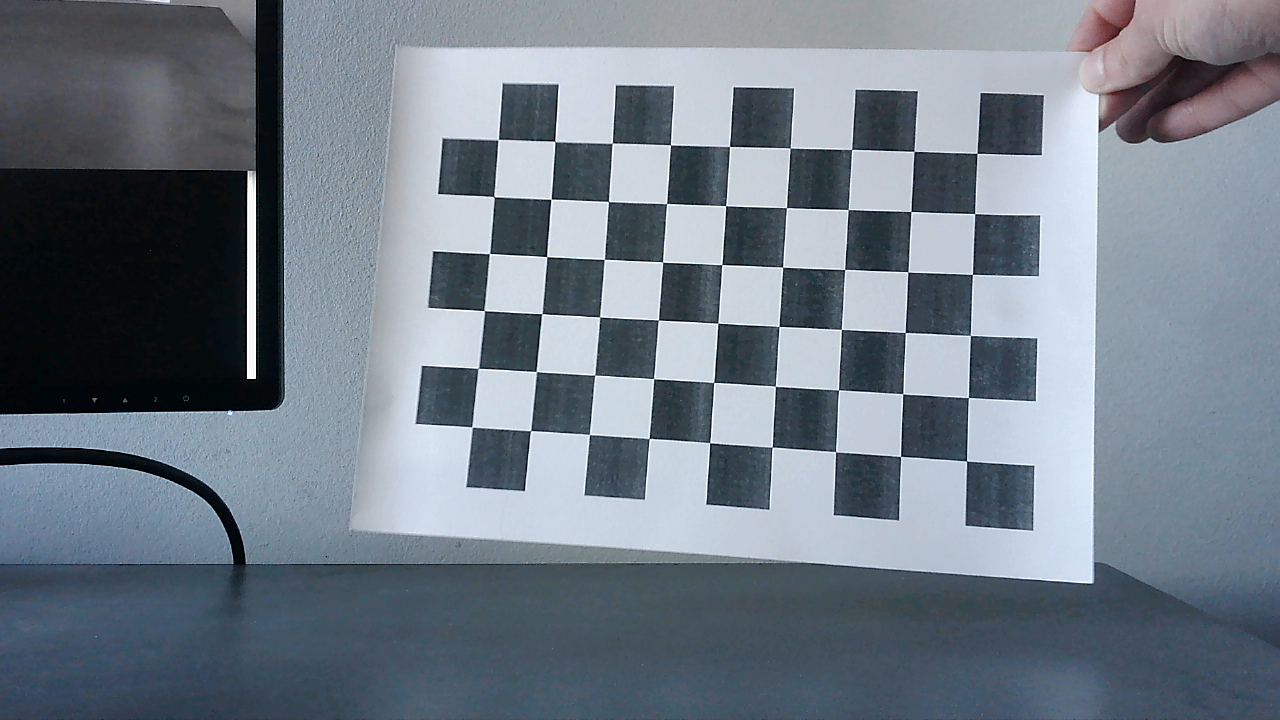

Supplement: Supplementary file 1 [file jimaging-12-00280-s001.zip › Supplementary Materials/first test/Pairs/raw/left/pair_0050_left.png]

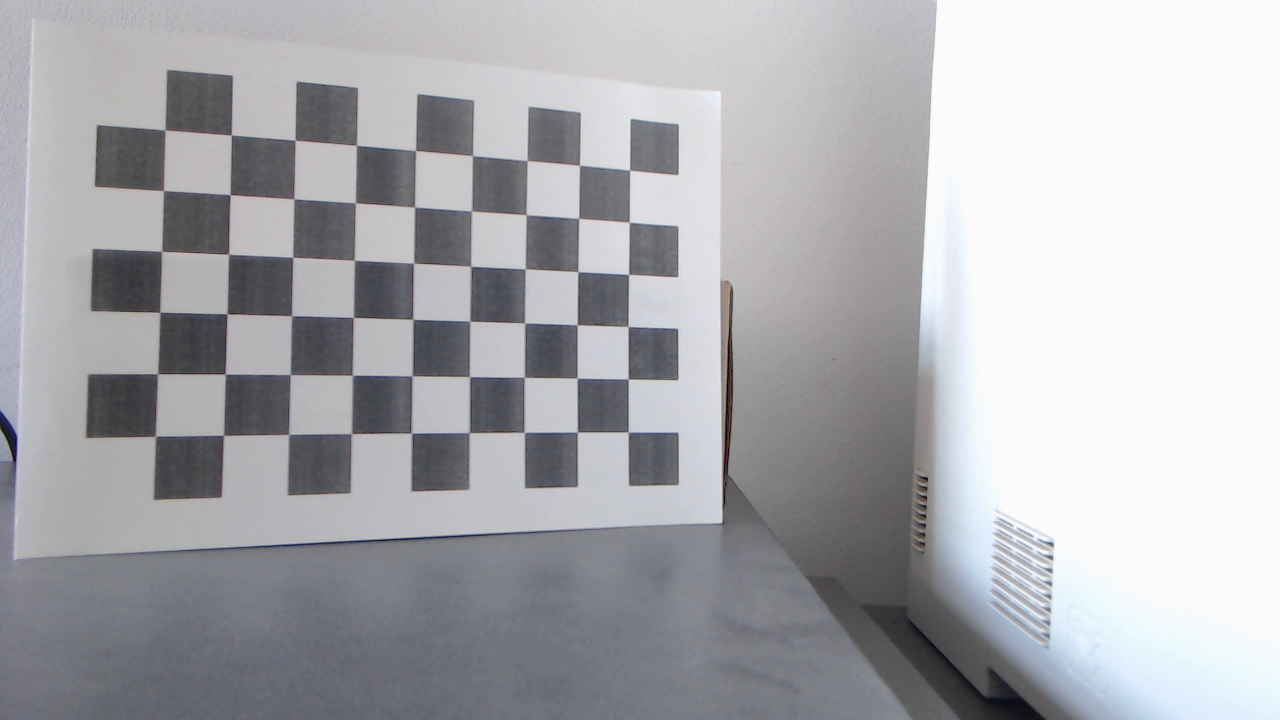

Supplement: Supplementary file 1 [file jimaging-12-00280-s001.zip › Supplementary Materials/first test/Pairs/raw/right/pair_0001_right.png]

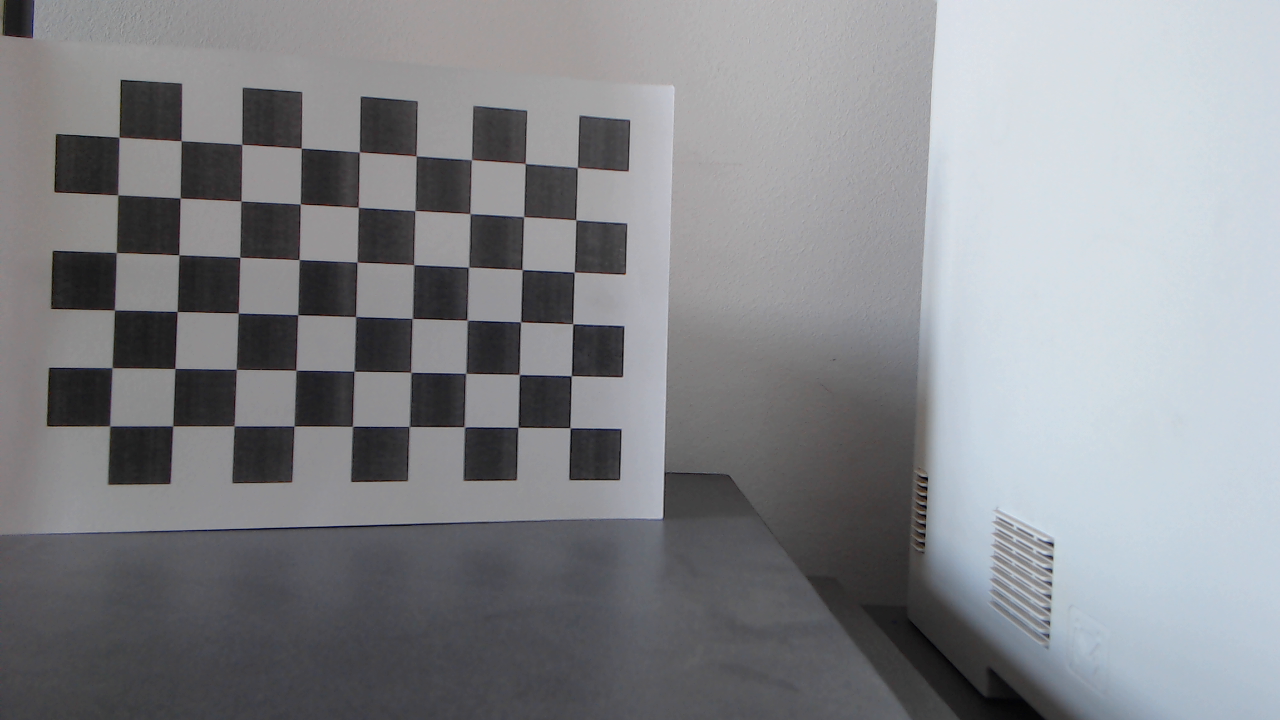

Supplement: Supplementary file 1 [file jimaging-12-00280-s001.zip › Supplementary Materials/first test/Pairs/raw/right/pair_0004_right.png]

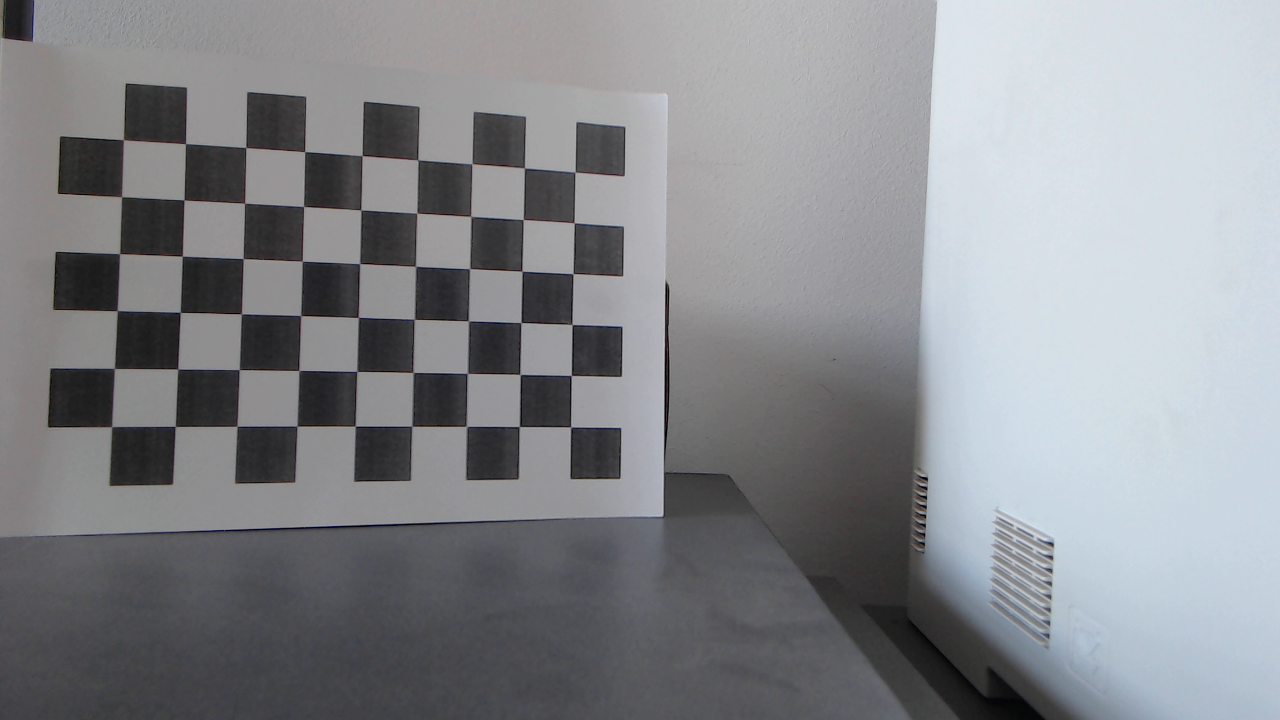

Supplement: Supplementary file 1 [file jimaging-12-00280-s001.zip › Supplementary Materials/first test/Pairs/raw/right/pair_0007_right.png]

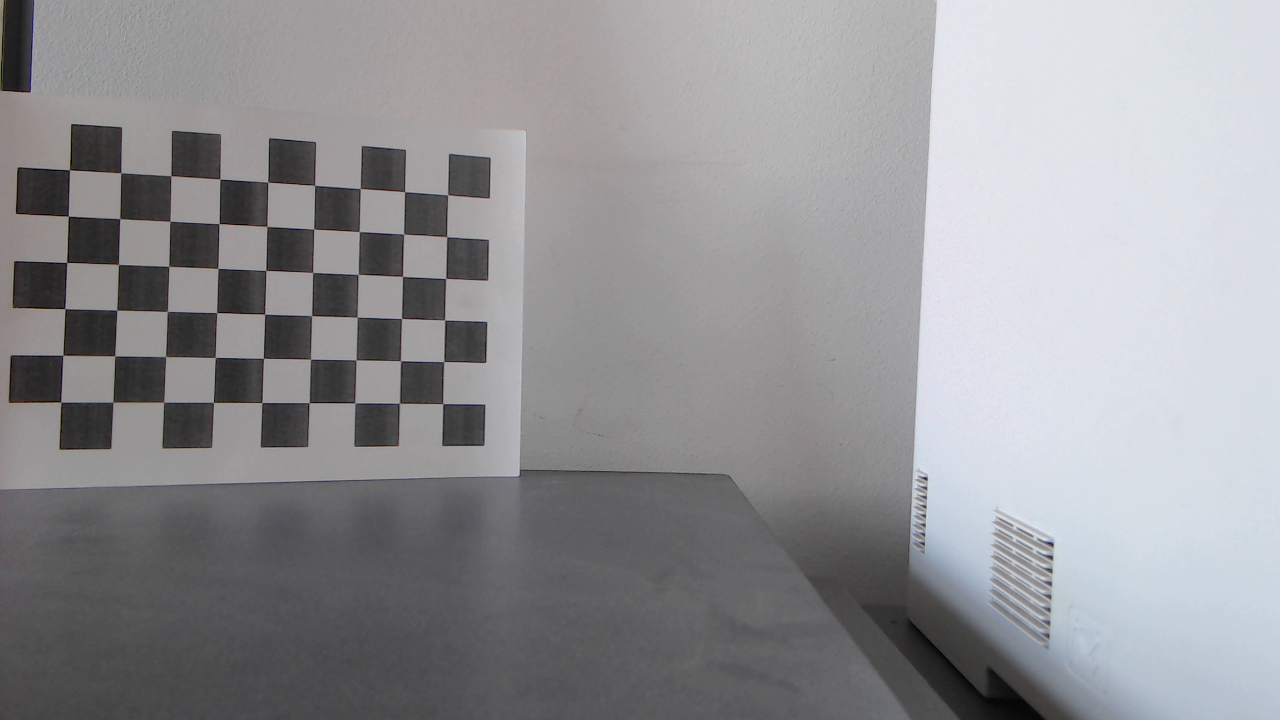

Supplement: Supplementary file 1 [file jimaging-12-00280-s001.zip › Supplementary Materials/first test/Pairs/raw/right/pair_0010_right.png]

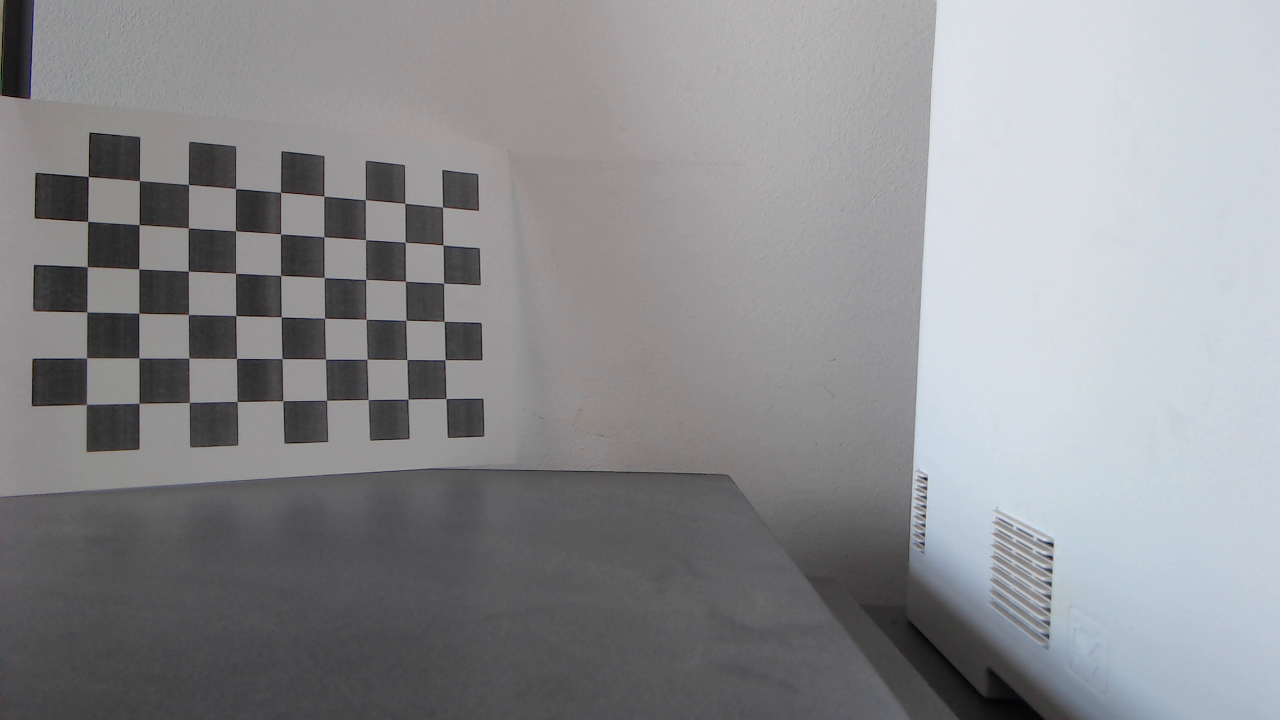

Supplement: Supplementary file 1 [file jimaging-12-00280-s001.zip › Supplementary Materials/first test/Pairs/raw/right/pair_0013_right.png]

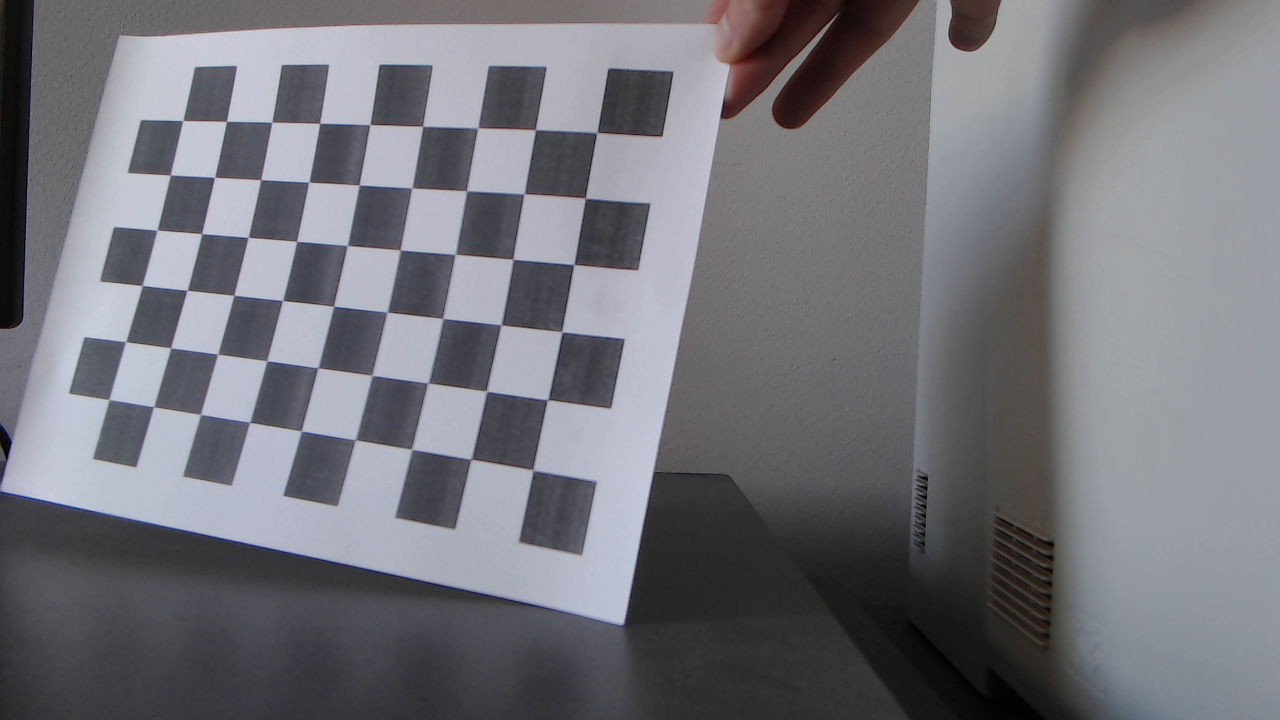

Supplement: Supplementary file 1 [file jimaging-12-00280-s001.zip › Supplementary Materials/first test/Pairs/raw/right/pair_0016_right.png]

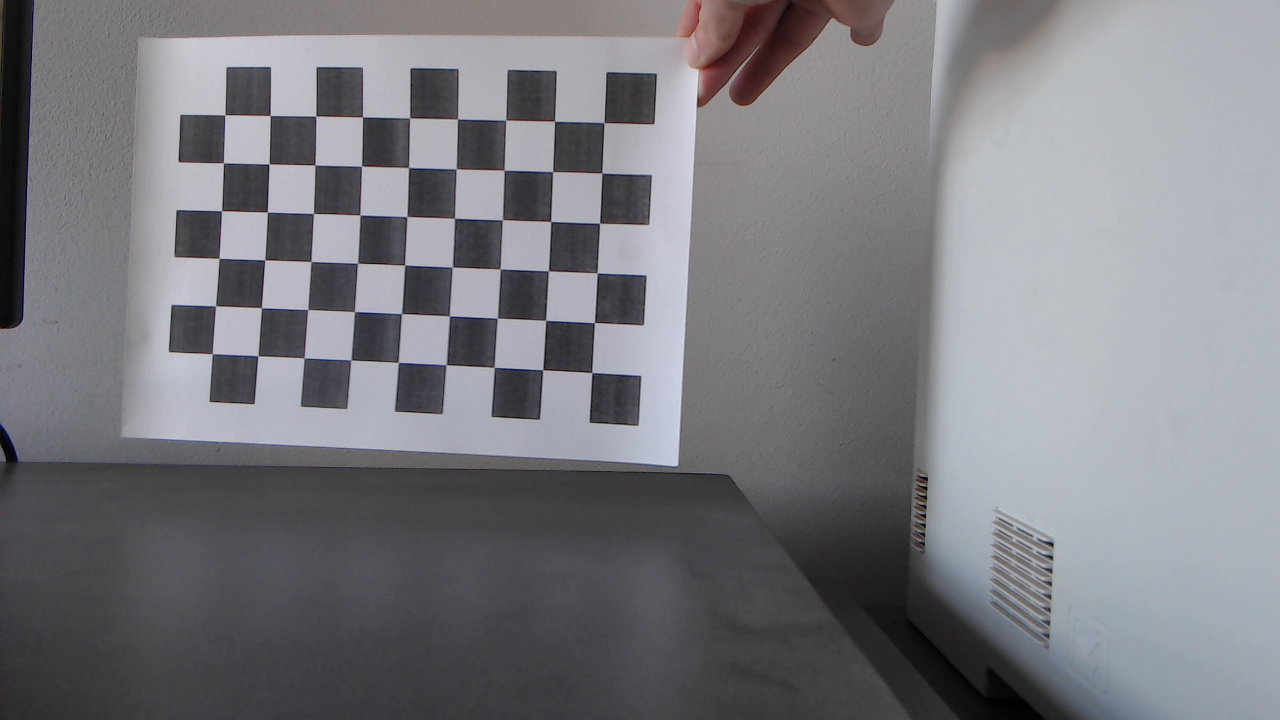

Supplement: Supplementary file 1 [file jimaging-12-00280-s001.zip › Supplementary Materials/first test/Pairs/raw/right/pair_0019_right.png]

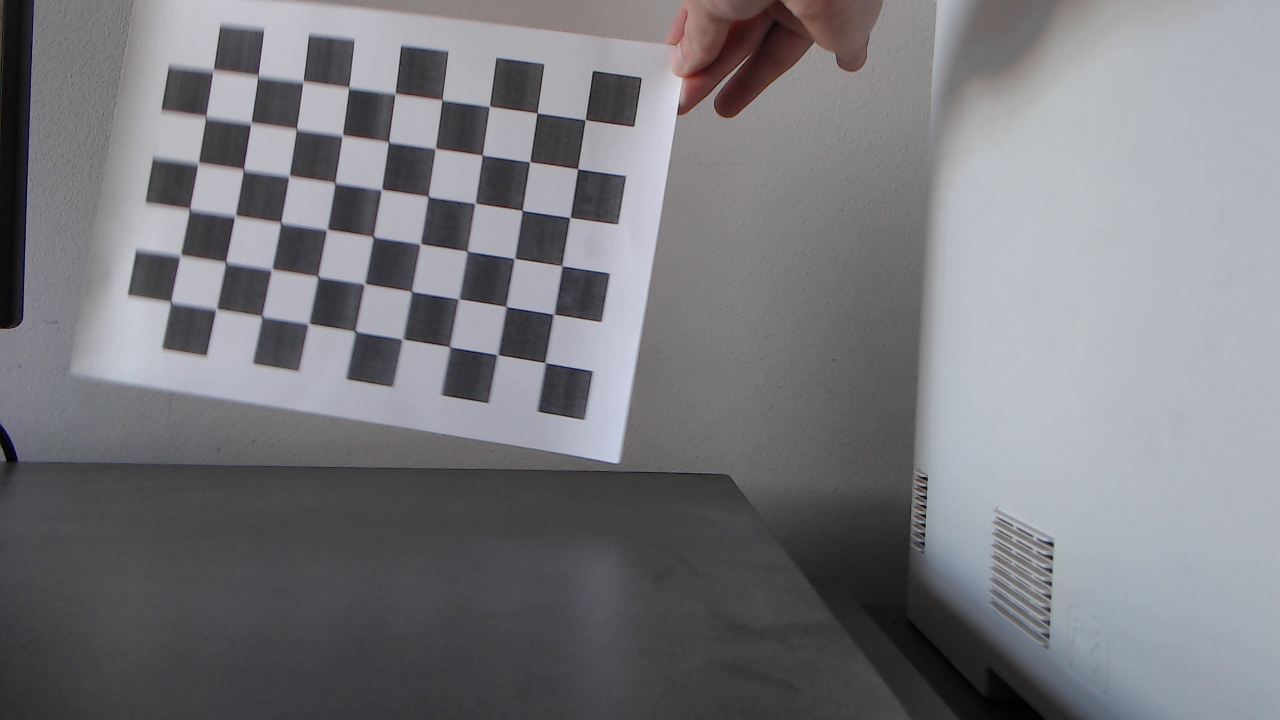

Supplement: Supplementary file 1 [file jimaging-12-00280-s001.zip › Supplementary Materials/first test/Pairs/raw/right/pair_0020_right.png]

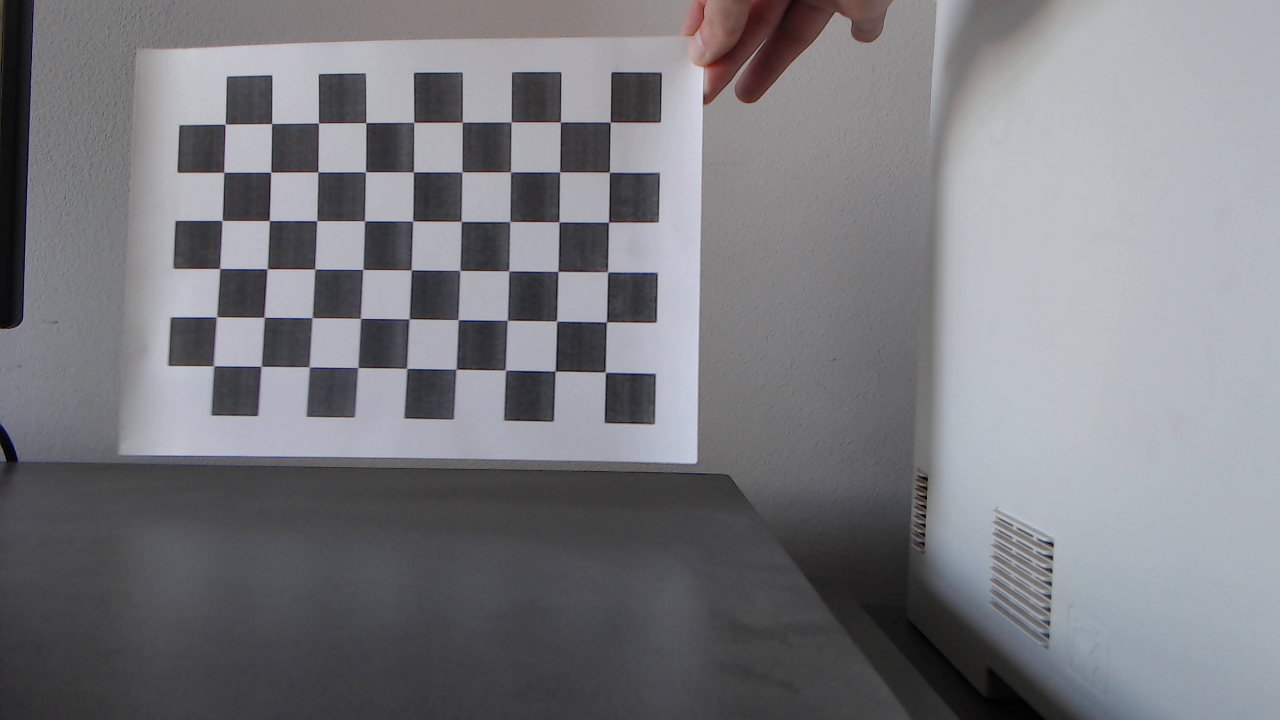

Supplement: Supplementary file 1 [file jimaging-12-00280-s001.zip › Supplementary Materials/first test/Pairs/raw/right/pair_0021_right.png]

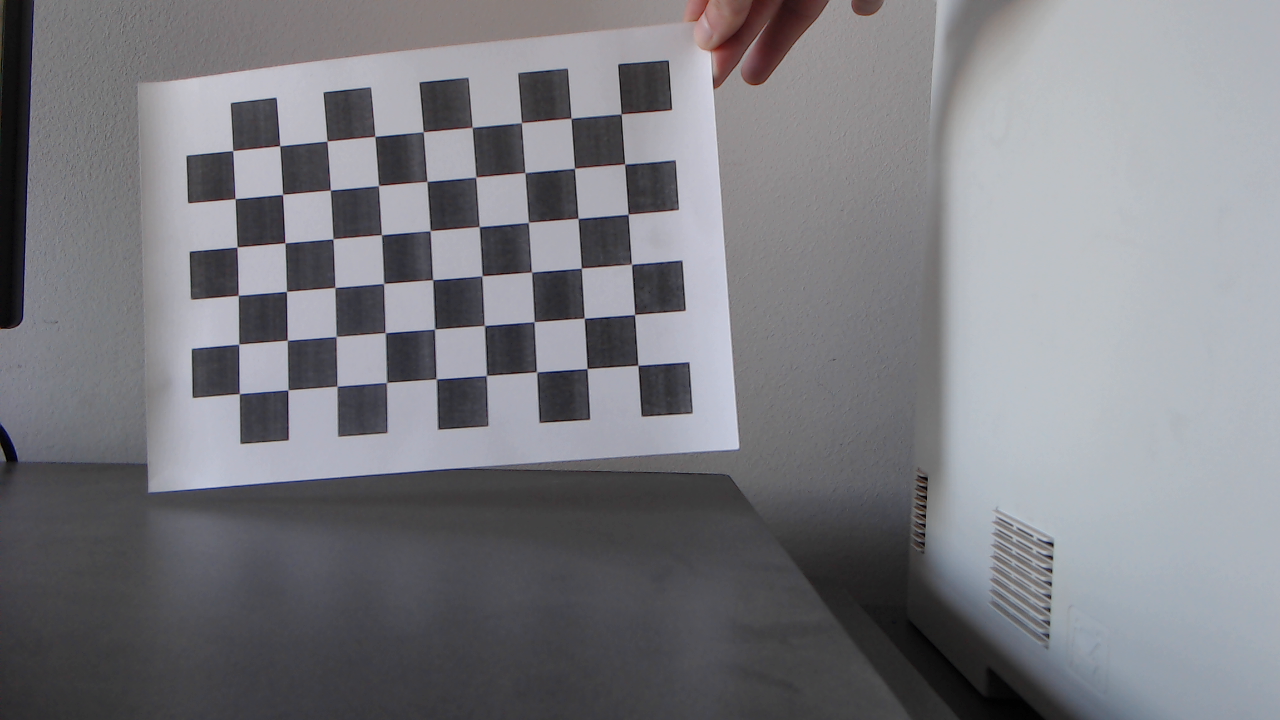

Supplement: Supplementary file 1 [file jimaging-12-00280-s001.zip › Supplementary Materials/first test/Pairs/raw/right/pair_0022_right.png]

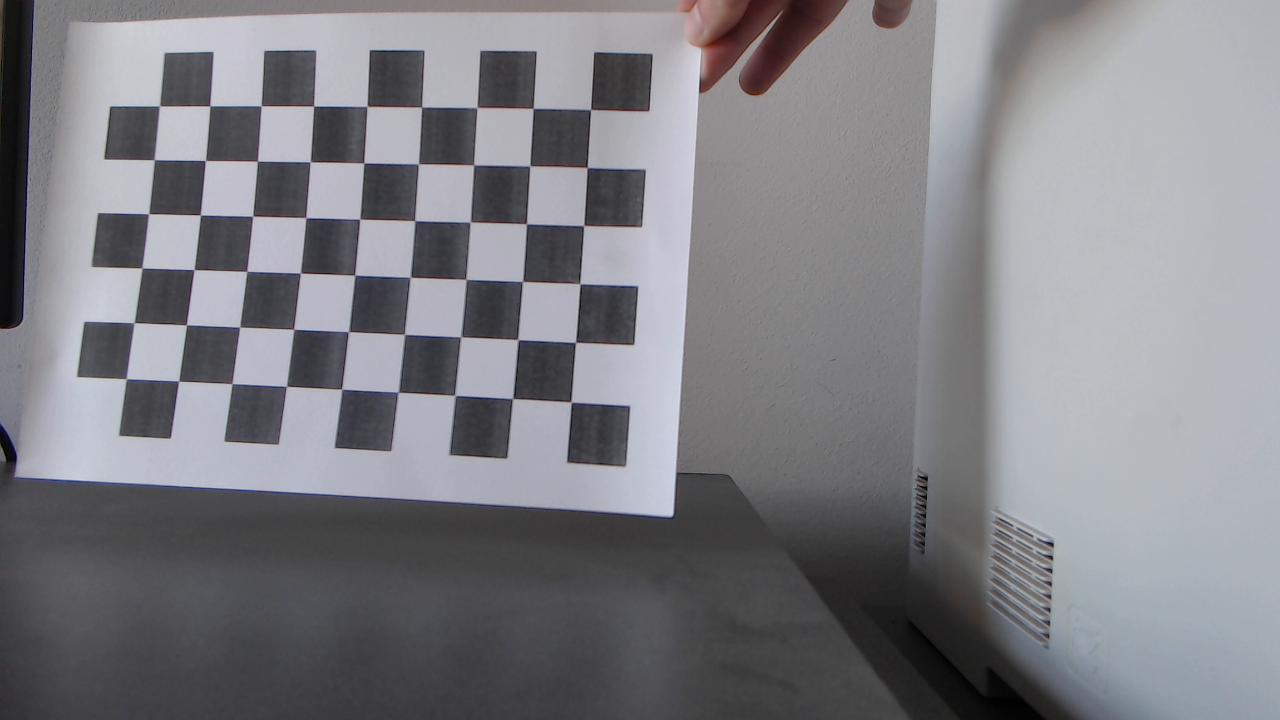

Supplement: Supplementary file 1 [file jimaging-12-00280-s001.zip › Supplementary Materials/first test/Pairs/raw/right/pair_0023_right.png]

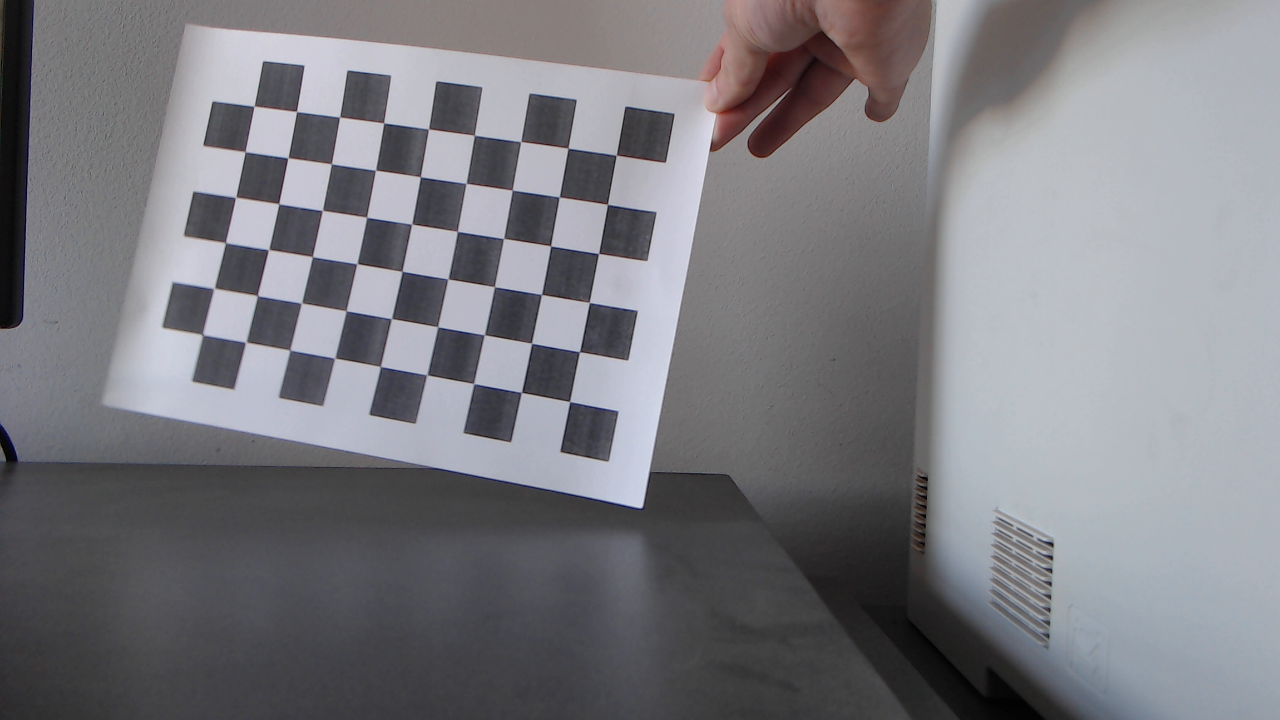

Supplement: Supplementary file 1 [file jimaging-12-00280-s001.zip › Supplementary Materials/first test/Pairs/raw/right/pair_0024_right.png]

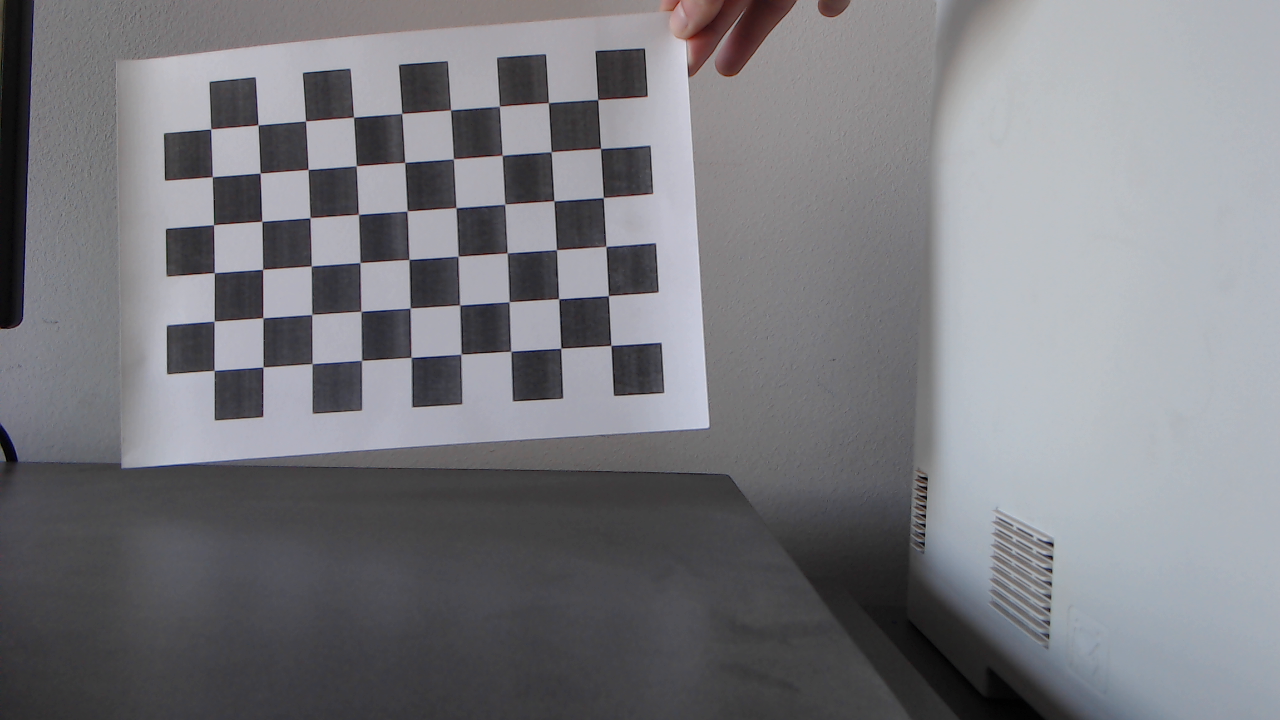

Supplement: Supplementary file 1 [file jimaging-12-00280-s001.zip › Supplementary Materials/first test/Pairs/raw/right/pair_0025_right.png]

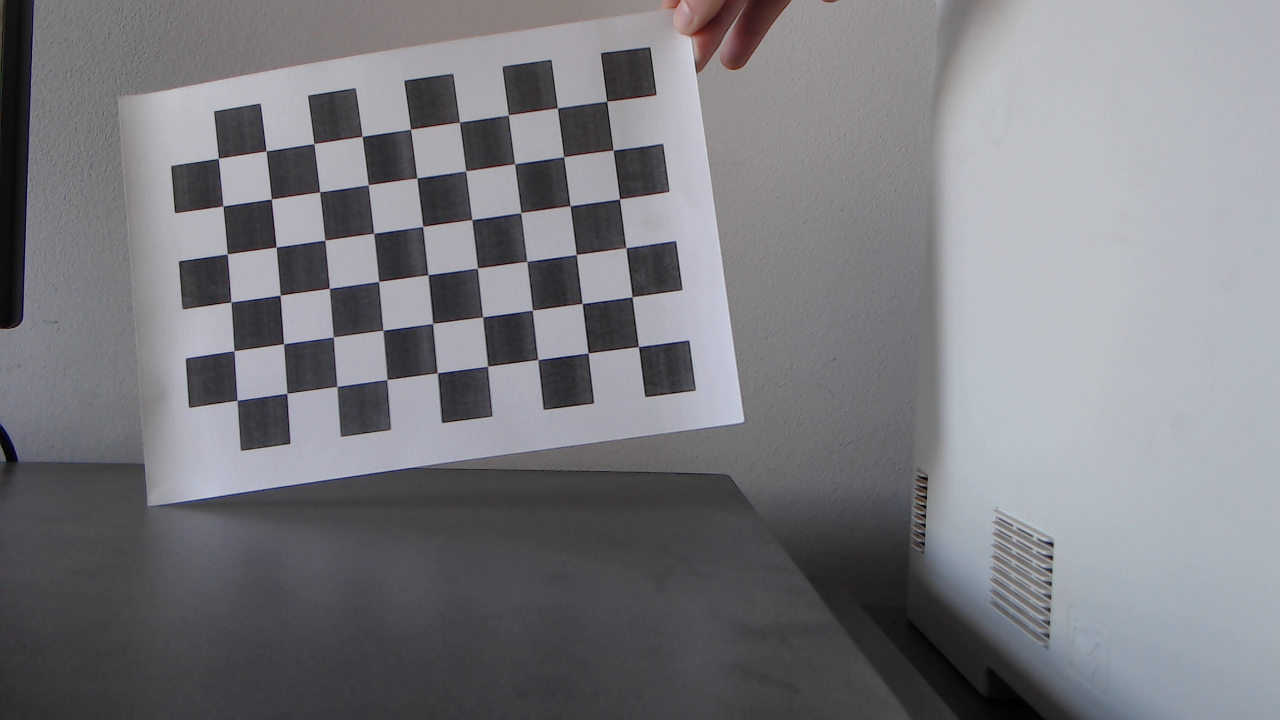

Supplement: Supplementary file 1 [file jimaging-12-00280-s001.zip › Supplementary Materials/first test/Pairs/raw/right/pair_0026_right.png]

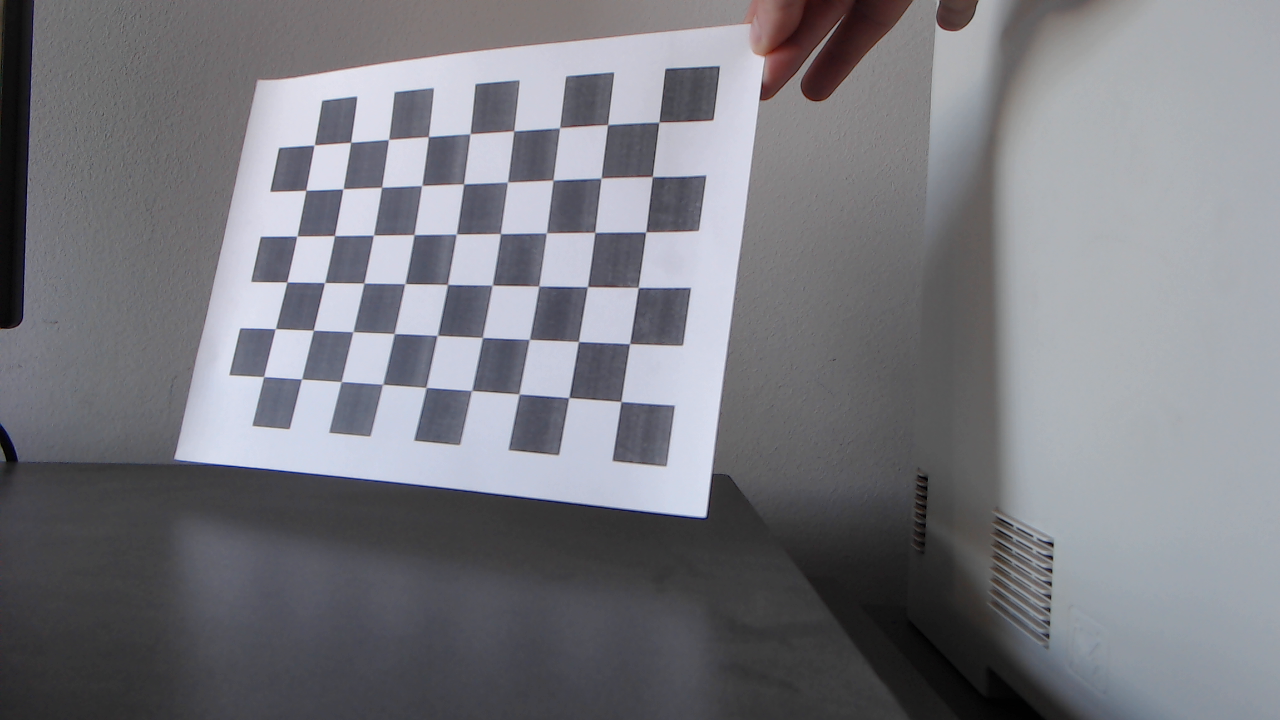

Supplement: Supplementary file 1 [file jimaging-12-00280-s001.zip › Supplementary Materials/first test/Pairs/raw/right/pair_0027_right.png]

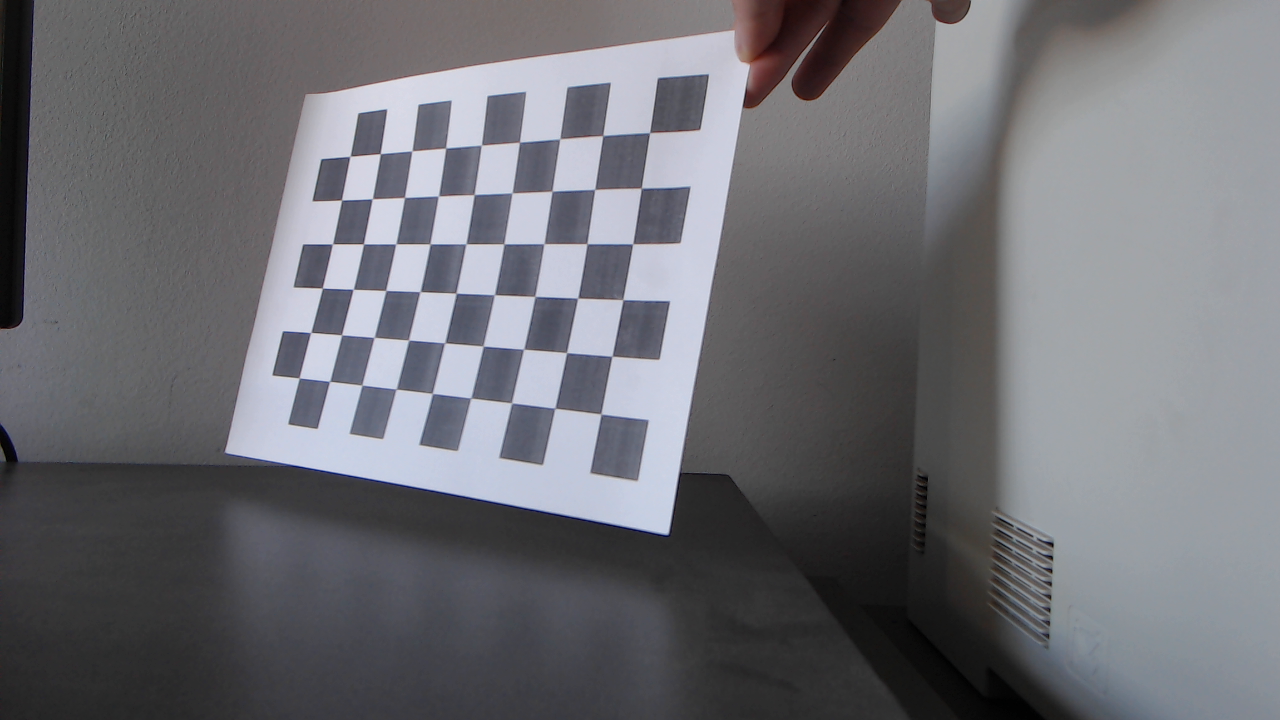

Supplement: Supplementary file 1 [file jimaging-12-00280-s001.zip › Supplementary Materials/first test/Pairs/raw/right/pair_0028_right.png]
